# Lewis‐Acidic Properties and Catalytic Behavior of Hydroboration‐ and Diolysis‐Derived NHC‐Stabilized Borenium Cations

**DOI:** 10.1002/chem.71035

**Published:** 2026-05-06

**Authors:** Michał Jakubczyk, Michaela Buziková, Matouš Kloda, Miroslava Litecká, Karel Škoch

**Affiliations:** ^1^ Department of Materials Chemistry, Institute of Inorganic Chemistry Czech Academy of Sciences Husinec‐Řež 1001 Czech Republic

**Keywords:** borenium, carbene ligands, hydroboration, Lewis acid, TM‐free catalysis

## Abstract

In this work, we report the synthesis and characterization of a series of thirteen N‐heterocyclic carbene‐stabilized borenium cations featuring varied steric and electronic properties of the supporting NHC ligands (IMe_2_, IMes, SIMes). The series comprises borenium species bearing distinct substituents, including hydrides, carbon‐based groups (sp^3^‐ and sp^2^‐hybridized), and diol‐derived moieties forming five‐ to seven‐membered rings. The Lewis acidity was quantified in terms of Gutmann's Acceptor Number (*AN*), revealing that most of the prepared boreniums exhibit higher acidity than the benchmark Lewis acid B(C_6_F_5_)_3_. These borenium species were subsequently evaluated as catalysts in two model transformations—homocoupling of diazo derivatives and hydrosilylation of acetophenones. The markedly different catalytic behavior observed across the series highlights the decisive influence of the structural and electronic factors on the global Lewis acidity of the boron center and, consequently, on its catalytic performance.

## Graphical abstract

A series of thirteen (N‐heterocyclic carbene) NHC‐stabilized borenium cations is synthesized from NHC→BH_3_ complexes via hydroboration and condensation with diols. The novel compounds are fully characterized. Eight crystal structures are presented. Kinetic and NMR data show how the structural features translate to catalytic activity in hydrosilylation and diazo‐homocoupling reactions. Particularly, the presence of a dioxaboracycle.

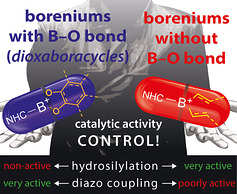

## 1 Introduction

**FIGURE 1:**
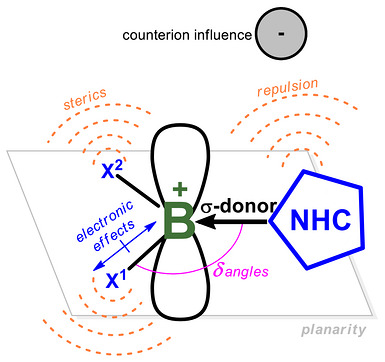
Factors controlling the resultant Lewis acidity of the boron center.

Tricoordinate boron compounds have been for long recognized as archetypal Lewis acids. In a specific case given, the resultant Lewis acidity‐derived reactivity relies on factors that can be separately described, even if they cannot be separately assessed experimentally (Figure 1). For example, it has been evidenced that the cyclic boronic esters (of diols) display higher Lewis acidity strength than the corresponding boronic acids (the p*K*_a_ lowers by about 2–4 units from acid to ester) [1, 2, 3]. This can be explained by the restricted rotation around the B‐O bonds in cyclic esters, preventing the oxygen lone pairs from optimal alignment with the empty boron p‐orbital. A different, complementary explanation associates Lewis acidity strength with the energy of geometry reorganization from flat planar to tetrahedral upon adduct formation [4]. Formation of a five‐membered cyclic ester results in narrowing of the O‐B‐O angle with respect to the idealized 120° in non‐cyclic boronic acids (and non‐cyclic esters). In turn, this results in lowering of the reorganization energy, making it easier to adopt tetrahedral geometry (and form an adduct) with any given Lewis base. This concept formed a basis for the *THC* (tetrahedral boron character) scale, which relies exclusively on structural data [5]. The intrinsic factors controlling the Lewis acidity of the boron center in rigid 1,3,2‐dioxaborolane rings are reflected in dioxaborol‐2‐ylium moieties in some of the structures presented in this work.

One of the most common experimental Lewis acidity assessment methods is the measurement of the acceptor number (*AN*)—utilizing Et_3_PO (TEPO) as a ^31^P NMR probe [6, 7, 8]. The presence of oxygen atoms directly bound to the boron center can affect the *AN*, as exemplified by the series B(OC_6_F_5_)_3_: 88.4 > BC_6_F_5_(O‐C_6_F_5_)_2_: 87.5 > B(C_6_F_5_)_2_OC_6_F_5_: 86.4 > B(C_6_F_5_)_3_: 78.9. This is a consequence of the raising *hardness* and therefore, stronger interaction with the Et_3_PO—a *hard* Lewis base in terms of the Persons’ HSAB theory [9]. The *AN* scale, like all other experimental scales, is dependent on the Lewis base‐partner. Thus, it does not correlate linearly with complementary scales, like computational FIA (fluoride ion affinity), for which a much smaller and charge‐denser F^–^ anion is used to assess the adduct formation energy [10]. The FIA scale also has its shortcomings, particularly in the context of cationic Lewis acids [11].

The chemical space of neutral Lewis acidic organoboron compounds is complemented by the relatively stable cationic species of high Lewis acidity strength. The enhanced electrophilic properties make borenium species promising candidates as catalysts [12, 13, 14]. The behavior and nomenclature of tri‐ and di‐coordinate boron cations were first reviewed by Nöth et al. [15, 16, 17]. In principle, when starting from a neutral tricoordinate boron, displacement of a substituent (anion) by a neutral donating ligand, such as pyridine or lutidine, generates a borenium cation. For this to happen, the neutral donor must be sterically bulky, and the displaced substituent must be a good leaving group (anion), so that the nucleophilic dissociation is favored over Lewis acid‐base adduct formation [17]. In other words, the neutral Lewis base is separating the cationic boron from direct interaction with the weakly coordinating anion (WCA), and a salt is formed. In the case of the NHC‐coordinated boranes, the hydride anion takes the role of a leaving group, being substituted by a weakly coordinating anion, resulting in a tricoordinate NHC→borenium cation in a salt with a WCA [18]. The reaction is driven by the thermodynamic stability of the byproduct (for example, H_2_ gas or triphenylmethane). ^11^B NMR studies reveal that, when triflic acid is used (HOSO_2_CF_3_), the boron atom is often firmly bonded and cannot be distinguished as a cation [19]. In the case of triflimic acid (HNTf_2_; HN(SO_2_CF_3_)_2_), the direct interaction is observed (for example, by diffractometry), but the compound acts as an ionic pair. In the case of Ph_3_C^+^ B(C_6_F_5_)_4_^–^, the two boron centers (cationic and anionic) have no possibility of contact [19].

The hydride abstraction strategy can also be used to generate single B‐H species, like the [IMes**→**B(H)C_6_F_5_]^+^ [B(C_6_F_5_)_4_]^–^ salt [20]. Chiral NHC‐stabilized borenium variants have been obtained as camphor‐ and oxazole‐ring analogues with 9‐borabicyclo[3.3.1]nonane (9‐BBN) and also from (+)‐diisopinocampheylborane, among others [21]. Cyclized, planarized boreniums can also be obtained by consecutive hydride abstraction/dehydrogenative borylation cycles from *N*‐benzyl substituted NHC→BH_3_ adducts [22]. NHC‐stabilized borenium cations can be also accessed by other methods, e.g., from diarylboron halides [23, 24] and by halogen abstraction [25] or substitution [26].

We have been interested in the synthetic applicability of simple NHC→BH_2_^+^ WCA^–^ species in the context of their reactivity in hydroboration. We envisioned that those intermediates could be used as starting points to obtain diverse borenium compounds in which the Lewis acidity and catalytic properties originate from the structure of the hydroboration and diolysis partners, in addition to the effects originating from a specific NHC ligand (and possibly the WCA counterion). Herein, we present a series of tricoordinate NHC‐stabilized borenium cations derived from IMes→BH_3_, SIMes→BH_3_, and IMe_2_→BH_3_ borane complexes, as salts with WCAs (IMes = 1,3‐dimesitylimidazol‐2‐ylidene, SIMes = 1,3‐dimesitylimidazolidin‐2‐ylidene, IMe_2_ = 1,3‐dimethylimidazol‐2‐ylidene). The strong σ‐electron donor character of NHCs assures the stability of such complexes. *AN* of the prepared compounds is assessed in DCM‐d_2_, and the catalytic activity is screened in two model reactions. Obtained compounds are structurally diverse, including bulky groups, unsaturated bonds, and dioxaboracycles (cationic analogues of cyclic boronic esters). They also differ in the donor strength of the NHC moiety.

## 2 Results and Discussion

### 2.1 Synthesis

**SCHEME 1:**
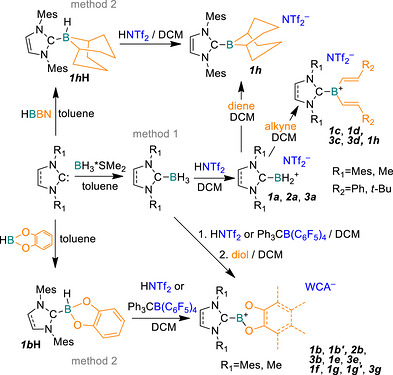
General synthetic scenarios used to obtain intermediates ***1a***, ***2a***, ***3a***, ***1b*H**, and ***1h*H**, and the title compounds ***1b***, ***1b’***, ***1c***, ***1d***, ***1e***, ***1f***, ***1** **g***, ***1g'*** *, **1** **h***, ***2b***, ***3b***, ***3c***, ***3d***, ***3e***, and ***3g***.

The title borenium compounds were obtained via hydroboration and diolysis (hydride‐to‐alkoxy substitution with a diol) in reactions between an NHC→BH_2_^+^ NTf_2_^–^ salt and unsaturated alkanes or diols, respectively (Scheme 1, method 1). Tetracoordinate NHC→BH_3_ species were obtained by typical methods; IMes→BH_3_ from isolated, free persistent carbene reacted with BH_3_·SMe_2_ [27], SIMes→BH_3_ from carbene generated in situ and IMe_2_→BH_3_ from doubly *N*‐alkylated imidazolium treated with NaBH_4_ [28]. These compounds are stable to moisture in air, unable to hydroborate, and engage in the reaction only after abstraction of one hydride anion by a WCA‐conjugated Brønsted acid, such as triflimic acid HNTf_2_ [29, 30]. Obtained NHC→BH_2_^+^ NTf_2_^–^ salts are white powders and can be stored for months inside an Ar‐filled glovebox with minimal to no decomposition. Compounds ***1b’*** and ***1g’*** were synthesized by using Ph_3_C^+^ B(C_6_F_5_)_4_^–^ in place of HNTf_2_, with the generation of Ph_3_CH byproduct, which can be removed from the mixture by washing with hexane [31].

**SCHEME 2:**
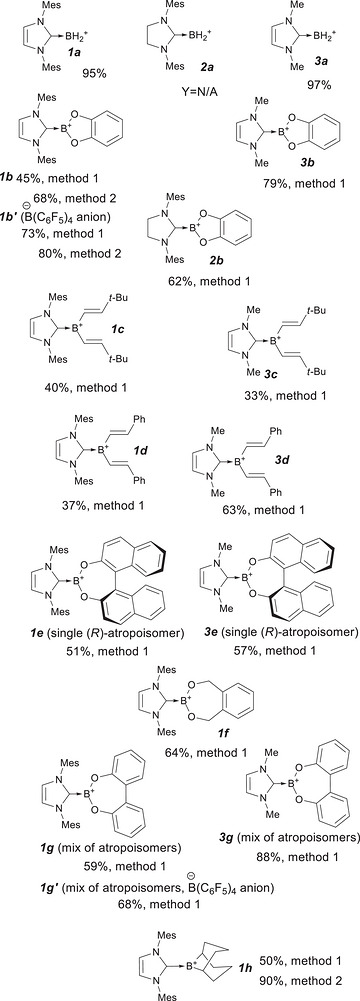
The scope of studied compounds. All boreniums are salts with the NTf_2_^–^ anion unless otherwise indicated. Preparative method and corresponding isolated yield are specified.

The boron center in the IMes→BH_2_^+^ directly interacts with the NTf_2_^–^ anionic species. The single ^11^B NMR wide signal *δ*_B_ = –17.5 ppm (FWHM ≈ 440 Hz) is evidence of a single tetrahedral boron species present. The same is true for the IMe_2_→BH_2_^+^ NTf_2_^–^ derivative. However, the reaction between the saturated SIMes→BH_3_ and 1 eq. HNTf_2_ is not selective, presumably producing a H‐bridged dimer and some unidentified byproducts in addition to the SIMes→BH_2_^+^ NTf_2_^–^. For this reason, we were unable to isolate and purify compound ***2a*** and excluded it from the assessment of catalytic performance. Nonetheless, upon triethylphosphine oxide (TEPO) addition to the sample, a single ^31^P{^1^H} NMR signal at *δ*_P_ = 84.6 ppm indicates the formation of [SIMes→BH_2_·TEPO]^+^ adduct. The in situ generated SIMes→BH_2_^+^ NTf_2_^–^ mixtures reacted with acetylenes and diols in our protocols resulted further in complicated mixtures that indeed contain some small amounts of the desired products. However, we were unable to isolate and characterize any of those, with the one exception of compound ***2b***.

In the case of derivatives ***1b***, ***1b’*** and ***1*** ***h***, an alternative pathway was also explored (Scheme 1, method 2) [32]. Combining the free carbene with CatBH or BBN results in the neutral NHC→BHR_2_ adduct in the first step (compounds ***1b*H** and ***1h*H**). Abstracting the H^–^ by HNTf_2_ or Ph_3_C^+^ B(C_6_F_5_)_4_^–^ in the second step yields the final product. This method seems to produce fewer impurities, but is limited by the availability of the starting carbene and mono‐hydroborane derivative.

### 2.2 X‐ray Diffractometry

For compounds ***1b***, ***1b’***, ***1d***, ***1f***, ***2b***, ***3b***, ***3e*,** and ***1h*H**, single crystals of sufficient quality for X‐ray diffraction were obtained from DCM/hexane mixtures inside the glovebox. Crystal data for all structures, data collection, and details of refinement are given in Table S2 in the Supporting Information.

**FIGURE 2:**
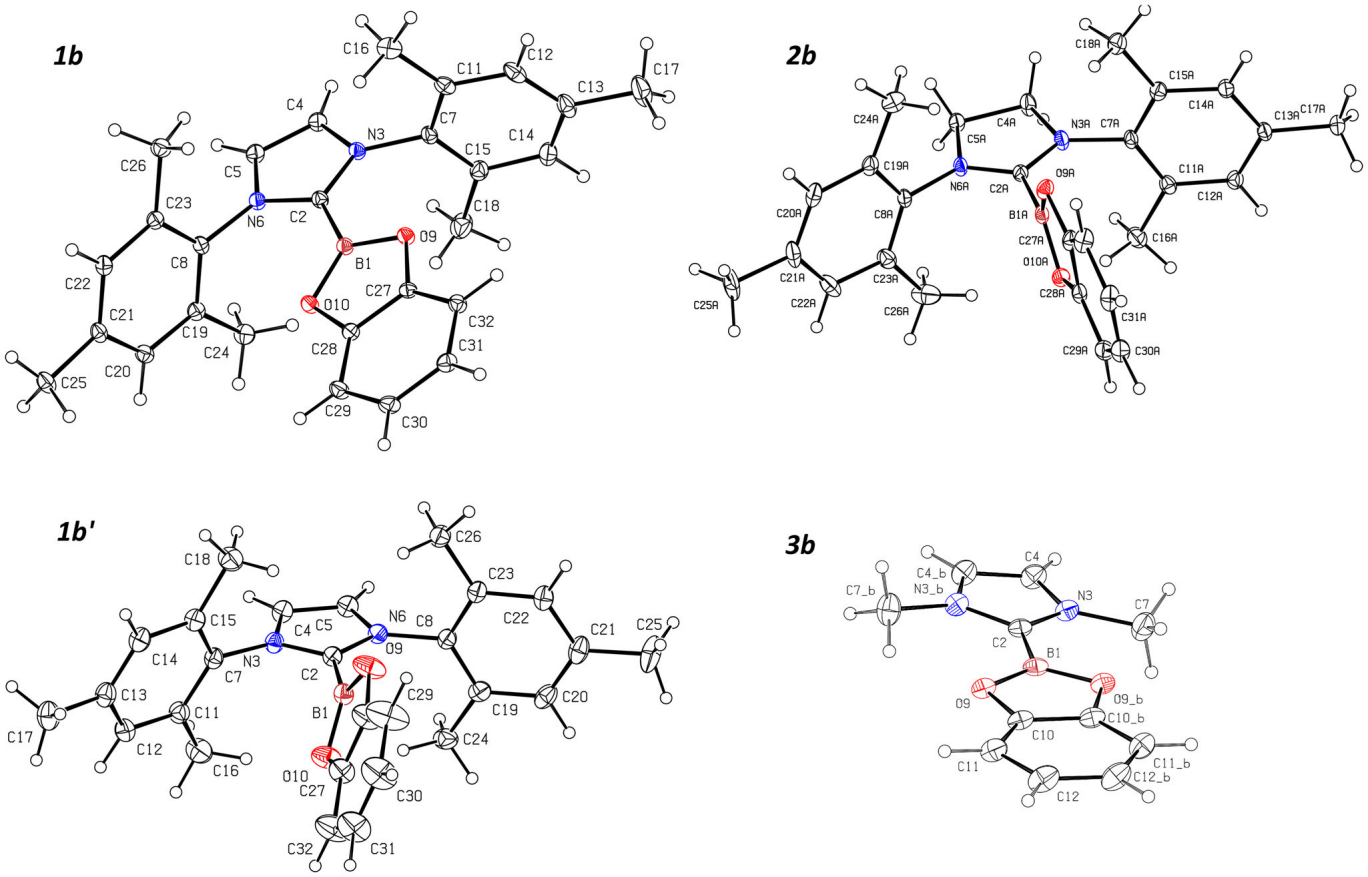
Molecular structure view of the 1,3,2‐benzodioxaborol‐2‐yl moiety‐bearing compounds ***1b***, ***1b’***, ***2b***, and ***3b***. Thermal ellipsoids are shown at the 30% probability level. Anions were omitted for clarity. For compound ***2b***, only one of the two independent molecules is shown. Selected bond lengths (Å) and angles (°): ***1** **b'*** {C2‐B1 1.572(4), C2‐N3‐C7‐C15 74.8(4), N3‐C2‐B1‐O10 64.3(4)}, ***1b*** {C2‐B1 1.561(2), C2‐N6‐C8‐C23 89.6(2), N6‐C2‐B1‐O10 13.2(2)}, ***2b*** {C2A‐B1A 1.583(3), C2A‐N3A‐C7A‐C11A 90.7(3), N3A‐C2A‐B1A‐O9A 89.8(3)}, ***3b*** {C2‐B1 1.547(5), N3‐C2‐B1‐O9 5.9}.

**FIGURE 3:**
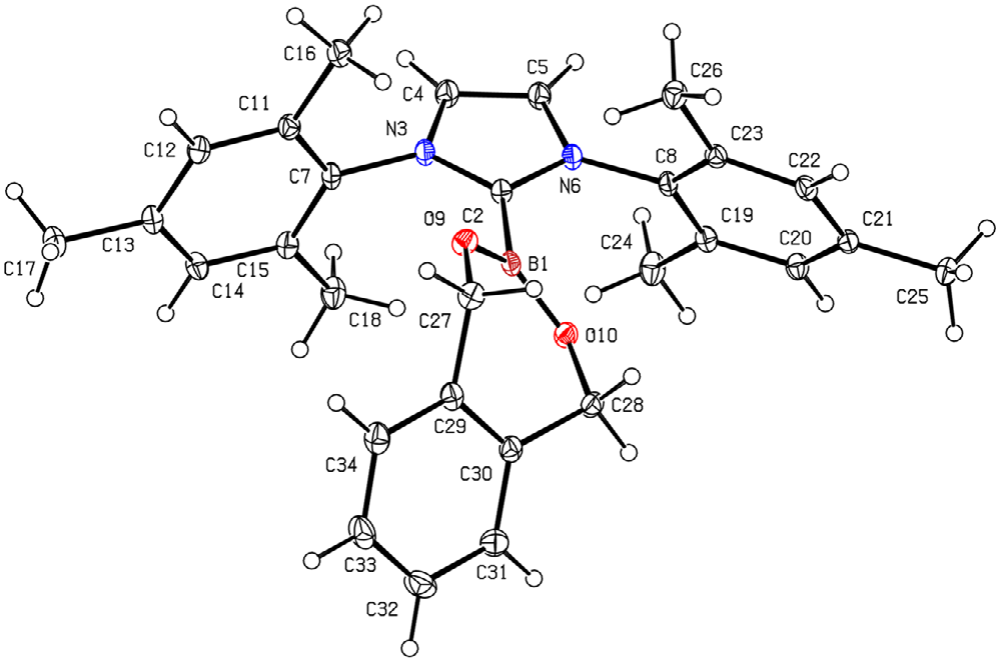
Molecular structure view of the dioxaborepine moiety‐bearing compound ***1f***. Thermal ellipsoids are shown at the 30% probability level. Anion is omitted for clarity. Selected bond lengths (Å) and angles (°): C2‐B1 1.595(2), O10‐B1‐O9 130.6(1), N3‐C2‐B1‐O10 133.5(2).

**FIGURE 4:**
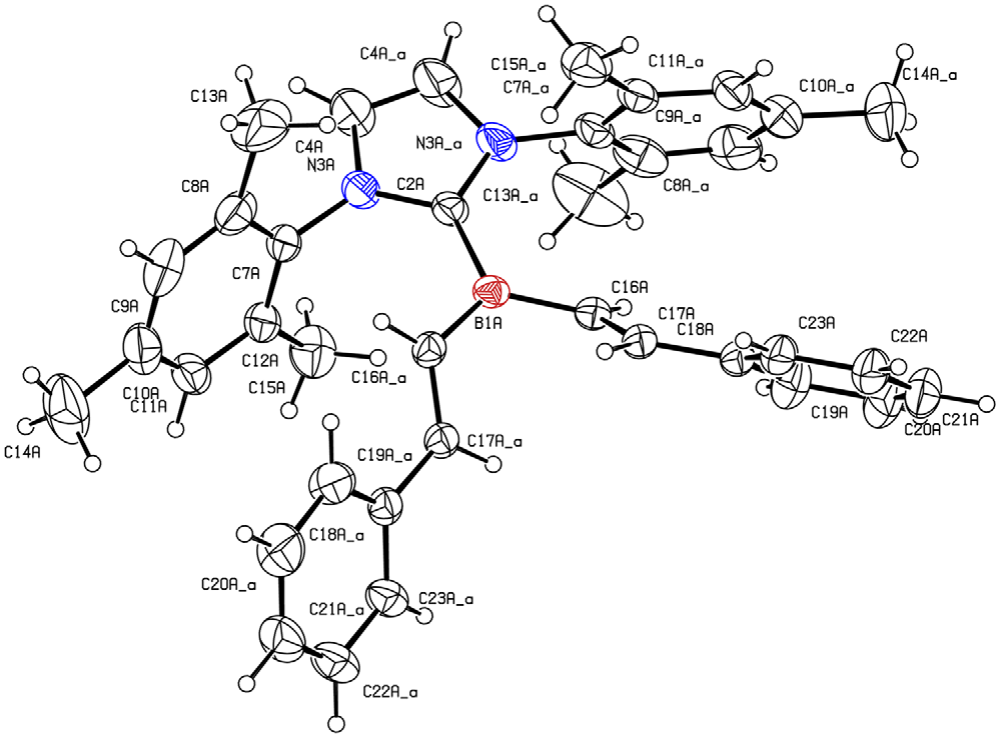
Molecular structure view of compound ***1d***. Thermal ellipsoids are shown at the 30% probability level. Anion and one of the two independent molecules are omitted for clarity. Selected bond lengths (Å) and angles (°): C2A‐B1A 1.580(4), C16A‐B1A‐C16A 124.2, C2A‐N3A‐C7A‐C12A 107.1.

Comparison of parent structures ***1b’***, ***1b***, ***2b*,** and ***3b*** (Figure 2) allows to assess the influence of NHC and anion characters on the molecular conformation in the crystal phase. The shortest B–C_NHC_ bond of 1.547(5) Å for compound ***3b*** shows the extent of steric repulsion from the mesityl‐ substituents to the 1,3,2‐benzodioxaborol‐2‐yl. Particularly, when compared with ***1b*** bond length (1.561(2) Å), for in both cases the dihedral angle between the NHC and the 1,3,2‐benzodioxaborol‐2‐yl moieties is quite low (13.2(2)° and 5.9° for ***1b*** and ***3b***, respectively). On the other hand, the stronger donor properties of SIMes versus IMes result in diminished imidazol‐dioxaborol conjugation and longer B–C_NHC_ bond distance (1.583(3) Å) in the case of ***2b***, what in turn allows the 1,3,2‐benzodioxaborol‐2‐yl to be rotated almost perpendicularly to the NHC ring (89.8(3)°), ultimately resulting in a denser crystal packing. In the case of ***1b’***, the large perfluorinated anion dominates as the source of interactions governing the crystal packing, which results in a dihedral angle around B–C_NHC_ bond of 64.3(4)° (NHC→B bond length of 1.572(4) Å). Regardless of the rotation around the B–C_NHC_ bond, the mesityl‐ moieties retain a perpendicular conformation to the 5‐membered diazacycle in all cases. This is also true for the structures of ***1f*** (Figure 3) and ***1d*** (Figure 4). In the case of ***1f***, the 7‐membered dioxaborepine cycle adopts a chair‐like conformation. The O‐B‐O angle is therefore wider 130.6(1)°) and corresponds to a lower *AN* for this derivative in comparison with the catechol‐derived compounds. This is consistent with the previously mentioned geometry reorganization energy theory [4] and results for parent neutral boronic esters [33]. The structure of ***1d*** reveals B–C_NHC_ bond length of 1.580(4) Å, which is most probably caused by a significant repulsion from the styryl groups against mesityls.

**FIGURE 5:**
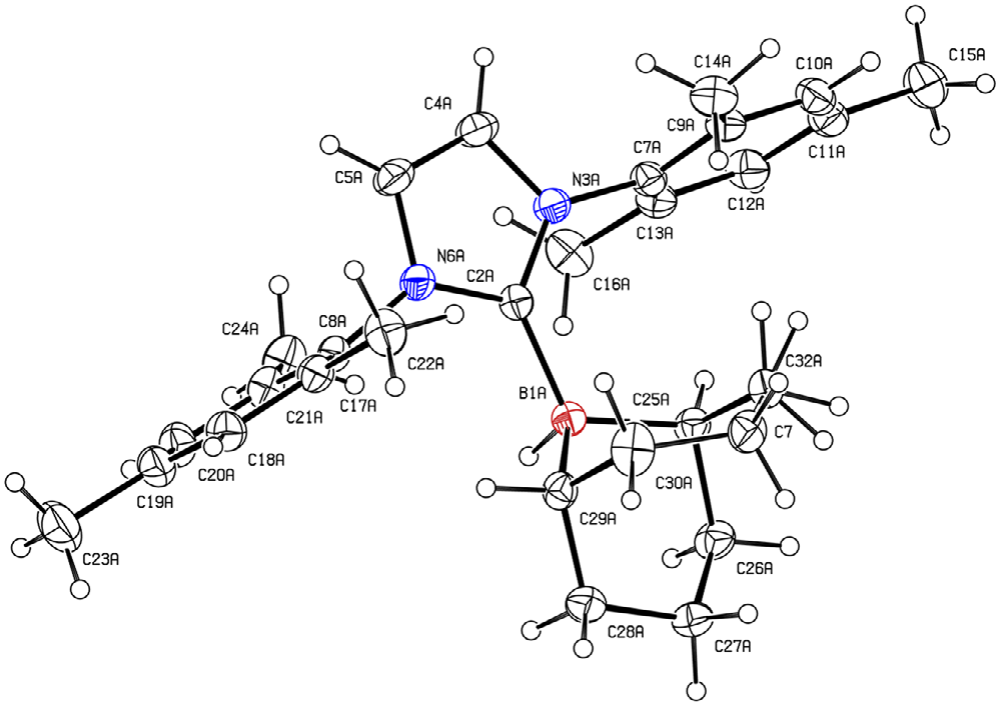
Molecular structure view of compound ***1** **h*** **H**. Thermal ellipsoids are shown at the 30% probability level. Anion and one of the two independent molecules are omitted for clarity. Selected bond lengths (Å) and angles (°): C2A‐B1A 1.638(2), C2A‐N3A‐C7A‐C13A 75.4(2), the B1A boron atom is about 0.474 Å out‐of‐plane defined by atoms C2A, C25A, and C29A.

We have also obtained a single crystal of intermediate ***1h*H** (Figure 5), the hydride adduct of ***1*** ***h***. The B–C_NHC_ bond length is significantly longer than in the borenium structures and equals 1.638(2) Å. The boron atom is about 0.474 Å out‐of‐plane (defined by the three carbon atoms C2A, C25A, and C29A), which is expected to align with after the hydride abstraction in ***1*** ***h***

### 2.3 Determination of the Acceptor Number and FIA, HIA Indices

Most of the obtained title borenium compounds (depicted in Scheme 2) are rather strong Lewis acids, and their *AN* has been assessed in DCM (*AN*_DCM_ = 20.4) at room temperature. Measurement in a solvent of lower *AN*, like benzene (*AN*_benzene_ = 8.2) or THF (*AN*_THF_ = 8.0), was not possible due to solubility issues. The *AN* of the obtained compounds is presented in Table 1.

**TABLE 1:** Characterization of the obtained borenium cations, their Acceptor Number (*AN*) in DCM, Fluoride Ion Affinity and Hydride Ion Affinity (at 298.15 K) in gas phase and in DCM (at the PW6B95(D3)/def2‐QZVPP level of theory—See Supporting Information).

	NHC	R^2^	WCA	^11^B NMR / ppm	TEPO_DCM_ / ppm	*AN*	*FIA* / kJ·mol^−1^	*FIA*_DCM_ ^b^ / kJ·mol^−1^	*HIA* / kJ·mol^−1^	*HIA*_DCM_ ^b^ / kJ·mol^−1^
***1a***	IMes	H	^−^NTf_2_	−17.5	84.5	96.5	714	288	751	325
***1b***		Cat.	^−^NTf_2_	24.9	85.1	97.7	672	251	646	227
***1b'***			^−^B(C_6_F_5_)_4_	24.7	84.7	96.9				
***1c***		(‐CH = CH‐*t*Bu)	^−^NTf_2_	54.2	81.3	89.4	611	192	628	208
***1d***		(‐CH = CH‐Ph)	^−^NTf_2_	53.7	83.2	93.5	629	214	647	231
***1e***		(‐O)_2_BINAP	^−^NTf_2_	25.1	83.1	93.3	656	244	634	227
***1f***		(‐OCH_2_)_2_Ph	^−^NTf_2_	21.2	75.2^a^	75.8^a^	614	194	590	170
***1g***		(‐O)_2_biphenyl	^−^NTf_2_	22.5	82.1	91.0	652	239	633	220
***1g'***			^−^B(C_6_F_5_)_4_	22.1	81.7	90.2				
***1h***		BBN	^−^NTf_2_	83.0	53.7^a^	28.2^a^	641	216	660	235
***2a***	SIMes	H	^−^NTf_2_	−18.0	84.6	96.7	740	319	777	355
***2b***		Cat.	^−^NTf_2_	25.3	85.0	97.6	676	261	653	237
***3a***	IMe_2_	H	^−^NTf_2_	−18.0	85.0	97.6	753	285	788	321
***3b***		Cat.	^−^NTf_2_	25.2	84.5	96.4	681	236	651	209
***3c***		(‐CH = CH‐*t*Bu)	^−^NTf_2_	55.3	81.7	90.2	648	210	668	226
***3d***		(‐CH = CH‐Ph)	^−^NTf_2_	53.2	83.2	93.5	646	216	668	236
***3e***		(‐O)_2_BINAP	^−^NTf_2_	24.9	83.0	93.1	702	266	677	242
***3g***		(‐O)_2_biphenyl	^−^NTf_2_	23.2	82.7	92.4	679	243	656	221
***4a***		B(C_6_F_5_)_3_	*n/a*	59.7	76.6	78.9	442	231	477	264

^a^ For compounds ***1f*** and ***1h***, the *AN* was calculated from the extrapolated ^31^P NMR chemical shift according to a titration procedure (see Supporting Information).

^b^ Solvent effects have been approximated using the PCM model.

The great majority of the title compounds indeed display a very high acceptor number, with the flat catechol‐derived boreniums ***1b***, ***1b’***, ***2b***, and ***3b*** at the top. Larger dioxaborepine ring‐containing examples ***1e***, ***1*** ***g***, ***1g’***, ***3e***, and ***3*** ***g*** have the *AN* a few units lower due to a nonflat 7‐member ring. However, the non‐cyclic boreniums ***1d*** and ***3d*** are on the same level, with the *t*‐Bu derivatives ***1c*** and ***3c*** a few units lower due to steric effect. As expected, the ***1a***, ***2a***, and ***3a*** dihydrido‐boreniums display a very high *AN*, having small ‐H substituents. Compound ***1f*** represents a mid‐point in our series with a lower *AN* of 75.8 caused by the chair‐like conformation of the dioxaborepine ring. Finally, the measurement for the most sterically crowded ***1*** ***h*** reveals *AN* comparable with the solvent. Moreover, for ***1f*** and ***1*** ***h***, we have observed a dependence of *δ*_P_ on the TEPO:LA ratio, for those cases the *AN* value was assessed in a titration experiment. For further details, see Supporting Information.

The ^11^B NMR chemical shift for the title compounds corresponds with the typical values for tricoordinated boreniums, and it does not correlate with the *AN* results. However, in the case of dihydridoboreniums ***1a***, ***2a*,** and ***3a***, the *δ*_B_  =  –18 ppm, which is consistent with a tetrahedral coordination sphere (B‐anion contact in solution). For the oxygen‐containing boracycles, the ^11^B NMR is measured around *δ*_B_ = 22–25 ppm, which is indicative of flat planar geometry, where boron is connected to oxygen heteroatoms. The presence of oxygen atoms negates any electron‐withdrawing effect from the aromatic moieties. In contrast, boreniums ***1c***, ***1d***, ***3c***, and ***3d*** display ^11^B around *δ*_B_ = 55 ppm, indicating an influence of the unsaturated substituents directly connected to the boron atom. ^11^B NMR *δ*_B_ = 83 ppm for compound ***1*** ***h*** is typical for alkyl‐substituted boron species [34].

The FIA and HIA values were calculated at the DFT level of theory in the gas phase and DCM according to the methodology specified by Greb et al. (Table 1) [35, 36]. FIA for the benchmark compound ***4a*** (B(C_6_F_5_)_3_) was calculated to be 442 kJ·mol^−1^, which is an acceptable result considering the computational effort (in comparison to the value of 448.1 kJ·mol^−1^ obtained at the DLPNO‐CCSD(T)/cc‐pVQZ level) [35]. Similarly, HIA for ***4a*** (our work: 477 kJ·mol^−1^, 481 kJ·mol^−1^ at DLPNO‐CCSD(T)/aug‐cc‐pVQZ) [36]. Both indices are highly dependent on the volume of substituents present, emphasizing the cooperative steric effect of the mesityl‐ and R^2^ substituents. The origin of this effect might be connected more to the intramolecular repulsion between moieties (rotational obstruction) necessary for the trigonal‐to‐tetrahedral geometry conversion (upon adduct formation) and less dependent on the repulsion toward the anion partner.

Gas phase FIA values for the R^2^ = Cat. variants favor the ***3b*** (FIA = 681 kJ·mol^−1^) by few kJ·mol^−1^ over ***1b*** (FIA = 672 kJ·mol^−1^) and ***2b*** (FIA = 676 kJ·mol^−1^), whereas ***3c*** (FIA = 648 kJ·mol^−1^) and ***3d*** (FIA = 646 kJ·mol^−1^) are about 17–19 and about 36–37 kJ·mol^−1^ over ***1d*** (FIA = 629 kJ·mol^−1^) and ***1c*** (FIA = 611 kJ·mol^−1^) respectively. Quite a high value for ***3e*** (702 kJ·mol^−1^) is surprising considering the skewed 7‐membered dioxaboracycle. The equally unexpected high value of 641 kJ·mol^−1^ for ***1*** ***h*** results from the small size of fluoride in comparison with TEPO. FIA values for the remaining variants present as expected and are in qualitative agreement with the measured *AN*. The difference between gas phase FIA and HIA reflects the properties of the anions. In all cases where boron is included in a dioxaboracycle, the HIA < FIA by about 20–30 kJ·mol^−1^. For the remaining borenium cations, this trend is reversed (HIA > FIA by about 17–23 kJ·mol^−1^), and the largest differences for the IMe_2_→BH_2_^+^ cations are around 37 kJ·mol^−1^.

### 2.4 Catalytic Activity Assessment

#### 2.4.1 Homocoupling of Bis(4‐methylphenyl)diazomethane

**SCHEME 3:**
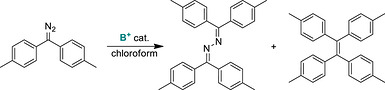
Homocoupling of bis(4‐methylphenyl)diazomethane. Model reaction catalyzed by the title compounds yields a mixture of azine and ethene products in a variable ratio (see Table 2).

Diazo‐compounds are very useful intermediates due to their rather high reactivity. Some of them undergo very slow homocoupling to the corresponding ethenes even at room temperature under light exposure. During our previous investigations, we noticed that this reaction can also be catalyzed with organoboron Lewis acids, including boreniums [37]. Therefore, it can be used as a model reaction to compare the catalytic potency of the currently investigated compounds. Similar to previously, the process is not selective toward the ethene product in all cases, and it can also yield a bis‐substituted hydrazine, as revealed by the NMR studies (Scheme 3).

A few of the title compounds do not catalyze the aforementioned reaction to a reasonable extent. Compounds ***1a***, ***1d***, ***1*** ***g***, ***1g’***, ***1*** ***h***, ***3a***, and ***3c*** require 10–20 mol. % or higher loadings to produce a visible amount of the product over 24 h. Therefore, those compounds were excluded from the kinetic experiments. We were, however, able to ascertain the NMR product distribution in all cases, regardless of the conversion. Dihydridoboreniums ***1a*** and ***3a*** (similarly to ***2a***) are very reactive and can engage in some side reactions, nullifying their catalytic properties in this process. Poor performance of ***1*** ***h*** can be explained by a very low *AN* = 28.2 and the greatest steric crowding around the boron center. Other excluded compounds do not share any common characteristics. The majority of the remaining compounds are excellent catalysts, and the reaction progress was monitored by UV‐Vis, which is a more suitable and faster technique than NMR.

For the kinetic UV‐Vis measurements, bis(4‐methylphenyl)diazomethane starting material was selected, with *λ*_max._ = 535 nm, for the products do not absorb at this wavelength region. Table 2 presents the results of the kinetic measurements (tracking the consumption of the starting material). For two comparably weaker catalysts in the series, ***1c*** and ***1f***, the catalyst loading was doubled (to 1 mol. %) for practical reasons. However, higher loadings would render the results incomparable. Table S1 in the Supporting Information presents additional details of the data fitting.

**TABLE 2:** Diazo‐homocoupling kinetic measurements.^a^

	Cat. / mol.%	*k*_obs._ ^a^ / 10^–^^5^ s^–^^1^	prod. distrib. / %^b^	
			ethen	azine
***1a***	—	‐^**^	100	0
***1b***	0.5	**35.4**	100	0
***1b’***	0.5	**42.4**	70	30
***1c***	1.0	3.54	100	0
***1d***	—	‐^**^	0	100
***1e***	0.5	**11.2**	100	0
***1f***	1.0	4.48	100	0
***1g***	—	‐^**^	90	10
***1g’***	—	‐^**^	100	0
***1h***	—	‐^**^	100	0
***2b***	0.5	**25.9**	100	0
***3a***	—	‐^**^	100	0
***3b***	0.5	**39.4**	0	100
***3c***	—	‐^**^	100	0
***3d***	0.5	6.24	99	*trace*
***3e***	0.5	**21.6**	0	100
***3g***	0.5	**35.2**	5	95
***4a*** ^c^	0.5	0.821	100	0

* Conditions: [diazo] = 10 mm, [cat.] = 0.05∼0.1 mm, *T* = 20 °C, *t* = 0–90 min.

** Kinetics too slow to measure at comparable conditions.

^a^ First‐order fitting,

^b^ NMR assessment after 24 h,

^c^ ***4** **a*** = tris(pentafluorophenyl)‐borane.

1,2‐Bis(di‐4‐tolylmethylene)hydrazine product is exclusively observed for catalysts ***1d***, ***3b*,** and ***3e***, and a mixture of products for ***1b’***, ***1g***, ***3d*** (trace azine), and ***3g***. One can see from Table 2 that none of the catalysts lacking aromatic rings in the structure of the ‐R^2^ substituents yield the azine product (***1a***, ***1c***, ***1h***, ***3a*,** and ***3c***). This suggests that some kind of π‐π or p‐π interaction is responsible for the selectivity control. All of the boreniums included in the kinetic experiments are better catalysts than B(C_6_F_5_)_3_ (***4a***), with ***1c***, ***1e***, ***1f***, and ***3d*** having comparable *k*_obs_. For the discussed model reaction, catalysts ***1b***, ***1b’***, ***1e***, ***2b***, ***3b***, ***3e*,** and ***3*** ***g*** display the fastest kinetics, having the observed kinetic constant an order of magnitude higher than the remaining compounds. The pattern here is clear, indicating the importance of flat, 5‐ and 7‐member dioxaboracycles. The contrasting results for ***1*** ***g*** (and ***1g’***) against ***3*** ***g*** can be explained by the significant repulsion from the mesityl‐ substituents on the labile 7‐membered cycle, which is causing the cycle to adopt a less flat conformation and more of a skewed chair‐like conformation. The BINOL moiety is less prone to this effect, as the *k*_obs._ for ***1e*** and ***3e*** are much more comparable. Overall, the catechol‐derived boreniums are all very good catalysts for this model reaction, with ***3b*** yielding azine product exclusively. In summary, the catalyst choice enables control over the reaction outcome and product distribution.

#### 2.4.2 Hydrosilylation of Acetophenone

**SCHEME 4:**
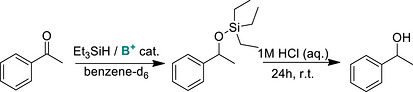
Hydrosilylation of acetophenone catalyzed by the title compounds in a model reaction, optionally followed by hydrolysis to benzyl alcohol.

Reductions of aldehydes, ketones, imines, and enamines are standard model reactions used to assess the catalytic properties of Lewis acids (like BCF) [38, 39]. The readily available acetophenone was selected to react with Et_3_SiH to form a silyl ether as the product, which can be easily hydrolyzed to 1‐phenylethanol and isolated via chromatography (Scheme 4, Table 3). The majority of the title compounds do not catalyze this process, even at 10 mol. % loading (and only traces of the product are found in some cases after 24 h at 55°C). In many of these cases, there is trace evidence of deoxygenation of acetophenone to ethylbenzene to a negligible degree. The hydrosilylation product can be obtained in high yield only for catalysts ***1d***, ***3c***, ***3d***, and clearly observed in the case of ***1c*** (up to 29%), and a small amount (6%) for ***1*** ***g*** (after 24 h, at elevated temp.). Compounds ***1c***, ***1d***, ***3c***, ***3d*** represent pairs of parent structures derived from phenylacetylene and *t*‐butyl acetylene in 2:1 hydroboration with IMes→BH_2_^+ ^NTf_2_^–^ and IMe_2_→BH_2_^+ ^NTf_2_^–^. Those are the only non‐cyclic and non‐oxygen‐containing structures in our series. The mechanism of this reaction was previously confirmed to proceed in the first step via hydride abstraction from Et_3_SiH to the Lewis acid catalyst, and hydride donation from it in the last step [40, 41].

**TABLE 3:** NMR yield for the silyl ether product of the hydrosilylation of acetophenone at r.t^a^.

Cat.	cat. 1 mol. %	cat. 10 mol. %
***1a***	0	0 (*0*)^a^
***1b***	0	0 (*trace*)^a^
***1b'***	0	0 (*trace*)^a^
***1c***	17	34 (*29*)^b^
***1d***	89 (*85*)^b^	98 (*92*)^b^ (*100*)^a^
***1e***	0	trace (*trace*)^a^
***1f***	0	0 (*trace*)^a^
***1g***	0	0 (*6*)^a^
***1g'***	0	0 (*0*)^a^
***1h***	0	0 (*0*)^a^
***2a***	0	0 (*0*)^a^
***2b***	0	0 (*0* ^a^ ^a^
***3a***	trace	trace (*trace*)^a^
***3b***	0	0 (*trace*)^a^
***3c***	90 (*84*)^b^	99 (*94*)^b^
***3d***	99 (*80*)^b^	100 (*85*)^b^
***3e***	0	trace (*trace*)^a^
***3g***	0	0 (*0*)^a^
***4a*** ^b^	80 (*100*)^a^	84 (*88*)^b^
***3** **d*** ^c^	—	100 (*88*)^b^
***3** **d*** ^d^	—	100 (*78*)^b^
***3d*** ^e^	—	100 (*69*)^b^
***3d*** ^f^	—	100 (*73*)^b^
***3d*** ^g^	—	0

* General reaction conditions: acetophenone (1 eq.), Et_3_SiH (2 eq.), C_6_D_6_ for NMR reactions (0.5 mL) or toluene (for 1.5 mmol scale), 1 h.

** ***4a*** = tris(pentafluorophenyl)borane.

^a^ NMR yield of the reaction conducted over 24 h at 55 °C with 10 mol. % cat. loading.

^b^ Isolated yield of the benzyl alcohol (scale 1.5 mmol).

^c^ Reaction with 2,2‐dimethyl‐1‐phenylpropan‐1‐one.

^d^ Reaction with cyclohexyl(phenyl)methanone.

^e^ Reaction with 4‐chloroacetophenone.

^f^ Reaction with 4‐bromoacetophenone.

^g^ Reaction with di‐*4,4´*‐tolylmethanone.

In order to gain insight into the reaction pathway, representative catalysts ***1b***, ***1*** ***g***, ***1d***, ***3d***, and ***3e*** were combined with excess amounts of acetophenone and Et_3_SiH separately and analyzed by NMR (See Figures S6–S11 in the Supporting Information). In the case of ***3d***, in reaction with Et_3_SiH, there is clear evidence of adduct formation, as indicated by ^11^B NMR signals observed in the region corresponding to tetrahedral boron. For ***3e***, there is evidence of catalyst decomposition, most probably resulting from the hydrosilylation of the catalyst B‐O bonds. Interestingly, cation ***3e*** is also engaging in a C═O→B adduct formation in the presence of acetophenone, which is not observed for ***3d***. Catalysts ***1b*** and ***1g*** are not showing any interaction with either reagent in NMR, demonstrating the extent of the steric hindrance from the two mesityl‐ moieties, having an impact on interaction even with Et_3_SiH. This outcome is somewhat unexpected, considering the observed strong interaction with TEPO for the majority of IMes‐stabilized boreniums. Therefore, catalyst ***1d*** was also investigated under excess of both reagents, manifesting only slow conversion to hydride adduct after 30 min. (Figure S8, bottom), which confirms the steric influence and is in agreement with the observed hydrosilylation efficiency (slightly lower than ***3c*** and ***3d*** after 1 h). The somewhat lower efficiency for ***1c*** can be also attributed to the combined steric hindrance from mesityl‐ and *t*‐Bu moieties, which is reflected in a lower *AN* assessed for this compound (*AN* = 89.4) and lower HIA, with respect to ***1d***, ***3c***, and ***3d***. For those four cases, the hydrosilylation was performed on a larger scale (1.5 mmol), and the product was hydrolyzed to alcohol and isolated by flash chromatography. In the case of ***1*** ***h*** (catalyst derived from 9‐BBN), no product was obtained even after 24 h at 55°C. This result stands in contrast to the very good performance displayed by the lutidine‐stabilized [9‐BBN‐LB]^+^[NTf_2_]^–^ borenium, investigated by Denmark et al. in the same process [40]. The very low *AN* measured for ***1*** ***h*** (*AN* = 28.2) further confirms the combined steric effect. The NHC→BH_2_^+^ species ***1a***, ***2a***, and ***3a*** are not active as catalysts for this reaction. Most probably because they are undergoing reduction in the presence of Et_3_SiH. In the case of ***3a***, traces of the product were observed, most probably resulting from the marginal pathway catalyzed by the in situ‐formed product of acetophenone hydroboration.

We have submitted other starting materials to this protocol with ***3d*** as the best catalyst. Phenyl‐*tert*‐butyl ketone and phenyl‐cyclohexyl ketone were selected to represent higher steric hindrance. 4‐Chloroacetophenone and 4‐bromoacetophenone—to probe EWG presence and di‐*4,4´*‐tolylmethanone as a benzophenone representative. In all the mentioned cases (except benzophenone), the reaction was quite rapid and quantitative (see Table 3, bottom entries). The reaction with di‐*4,4´*‐tolylmethanone gave traces of an unidentified product, suggesting ***3d*** enters a stoichiometric reaction with the starting material.

## 3 Conclusion

We have synthesized and characterized a series of 13 diverse borenium cationic compounds in pure form and assessed their catalytic behavior in two benchmark model reactions. The great majority of the title compounds are characterized by a very high *AN* (Acceptor Number) around 90–98 units, indicating a Lewis acidity significantly stronger than that of the neutral benchmark Lewis acid B(C_6_F_5_)_3_. Both calculated FIA and HIA indices confirm this trend. However, the catalytic performance is diverse and depends strongly on the structural properties and thus the variable factors affecting the global Lewis acidity of the boron center and the overall stability of the borenium cations. All of the investigated catalysts are active in diazo‐homocoupling reaction, though with different efficiency and leading to a different product distribution. Notably, the choice of the catalyst allows for the control over the reaction outcome, leading, instead of tetraarylethylene, to the formation of the corresponding bis(diarylmethylene)hydrazine. Simplifying, catalysts with cationic boron included in a dioxacycle are performing much better in this reaction. In contrast, the situation is somewhat reversed in the case of hydrosilylation. Only the non‐cyclic, non‐oxygen‐containing variants are active in this process. This outcome shows that the catalytic reactivity of boreniums can be controlled by the structural factors to a great extent. This information can be particularly useful while preparing the catalyst directly in the reaction media, considering that all the title compounds were synthesized from the same in situ‐generated dihydridoborenium species.

## Conflicts of Interest

The authors declare no conflicts of interest.

## Supporting information



The authors have cited additional references within the Supporting Information [42, 43, 44, 45, 46, 47, 48, 49]. CCDC Deposition Numbers [50].
