# Supplementary material for: Lewis‐Acidic Properties and Catalytic Behavior of Hydroboration‐ and Diolysis‐Derived NHC‐Stabilized Borenium Cations
Source: Chemistry. 2026 May 6;32(31):e71035. doi: 10.1002/chem.71035 (PMC13485243; doi:10.1002/chem.71035)
Supplement: Supplementary file 1 [file CHEM-32-e71035-s001.pdf]

Supporting Information

Lewis-acidic properties and catalytic behavior of hydroboration- and diolysis-derived NHC-stabilized borenium cations.

Michał Jakubczyk,* Michaela Buziková, Matouš Kloda, Miroslava Litecká and Karel Škoch.

Department of Materials Chemistry
Institute of Inorganic Chemistry, Czech Academy of Sciences
250 68 Husinec-Řež č.p. 1001, Czech Republic
E-mail: *jakubczyk@iic.cas.cz*

TABLE OF CONTENTS

MATERIALS AND METHODS 2
PREPARATIVE PROCEDURES........ 3
CHARACTERIZATION OF THE OBTAINED TITLE BORENIUM COMPOUNDS 4
CATALYTIC EXPERIMENTS 10
HOMOCOUPLING OF BIS(4-METHYLPHENYL)DIAZOMETHANE 10
HYDROSILYLATION OF ACETOPHENONE 13
KINETIC MEASUREMENTS........ 23
ASSESSMENT OF THE LEWIS ACIDITY BY GUTMANN METHOD 24
CRYSTAL STRUCTURE DATA........ 26
COMPUTATIONAL DETAILS 32
NMR SPECTRA REPRINTS........ 34
REFERENCES 112

MATERIALS AND METHODS

All reagents were obtained from commercial sources (Sigma-Aldrich, Fluorochem, TCI, ABCR, BLDPharm) and used as received unless otherwise indicated. Tris(pentafluorophenyl)borane was obtained commercially, recrystallized from hexane, and sublimated under Ar before use. Synthesis of the title compounds and catalytic experiments were completed in an atmosphere-controlled glovebox filled with dry Ar gas, unless otherwise indicated. Solvents for glovebox use were pre-dried with activated 4Å molecular sieves, refluxed over Na or Na/K alloy until dry (benzophenone indicator), and distilled under Ar, and stored in the glovebox over molecular sieves.

1,3-(Bis(2,4,6-trimethylphenyl)imidazol-2-ylidene (IMes),[1] (1,3-(bis(2,4,6-trimethylphenyl)-imidazol-2-ylidene)trihydridoboron (IMes→BH_3),[2] (1,3-bis(2,4,6-trimethylphenyl)imidazolidin-2-ylidene)-trihydrido-boron (SIMes→BH_3),[3] (1,3-dimethylimidazol-2-ylidene)trihydridoboron (IMe_2→BH_3),[4] and bis-(4-methylphenyl)diazomethane[5] were prepared according to previously reported procedures.

Deuterated solvents were dried over activated 4Å molecular sieves and stored in a glovebox over freshly activated molecular sieves.

Characterization

All NMR measurements were performed in J. Young NMR tubes and the samples were prepared inside the glovebox. NMR measurements were completed using a JEOL Delta 600 MHz instrument at 20 °C. ^{1}H and ^{13}C NMR chemical shifts (δ in ppm) are given relative to TMS and referenced to the residual solvent signal ($CDCl_3$: δ_H = 7.26 ppm, δ_C = 77.0 ppm, CD_2Cl_2: δ_H = 5.32 ppm, δ_C = 53.8 ppm, C_6D_6: δ_H = 7.16 ppm, δ_C = 128.06 ppm). ^{11}B, ^{19}F and ^{31}P are referenced according to the primary reference for the unified chemical shift scale following the IUPAC recommendation (R.K. Harris, E.D. Becker, S.M. Cabral de Menezes, R. Goodfellow, P. Granger, *Pure Appl. Chem.* 2001, 73, 1795-1818). The multiplicity of the signals is indicated as s, d, t, q, or m for singlets, doublets, triplets, quartets or multiplets. Coupling constants *J* are given in Hertz as positive values regardless of their sign. Assignment of the peaks was supported by 2D experiments (COSY, HSQC, and HMBC). The MestReNova software package was used for analyzing the spectra.

Elemental Analysis was completed with FlashSmartTM Elemental Analyzer. Tin capsules were loaded with samples and sealed in a glovebox.

Mass spectrometry measurements were performed on an Orbitrap ExplorisTM 120 instrument using electrospray ionization.

UV-Vis absorption spectra were recorded on a Perkin Elmer Lambda 35 spectrometer equipped with a Peltier modules and thermostat cuvette holders. Plots were visualized with the LabPlot Data Visualization and Analysis software (LabPlot Team (2025), Version 2.12.1, https://labplot.org).

Single-Crystal X-ray Analyses

X-ray diffraction analysis was done with a Rigaku XtaLAB Synergy S diffractometer equipped with Mo (Mo/Kα radiation; λ = 0.71073 Å) and Cu (Cu/Kα radiation; λ = 1.54184 Å) micro-focus X-ray source and Hybrid Pixel Array Detector (HyPix-6000HE). Oxford Cryosystems (Cryostream 800) cooling device was used to collect data at 100 K. CrysAlis Pro software was used for data collection and cell refinement, data reduction and absorption correction [CrysAlis PRO 1.171.42.94a (Rigaku OD, 2023)]. Data was corrected for absorption effects using empirical absorption correction (spherical harmonics), implemented in SCALE3 ABSPACK scaling algorithm and numerical absorption correction based on gaussian integration over a multifaceted crystal model. All the structures were solved with the ShelXT (Sheldrick, G.M. (2015). *Acta Cryst*. A71, 3-8.) structure solution program using Intrinsic Phasing and refined with the ShelXL (Sheldrick, G.M. (2015). *Acta Cryst*. C71, 3-8, 10.1107/S2053229614024218) refinement package using least squares minimization procedure implemented in Olex2 (Dolomanov, O.V., Bourhis, L.J., Gildea, R.J, Howard, J.A.K. & Puschmann, H. (2009), *J. Appl. Cryst*. 42, 339-341, 10.1107/S0021889808042726). Anisotropic displacement parameters were refined for all non-H atoms. The hydrogen atoms were calculated to the idealized positions or found in the residual electron density map (boron hydrides). The structure of ***3e*** was refined as an inversion twin. For crystallographic data and structure refinement, see SI tables below. Molecular graphic for all compounds was generated using PLATON software (Spek, A.L. (2009) *Acta Cryst*. D65, 148-155, doi:10.1107/S090744490804362X).

Theoretical calculations

All DFT computations were performed in the Gaussian 16 program package (Gaussian 16, Revision C.01, M. J. Frisch, G. W. Trucks, H. B. Schlegel, G. E. Scuseria, M. A. Robb, J. R. Cheeseman, G. Scalmani, V. Barone, G. A. Petersson, H. Nakatsuji, X. Li, M. Caricato, A. V. Marenich, J. Bloino, B. G. Janesko, R. Gomperts, B. Mennucci, H. P. Hratchian, J. V. Ortiz, A. F. Izmaylov, J. L. Sonnenberg, D. Williams-Young, F. Ding, F. Lipparini, F. Egidi, J. Goings, B. Peng, A. Petrone, T. Henderson, D. Ranasinghe, V. G. Zakrzewski, J. Gao, N. Rega, G. Zheng, W. Liang, M. Hada, M. Ehara, K. Toyota, R. Fukuda, J. Hasegawa, M. Ishida, T. Nakajima, Y. Honda, O. Kitao, H. Nakai, T. Vreven, K. Throssell, J. A. Montgomery, Jr., J. E. Peralta, F. Ogliaro, M. J. Bearpark, J. J. Heyd, E. N. Brothers, K. N. Kudin, V. N. Staroverov, T. A. Keith, R. Kobayashi, J. Normand, K. Raghavachari, A. P. Rendell, J. C. Burant, S. S. Iyengar, J. Tomasi, M. Cossi, J. M. Millam, M. Klene, C. Adamo, R. Cammi, J. W. Ochterski, R. L. Martin, K. Morokuma, O. Farkas, J. B. Foresman, and D. J. Fox, Gaussian, Inc., Wallingford CT, 2019.).

If available, the geometry optimizations were started from atomic coordinates determined by X-ray diffraction analysis. All geometries were optimized using the PBE0 density functional in conjunction with the def2-TZVP basis set with added Grimme's D3 dispersion correction. Solvent effects (DCM) have been approximated using the PCM model (IEFPCM). Frequencies were calculated to ensure that the optimized structure is not a transition state. The FIA and HIA were calculated according to the method developed by Greb *et al.* using the geometries and the thermal corrections obtained at the PBE0(D3)/def2-TZVP level of theory, and calculating final single-point electronic energies at the PW6B95(D3)/def2-QZVPP level.[6,7] The Me_3Si^+/Me_3SiF and Me_3Si^+/Me_3SiH quasi-isodesmical anchor reaction systems were applied.

PREPARATIVE PROCEDURES

General method 1: In a glovebox, 0.251 mmol (1 equiv.) of NHC$\rightarrow$$BH_3$ complex was placed in a Schlenk vessel and dissolved in approx. 4 mL of dry DCM. To this solution, a solution of 0.251 mmol (1 equiv.) of $HN(SO_2CF_3)_2$ or $Ph_3CB(C_6F_5)_4$ in 1-2 mL DCM was added slowly. In case of $HN(SO_2CF_3)_2$ gas evolution was visible instantly. The reaction mixture was stirred for 30 min at room temperature to ensure complete conversion. (Compounds ***1a*** and ***3a*** can be isolated by solvent removal, washing with hexane and drying *in vacuo*). In the second step, 0.251 mmol (1 equiv.) of a diol (or diene) or 0.502 mmol (2 equiv.) of alkyne was dissolved in 1 mL of DCM and added. In case of diols gas evolution was visible. The reaction mixture was stirred for 12 h at room temperature. All the volatiles were evaporated *in vacuo*. 1-2 mL of fresh DCM were added to dissolve the residue, and 5-6 mL of dry hexane was slowly added until the solution became cloudy. Vigorous mixing over 30 min resulted in two layers. Hexane was pipetted away from above the clear oil. The remaining oil was washed twice with 2-3 mL of fresh hexane. The residue was dried under vacuum for 1-3 h until it solidified. The foamy sticky solid was scratched from the walls of the vessel and dried further under vacuum until a fine powder was obtained.

General method 2: In a glovebox, 0.334 mmol (1 equiv.) of IMes: was dissolved in 3 mL of dry toluene in a Schlenk vessel, 0.334 mmol (1 equiv.) of mono-hydroborane was dissolved in 3 mL of dry toluene in a vial, and was slowly added to the solution. The reaction mixture was initially clear. After a few minutes of stirring, a precipitate formed. The toluene suspension was stirred for 2 h at room temperature to ensure complete conversion. Toluene was evaporated. (Compounds ***1b*H** and ***1h*H** can be isolated as powders at this point by washing with hexane and drying *in vacuo*, and can be stored in the glovebox over a few months without decomposition). In the second step, the obtained residue was dissolved in 4 mL of dry DCM. 0.334 mmol (1 equiv.) of $HN(SO_2CF_3)_2$ or $Ph_3CB(C_6F_5)_4$ were dissolved in 4 mL of dry DCM in a vial. The acid solution was added slowly to the Schenk vessel (in case of $HN(SO_2CF_3)_2$ gas evolution was observed). The reaction mixture was stirred for 1 h at room temperature. Then, the volatiles were evaporated, and the residue was dissolved in a few drops of DCM and washed with dry hexane. The solvent was pipetted away from above the residue. This procedure was repeated until the product solidified or a thick oil was obtained. Then, the residue was dried under vacuum until a solid foam formed that could be crushed to obtain a powder.

CHARACTERIZATION OF THE OBTAINED TITLE BORENIUM COMPOUNDS

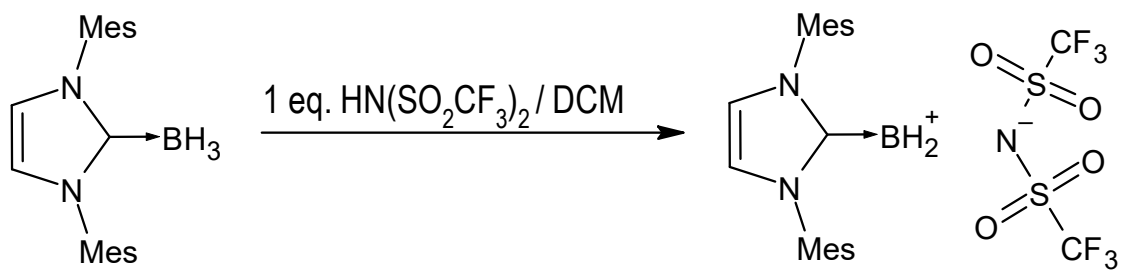

$[IMesBH_2]^{\oplus}[N(SO_2CF_3)_2]^{\ominus}$ (***1a***): Obtained in the first step of *method 1*. Yield = 95%, white powder.
^1H NMR (600 MHz) *δ*/ppm (benzene-d_6): 6.76 (s, 4H, CH-Mes), 5.85 (s, 2H, imidazolylidene-CH), 2.90 (br s, 2H, BH_2), 2.08 (s, 6H, *para*-CH_3), 2.04 (s, 12H, *ortho*-CH_3).
^{19}F NMR (565 MHz) *δ*/ppm (benzene-d_6): -71.2.
^{11}B NMR (193 MHz) *δ*/ppm (benzene-d_6): -17.5 (FWHM: 440 Hz).
$^{11}B\{^1H\}$ NMR (193 MHz) *δ*/ppm (benzene-d_6): -17.5 (FWHM: 350 Hz).
$^{13}C\{^1H\}$ NMR (151 MHz) *δ*/ppm (benzene-d_6): 163.7 (*C*-carbene), 140.0 (*para*-CH_3-*C*-Mes), 135.1 (*ortho*-CH_3-*C*-Mes), 133.4 (N-*C*-Mes), 129.8 (*C*H-Mes), 122.4 (imidazolylidene-*C*H), 120.4 (q, $^1J_{CF}$ = 326.5 Hz, *C*F_3), 21.0 (*para*-*C*H_3), 17.9 (*ortho*-*C*H_3).
EA: Calc. for $C_{23}H_{26}BF_6N_3O_4S_2 \cdot 0.25$ DCM C: 45.14%, H: 4.32%, N: 6.79%, Found C: 45.10%, H: 4.38% N: 7.16%.

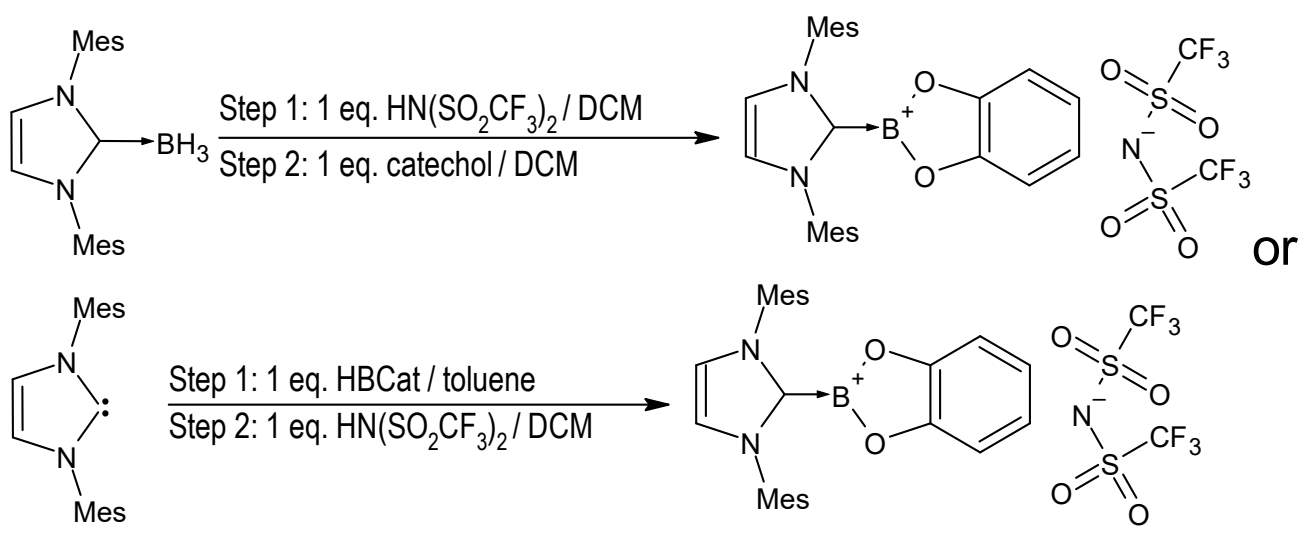

$[IMesBCat]^{\oplus}[N(SO_2CF_3)_2]^{\ominus}$ (***1b***): Obtained by *method 1,* yield = 45% and *method 2,* yield = 68%, off white powder.
^1H NMR (600 MHz) *δ*/ppm (DCM-d_2): 7.88 (s, 2H, imidazolylidene-CH), 7.20 - 7.09 (m, 4H, CH-catechol), 7.17 (s, 4H, CH-Mes), 2.43 (s, 6H, *para*-CH_3), 2.20 (s, 12H, *ortho*-CH_3).
^{19}F NMR (565 MHz) *δ*/ppm: -79.4.
$^{11}B\{^1H\}$ NMR (193 MHz) *δ*/ppm: 24.9 (FWHM: 270 Hz).
$^{13}C\{^1H\}$ NMR (151 MHz) *δ*/ppm: 160.4 (*C*-carbene), 147.3 (*C*-O), 142.8 (*para*-CH_3-*C*-Mes), 134.5 (*ortho*-CH_3-*C*-Mes), 131.7 (N-*C*-Mes), 130.5 (*C*H-Mes), 129.5 (imidazolylidene-*C*H), 125.3 (*C*H-catechol), 120.4 (q, $^1J_{CF}$ = 321.5 Hz, *C*F_3), 114.1 (*C*H-catechol), 21.6 (*para*-CH_3), 17.6 (*ortho*-CH_3).
HRMS [m/z]: (pos.) Found: 423.2280 Da (Calc. for molecular borenium $C_{27}H_{28}BN_2O_2^{\oplus}$: 423.2238 Da), Found: 305.2044 Da (Calc. for $IMesH^{\oplus}$ fragment $C_{21}H_{24}N_2H^{\oplus}$: 305.2012 Da), (neg.) Found 279.9152 Da (Calc. for triflimide anion $C_2S_2O_4F_6N^{\ominus}$: 279.9178 Da).

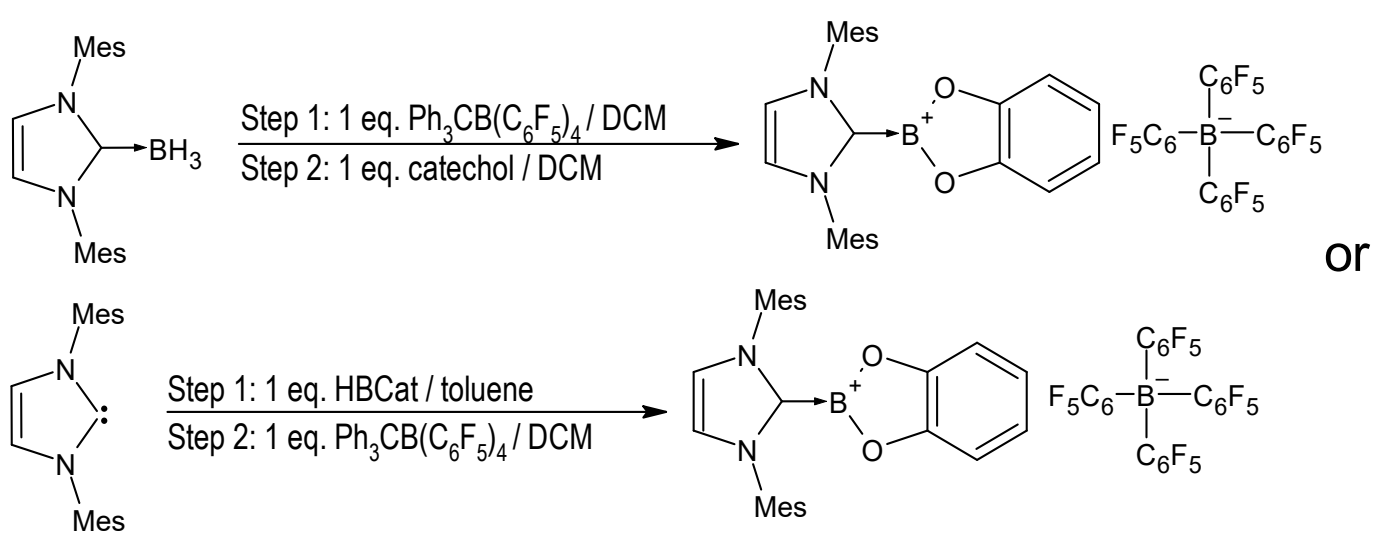

$[IMesBCat]^{\oplus}[B((C_6F_5)_4]^{\ominus}$ (***1b'***): Obtained by *method 1*, yield = 73% and *method 2* yield = 80%, pale violet powder.
^1H NMR (600 MHz) *δ*/ppm (DCM-d_2): 7.74 (s, 2H, imidazolylidene-CH), 7.17 (s, 4H, CH-Mes), 7.18 - 7.10 (m, 4H, CH-catechol), 2.43 (s, 6H, *para*-CH_3), 2.11 (s, 12H, *ortho*-CH_3).
^{19}F NMR (565 MHz) *δ*/ppm (DCM-d_2): -133.1 (s, 8F), -163.6 (t, 4F, $^3J_{FF}$ = 20.4 Hz), -167.47 (t, 8F, $^3J_{FF}$ = 17.9 Hz).
$^{11}B\{^1H\}$ NMR (193 MHz) *δ*/ppm (DCM-d_2): 24.7 (FWHM: 190 Hz) (borenium), -17.7 (FWHM: 20 Hz) (borate).

$^{13}C\{^1H\}$ NMR (151 MHz) δ/ppm: (DCM-d_2): *C*-B⊖ (not observed), 160.1 (*C*-carbene), 148.7 (dm, $^1J_{CF}$ = 239.5 Hz, *C*F), 147.2 (*C*-O), 143.1 (*para*-CH$_3$-*C*-Mes), 138.8 (dm, $^1J_{CF}$ = 253.9 Hz, *C*F), 136.8 (dm, $^1J_{CF}$ = 248.4 Hz, *C*F), 134.4 (*ortho*-CH$_3$-*C*-Mes), 131.5 (N-*C*-Mes), 130.7 (*C*H-Mes), 129.5 (imidazolylidene-*C*H), 125.4 (*C*H-catechol), 114.1 (*C*H-catechol), 21.5 (*para*-*C*H$_3$), 17.5 (*ortho*-*C*H$_3$).
EA: Calc. for $C_{51}H_{28}B_2F_{20}N_2O_2$ C: 55.57%, H: 2.56%, N: 2.54%, Found C: 55.29%, H: 3.01% N: 2.83%.

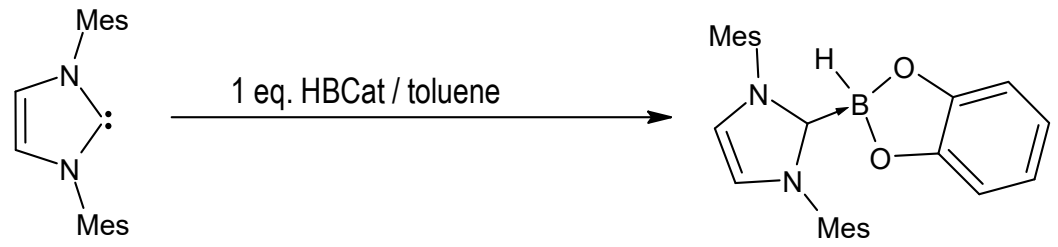

IMesB(H)Cat (***1b*H**): Obtained in the first step of *method 2*, yield = 99% white powder.
^1H NMR (600 MHz) δ/ppm (DCM-d_2): 7.05 (s, 2H, imidazolylidene-C*H*), 6.89 (s, 4H, CH-Mes), 6.24 - 6.22 (m, 2H, CH-catechol), 6.04 - 6.01 (m, 2H, CH-catechol), 3.61 (m, 1H, B-H), 2.30 (s, 6H, *para*-CH$_3$), 2.03 (s, 12H, *ortho*-CH$_3$).
^{11}B NMR (193 MHz) δ/ppm (DCM-d_2): 3.97 (d, $^1J_{BH}$ = 121.1 Hz).
$^{11}B\{^1H\}$ NMR (193 MHz) δ/ppm (DCM-d_2): 3.98 (s) (FWHM: 50 Hz).
$^{13}C\{^1H\}$ NMR (151 MHz) δ/ppm: (DCM-d_2): *C*-carbene (not observed), 153.4 (*C*-O), 140.0 (*para*-CH$_3$-*C*-Mes), 135.2 (N-*C*-Mes), 134.0 (*ortho*-CH$_3$-*C*-Mes), 129.3 (*C*H-Mes), 122.9 (imidazolylidene-*C*H), 117.6 (CH-catechol), 108.4 (CH-catechol), 21.4 (*para*-*C*H$_3$), 17.6 (*ortho*-*C*H$_3$).
HRMS [m/z]: (pos.) Found: 423.2260 Da (Calc. for borenium $C_{27}H_{28}BN_2O_2^{\oplus}$: 423.2238 Da), Found: 305.2028 Da (Calc. for IMesH⊕ fragment $C_{21}H_{24}N_2H^{\oplus}$: 305.2012 Da).

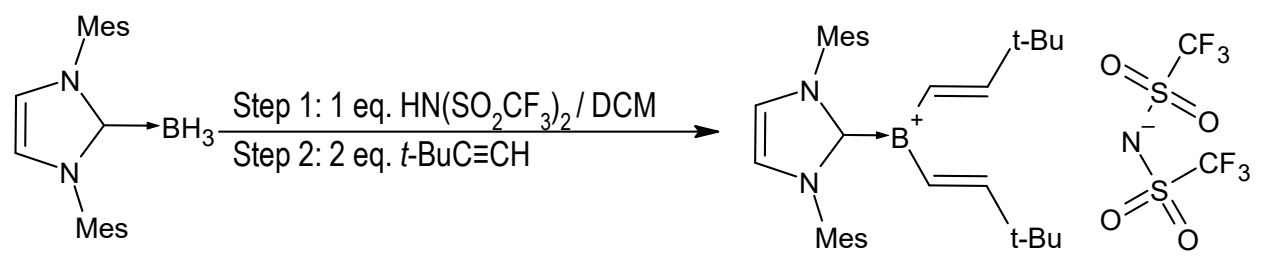

[IMesB(CH=CH-*t*Bu)$_2$] ⊕[N(SO$_2$CF$_3$)$_2$]⊖ (***1c***): Obtained by *method 1*. Yield = 40%, light yellow powder.
^1H NMR (600 MHz) δ/ppm (DCM-d_2): 7.69 (s, 2H, imidazolylidene-CH), 7.07 (s, 4H, CH-Mes), 6.47 (d, 2H, $^3J_{HH}$ = 17.2 Hz, B-C*H*=CH), 5.52 (d, 2H, $^3J_{HH}$ = 17.2 Hz, B-CH=C*H*), 2.36 (s, 6H, *para*-CH$_3$), 2.07 (s, 12H, *ortho*-CH$_3$), 0.77 (s, 18H, *t*-Bu).
^{19}F NMR (565 MHz) δ/ppm (DCM-d_2): -79.3.
$^{11}B\{^1H\}$ NMR (193 MHz) δ/ppm (DCM-d_2): 54.2 (FWHM: 670 Hz).
$^{13}C\{^1H\}$NMR (151 MHz) δ/ppm: (DCM-d_2): 177.4 (*C*H=CH–*t*-Bu), 152.5 (*C*-carbene), 142.4 (*para*-CH$_3$-*C*-Mes), 134.4 (N-*C*-Mes), 132.8 (*ortho*-CH$_3$-*C*-Mes), 130.6 (*C*H-Mes), 127.6, (CH=*C*H–*t*-Bu), 125.8 (imidazolylidene-*C*H), 120.5 (d, $^3J_{CF}$ = 321.8 Hz, *C*F$_3$), 36.7 (*t*-Bu-*C*), 28.3 (*t*-Bu-*C*H$_3$), 21.3 (*para*-*C*H$_3$), 18.0 (*ortho*-*C*H$_3$).
EA: Calc. for $C_{35}H_{46}BF_6N_3O_4S_2$ C: 55.19%, H: 6.09%, N: 5.52%, Found C: 55.0%, H: 6.52% N: 5.87%.

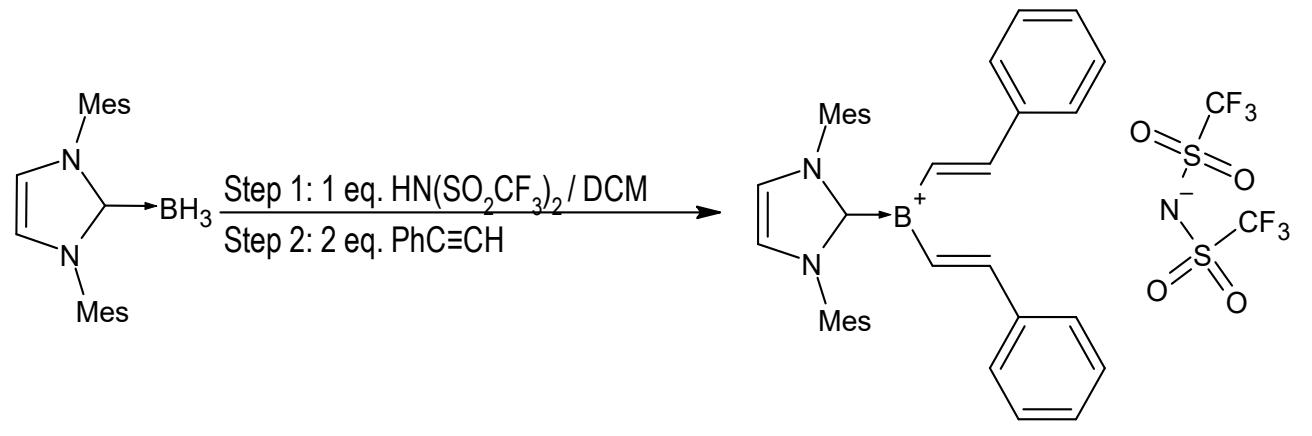

[IMesB(CH=CH–Ph)$_2$]⊕ [N(SO$_2$CF$_3$)$_2$]⊖ (***1d***): Obtained by *method 1*. Yield = 37%, yellow powder.
^1H NMR (600 MHz) δ/ppm (DCM-d_2): 7.79 (s, 2H, imidazolylidene-CH), 7.41 - 7.35 (m, 6H, Ar), 7.33, (d, 2H, $^3J_{HH}$ = 17.8 Hz, B-C*H*=CH), 7.21 (d, 4H, $^3J_{HH}$ = 7.5 Hz, Ar), 7.07 (s, 4H, CH-Mes), 6.38 (d, 2H, $^3J_{HH}$ = 17.6 Hz, B-CH=C*H*), 2.23 (s, 6H, *para*-CH$_3$), 2.15 (s, 12H, *ortho*-CH$_3$).
^{19}F NMR (565 MHz) δ/ppm (DCM-d_2): -75.5.
$^{11}B\{^1H\}$ NMR (193 MHz) δ/ppm (DCM-d_2): 53.7 (FWHM: 880 Hz).
$^{13}C\{^1H\}$ NMR (151 MHz) δ/ppm: (DCM-d_2): 159.2 (*C*H=CH–Ph), 151.8 (*C*-carbene), 142.7 (*para*-CH$_3$-*C*-Mes), 136.7 (Ph-*C*-ipso), 134.5 (N-*C*-Mes), 132.8 (*C*H-Ph), 131.9 (*ortho*-CH$_3$-*C*-Mes), 130.7 (CH=*C*H–Ph), 130.5 (*C*H-Mes), 129.4 (*C*H-Ph), 128.9 (*C*H-Ph), 128.0 (imidazolylidene-*C*H), 120.5 (q, $^1J_{CF}$ = 321.6 Hz, *C*F$_3$), 21.3 (*para*-*C*H$_3$), 18.0 (*ortho*-*C*H$_3$).
HRMS [m/z]: (pos.) Found: 521.3130 Da (Calc. for molecular borenium $C_{37}H_{38}BN_2^{\oplus}$: 521.3123 Da), Found: 349.2086 Da (Calc. for hydrolysis product $C_{21}H_{26}BN_2O_2^{\oplus}$: 349.2082 Da. Found: 305.2016 Da (Calc. for IMesH⊕ fragment $C_{21}H_{24}N_2H^{\oplus}$: 305.2012 Da), (neg.) Found 279.9176 Da (Calc. for triflimide anion $C_2S_2O_4F_6N^{\ominus}$: 279.9178 Da).

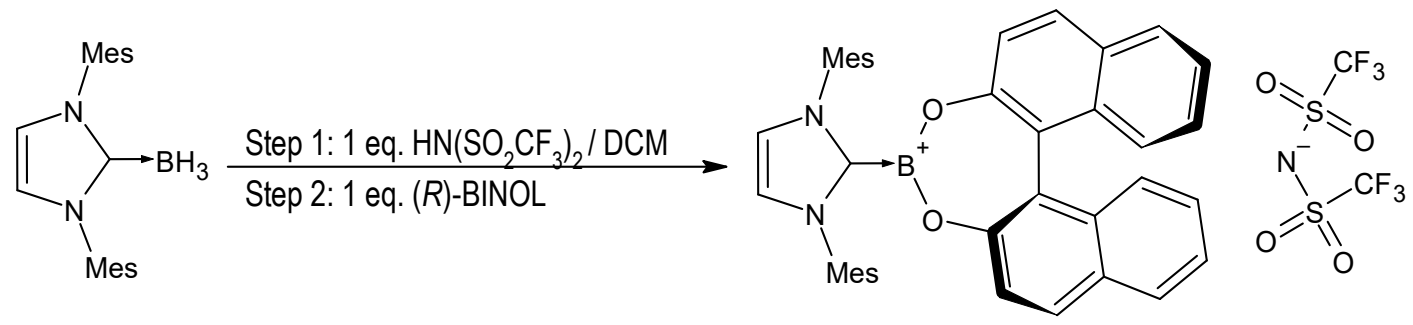

[(R_a)-IMesBO$_2$BINOL]$^{\oplus}$ [N(SO$_2$CF$_3$)$_2$]$^{\ominus}$ (***1e***): Obtained by *method 1*. Yield = 51%, white powder.
^{1}H NMR (600 MHz) *δ*/ppm (DCM-d$_2$): 7.86 (d, 2H, $^3J_{HH}$ = 8.0 Hz, Ar), 7.82 (s, 2H, imidazolylidene-CH), 7.72 (d, 2H, $^3J_{HH}$ = 8.7 Hz, Ar), 7.43 (t, 2H, $^3J_{HH}$ = 7.2 Hz, Ar), 7.35 (s, 2H, CH-Mes), 7.22 (s, 2H, CH-Mes), 7.17 (t, 2H, $^3J_{HH}$ = 7.3 Hz, Ar), 6.96 (d, 2H, $^3J_{HH}$ = 8.4 Hz, Ar), 5.69 (d, 2H, $^3J_{HH}$ = 8.7 Hz, Ar), 2.58 (s, 6H, *para*-CH$_3$), 2.42 (s, 6H, *ortho*-CH$_3$), 1.99 (s, 6H, *ortho*-CH$_3$).
^{19}F NMR (565 MHz) *δ*/ppm (DCM-d$_2$): -79.4.
^{11}B{^{1}H} NMR (193 MHz) *δ*/ppm (DCM-d$_2$): 25.1 (FWHM: 700 Hz).
^{13}C{^{1}H} NMR (151 MHz) *δ*/ppm: (DCM-d$_2$): (*C*-carbene not observed), 151.6 (*C*-O), 142.8 (*para*-CH$_3$-*C*-Mes), 135.6 (*ortho*-CH$_3$-*C*-Mes), 134.5 (*ortho*-CH$_3$-*C*-Mes), 133.0 (N-*C*-Mes), 132.0 (*C*-Ar), 131.4 (*C*H-Ar), 130.8 (*C*H-Mes), 130.5 (*C*H-Mes), 129.3 (imidazolylidene-*C*H), 128.7 (*C*H-Ar), 127.3 (*C*H-Ar), 127.1 (*C*H-Ar), 126.3 (*C*H-Ar), 120.4 (d, $^1J_{CF}$ = 321.5 Hz, *C*F$_3$), 120.0 (*C*-Ar), 119.8 (*C*H-Ar), 21.6 (*para*-*C*H$_3$), 17.8 (*ortho*-*C*H$_3$), 17.4 (*ortho*-*C*H$_3$).
HRMS [m/z]: (pos.) Found: 599.2870 Da (Calc. for molecular borenium $C_{41}H_{36}BN_2O_2^{\oplus}$: 599.2866 Da), Found: 391.2560 Da (Calc. for hydrolysis product with *i*-PrOH $C_{24}H_{32}BN_2O_2^{\oplus}$: 391.2551 Da), Found: 363.2246 Da (Calc. for hydrolysis product with MeOH $C_{22}H_{28}BN_2O_2^{\oplus}$: 363.2238 Da), Found: 305.2019 Da (Calc. for IMesH$^{\oplus}$ fragment $C_{21}H_{24}N_2H^{\oplus}$: 305.2012 Da), (neg.) Found 279.9175 Da (Calc. for triflimide anion $C_2S_2O_4F_6N^{\ominus}$: 279.9178 Da).

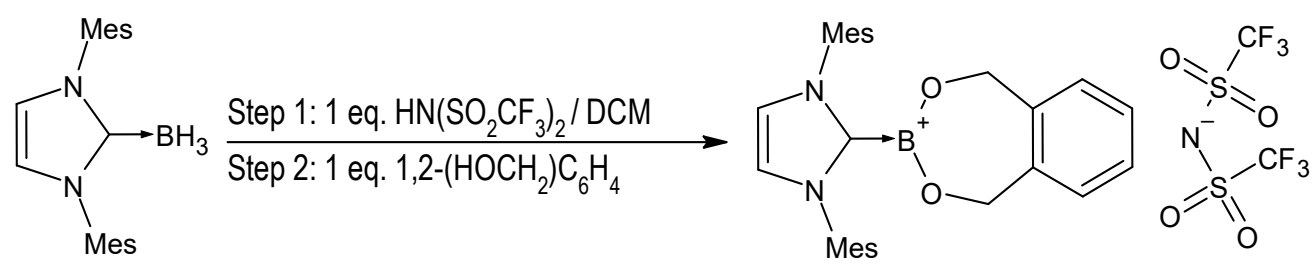

[IMesB(OCH$_2$)$_2$C$_6$H$_4$]$^{\oplus}$ [N(SO$_2$CF$_3$)$_2$]$^{\ominus}$ (***1f***): Obtained by *method 1*. Yield = 64%, yellow powder.
^{1}H NMR (600 MHz) *δ*/ppm (DCM-d$_2$): 7.42 (s, 2H, imidazolylidene-CH), 7.31 - 7.29 (m, 2H, Ar), 7.05 - 7.03 (m, 2H, Ar), 6.96, (s, 4H, CH-Mes), 4.81 (s, 4H, CH$_2$), 2.35 (s, 6H, *para*-CH$_3$), 1.93 (s, 12H, *ortho*-CH$_3$).
^{19}F NMR (565 MHz) *δ*/ppm (DCM-d$_2$): -79.5.
^{11}B{^{1}H} NMR (193 MHz) *δ*/ppm (DCM-d$_2$): 21.2 (FWHM: 190 Hz).
^{13}C{^{1}H} NMR (151 MHz) *δ*/ppm: (DCM-d$_2$): (*C*-carbene not observed) 141.9 (*para*-CH$_3$-*C*-Mes), 137.6 (*ortho*-CH$_3$-*C*-Mes), 134.7 (*C*H-Ar), 132.0 (*C*-Ar), 132.0 (N-*C*-Mes), 130.0 (*C*H-Mes), 129.3 (imidazolylidene-*C*H), 126.0 (*C*H-Ar), 120.44 (q, $^1J_{CF}$ = 321.4 Hz, *C*F$_3$), 68.4 (*C*H$_2$), 21.4 (*para*-*C*H$_3$), 17.3 (*ortho*-*C*H$_3$).
HRMS [m/z]: (pos.) Found: 451.2563 Da (Calc. for molecular borenium $C_{29}H_{32}BN_2O_2^{\oplus}$: 451.2551 Da), (neg.) 279.9157 Da (Calc. for triflimide anion $C_2S_2O_4F_6N^{\ominus}$: 279.9178 Da).

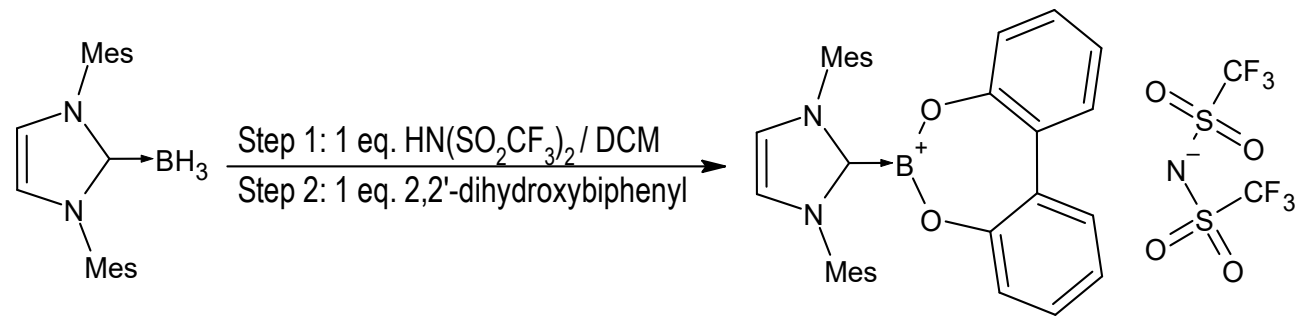

[IMesBO$_2$C$_{12}$H$_8$]$^{\oplus}$ [N(SO$_2$CF$_3$)$_2$]$^{\ominus}$ (***1g***): Obtained as racemate by *method 1*. Yield = 59%, white powder.
^{1}H NMR (600 MHz) *δ*/ppm (DCM-d$_2$): 7.77 (s, 2H, imidazolylidene-CH), 7.36 (dd, 2H, $^3J_{HH}$ = 7.8 Hz, $^4J_{HH}$ = 1.5 Hz, Ar), 7.21 (s, 4H, CH-Mes), 7.15 (dd, 2H, $^3J_{HH}$ = 7.6 Hz, $^4J_{HH}$ = 1.0 Hz, Ar), 7.08 (dd, 2H, $^3J_{HH}$ = 7.7 Hz, $^4J_{HH}$ = 1.2 Hz, Ar), 5.49 (dd, 2H, $^3J_{HH}$ = 8.2 Hz, $^4J_{HH}$ = 1.0 Hz, Ar), 2.48 (s, 6H, *para*-CH$_3$), 2.11 (s, 12H, *ortho*-CH$_3$).
^{19}F NMR (565 MHz) *δ*/ppm (DCM-d$_2$): -79.5.
^{11}B{^{1}H} NMR (193 MHz) *δ*/ppm (DCM-d$_2$): 22.5 (FWHM: 450 Hz).
^{13}C{^{1}H} NMR (151 MHz) *δ*/ppm: (DCM-d$_2$): (*C*-carbene not observed), 151.9 (*C*-O), 142.5 (*para*-CH$_3$-*C*-Mes), 134.8 (*ortho*-CH$_3$-*C*-Mes), 133.1 (N-*C*-Mes), 130.8 (*C*H-Ar), 130.6 (*C*H-Ar), 130.5 (*C*H-Mes), 129.1 (*C*H-Ar), 127.1 (imidazolylidene-*C*H), 126.8 (*C*H-Ar), 120.9 (*C*-Ar), 120.5 (q, $^1J_{CF}$ = 322.0 Hz, *C*F$_3$), 21.5 (*para*-*C*H$_3$), 17.6 (*ortho*-*C*H$_3$).
HRMS [m/z]: (pos.) Found: 499.2563 Da (Calc. for molecular borenium $C_{33}H_{32}BN_2O_2^{\oplus}$: 499.2551 Da), Found: 349.2090 Da (Calc. for fragment $C_{21}H_{26}BN_2O_2^{\oplus}$: 349.2082 Da), (neg.) Found: 279.9157 Da (Calc. for triflimide anion $C_2S_2O_4F_6N^{\ominus}$: 279.9178 Da).

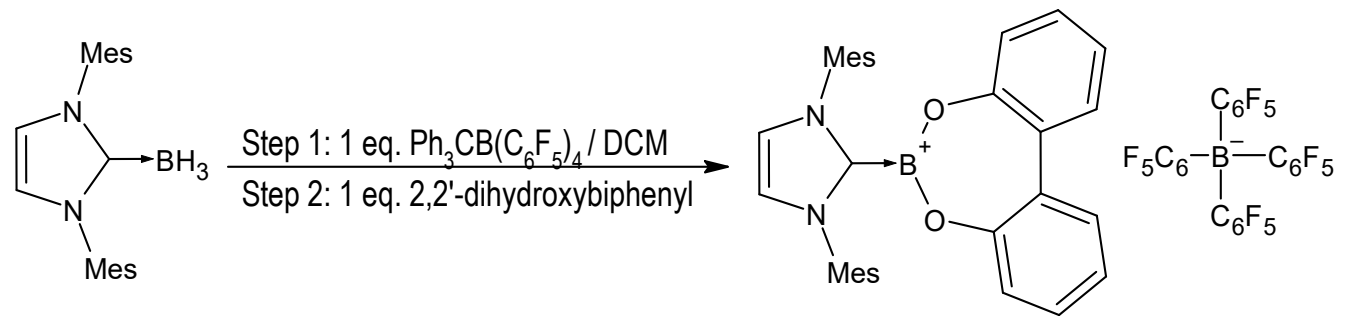

$[IMesBO_2C_{12}H_8]^{\oplus}$ $[B(C_5F_6)_4]^{\ominus}$ (***1g'***): Obtained as racemate by *method 1* (replacing catechol with 2,2'-dihydroxybiphenyl and $HNTf_2$ with $Ph_3C^{\oplus}B(C_6F_5)_4^{\ominus}$). Yield = 68%, yellow powder.
^{1}H NMR (600 MHz) *δ*/ppm (DCM-d2): 7.65 (s, 2H, imidazolylidene-CH), 7.39 (d, 2H, $^3J_{HH}$ = 7.6 Hz, Ar), 7.24 (s, 4H, CH-Mes), 7.17 (t, 2H, $^3J_{HH}$ = 7.5 Hz, Ar), 7.10 (t, 2H, $^3J_{HH}$ = 7.6 Hz, Ar), 5.51 (d, 2H, $^3J_{HH}$ = 8.1 Hz, Ar), 2.50 (s, 6H, *para*-CH_3), 2.12 (s, 12H, *ortho*-CH_3).
^{19}F NMR (565 MHz) *δ*/ppm (DCM-d2): -133.0 (m, 8F), -163.56 (t, 4F, $^3J_{FF}$ = 20.4 Hz), -167.44 (t, 8F, $^3J_{FF}$ = 17.2 Hz).
^{11}B{^{1}H} NMR (193 MHz) *δ*/ppm (DCM-d2): 22.2 (FWHM: 420 Hz) (borenium), -17.7 (FWHM: 20 Hz) (borate).
^{13}C{^{1}H} NMR (151 MHz) *δ*/ppm: (DCM-d2): (*C*-B$^{\ominus}$ not observed), (*C*-carbene not observed), 151.8 (*C*-O), 148.71 (dm, $^1J_{CF}$ = 241.6 Hz, *C*F), 142.7 (*para*-CH_3-*C*-Mes), 138.8 (dm, $^1J_{CF}$ ≈ 250 Hz, *C*F), 136.8 (dm, $^1J_{CF}$ ≈ 250 Hz, *C*F), 134.7 (*ortho*-CH_3-*C*-Mes), 132.9 (N-*C*-Mes), 130.9 (*C*H-Ar), 130.6 (*C*H-Ar), 130.6 (*C*H-Mes), 128.3 (*C*H-Ar), 127.1 (imidazolylidene-*C*H), 126.9 (*C*H-Ar), 120.9 (*C*-Ar), 21.4 (*para*-*C*H_3), 17.6 (*ortho*-*C*H_3),
HRMS [m/z]: (pos.) Found: 499.2576 Da (Calc. for molecular borenium $C_{33}H_{32}BN_2O_2^{\oplus}$: 499.252 Da), Found 391.2575 Da (Calc. for hydrolysis product with *i*-PrOH $C_{24}H_{32}BN_2O_2^{\oplus}$: 391.2551 Da), Found 305.2029 Da (Calc. for $IMesH^{\oplus}$ fragment $C_{21}H_{24}N_2H^{\oplus}$: 305.2012 Da), (neg.) Found 678.9786 Da (Calc. for $^{\ominus}B(C_6F_5)_4$ borate anion: 678.9780 Da).

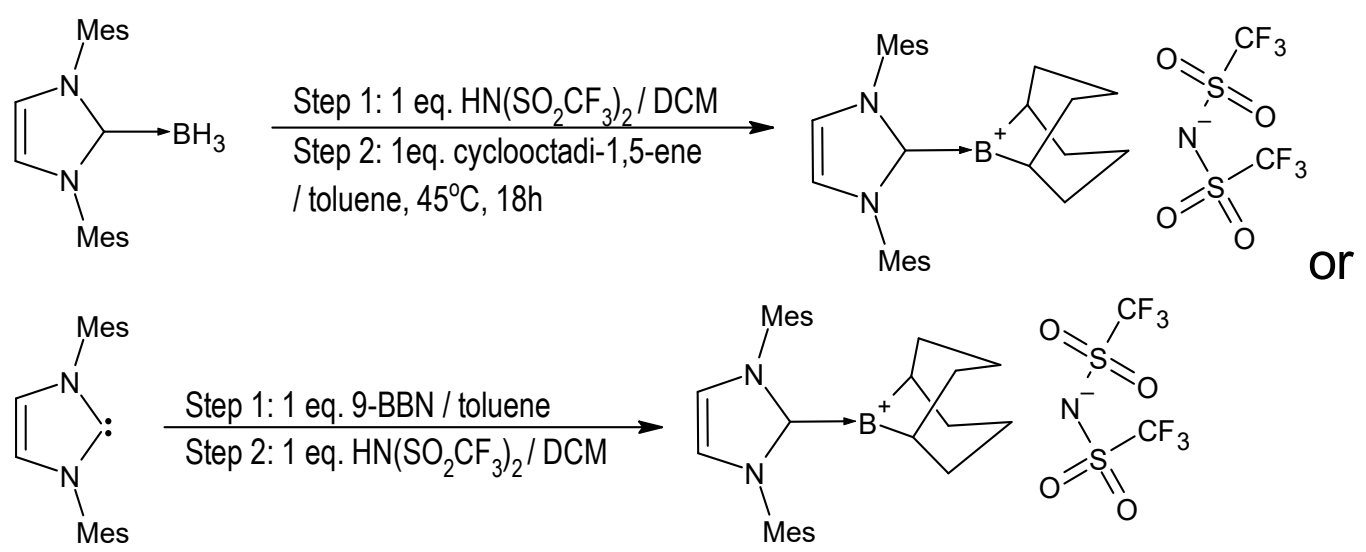

$[IMesBC_8H_{14}]^{\oplus}$ $[N(SO_2CF_3)_2]^{\ominus}$ (***1h***): Obtained by *method 1* (the second step with cyclooctadiene was conducted at 45°C for 18h in toluene instead of DCM to accelerate the rearrangement of the product from 1,4-isomer to 1,5-isomer), Yield = 50% and *method 2*, Y = 90%, yellow powder.
^{1}H NMR (600 MHz) *δ*/ppm (DCM-d2): 7.75 (s, 2H, imidazolylidene-CH), 7.11 (s, 4H, CH-Mes), 2.39 (s, 6H, *para*-CH_3), 2.11 (s, 12H, *ortho*-CH_3), 1.82 - 1.75 (m, 6H, CH_2, CH_2, CH), 1.36 - 1.33 (m, 2H, CH_2), 1.19 - 1.12 (m, 4H, CH_2), 1.08 - 1.03 (m, 2H, CH_2).
^{19}F NMR (565 MHz) *δ*/ppm (DCM-d2): -79.5.
^{11}B{^{1}H} NMR (193 MHz) *δ*/ppm (DCM-d2): 83.0 (FWHM: 280 Hz).
^{13}C{^{1}H} NMR (151 MHz) *δ*/ppm: (DCM-d2): (*C*-carbene not observed), 142.9 (*para*-CH_3-*C*-Mes), 134.7 (*ortho*-CH_3-*C*-Mes), 132.1 (N-*C*-Mes), 130.6 (*C*H-Mes), 128.6 (imidazolylidene-*C*H), 120.4 (d, $^1J_{CF}$ = 321.9 Hz, *C*F_3), 35.3 (*C*H_2), 22.7 (*C*H), 21.45 (*para*-*C*H_3), 17.8 (*ortho*-*C*H_3).
HRMS [m/z]: (pos.) Found 425.3133 Da (Calc. for molecular borenium $C_{29}H_{38}BN_2^{\oplus}$: 425.3123 Da), (neg.) Found 279.9180 Da (Calc. for triflimide anion $C_2S_2O_4F_6N^{\ominus}$: 279.9178 Da).

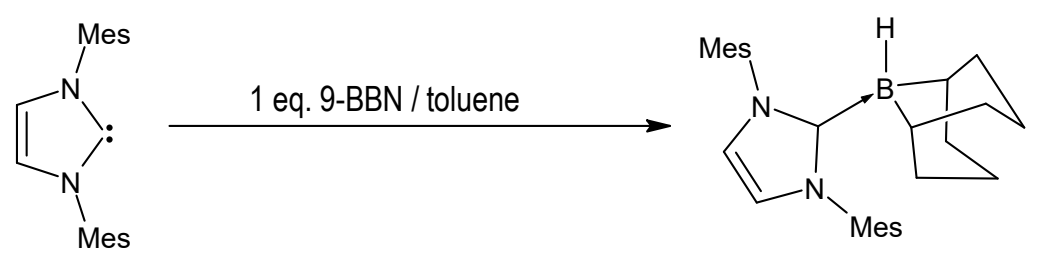

$IMesB(H)C_8H_{14}$ (***1h*H**): Obtained in the first step of *method 2*, yield = 99%, white powder.
^{1}H NMR (600 MHz) *δ*/ppm (DCM-d2): 6.94 (s, 4H, CH-Mes), 6.82 (s, 2H, imidazolylidene-CH), 2.32 (s, 6H, *para*-CH_3), 2.12 (s, 12H, *ortho*-CH_3), 1.57 - 1.48 (m, 2H, CH_2), 1.47 - 1.29 (4H, CH_2, CH), 1.22 - 1.17 (2H, CH,), 1.17 - 1.08 (m, 4H, CH_2), 1.06 - 0.99 (m, 2H, CH_2), 0.18 (s, 1H, BH).
^{11}B NMR (193 MHz) *δ*/ppm (DCM-d2): -16.9 (d, $^1J_{BH}$ = 78.6 Hz).
^{11}B{^{1}H} NMR (193 MHz) *δ*/ppm (DCM-d2): -16.9 (FWHM: 60 Hz).

$^{13}C\{^1H\}$ NMR (151 MHz) δ/ppm: (DCM-d_2): (*C*-carbene not observed), 139.5 (*para*-CH_3-*C*-Mes), 136.0 (N-*C*-Mes), 135.9 (*ortho*-CH_3-*C*-Mes), 129.1 (*C*H-Mes), 122.5 (imidazolylidene-*C*H), 37.5 (*C*H_2), 32.2 (*C*H_2), 26.1 (*C*H), 24.4 (*C*H_2), 21.4 (*para*-*C*H_3), 18.4 (*ortho*-*C*H_3).
HRMS [m/z]: (pos.) Found 425.3117 Da (Calc. for molecular borenium $C_{29}H_{38}BN_2^{\oplus}$: 425.3123 Da).

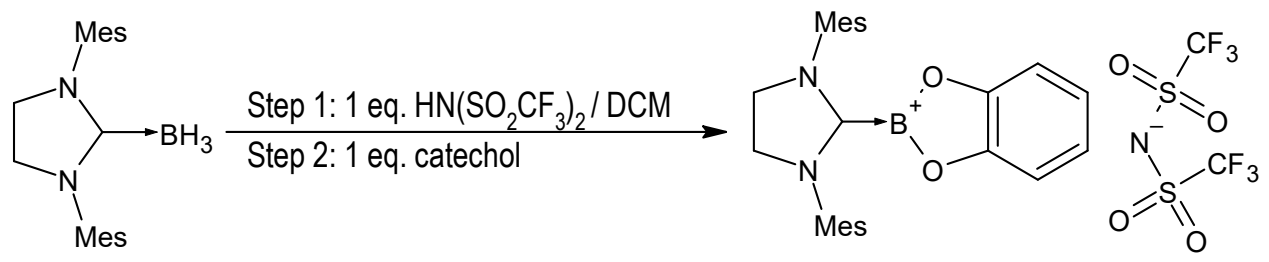

[SIMesBCat]⊕[$N(SO_2CF_3)_2$]⊖ (***2b***): Obtained by *method 1.* Yield = 62%, white powder.
^1H NMR (600 MHz) δ/ppm (DCM-d_2): 7.18 - 7.12 (m, 4H, CH-catechol), 6.99 (s, 4H, CH-Mes), 4.58 (s, 4H, imidazolidinylidene-CH_2), 2.42 (s, 12H, *ortho*-CH_3), 2.26 (s, 6H, *para*-CH_3).
^{19}F NMR (565 MHz) δ/ppm (DCM-d_2): -79.5.
$^{11}B\{^1H\}$ NMR (193 MHz) δ/ppm (DCM-d_2): 25.0 (FWHM: 300 Hz).
$^{13}C\{^1H\}$ NMR (151 MHz) δ/ppm: (DCM-d_2): (*C*-carbene not observed), 147.1 (*C*-O), 142.1 (*para*-CH_3-*C*-Mes), 135.81 (*ortho*-CH_3-*C*-Mes), 130.8 (*C*H-Mes), 130.4 (N-*C*-Mes), 125.4 (*C*H-Ar), 120.5 (q, $^1J_{CF}$ = 321.5 Hz, *C*F_3), 114.2 (*C*-Ar), 53.5 (imidazolidinylidene-*C*H_2), 21.4 (*para*-*C*H_3), 17.8 (*ortho*-*C*H_3).
HRMS [m/z] (pos.) Found 425.2416 Da (Calc. for molecular borenium $C_{27}H_{30}BN_2O_2^{\oplus}$: 425.2395 Da), Found 365.2400 Da (fragment $C_{22}H_{30}BN_2O_2^{\oplus}$: 365.2395 Da), Found 351.2257 Da (Calc. for fragment $C_{21}H_{28}BN_2O_2^{\oplus}$: 351.2238 Da), 307.2184 Da (Calc. for IMesH⊕ fragment $C_{21}H_{27}N_2^{\oplus}$: 307.2169 Da), Found 349.2090 Da (Calc. for fragment $C_{21}H_{26}BN_2O_2^{\oplus}$: 349.2082 Da), (neg.) Found 279.9180 Da (Calc. for triflimide anion $C_2S_2O_4F_6N^{\ominus}$: 279.9178 Da).

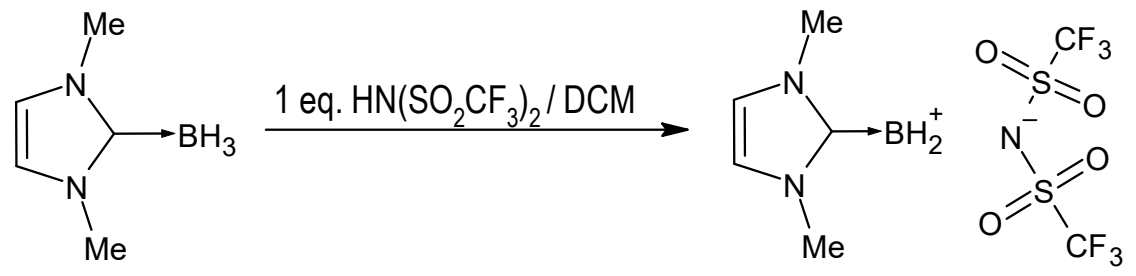

[IMe_2BH_2]⊕[$N(SO_2CF_3)_2$]⊖ (***3a***): Obtained in the first step of *method 1.* Yield = 97%, white powder.
^1H NMR (600 MHz) δ/ppm (DCM-d_2): 7.00 (s, 2H, imidazolylidene-CH), 3.86 (s, 6H, CH_3), 2.84 (br, 2H, BH_2).
^{19}F NMR (565 MHz) δ/ppm (DCM-d_2): -73.9.
^{11}B NMR (193 MHz) δ/ppm (DCM-d_2): -18.1 (t, $^1J_{BH}$ = 89.6 Hz).
$^{11}B\{^1H\}$ NMR (193 MHz) δ/ppm (DCM-d_2): -18.0 (FWHM: 110 Hz).
$^{13}C\{^1H\}$ NMR (151 MHz) δ/ppm: (benzene-d_6): 159.1 (*C*-carbene), 121.2 (imidazolylidene-*C*H), 120.18 (q, $^1J_{CF}$ = 324.6 Hz, *C*F_3), 35.7 (*C*H_3).
EA: Calc. for $C_7H_{10}BF_6N_3O_4S_2$ C: 21.61%, H: 2.59%, N: 10.80%, Found C: 21.45%, H: 2.50%, N: 11.23%.

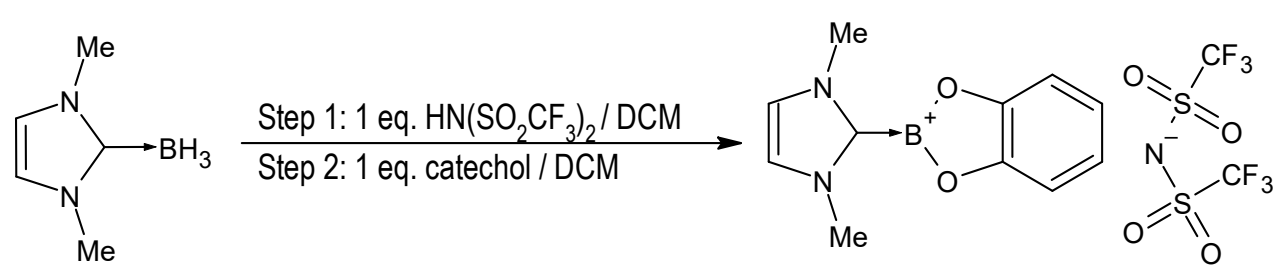

[IMe_2BCat]⊕[$N(SO_2CF_3)_2$]⊖ (***3b***): Obtained by *method 1.* Yield = 79%, off white powder.
^1H NMR (600 MHz) δ/ppm (DCM-d_2): 7.57 (s, 2H, imidazolylidene-CH), 7.49 (dd, 2H, $^3J_{HH}$ = 6.0 Hz, $^4J_{HH}$ = 3.4 Hz, CH-catechol), 7.33 (dd, 2H, $^3J_{HH}$ = 6.0 Hz, $^4J_{HH}$ = 3.3 Hz, CH-catechol), 4.30 (s, 6H, CH_3).
^{19}F NMR (565 MHz) δ/ppm (DCM-d_2): -79.5.
$^{11}B\{^1H\}$ NMR (193 MHz) δ/ppm (DCM-d_2): 25.1 (FWHM: 200 Hz).
$^{13}C\{^1H\}$ NMR (151 MHz) δ/ppm: (DCM-d_2): (*C*-carbene not observed), 147.3 (*C*-O), 127.9 (*C*H-Ar), 125.3 (imidazolylidene-*C*H), 120.32 (q, $^1J_{CF}$ = 321.3 Hz, *C*F_3), 114.1 (*C*H-Ar), 38.8 (*C*H_3).
EA: Calc. for $C_{13}H_{12}BF_6N_3O_6S_2$ C: 31.53%, H: 2.44%, N: 8.49%, Found C: 31.41%, H: 2.47%, N: 8.60%.

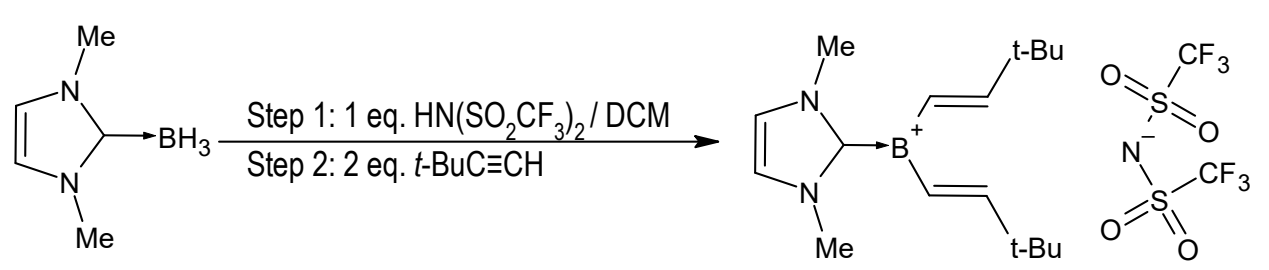

[$IMe_2B(CH{=}CH\text{-}tBu)_2$]⊕ [$N(SO_2CF_3)_2$]⊖ (***3c***): Obtained by *method 1.* Yield = 33%, yellow powder.

^{1}H NMR (600 MHz) δ/ppm (CDCl$_3$): 7.53 (s, 2H, imidazolylidene-CH), 6.77 (d, 2H, $^{3}J_{HH}$ = 17.9 Hz, B-C*H*=CH), 6.51 (d, 2H, $^{3}J_{HH}$ = 17.9 Hz, B-CH=C*H*), 3.74 (s, 6H, CH$_3$), 1.11 (s, 18H, *t*-Bu).
^{19}F NMR (565 MHz) δ/ppm (CDCl$_3$): -78.7.
$^{11}B\{^{1}H\}$ NMR (193 MHz) δ/ppm (DCM-d$_2$): 55.3. (FWHM: 620 Hz).
$^{13}C\{^{1}H\}$ NMR (151 MHz) δ/ppm (CDCl$_3$): 179.4 (*C*H=CH–*t*-Bu), 151.9 (*C*-carbene), 128.1 (CH=*C*H–*t*-Bu), 125.5 (imidazolylidene-*C*H), 120.0 (q, $^{1}J_{CF}$ = 321.3 Hz, *C*F$_3$), 37.2 (CH$_3$), 37.0 (*t*-Bu-*C*), 28.3 (*t*-Bu-*C*H$_3$).
HRMS [m/z] (pos.) Found 273.2522 Da (Calc. for molecular borenium $C_{17}H_{30}BN_2^{\oplus}$: 273.2497 Da), (neg.) Found 279.9166 Da (Calc. for triflimide anion $C_2S_2O_4F_6N^{\ominus}$: 279.9178 Da).

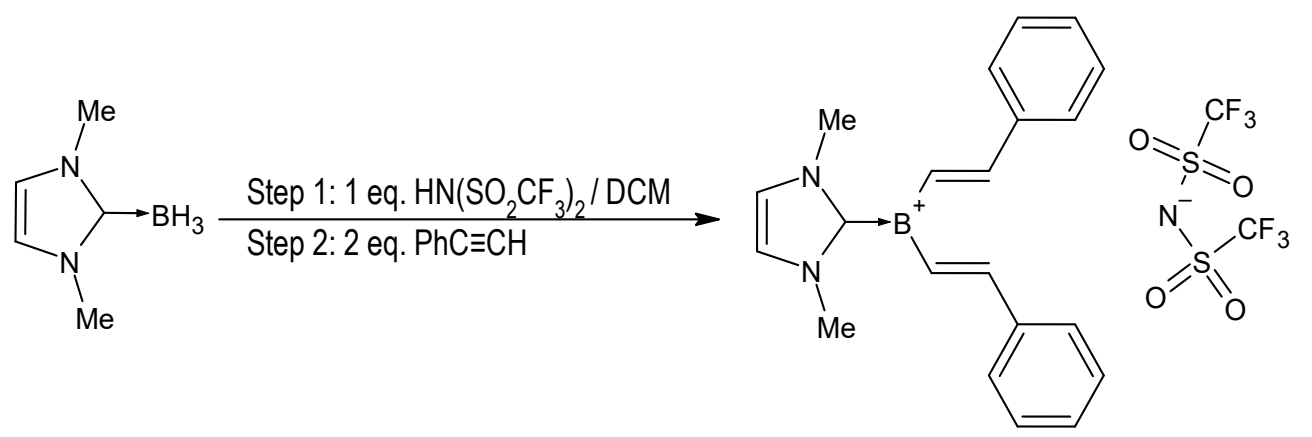

[IMe$_2$B(CH=CH–Ph)$_2$]$^{\oplus}$ [N(SO$_2$CF$_3$)$_2$]$^{\ominus}$ (***3d***): Obtained by *method 1*. Yield = 63%, brown solid.
^{1}H NMR (600 MHz) δ/ppm (DCM-d$_2$): 7.73 (d, 4H, $^{3}J_{HH}$ = 6.8 Hz, Ar), 7.53 (s, 2H, imidazolylidene-CH), 7.51 - 7.45 (m, 10H, Ar and vinyl CH), 3.80 (s, 6H, CH$_3$).
^{19}F NMR (565 MHz) δ/ppm (DCM-d$_2$): -79.3.
$^{11}B\{^{1}H\}$ NMR (193 MHz) δ/ppm (DCM-d$_2$): 53.2 (FWHM: 870 Hz).
$^{13}C\{^{1}H\}$ NMR (151 MHz) δ/ppm: (DCM-d$_2$): 161.7 (*C*H=CH–Ph), 152.9 (*C*-carbene), 136.3 (Ph-*C*-ipso), 132.8 (*para*-CH-Ar), 131.2 (CH=*C*H–Ph), 129.8 (*C*H-Ar), 129.7 (*C*H-Ar), 125.7 (imidazolylidene-*C*H), 120.4 (q, $^{1}J_{CF}$ = 321.4 Hz, *C*F$_3$), 37.6 (*C*H$_3$).
HRMS [m/z] (pos.) Found 313.1902 Da (Calc. for molecular borenium $C_{21}H_{22}BN_2^{\oplus}$: 313.1871 Da), (neg.) Found 279.9164 Da (Calc. for triflimide anion $C_2S_2O_4F_6N^{\ominus}$: 279.9178 Da).

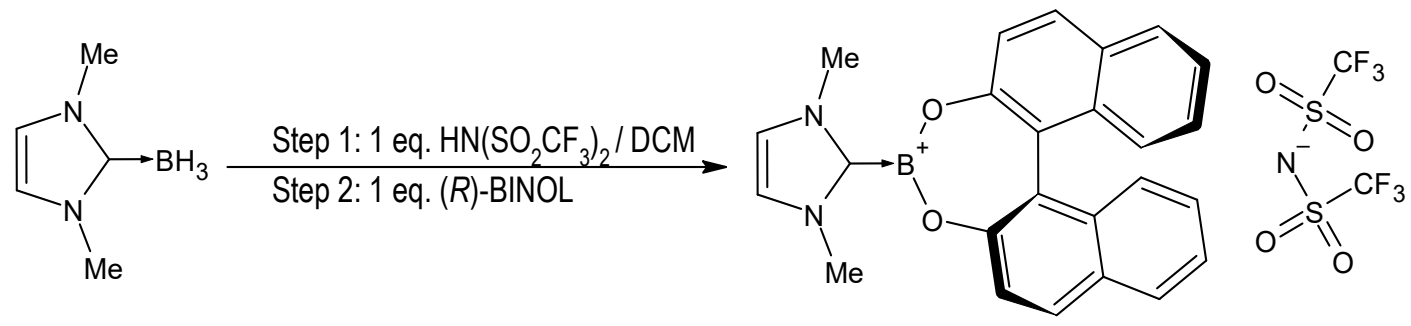

[(*R*$_a$)-IMe$_2$BO$_2$BINOL]$^{\oplus}$ [N(SO$_2$CF$_3$)$_2$]$^{\ominus}$ (***3e***): Obtained by *method 1*. Yield = 57%, white powder.
^{1}H NMR (600 MHz) δ/ppm (DCM-d$_2$): 8.03 (d, 2H, $^{3}J_{HH}$ = 8.8 Hz, Ar), 7.96 (d, 2H, $^{3}J_{HH}$ = 8.1 Hz, Ar), 7.51 - 7.46 (m, 2H, Ar), 7.47 (s, 2H, imidazolylidene-CH), 7.43 (d, 2H, $^{3}J_{HH}$ = 8.8 Hz, Ar), 7.26 (t, 2H, $^{3}J_{HH}$ = 7.6 Hz, Ar), 7.17 (d, 2H, $^{3}J_{HH}$ = 8.6 Hz, Ar), 4.31 (s, 6H, N-CH$_3$).
^{19}F NMR (565 MHz) δ/ppm (DCM-d$_2$): -79.4.
$^{11}B\{^{1}H\}$ NMR (193 MHz) δ/ppm (DCM-d$_2$): 25.5 (FWHM: 740 Hz).
$^{13}C\{^{1}H\}$ NMR (151 MHz) δ/ppm: (DCM-d$_2$): (*C*-carbene not observed), 152.2 (*C*-O), 133.2 (*C*-Ar), 132.0 (*C*H-Ar), 131.7 (*C*H-Ar), 128.9 (*C*H-Ar), 127.6 (*C*-Ar), 127.4 (*C*H-Ar), 127.2 (*C*H-Ar), 126.2 (*C*H-Ar), 120.9 (imidazolylidene-*C*H), 120.5 (*C*-Ar), 120.3 (q, $^{1}J_{CF}$ = 321.5 Hz, *C*F$_3$), 39.4 (N-*C*H$_3$).
HRMS [m/z] (pos.) Found 391.1622 Da (Calc. for molecular borenium $C_{25}H_{20}BN_2O_2^{\oplus}$: 391.1612 Da), Found 285.0917 Da (Calc. for fragment $C_{20}H_{13}O_2^{\oplus}$: 285.0910 Da), Found 155.0990 Da (Calc. for fragment $C_6H_{12}BN_2O_2^{\oplus}$: 155.0986 Da), Found 141.0833 Da (Calc. for fragment $C_5H_{10}BN_2O_2^{\oplus}$: 141.0830 Da), (neg.) Found 279.9176 Da (Calc. for triflimide anion $C_2S_2O_4F_6N^{\ominus}$: 279.9178 Da).

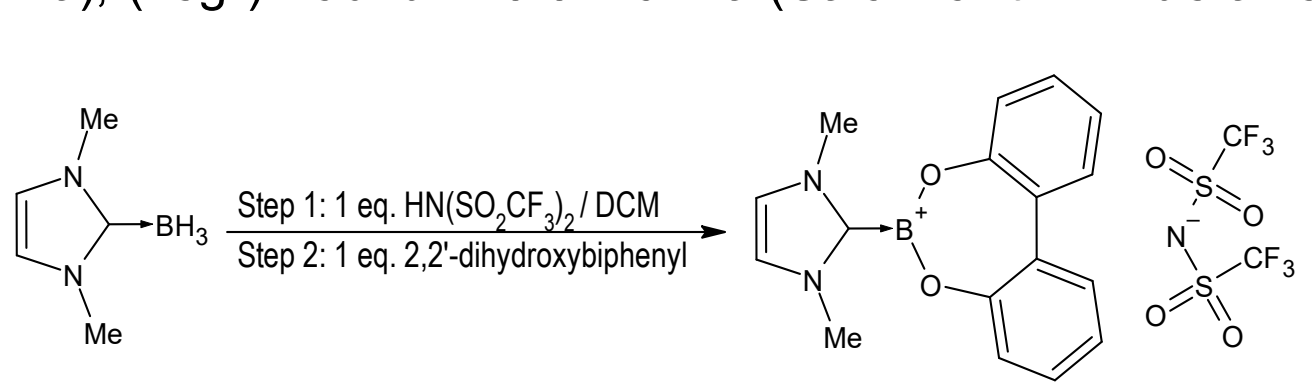

[IMe$_2$BO$_2$C$_{12}$H$_8$]$^{\oplus}$ [N(SO$_2$CF$_3$)$_2$]$^{\ominus}$ (***3g***): Obtained as racemate by *method 1*. Yield = 88%, light brown powder.
^{1}H NMR (600 MHz) δ/ppm (DCM-d$_2$): 7.59 (dd, 2H, $^{3}J_{HH}$ = 7.8 Hz, $^{4}J_{HH}$ = 1.6 Hz, Ar), 7.47, (s, 2H, imidazolylidene-CH) 7.41 (dd, 2H, $^{3}J_{HH}$ = 7.8 Hz, $^{4}J_{HH}$ = 1.6 Hz, Ar), 7.33 (dd, 2H, $^{3}J_{HH}$ = 7.8 Hz, $^{4}J_{HH}$ = 1.1 Hz, Ar), 7.14 (dd, 2H $^{3}J_{HH}$ = 8.1 Hz, $^{4}J_{HH}$ = 1.0 Hz, Ar), 4.28 (s, 6H, CH$_3$).
^{19}F NMR (565 MHz) δ/ppm (DCM-d$_2$): -79.4.
$^{11}B\{^{1}H\}$ NMR (193 MHz) δ/ppm (DCM-d$_2$): 23.2 (FWHM: 300 Hz).

$^{13}C\{^1H\}$ NMR (151 MHz) δ/ppm: (DCM-d$_2$): (*C*-carbene not observed), 152.7 (*C*-O), 131.2 (*C*H-Ar), 130.8 (*C*H-Ar), 127.7 (*C*-Ar), 127.4 (imidazolylidene-*C*H), 126.8 (*C*H-Ar), 121.6 (*C*H-Ar), 120.4 (d, $^1J_{CF}$ = 321.3 Hz, *C*F$_3$), 39.4 (*C*H$_3$).
HRMS [m/z] (pos.) Found 291.1305 Da (Calc. for molecular borenium $C_{17}H_{16}BN_2O_2^{\oplus}$: 291.1299 Da), (neg.) Found 279.9178 Da (Calc. for triflimide anion $C_2S_2O_4F_6N^{\ominus}$: 279.9178 Da).

CATALYTIC EXPERIMENTS

HOMOCOUPLING OF BIS(4-METHYLPHENYL)DIAZOMETHANE

The title borenium catalysts were initially screened against the homocoupling of bis(4-methylphenyl)-diazomethane at NMR scale, preparing 20 mg solutions of the starting material in J. Young tubes inside a glovebox and adding a variable amount of each catalyst. For our series of borenium compounds the process is selective or yields a mixture of 1,1,2,2-tetra-p-tolylethene and 1,2-bis(di-4-tolylmethylene)-hydrazine in a variable ratio. The NMR spectra were recorded after 2 and 24 h. Ratio of the products was calculated from the integration of the representative signals (see **Table S1**). Reference spectra were recorded for substances isolated from large-scale experiments and compared with literature data.

Preparation of *1,1,2,2-tetra-p-tolylethene*: Inside a glovebox, 100 mg (0.45 mmol, 1 equiv.) of bis(4-methylphenyl)diazomethane was placed in a Schlenk flask and dissolved in 8 mL of dry CHCl$_3$. 16 mg (0.023 mmol, 0.05 equiv.) of ***1b*** was added and the solution was stirred for 30 min at room temperature. The reaction mixture was taken out of the glovebox and the crude product was purified on a very short silica column (elution with hexane:ethyl acetate 5:3) to give the ethene product in 99% yield.

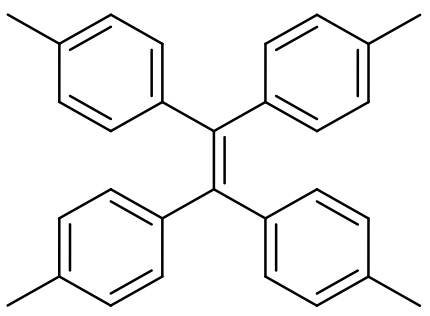

^1H NMR (600 MHz) δ/ppm (CDCl$_3$): 6.90 (m, 16H, Ar), 2.26 (s, 12H, CH$_3$).
$^{13}C\{^1H\}$ NMR (151 MHz) δ/ppm: (CDCl$_3$): 141.5 (*C*H-Ar), 140.0 (ethen), 135.8 (*C*H-Ar), 131.4 (*C*H-Ar), 128.5 (*C*H-Ar), 21.3 (*C*H$_3$).

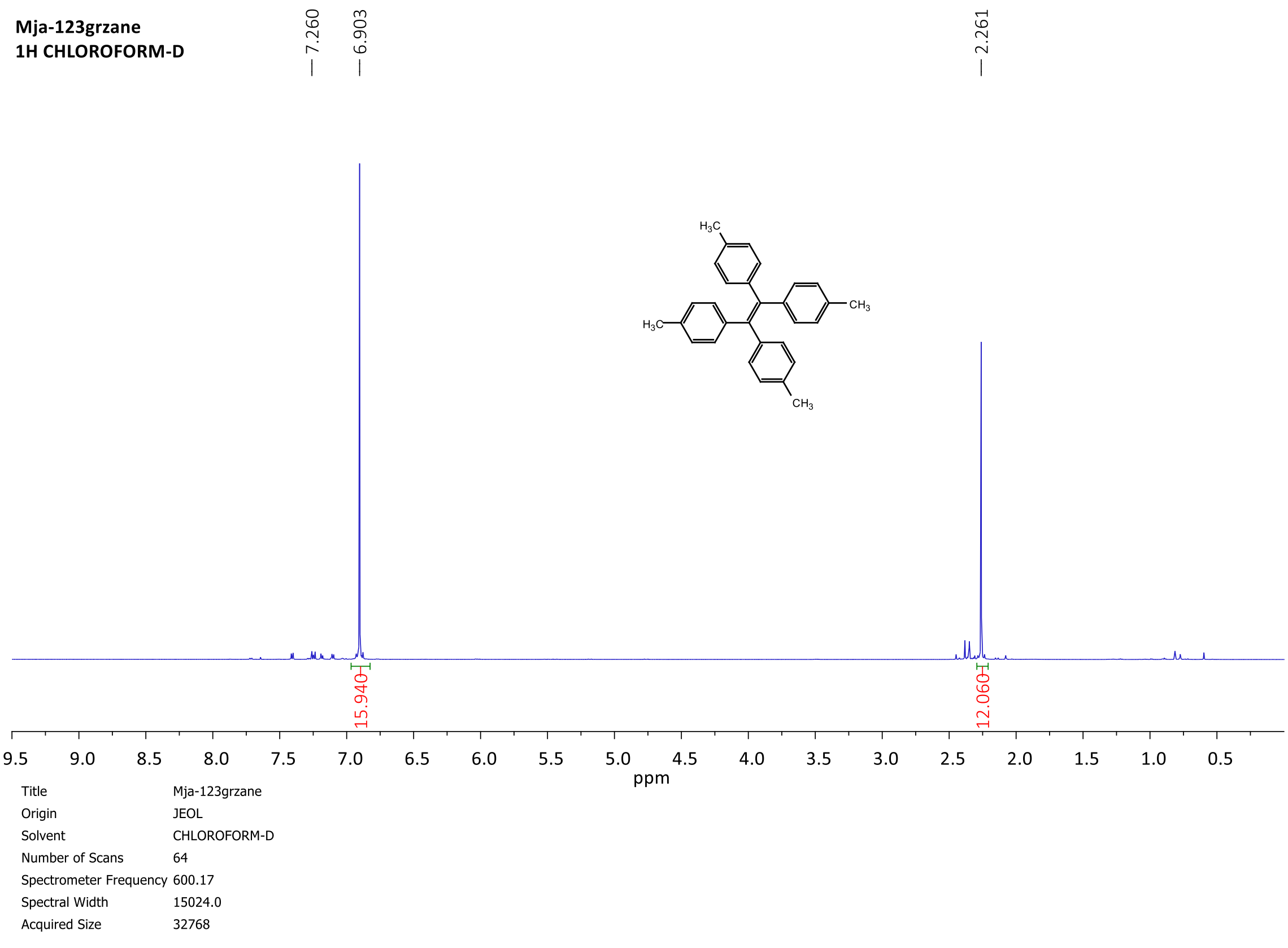

^1H NMR for 1,1,2,2-tetra-*p*-tolylethene.

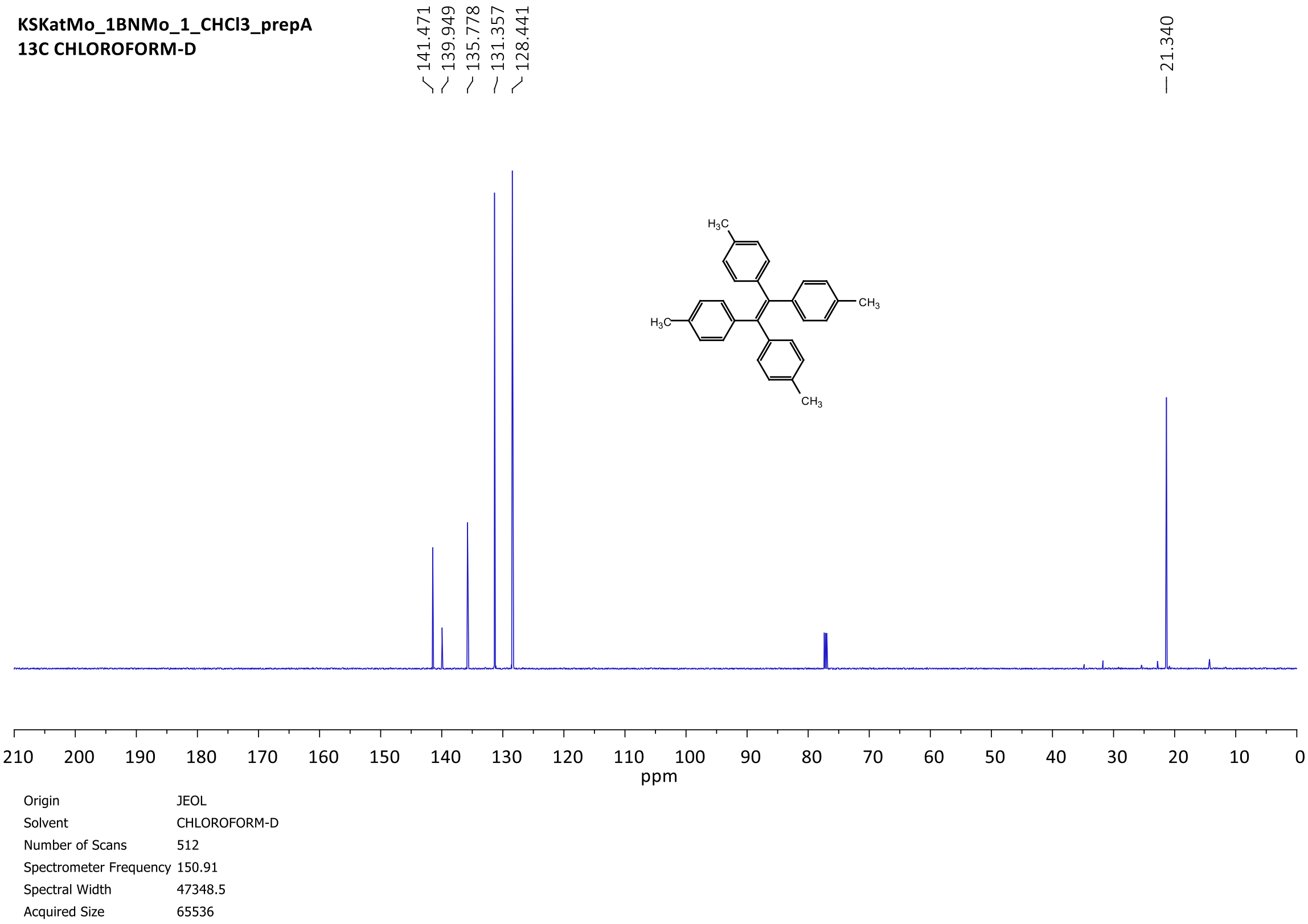

$^{13}C\{^{1}H\}$ NMR for 1,1,2,2-tetra-*p*-tolylethene.

Preparation of *1,2-Bis(di-4-tolylmethylene)hydrazine*: Inside a glovebox, 100 mg (0.45 mmol, 1 equiv.) of bis(4-methylphenyl)diazomethane was placed in a Schlenk flask and dissolved in 8 mL of dry $CHCl_3$. 11 mg (0.023 mmol, 0.05 equiv.) of ***3b*** was added and the solution was stirred for 30 min. The reaction mixture was taken out of the glovebox and the crude product was purified on a very short silica column (elution with hexane:ethyl acetate 1:1) to give the azine product in 95% yield.

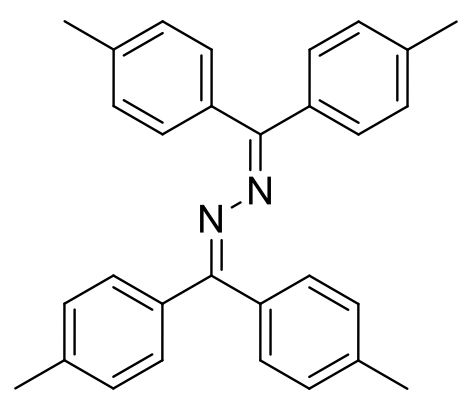

^{1}H NMR (600 MHz) δ/ppm ($CDCl_3$): 7.43 (d, 4H, $^{3}J_{HH}$ = 8.2 Hz, Ar), 7.26 (d, 4H, $^{3}J_{HH}$ = 8.1 Hz, Ar), 7.20 (d, 4H, $^{3}J_{HH}$ = 8.0 Hz, Ar), 7.12 (d, 4H, $^{3}J_{HH}$ = 8.1 Hz, Ar), 2.40 (s, 6H, CH_3), 2.37 (6H, CH_3).

$^{13}C\{^{1}H\}$ NMR (151 MHz) δ/ppm: ($CDCl_3$): 159.0 (N=*C*), 139.6 (*C*H-Ar), 138.5 (*C*H-Ar), 136.0 (*C*H-Ar), 132.8 (*C*H-Ar), 129.7 (*C*H-Ar), 128.8 (*C*H-Ar), 128.8 (*C*H-Ar), 128.5 (*C*H-Ar), 21.6 (*C*H_3), 21.5 (*C*H_3).

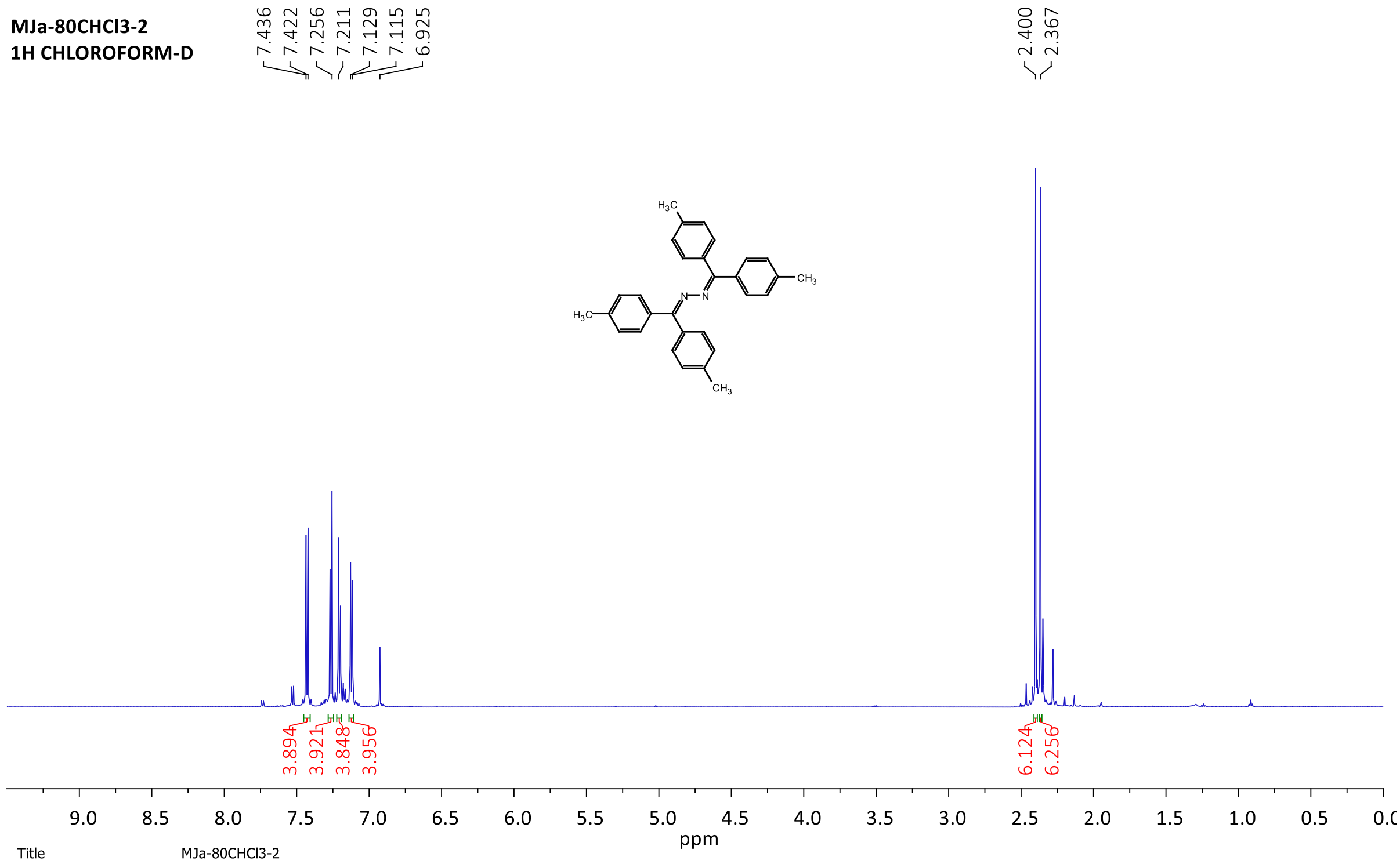

Title	MJa-80CHCl3-2
Origin	JEOL
Solvent	CHLOROFORM-D
Number of Scans	64
Spectrometer Frequency	600.17
Spectral Width	22482.0
Acquired Size	16384

^{1}H NMR for 1,2-bis(di-4-tolylmethylene)hydrazine.

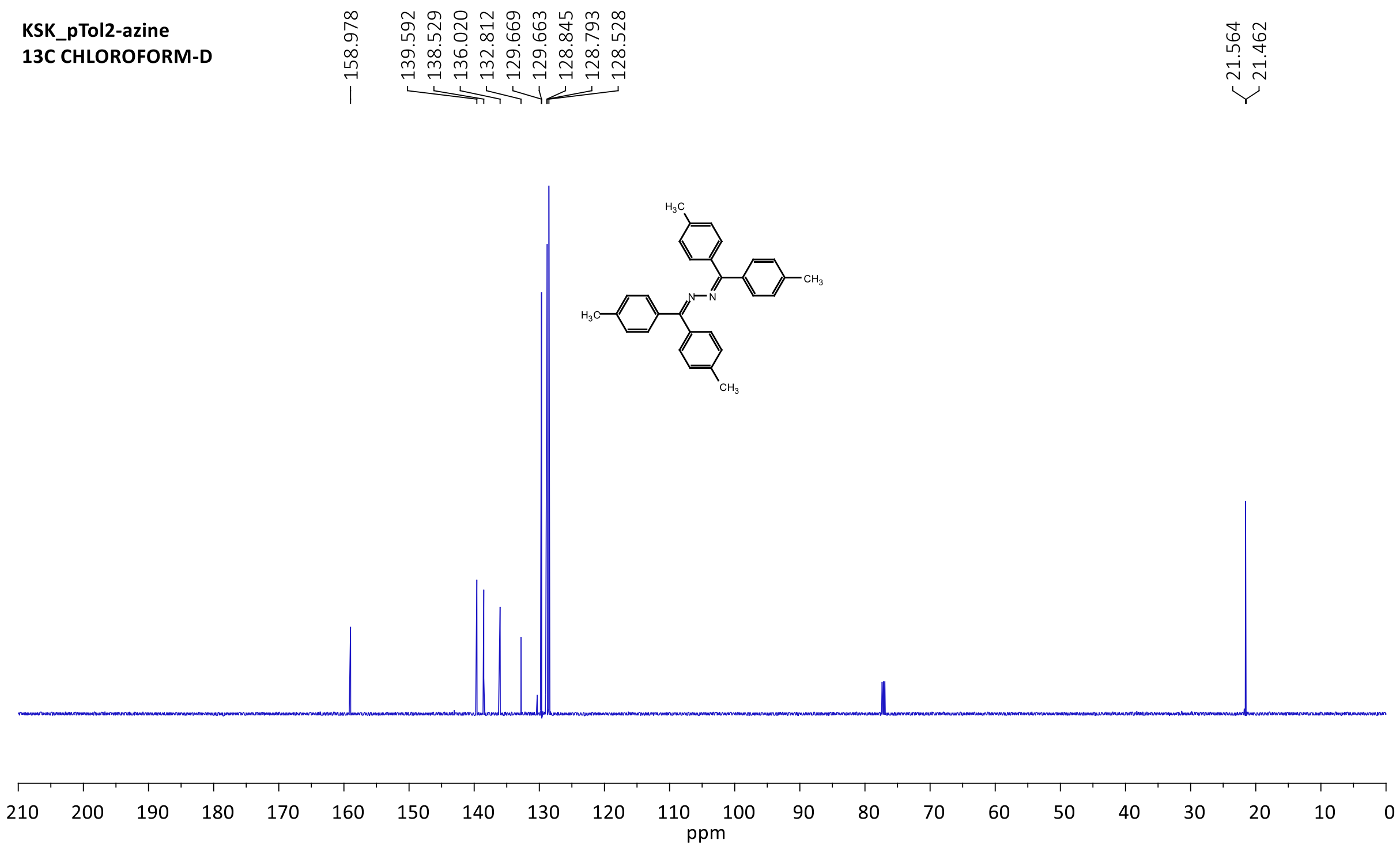

Origin	JEOL
Solvent	CHLOROFORM-D
Number of Scans	190
Spectrometer Frequency	150.91
Spectral Width	47348.5
Acquired Size	65536

^{13}C{^{1}H} NMR for 1,2-bis(di-4-tolylmethylene)hydrazine.

HYDROSILYLATION OF ACETOPHENONE

The typical NMR experiment was done preparing the sample in 0.5 mL of C_6D_6, in a J. Young NMR tube inside a glovebox, keeping the acetophenone concentration at 0.33 M and triethylsilane at about 0.67 M. The NMR yield was calculated from the representative ^{1}H NMR signals of the triethyl(1-phenyl-ethoxy)silane against the starting material after 1 h. Representative example of reaction progress for a weaker catalyst in this reaction (***1c***) over prolonged time period is presented below.

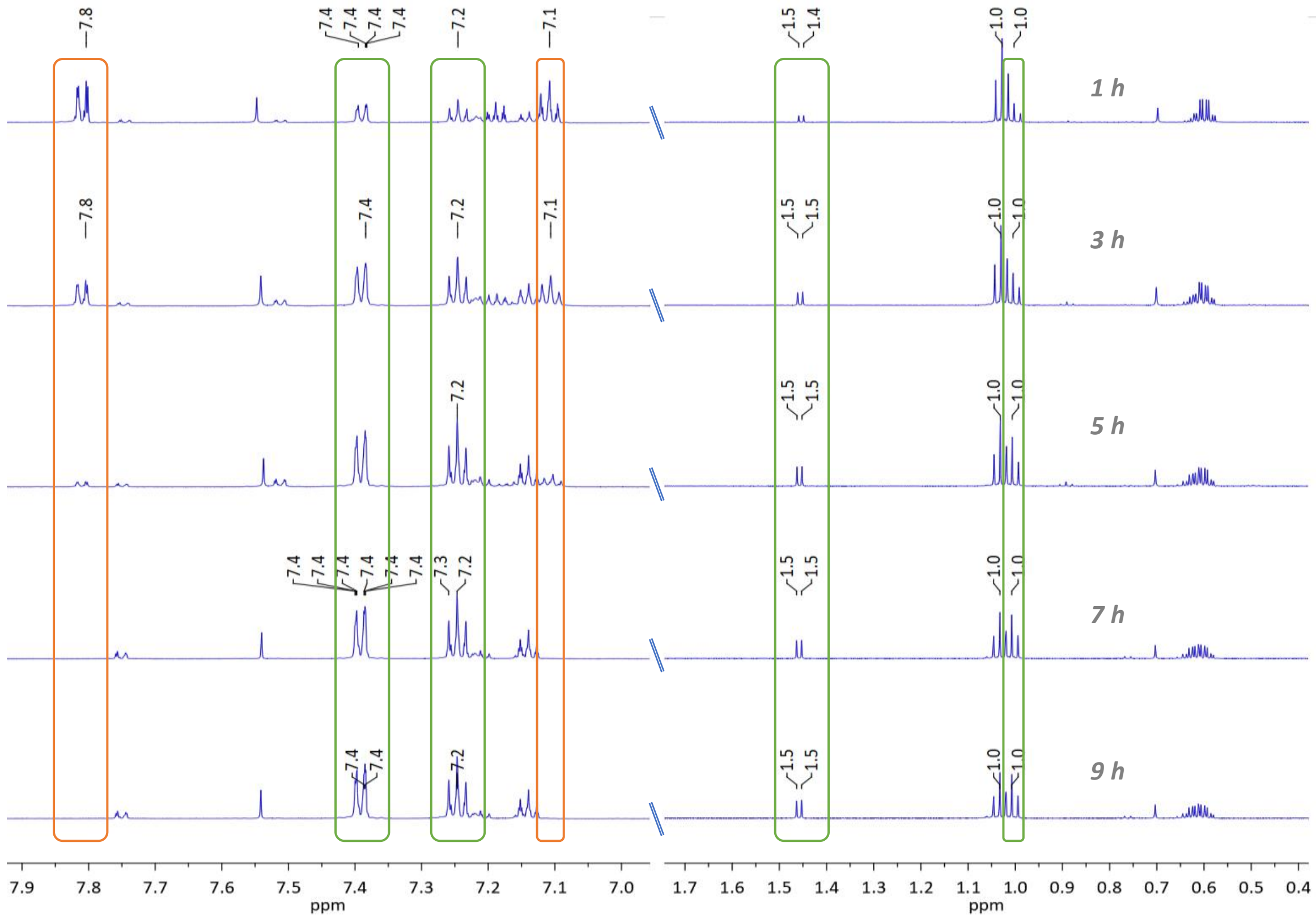

Figure S1: Hydrosilylation of acetophenone catalyzed by ***1c*** over 9 h monitored by ^{1}H NMR in C_6D_6. Representative signals in orange brackets correspond to the starting material, whereas those in green brackets correspond to the triethyl(1-phenylethoxy)-silane product.

The 1-phenylethanol product was obtained in 1.5 mmol scale reaction according to the following procedure.

Preparation of *1-phenylethanol*: 197 mg (1.637 mmol, 1 equiv.) of acetophenone was dissolved in 10 mL of dry toluene in a Schlenk vessel inside a glovebox. 381 mg (3.274 mmol, 2 equiv.) of triethylsilane were added, followed by 131 mg (0.164 mmol, 0.10 equiv.) of ***1d***. The reaction mixture was stirred for 4h at room temperature. The Schlenk flask was taken outside of the glovebox and 10 mL of 1 M HCl (aq.). solution was added slowly. The reaction mixture was emulsified by sonication for 10 min, then stirred for 18 h at room temperature and sonicated for 10 min again. Extraction with ethyl acetate (3 x 20 mL) and flash column chromatography (hexane/ethyl acetate) gave 183 mg of the alcohol product (92% yield).

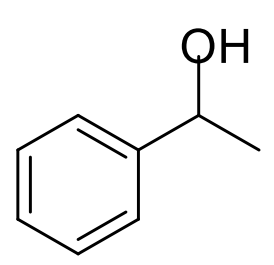

^{1}H NMR (600 MHz) δ/ppm (chloroform-d): 7.39 - 7.33 (m, 4H, Ar), 7.31 - 7.26 (m, 1H, Ar), 4.86 (q, $^3J_{HH}$ = 6.5 Hz, CH), 2.50 (br s, 1H, OH), 1.48 (d, $^3J_{HH}$ = 6.5 Hz, CH_3).

$^{13}C\{^1H\}$ NMR (151 MHz) δ/ppm: (chloroform-d): 145.9 (*C*H-Ar), 128.4 (*C*H-Ar), 127.3 (*C*H-Ar), 125.4 (*C*H-Ar), 70.2 (*C*H), 25.1 (*C*H_3).

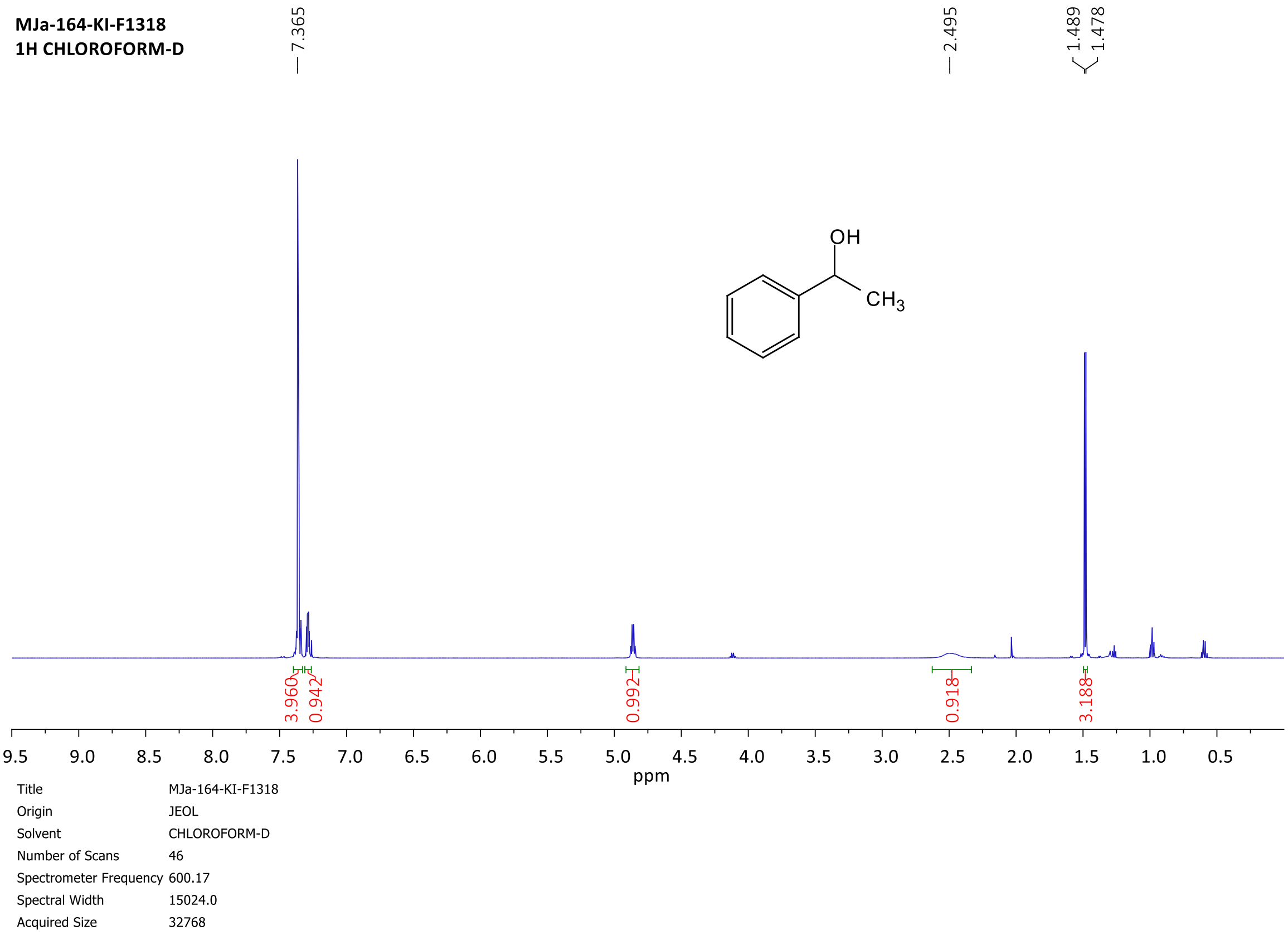

^{1}H NMR for 1-phenylethanol.

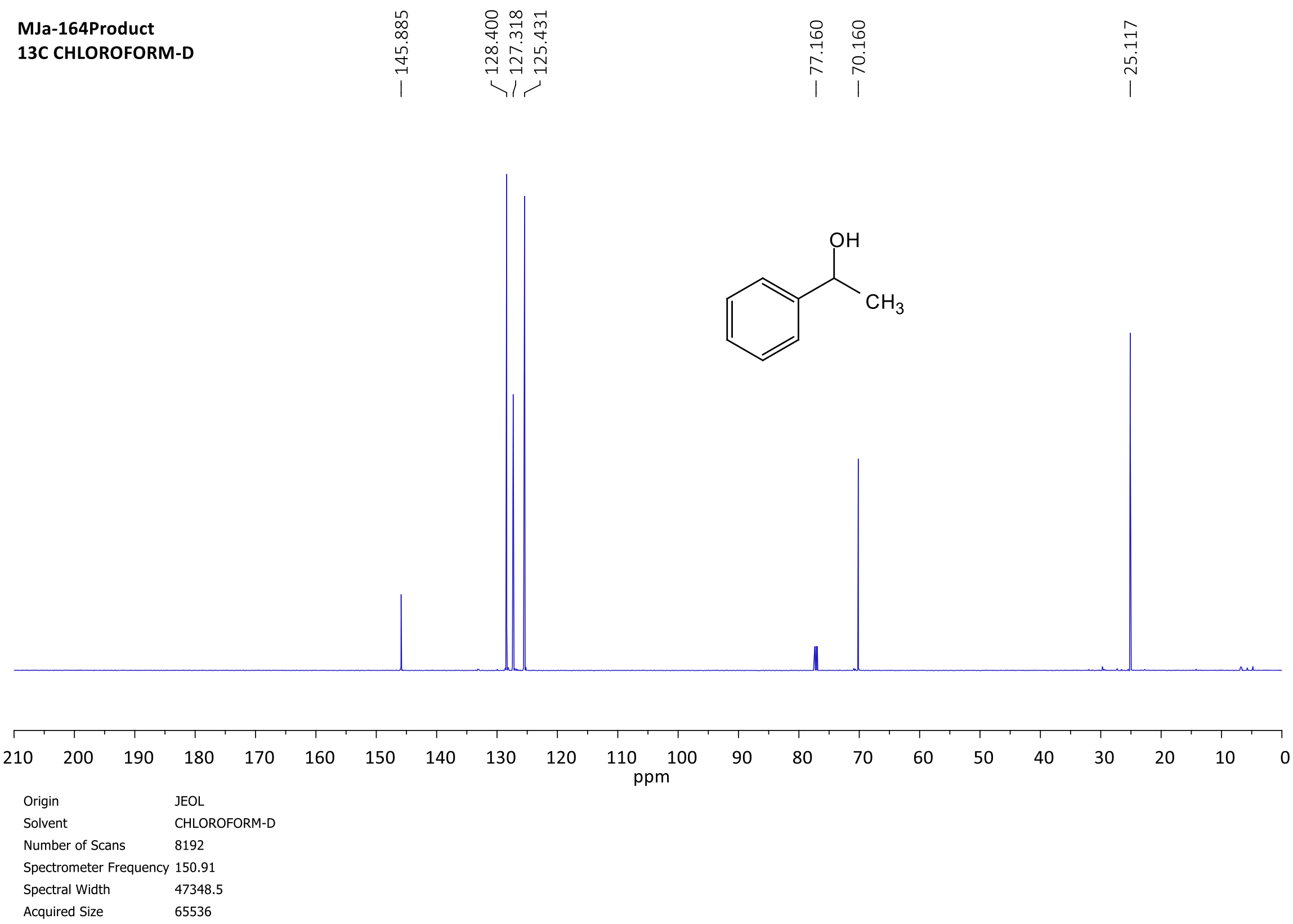

$^{13}C\{^{1}H\}$ NMR for 1-phenylethanol.

^{1}H NMR of reactions involving 4-chloroacetophenone, 4-bromoacetophenone, phenyl-*tert*-butyl ketone and phenyl-cyclohexyl ketone are presented below.

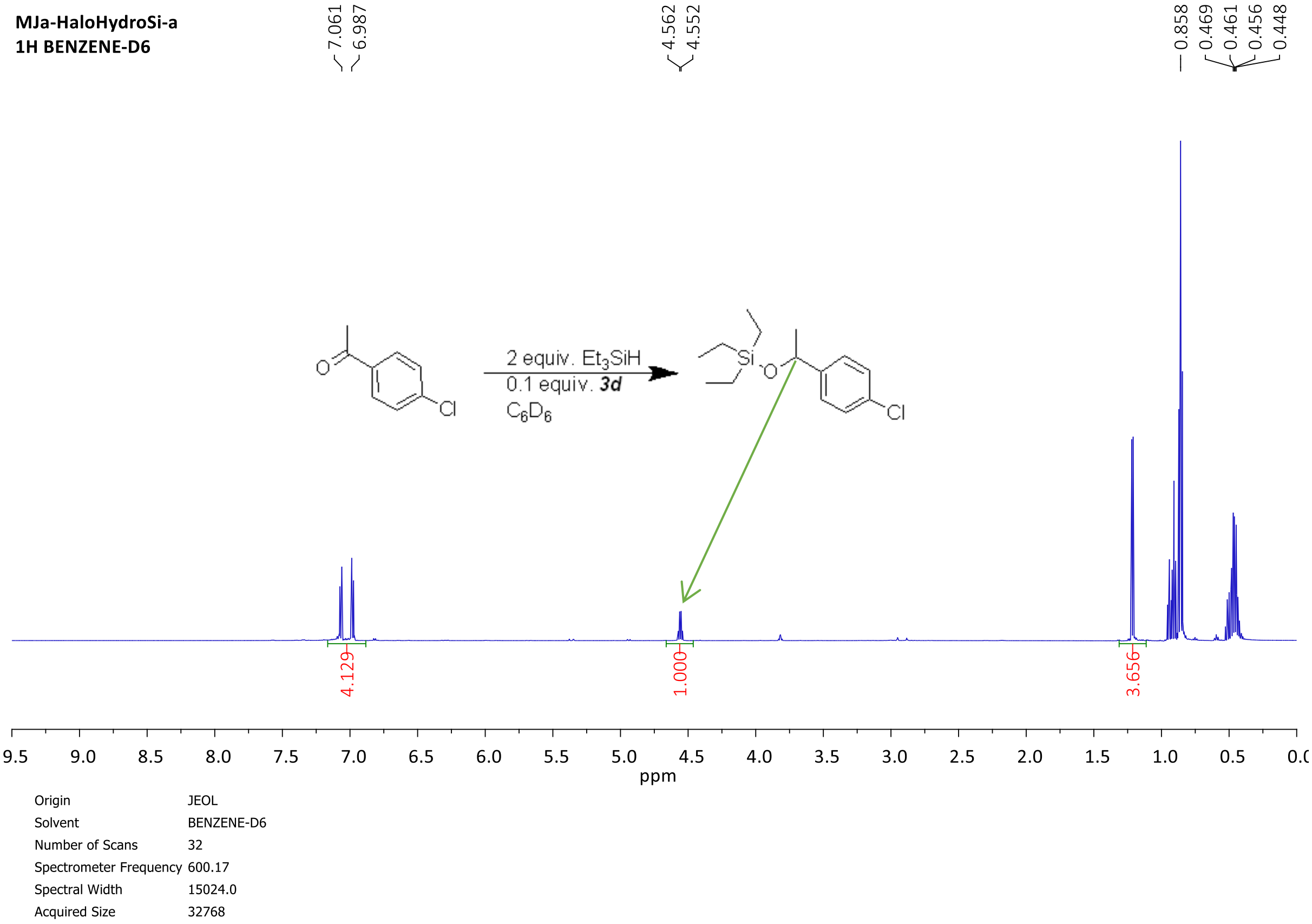

Figure S2: Hydrosilylation of 4-chloroacetophenone catalyzed by ***3d*** after 1 h monitored by ^{1}H NMR in C_6D_6.

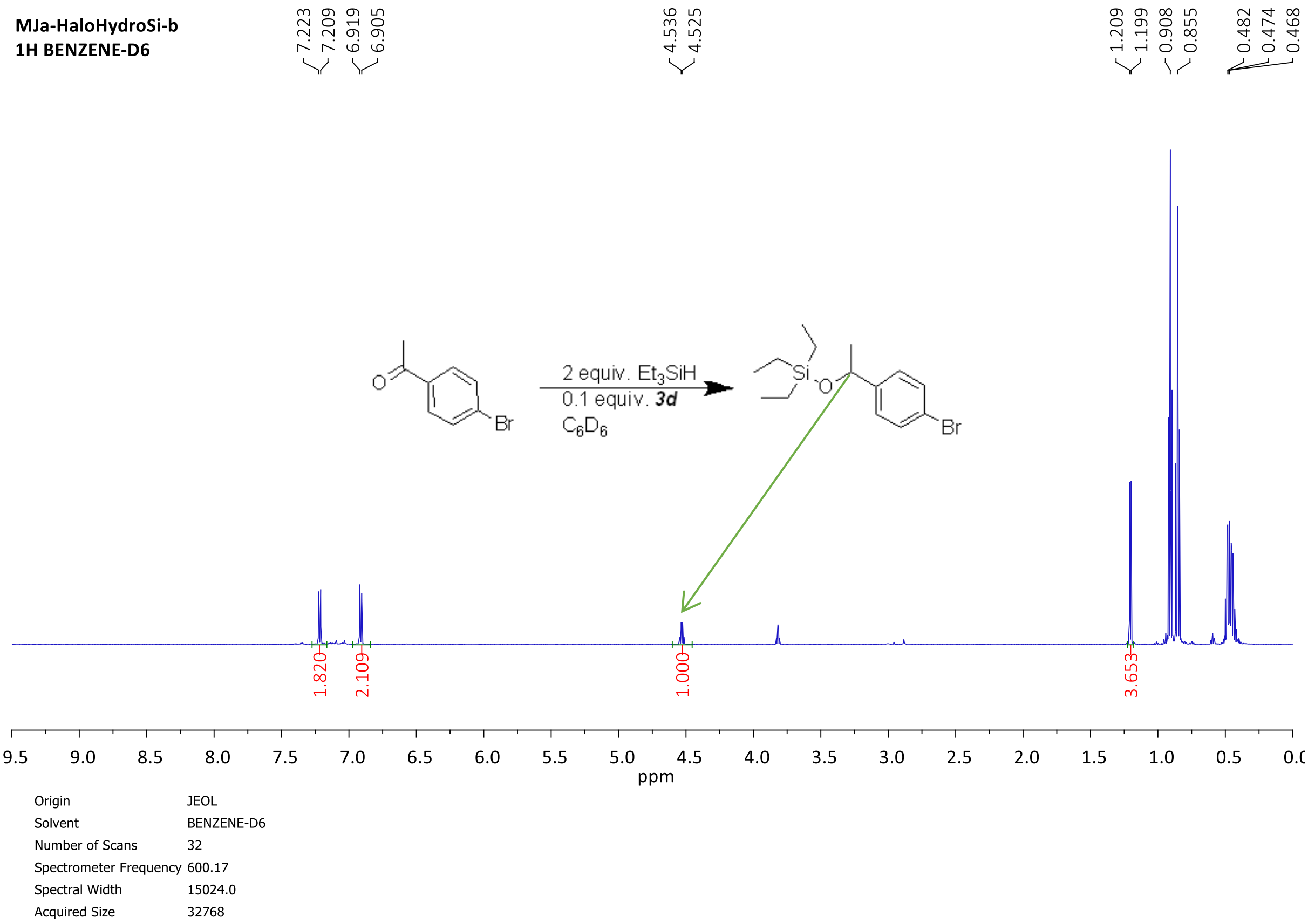

Figure S3: Hydrosilylation of 4-bromoacetophenone catalyzed by ***3d*** after 1 h monitored by ^{1}H NMR in C_6D_6.

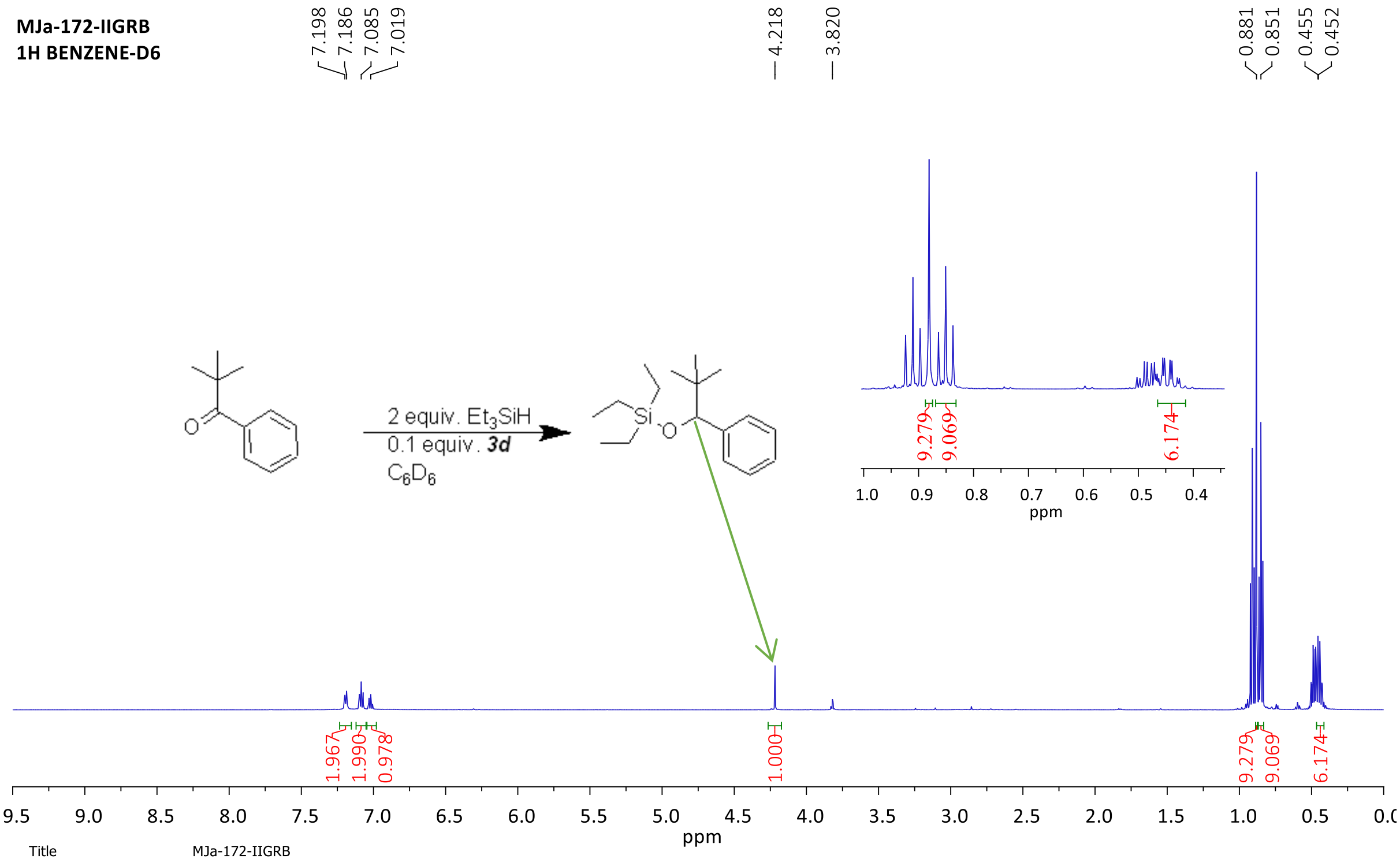

Title	MJa-172-IIGRB
Origin	JEOL
Solvent	BENZENE-D6
Number of Scans	64
Spectrometer Frequency	600.17
Spectral Width	15024.0
Acquired Size	32768

Figure S4: Hydrosilylation of phenyl-tert-butyl ketone catalyzed by ***3d*** after 1 h monitored by ^{1}H NMR in C_6D_6.

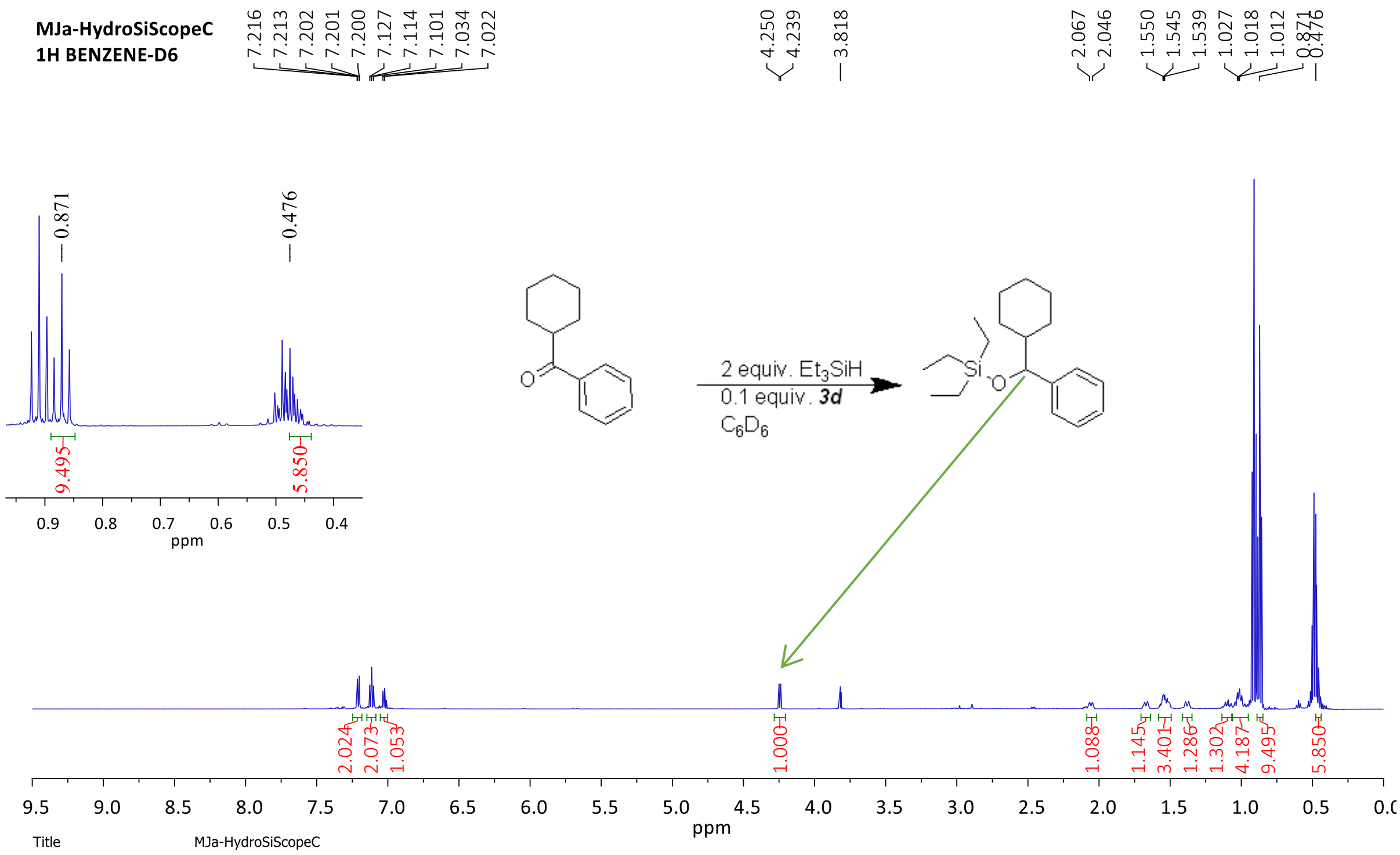

Title	MJa-HydroSiScopeC
Origin	JEOL
Solvent	BENZENE-D6
Number of Scans	59
Spectrometer Frequency	600.17
Spectral Width	15024.0
Acquired Size	32768

Figure S5: Hydrosilylation of phenyl-cyclohexyl ketone catalyzed by ***3d*** after 1 h monitored by ^{1}H NMR in C_6D_6.

In order to elucidate the reaction pathway, about 0.025-0.035 mmol of the representative catalysts ***1b***, ***1g***, ***3e*** (all hydrosilylation non-active) and ***1d***, ***3d*** (hydrosilylation active) were combined separately with excess amounts of acetophenone and Et_3SiH in DCM-d_2. Based on the ^{11}B NMR spectra recorded for acetophenone mixtures, the formation of a tetrahedral boron adduct is evident only in the case of ***3e*** (δ_B = –9.4 ppm) (**Fig. S6.**), which is reflected in ^1H NMR spectra (**Fig. S7**). In the remaining cases, the FWHM broadening of the δ_B signal by approximately 50-100 Hz (relative to pure substance) suggests that adduct formation occurs via a rapid equilibrium, strongly shifted toward the free components, indicating a very weak interaction with acetophenone and, consequently, a low adduct population.

Analysis of Et_3SiH mixtures by ^{11}B NMR reveals adduct formation for ***1d*** (δ_B = –18.8 ppm) and ***3d*** (δ_B = –15.0 ppm) (**Fig. S8**). However, in the case of ***1d***, the adduct population appears to be quite small (after 30 min.). In case of ***1b***, the FWHM of the δ_B signal is broadened (by 900 Hz), which also suggests a weak interaction resulting in an equilibrium shifted towards the free borenium. This is not the case for ***1g*** (no substantial FWHM δ_B signal broadening).

Furthermore, in the case of ***3e***, there is an indication of compound degradation as a result of B-O bond hydrosilylation. ^{11}B NMR spectra (**Fig. S11**) reveal signals matching $IMe_2BH_2^+$ NTf_2^- (tripletic δ_B = –18.0 ppm), and possibly a new trigonal borenium species (wide signal at δ_B = 20.5 ppm), a hydrosilylated intermediate. Trace IMe_2BH_3 is also visible (δ_B = -38.1 ppm). An additional minor, triplet signal δ_B = –22.6 ppm could not be identified with certainty. Presence of these species in ***3e*** mixture seems to be confirmed by both: ^1H NMR (**Fig. S9** (**A**)) (two singlet signals δ_H = 3.9 ppm and δ_H = 3.0 ppm corresponding to two types of N-CH_3 groups), as well as by ^{19}F NMR (**Fig. S10**), as presence of the $NTf_2^–$ anion existing in two different environments is confirmed by signals δ_F = –79.2 ppm (free isolated anion) and δ_F = –73.9 ppm (in contact with BH_2^+ cation species). However, we were unable to elucidate the structure of any new borenium species, and attempts to isolate them proved unsuccessful, as well as identification by HRMS analysis of the sample. Degradation of ***3e*** is slowly progressing at room temperature, and the unidentified borenium signals are diminishing. To accelerate the process, the sample containing ***3e*** mixture with excess Et_3SiH was incubated at 30 °C for 30 min., after which only traces of the mentioned intermediates were present (**Fig. S9** (**B**)). At this point, the sample contains mostly the reduced $IMe_2BH_2^+$ NTf_2^- borenium salt, doubly silylated diol ((*R*)-2,2'-bis((triethylsilyl)oxy)-1,1'-binaphthalene) as byproduct, and remaining Et_3SiH. To confirm the presence of the final silylated diol byproduct, the sample was thoroughly evaporated and re-recorded in $CDCl_3$. **Fig. S9** (**C**) clearly shows a triplet signal at δ_H = 0.61 ppm (separated in $CDCl_3$ from the Et_3SiH methylene signal) and a corresponding doublet of quartets at δ_H = 0.41 ppm. Moreover, a pattern of signals in the aromatic region is matching the doubly silylated diol. Furthermore, the $^{13}C\{^1H\}$ NMR spectrum of this sample (**Fig. S9** (**D**)) unequivocally confirms the sample composition and the presence of (*R*)-2,2'-bis((triethylsilyl)oxy)-1,1'-binaphthalene, as the signals match the literature data.[8]

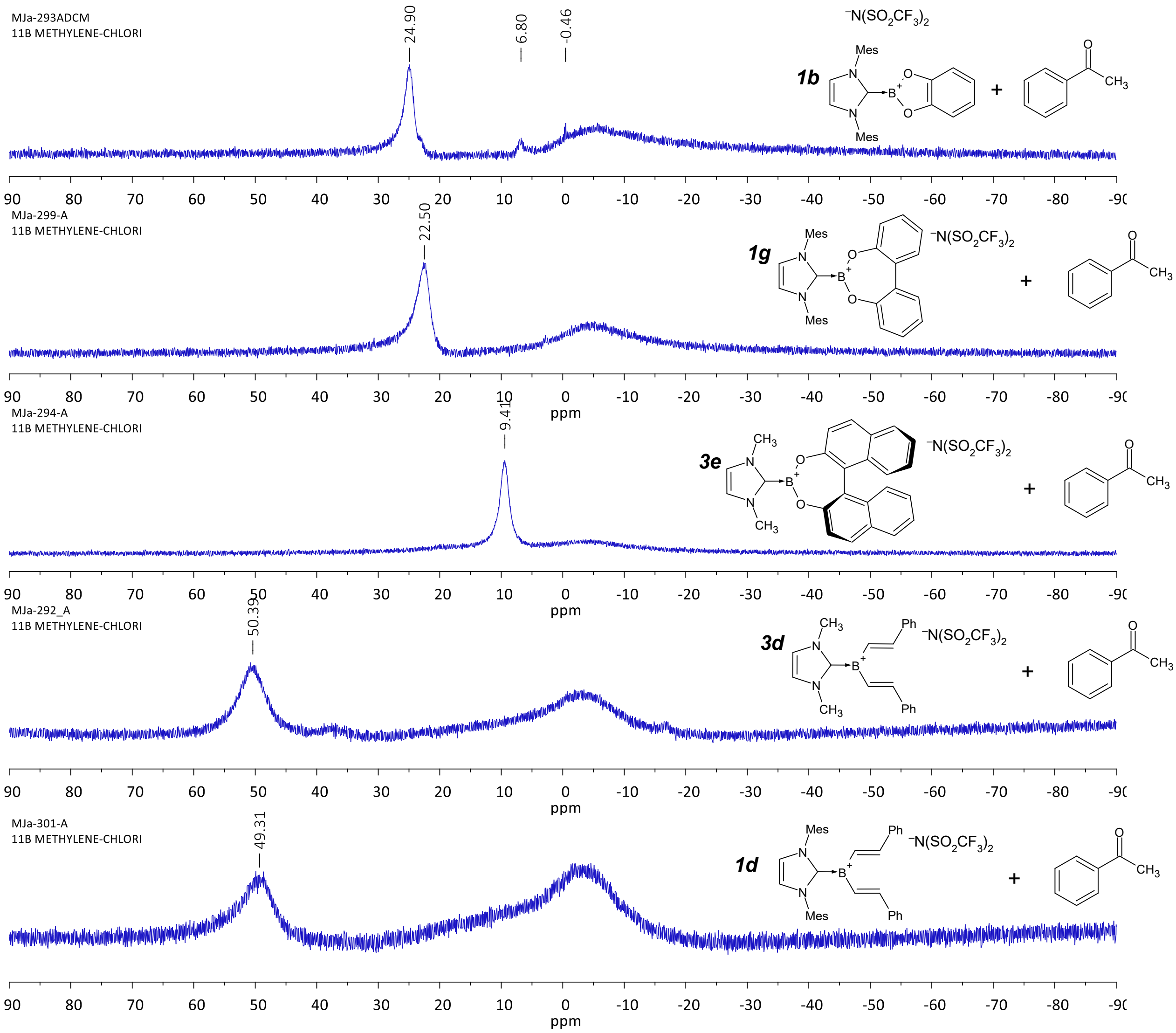

Figure S6: $^{11}B\{^{1}H\}$ NMR spectra for mixtures of ***1b***, ***1g***, ***3e***, ***1d*** and ***3d*** with acetophenone in DCM-d_2.

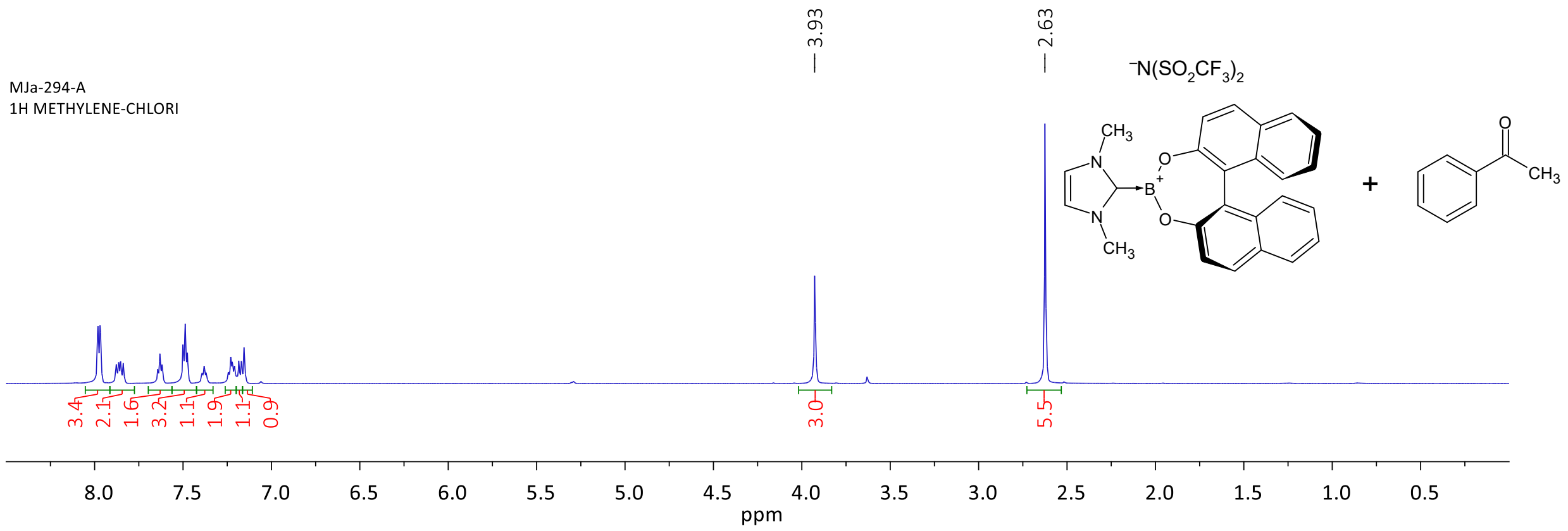

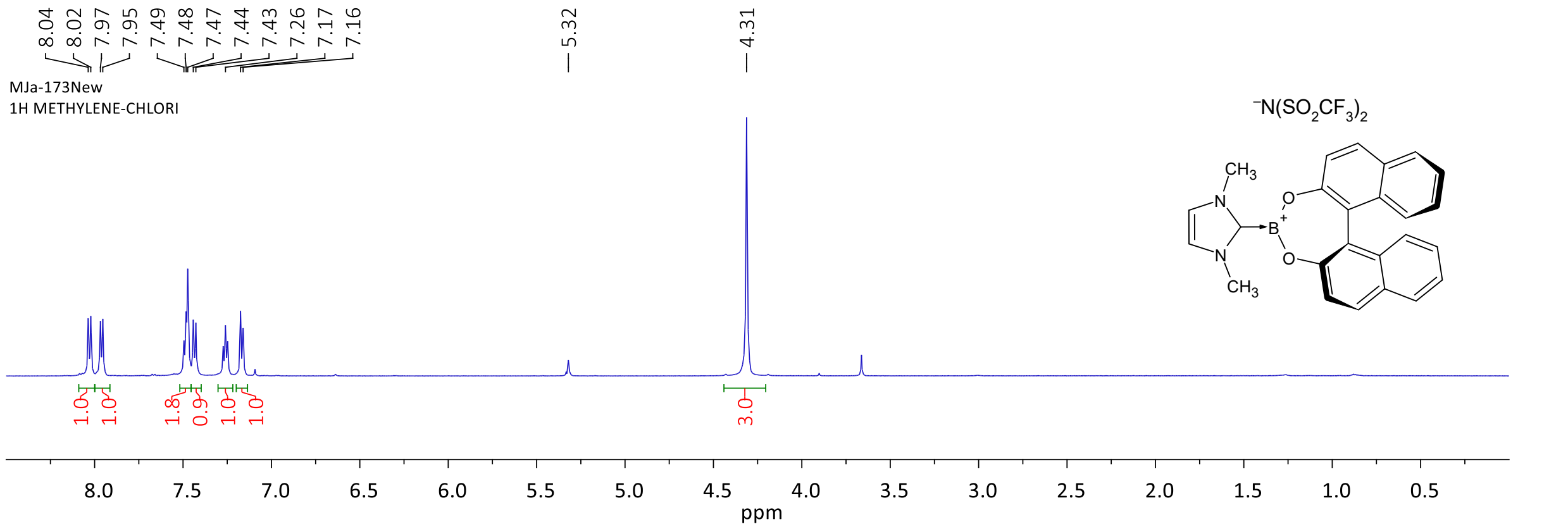

Figure S7: ^{1}H NMR spectra for mixture of ***3e*** with acetophenone and pure ***3e*** in DCM-d_2.

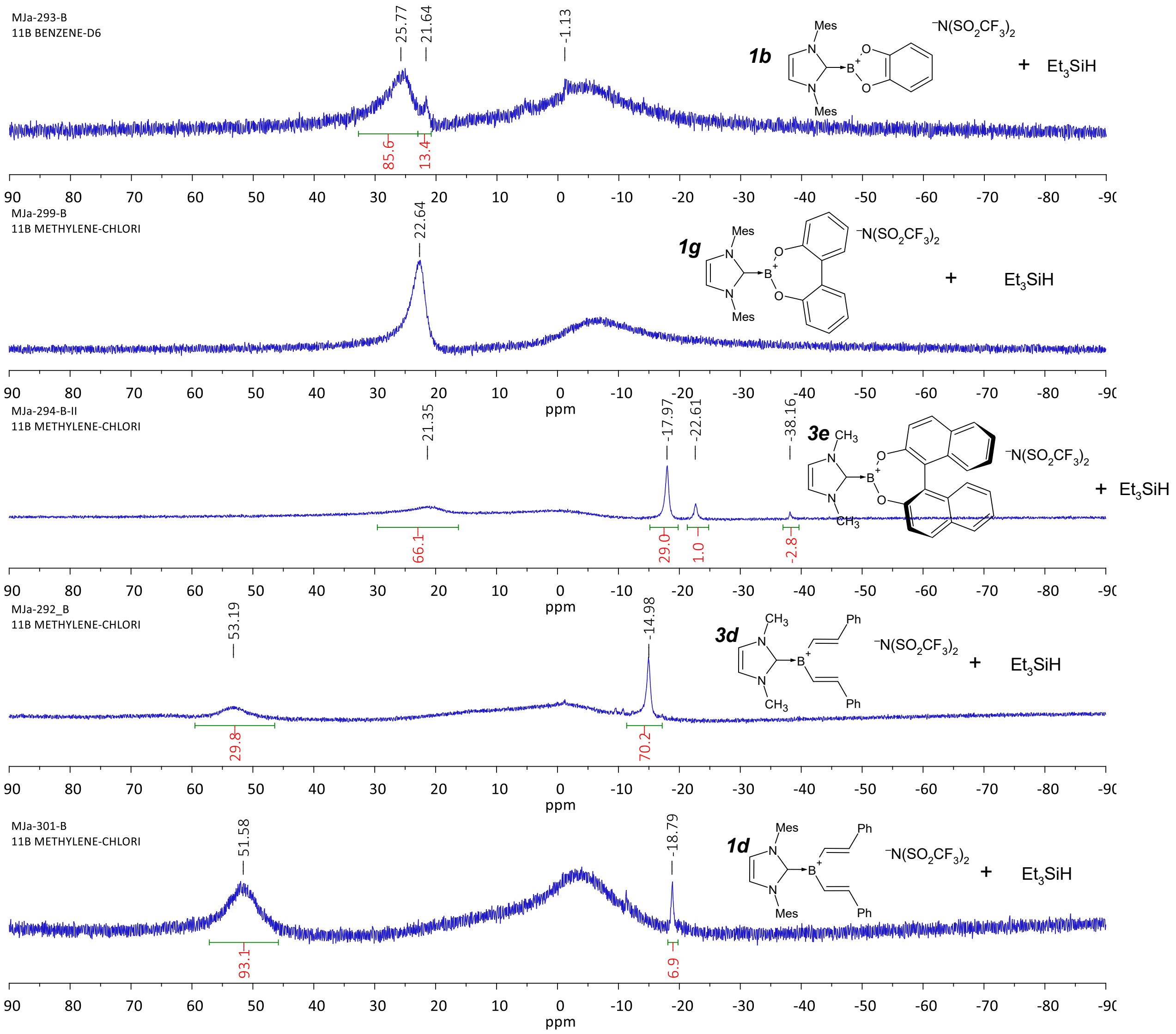

Figure S8: $^{11}B\{^1H\}$ NMR spectra for mixtures of ***1b***, ***1g***, ***3e***, ***1d*** and ***3d*** with Et_3SiH in DCM-d_2.

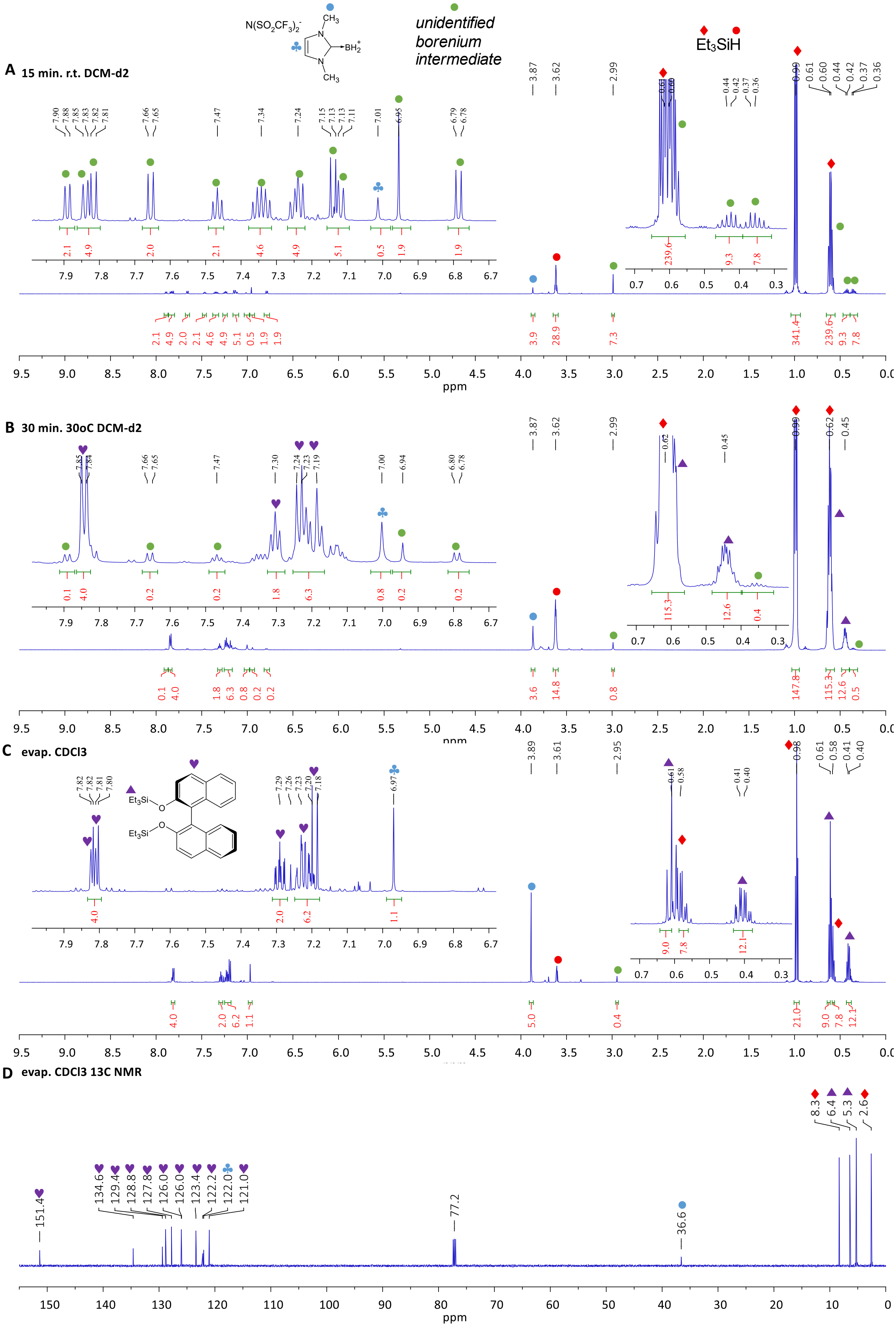

Figure S9: (**A**) ^{1}H NMR spectra for mixture of ***3e*** with excess Et_3SiH in DCM-d_2 after 15 min. at room temp., (**B**) the same sample after an additional 30 min. at 30 °C, (**C**) the same sample evaporated and re-recorded in $CDCl_3$, (**D**) $^{13}C\{^{1}H\}$ NMR of the sample in $CDCl_3$.

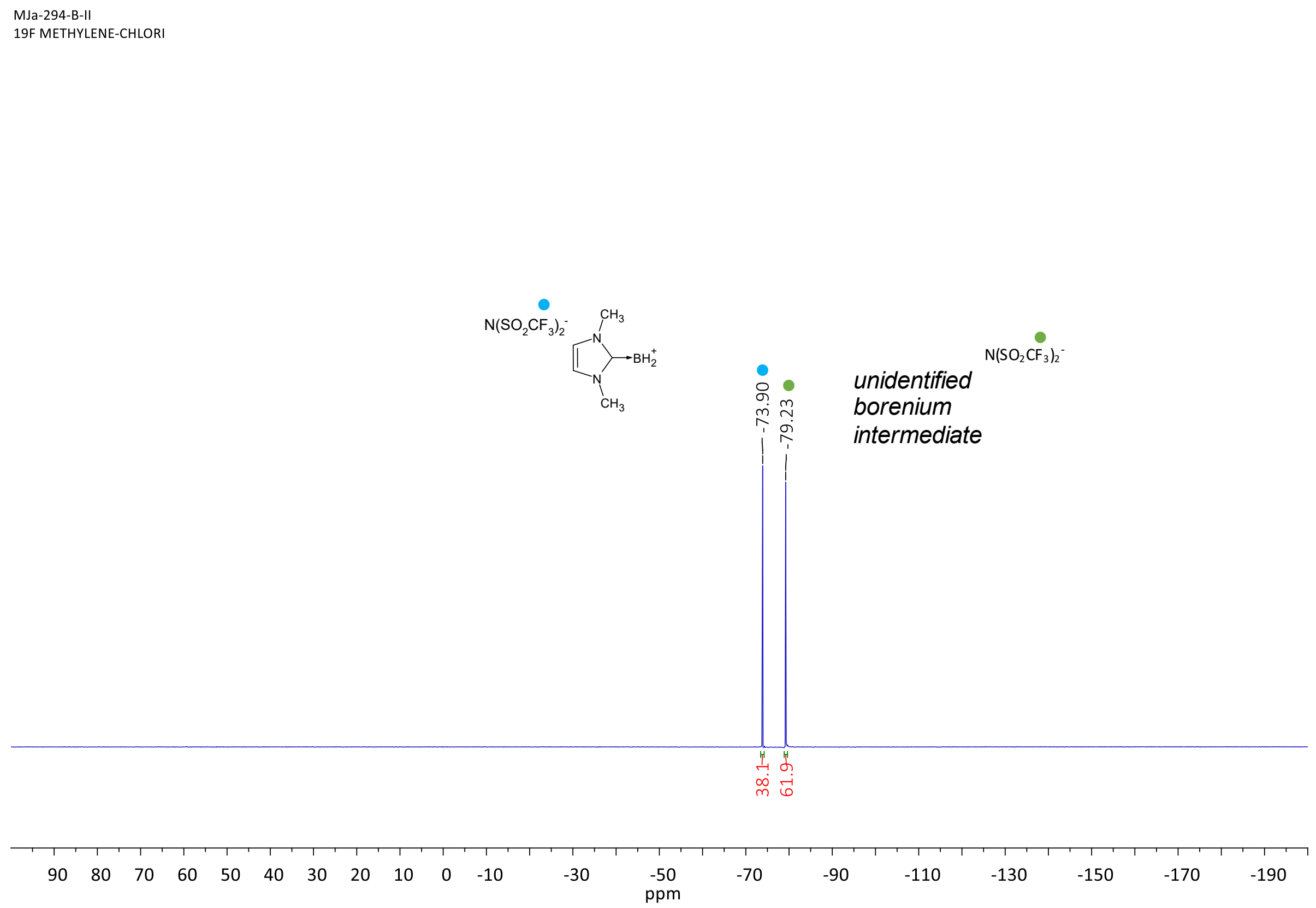

Figure S10: ^{19}F NMR spectra for mixture of ***3e*** with Et_3SiH in DCM-d_2 after 15 min. at room temp.

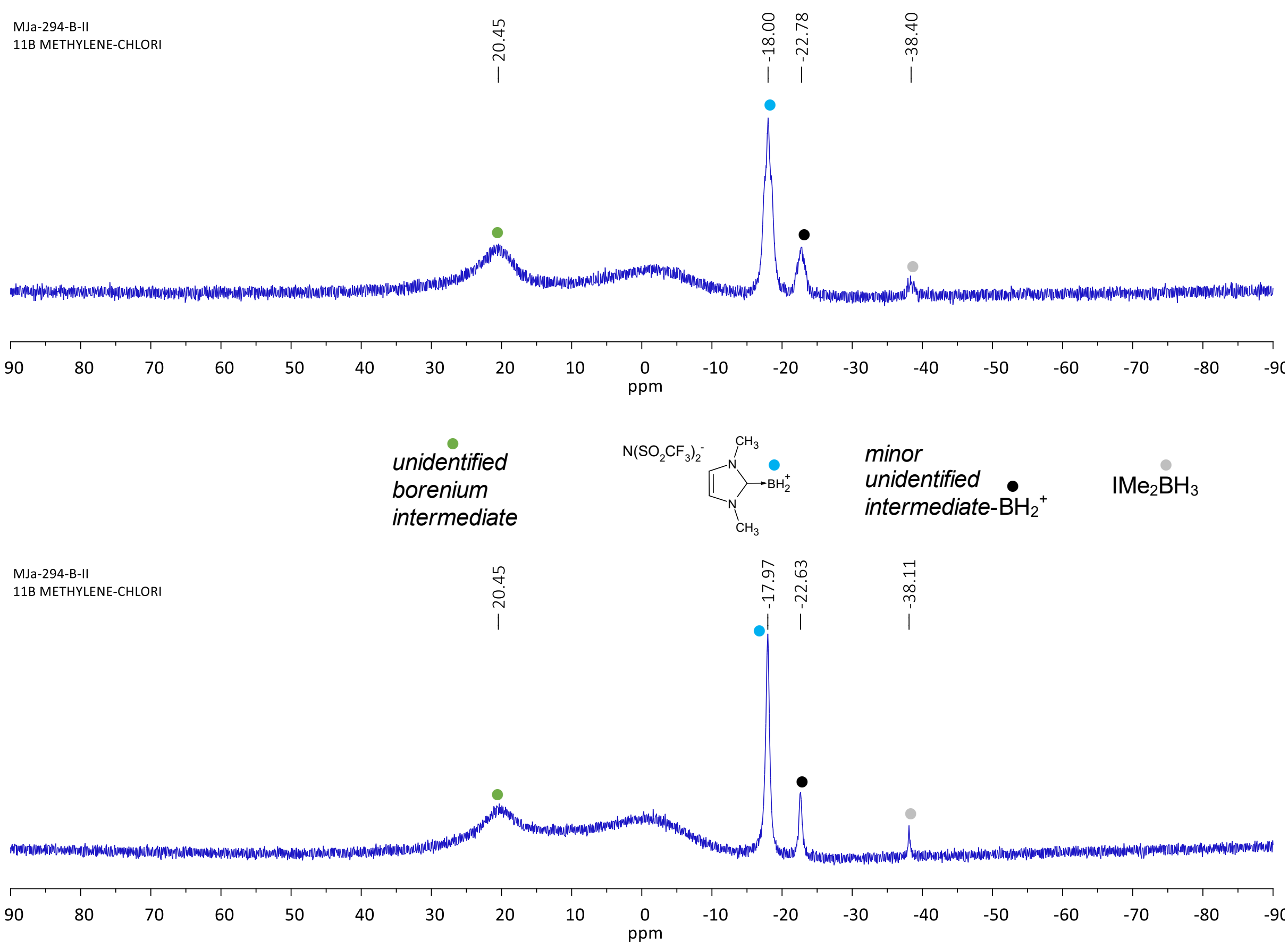

Figure S11: ^{11}B (top) and $^{11}B\{^1H\}$ (bottom) NMR spectra for mixture of ***3e*** with Et_3SiH in DCM-d_2 after 15 min. at room temp.

KINETIC MEASUREMENTS

Typical experiment description: Two oven-dried quartz cuvettes with air-tight screw-caps were transported into the glovebox. A 10.71 M stock solution of bis(4-methylphenyl)diazomethane in dry $CHCl_3$ has been prepared, and 2.8 mL was placed in the cuvette #1 via a syringe (final concentration of the starting material to be 0.01 M). A 0.754 mM stock solution of the catalyst under investigation was prepared, and about 2 mL were placed in a glass vial with a tight septum cap (final concentration in the cuvette to be 0.05 mM for 0.5 mol %). 2.8 mL of pure $CHCl_3$ were placed in the cuvette #2 (for the reference slot), followed by 0.2 mL of the catalyst stock solution. Both cuvettes and the glass vial were transported outside the glovebox. The UV-Vis spectrometer was autozeroed to air, and cuvette #1 was placed in the sample holder, placing cuvette #2 in the reference holder. 2.8 mL of the starting material solution was complemented with 0.2 mL of the catalyst stock solution in cuvette #1, followed by quick shaking, and the experiment was started promptly. Typical data collection was done by scanning the UV-Vis spectrum from 350 to 700 nm every 5 min. The reaction progress was recorded for about 2 h by tracking the consumption of the starting material. It was determined beforehand that bis(4-methylphenyl)diazomethane has an absorption maximum at 535 nm, and data for this wavelength was used to calculate the $k_{obs.}$ as the slope coefficient by fitting a linear regression line to the plot obtained for the first-order kinetics equation $\ln[A] = -kt + \ln[A]_0$. Data for all the investigated catalysts are shown in **Plot S1** and summarized in **Table S1** and in the main text. In some cases, an extremely weak drop in absorbance was indicative of very slow reaction progress even at higher catalyst loadings; therefore, compounds ***1a***, ***1d***, ***1g***, ***1g'***, ***1h***, ***3a***, and ***3c*** were excluded from the kinetic measurements. In other cases (catalysts comparably weak in this model reaction ***1c***, ***1f***), 1 mol % of the catalyst was applied to measure a reasonable signal change in a comparable time period. On the other hand, for the fastest catalysts in the series, the reaction progress at 0.05 mol % catalyst loading was too rapid to record the point of absorbance equal to 1. Much higher or lower loadings would render the results incomparable to each other, as the $k_{obs.}$ depends on the concentration.

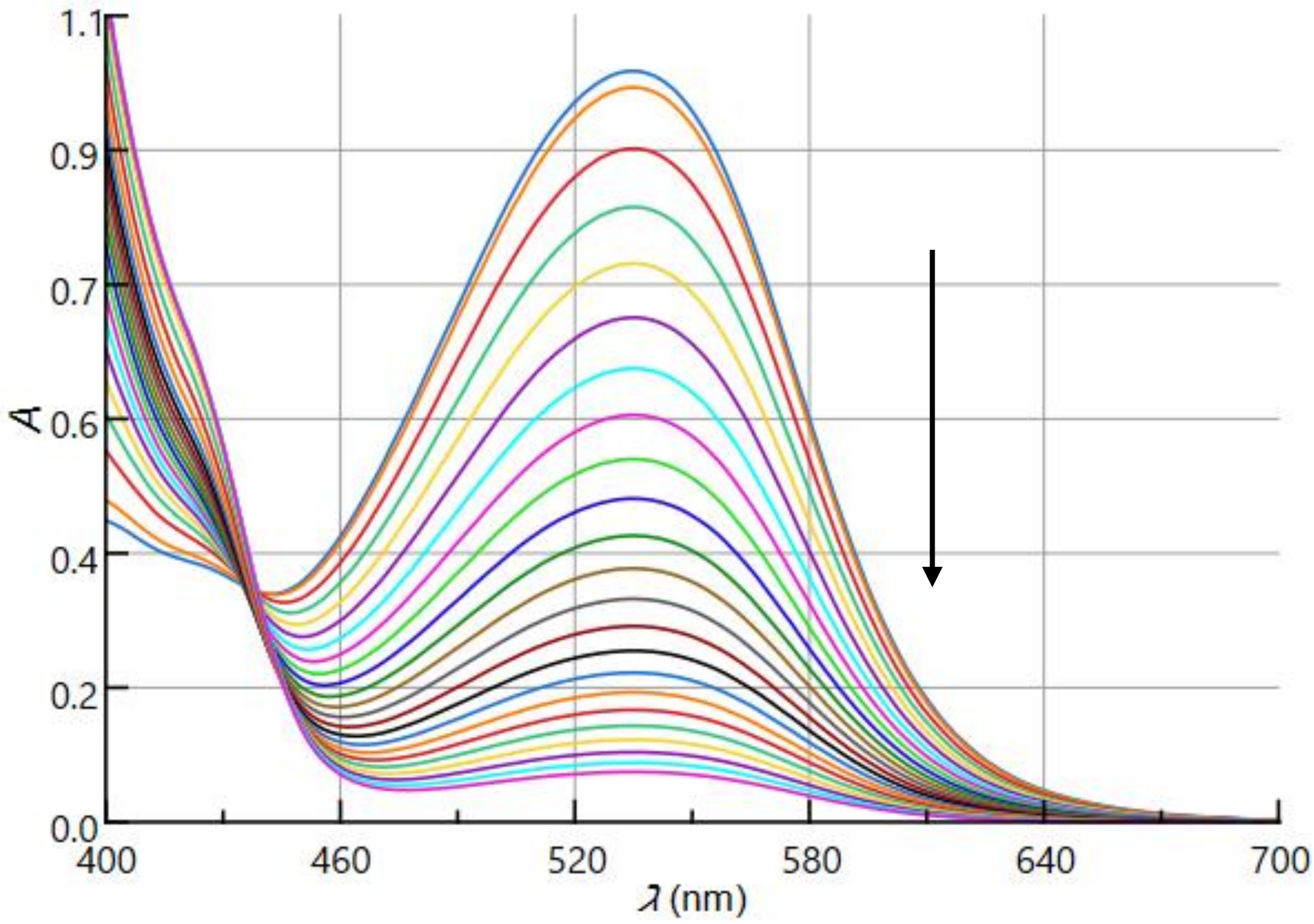

Plot S1: Example of a course of the homocoupling reaction (catalysed by ***1b'***) monitored by UV–Vis spectroscopy, showing the decrease in absorbance at 535 nm corresponding to the absorption maximum of bis(4-methylphenyl)diazomethane.

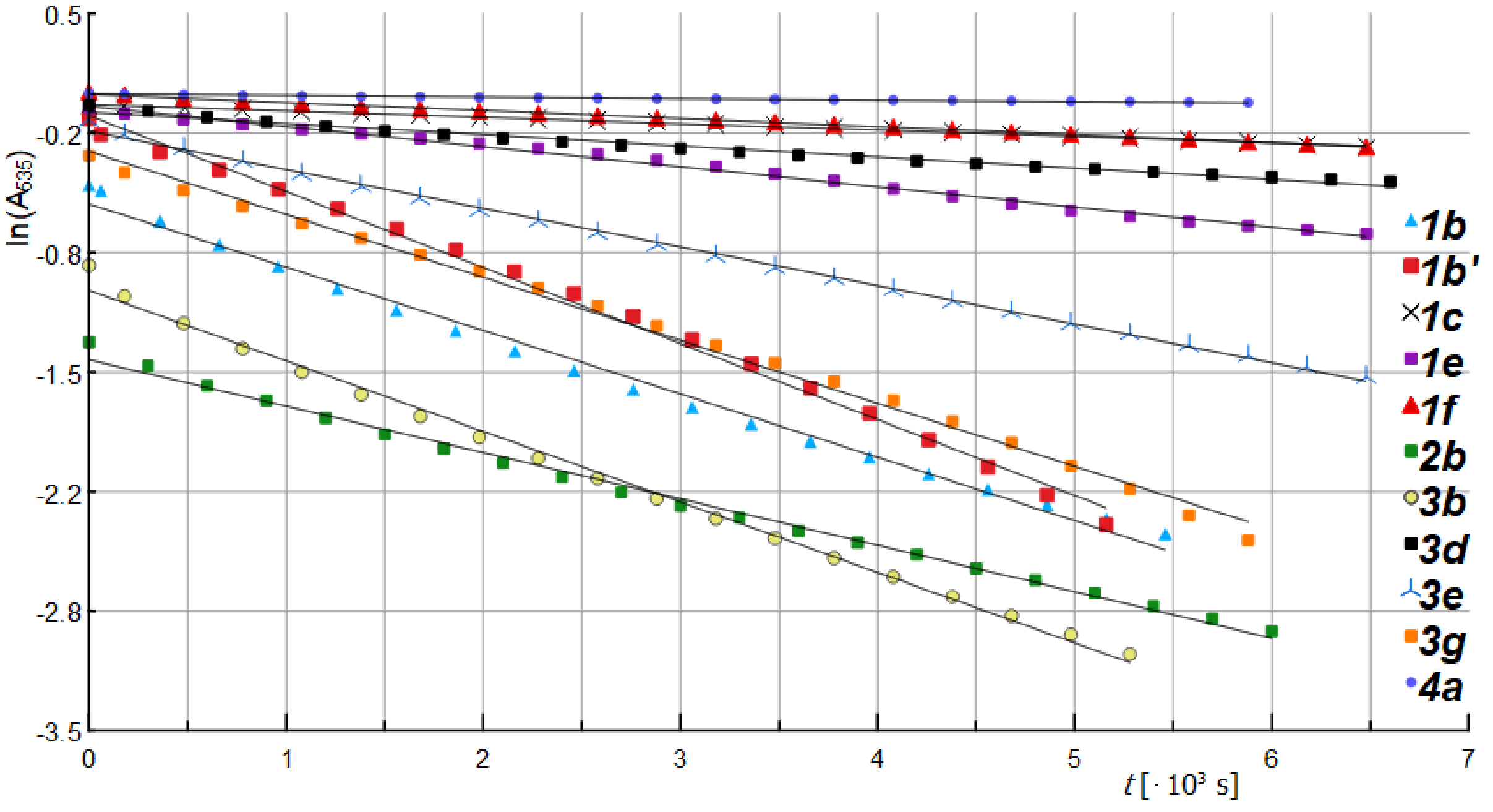

Plot S2: Logarithmic first-order kinetic equation fitted to the experimental logarithm of absorbance vs. time data for compounds ***1b***, ***1b'***, ***1c***, ***1e***, ***1f***, ***2b***, ***3b***, ***3d***, ***3e***, ***3g***, and ***4a***. Fitting parameters are summarized in **Table S1**.

Table S1: Diazo-homocoupling kinetic measurements.*

Entry	Catalyst	cat. / mol.%	$k_{obs.}$[a] / 10^{-5} s^{-1}	Δ $k_{obs.}$	R^2	product distribution / % [b]	
						ethen	azine
1	***1a***	-	-**	-	-	100	0
2	***1b***	0.5	**35.4**	± 0.7	0.992	100	0
3	***1b'***	0.5	**42.4**	± 0.7	0.996	70	30
4	***1c***	1.0	3.54	± 0.03	0.998	100	0
5	***1d***	-	-**	-	-	0	100
6	***1e***	0.5	**11.2**	± 0.2	0.995	100	0
7	***1f***	1.0	4.48	± 0.05	0.997	100	0
8	***1g***	-	-**	-	-	90	10
9	***1g'***	-	-**	-	-	100	0
10	***1h***	-	-**	-	-	100	0
11	***2b***	0.5	**25.9**	± 0.4	0.995	100	0
12	***3a***	-	-**	-	-	100	0
13	***3b***	0.5	**39.4**	± 0.6	0.995	0	100
14	***3c***	-	-**	-	-	100	0
15	***3d***	0.5	6.24	± 0.17	0.985	99	*trace*
16	***3e***	0.5	**21.6**	± 0.2	0.998	0	100
17	***3g***	0.5	**35.2**	± 0.5	0.996	5	95
18	***4a***[c]	0.5	0.821	± 0.025	0.983	100	0

*Conditions: [diazo] = 10 mM, [cat.] = 0.05~0.1 mM, T = 20 °C, t = 0–90 min. **Kinetics too slow to measure at comparable conditions. [a]First-order fitting, [b]NMR assessment after 24 h, [c]***4a*** = tris(pentafluorophenyl)-borane.

Assessment of the Lewis acidity by Gutmann method

The acceptor number (*AN*) of the title compounds was calculated from the measured ^{31}P NMR chemical shift of adducts with triethylphosphine oxide (TEPO) in DCM-d_2 at room temperature according the following equation:

$$AN = \frac{\delta_{\text{adduct}} - \delta_{\text{TEPO}_{\text{hexane}}}}{\delta_{\text{TEPO}_{\text{hexane}}} - \delta_{\text{TEPO}_{\text{SbCl}_5}}} \times 100$$

AN was originally proposed by Gutmann for the assessment of solvent properties.[9,10] The formation of a TEPO→LA adduct is reversible (which is clearly observed for weaker LAs), and the population of free TEPO can constitute a significant increment to the ^{31}P NMR signal, which is averaged between the free and adduct-engaged TEPO forms (the exchange rate is faster than the ^{31}P NMR acquisition time at room temperature). Samples of solid compounds must be prepared in a solvent characterized by a low *AN*. Measurements at a 1:1 ratio (TEPO:LA) might lead to an underestimated *AN* value for weaker LAs, and NMR titration must be performed to assess the accurate *AN*.[11] However, for strong LAs the titration procedure can be neglected.[12] Moreover, Jaekle *et al.* evidenced that TEPO interaction with neutral BCF (tris(pentafluorophenyl)borane, $B(C_6F_5)_3$) is stronger than with the borenium compound they obtained, despite the higher *AN* value for borenium (by about 6.1 ppm).[13] In a competition experiment, the population of TEPO→BCF adduct was larger.

In our case, the NMR samples were prepared in J. Young gas-tight NMR tubes in the glovebox at a 1:1 ratio, at 90 mM concentration in 0.5 mL of DCM-d_2. A single ^{31}P NMR signal was observed. For a few cases, the measurement was repeated for samples prepared at TEPO:LA ratio 1:2, to ensure that the measured ^{31}P NMR chemical shift fully corresponds to the TEPO engaged in the adduct with the investigated substance. However, in the case of compounds ***1f*** and ***1h***, the NMR titration was necessary, and *AN* was calculated from the extrapolated ^{31}P NMR chemical shift using equation $\delta(^{31}P) = (A \cdot x^B + C)/(D \cdot x^E + F)$, where $x = (^{n_{LA}}/_{n_{TEPO}})$ and the function limit in infinite LA molar excess over TEPO is $\delta(^{31}_{limit}P) = {}^{A}/_{D}$. The detailed procedure is described elsewhere.[11,12] For the mentioned two compounds, the ^{31}P NMR was measured for specific $^{n_{\mathrm{LA}}}/_{n_{\mathrm{TEPO}}}$ ratios, and the results are presented in **Plot S3**. It is clearly visible that for ***1f*** the measurement of *AN* at a 1:1 ratio leads to an underestimated value. In the case of ***1h***, the global Lewis acidity towards TEPO is very low, and the aforementioned error is negligible.

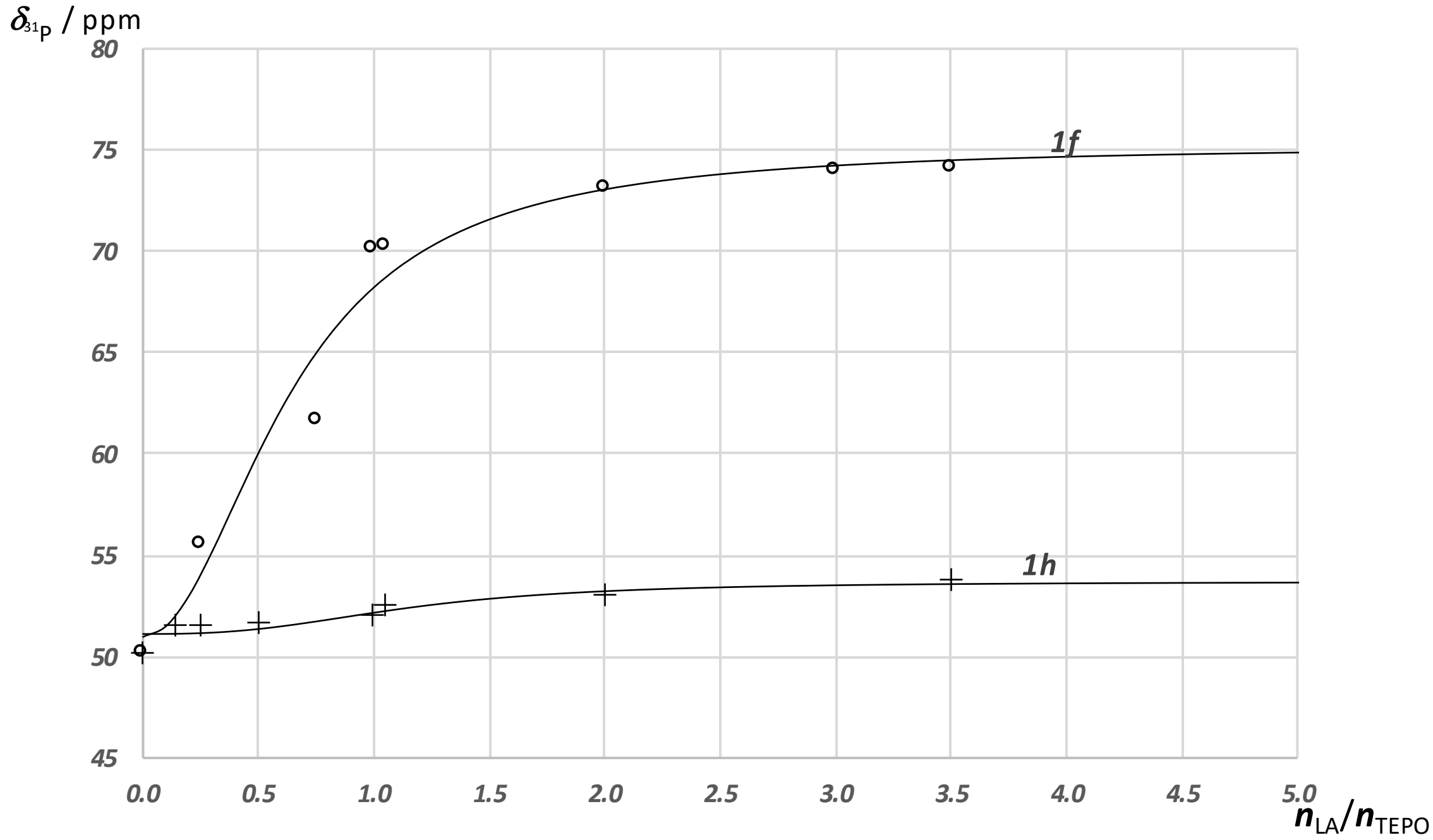

Plot S3: ^{31}P NMR titration as a function of x = $^{n_{LA}}/_{n_{TEPO}}$ molar ratio. The lines represent an irrational function $\delta(^{31}P) = (A \cdot x^B + C)/(D \cdot x^E + F)$, which parameters were numerically fitted using least squares method. For ***1f*** (data points as **o**) the parameters equal *A* = 8.0969, *B* = 2.0406, *C* = 2.2325, *D* = 0.1076, *E* = 2.0406, *F* = 0.0437 and function limit equals 75.2 ppm. For the very weak LA ***1h*** (data points as +) the parameters equal *A* = 0.8568, *B* = 2.7026, *C* = 1.1930, *D* = 0.0159, *E* = 2.7026, *F* = 0.0233 and function limit equals 53.7 ppm.

CRYSTAL STRUCTURE DATA

Suitable crystals for SC-XRD structure determination were obtained by diffusion of hexane into dichloromethane solutions of ***1b***, ***1b'***, ***1d***, ***1f***, ***2b***, ***3b***, ***3e*** and ***1h*****H** at room temperature inside the glovebox. The ORTEP images for all eight structures are included in **Figure S12 - 19**. Deposition Numbers in the **Table S2** below contain the supplementary crystallographic data for this paper. This data can be obtained free of charge *via* the joint Cambridge Crystallographic Data Centre (CCDC) and das Fachinformationszentrum Karlsruhe Access Structures Service.

The structure of compound ***3e*** (**Figure S18**) contains nine independent cation-anion pairs with varying bond lengths and angles. The average B–C_{NHC} bond length in the structure is 1.54 Å, which is the shortest of all the presented structures. The N3-C2-B1-O10 dihedral angle varies between 158.4° and 178.5°, however, there is no clear correlation between the angle and the B–C_{NHC} bond length for individual molecules in the structure. The precision of the values is compromised by the degradation of the crystal during measurement.

Table S2: Crystal data for all structures, data collection, and details of refinement.

Compound	*1f*	*1b*	*2b*	*3b*
CCDC No.	2499423	2499426	2499431	2499432
Emp. formula	$C_{62}H_{64}B_2F_{12}N_6O_{12}S_4$	$C_{29}H_{28}BF_6N_3O_6S_2$	$C_{60}H_{64}B_2Cl_4F_{12}N_6O_{12}S_4$	$C_{13}H_{12}BF_6N_3O_6S_2$
Formula weight	1463.05	703.47	1580.83	495.19
Crystal system	monoclinic	monoclinic	monoclinic	monoclinic
Space group	*P*2₁/*c*	*P*2₁/*n*	P2₁/c	C2/c
a / Å	12.5580(2)	8.80920(10)	19.2066(4)	9.4951(4)
b / Å	10.8009(2)	10.80100(10)	19.7534(3)	12.3004(5)
c / Å	25.1195(4)	33.0400(2)	21.2220(4)	16.6695(7)
α / °	90	90	90	90
β /°	103.131(2)	91.7900(10)	114.476(2)	101.500(4)
γ / °	90	90	90	90
Volume / Å^3	3318.07(10)	3142.16(5)	7328.0(3)	1907.80(14)
Z	2	4	4	4
Density / g cm^{-3}	1.464	1.487	1.433	1.724
M / mm^{-1}	2.184	2.284	3.331	3.457
F(000)	1512.0	1448.0	3248.0	1000.0
Crystal size / mm	0.23×0.19×0.09	0.305×0.245×0.14	0.22×0.18×0.1	0.22×0.12×0.06
2Θ range for data collection / °	7.228 to 153.048	5.352 to 160.282	6.4 to 154.358	10.832 to 133.136
Reflections collected	20084	79540	50200	9768
Independent reflections	6480 [R_{int} = 0.0278, R_{sigma} = 0.0264]	6846 [R_{int} = 0.0335, R_{sigma} = 0.0155]	14621 [R_{int} = 0.0370, R_{sigma} = 0.0359]	1692 [R_{int} = 0.0413, R_{sigma} = 0.0282]
Data / restraints / parameters	6480/0/448	6846/0/431	14621/0/1000	1692/93/186
Goodness-of-fit on F^2	1.059	1.041	1.068	1.063
R [*I*>=2σ(*I*)]	R_1 = 0.0378, wR_2 = 0.1029	R_1 = 0.0338, wR_2 = 0.0901	R_1 = 0.0653, wR_2 = 0.1864	R_1 = 0.0393, wR_2 = 0.0978
R_w (all data)	R_1 = 0.0400, wR_2 = 0.1046	R_1 = 0.0341, wR_2 = 0.0903	R_1 = 0.0739, wR_2 = 0.1951	R_1 = 0.0436, wR_2 = 0.1006
Largest diff. peak / hole e Å^{-3}	0.41/-0.43	0.34/-0.43	1.09/-1.31	0.55/-0.57

**Table S2**: …continued.				
**Compound**	***1b'***	***3e***	***1d***	***1h*H**
**CCDC No.**	2499434	2499437	2499440	2504323
**Emp. formula**	$C_{52}H_{30}B_2Cl_2F_{20}N_2O_2$	$C_{285}H_{278}B_9F_{54}N_{27}O_{54}S_{18}$	$C_{78}H_{76}B_2F_{12}N_6O_8S_4$	$C_{61}H_{85}B_2N_4$
**Formula weight**	1187.30	6645.70	1603.30	895.94
**Crystal system**	triclinic	monoclinic	monoclinic	monoclinic
**Space group**	P-1	$P2_1$	P2/c	$P2_1/n$
***a* / Å**	12.7208(5)	19.7316(7)	20.5067(5)	17.10980(19)
***b* / Å**	13.7574(6)	14.4170(5)	9.3136(2)	18.0279(2)
***c* / Å**	15.5159(6)	53.2452(19)	22.4590(6)	17.7494(2)
***α* / °**	70.483(4)	90	90	90
***β* /°**	80.618(3)	93.470(4)	113.725(3)	100.9658(11)
***γ* / °**	78.952(3)	90	90	90
**Volume / Å^3**	2497.49(19)	15118.9(9)	3926.95(18)	5374.90(11)
Z	2	2	2	4
**Density / g cm^{-3}**	1.579	1.460	1.356	1.107
**M / mm^{-1}**	2.256	2.164	1.857	0.470
**F(000)**	1192.0	6856.0	1664.0	1956.0
**Crystal size / mm**	0.32×0.22×0.03	0.42×0.3×0.21	0.37×0.21×0.15	0.658×0.132×0.097
**2Θ range for data collection / °**	6.078 to 162.252	4.486 to 152.694	4.706 to 157.84	6.578 to 151.768
**Reflections collected**	58242	170366	48241	33170
**Independent reflections**	10831 [R_{int} = 0.0642, R_{sigma} = 0.0422]	55325 [R_{int} = 0.1362, R_{sigma} = 0.0936]	8180 [R_{int} = 0.0637, R_{sigma} = 0.0382]	10502 [R_{int} = 0.0357, R_{sigma} = 0.0388]
**Data / restraints / parameters**	10831/72/738	55325/457/3653	8180/0/505	10502/0/628
**Goodness-of-fit on *F*^{2}**	1.076	1.086	1.100	1.061
***R* [*I*>=2σ(*I*)]**	R_1 = 0.0783, wR_2 = 0.2299	R_1 = 0.1095, wR_2 = 0.3011	R_1 = 0.0525, wR_2 = 0.1546	R_1 = 0.0491, wR_2 = 0.1349
**R_w (all data)**	R_1 = 0.0845, wR_2 = 0.2379	R_1 = 0.1681, wR_2 = 0.3512	R_1 = 0.0629, wR_2 = 0.1673	R_1 = 0.0597, wR_2 = 0.1441
**Largest diff. peak / hole e Å^{-3}**	1.36/-1.71	0.53/-0.42	0.39/-0.34	0.26/-0.20

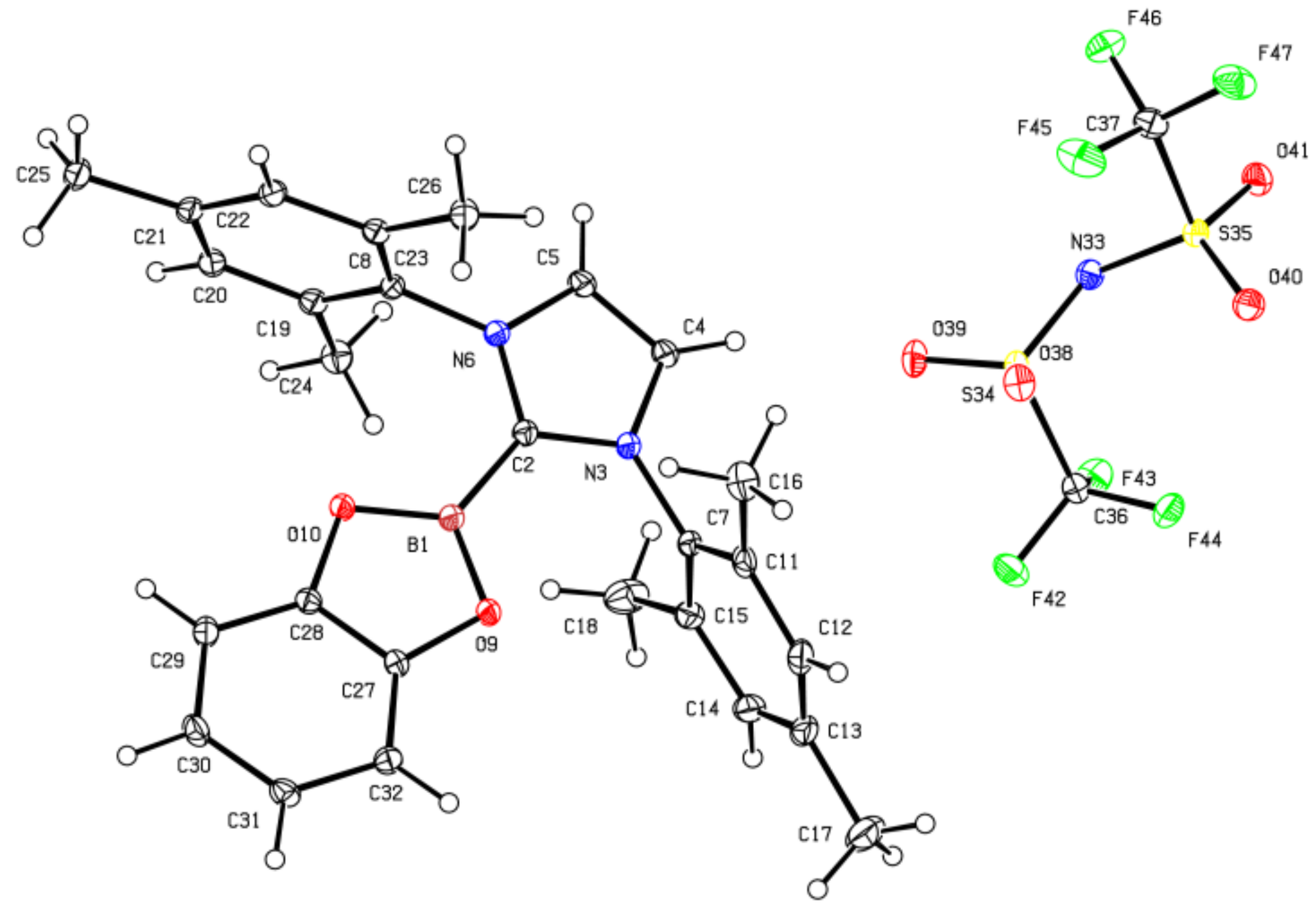

Figure S12: Molecular structure view of ***1b*** including the anion.

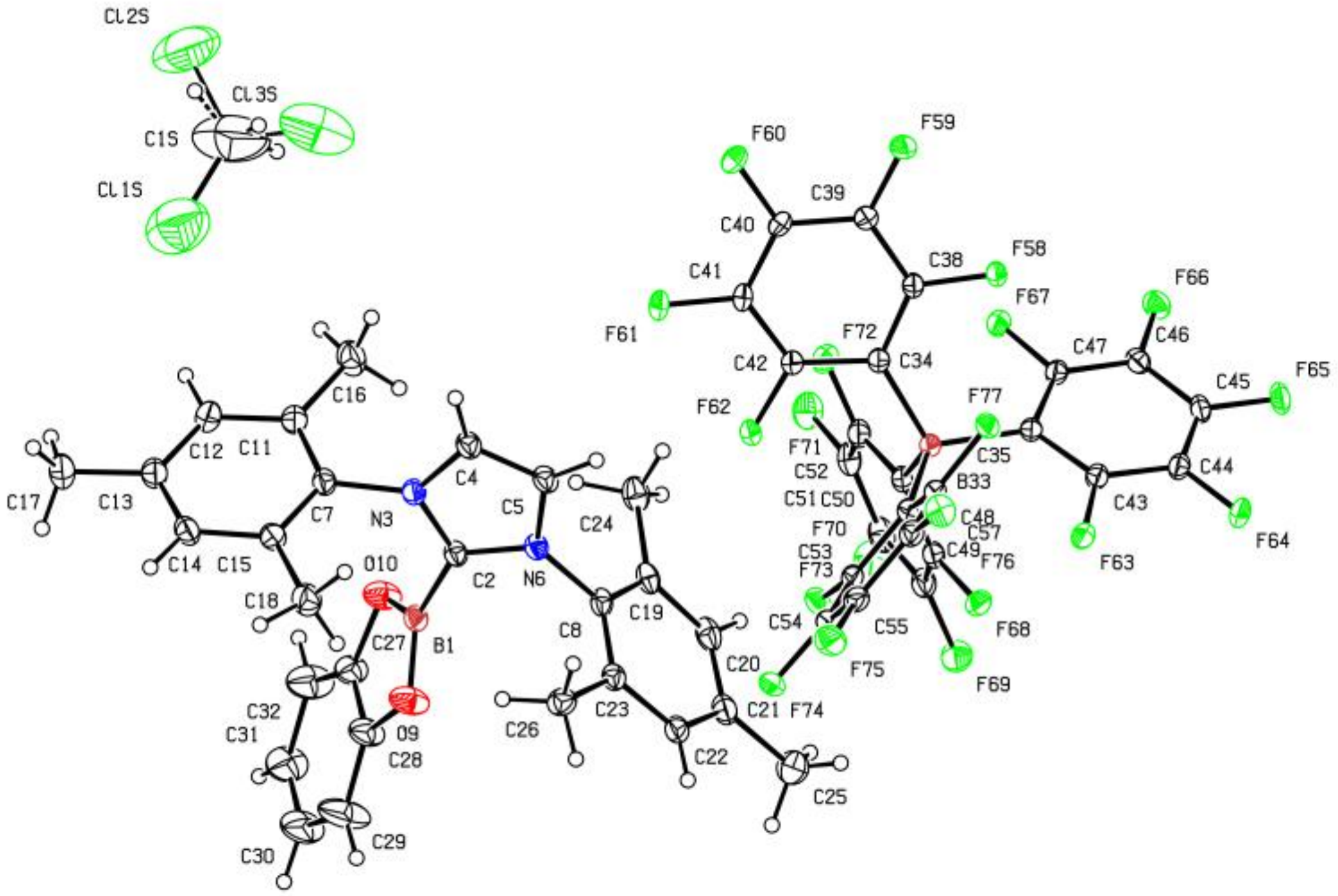

Figure S13: Molecular structure view of ***1b'*** including the anion.

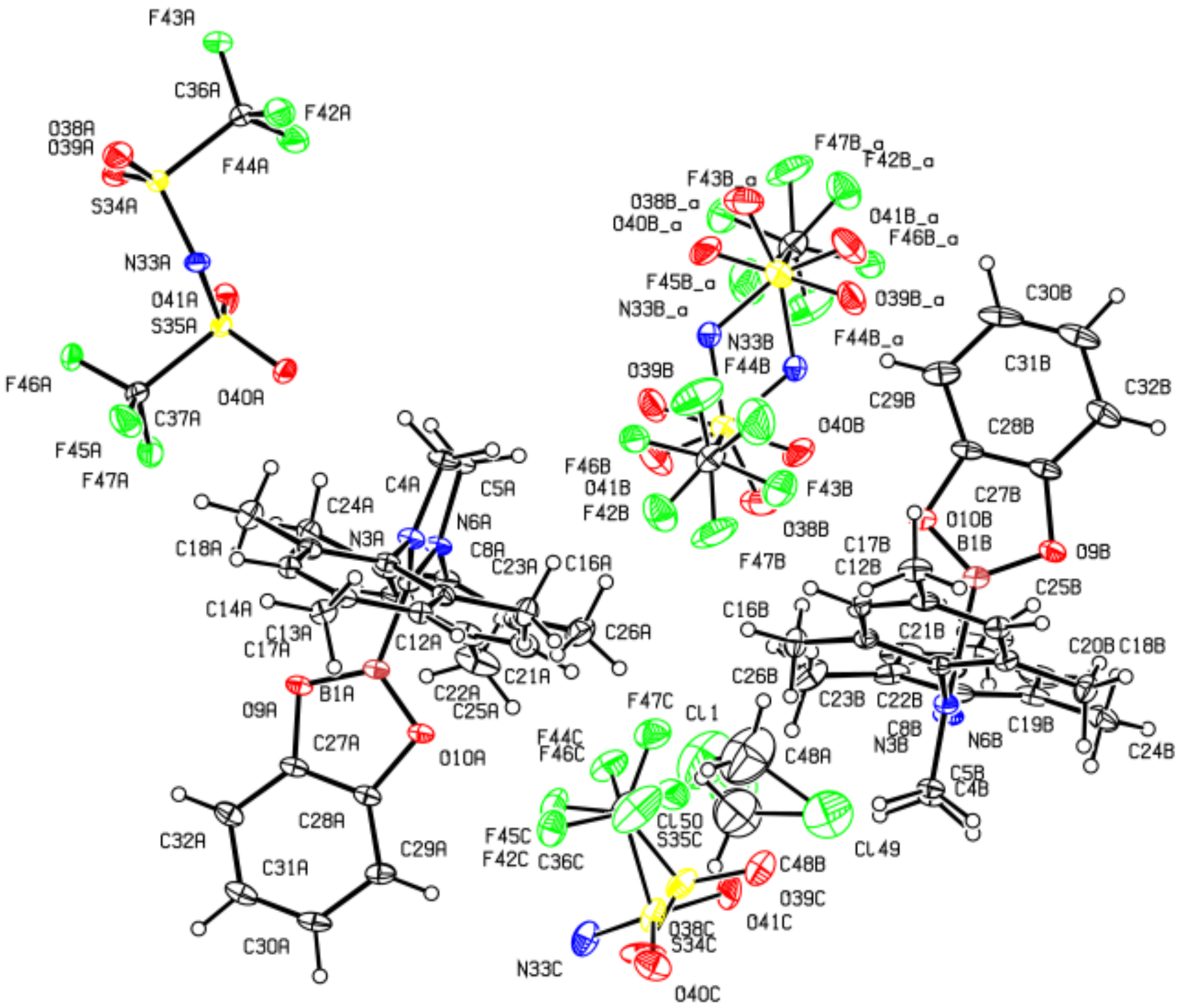

Figure S14: Molecular structure view of ***2b*** including the anion.

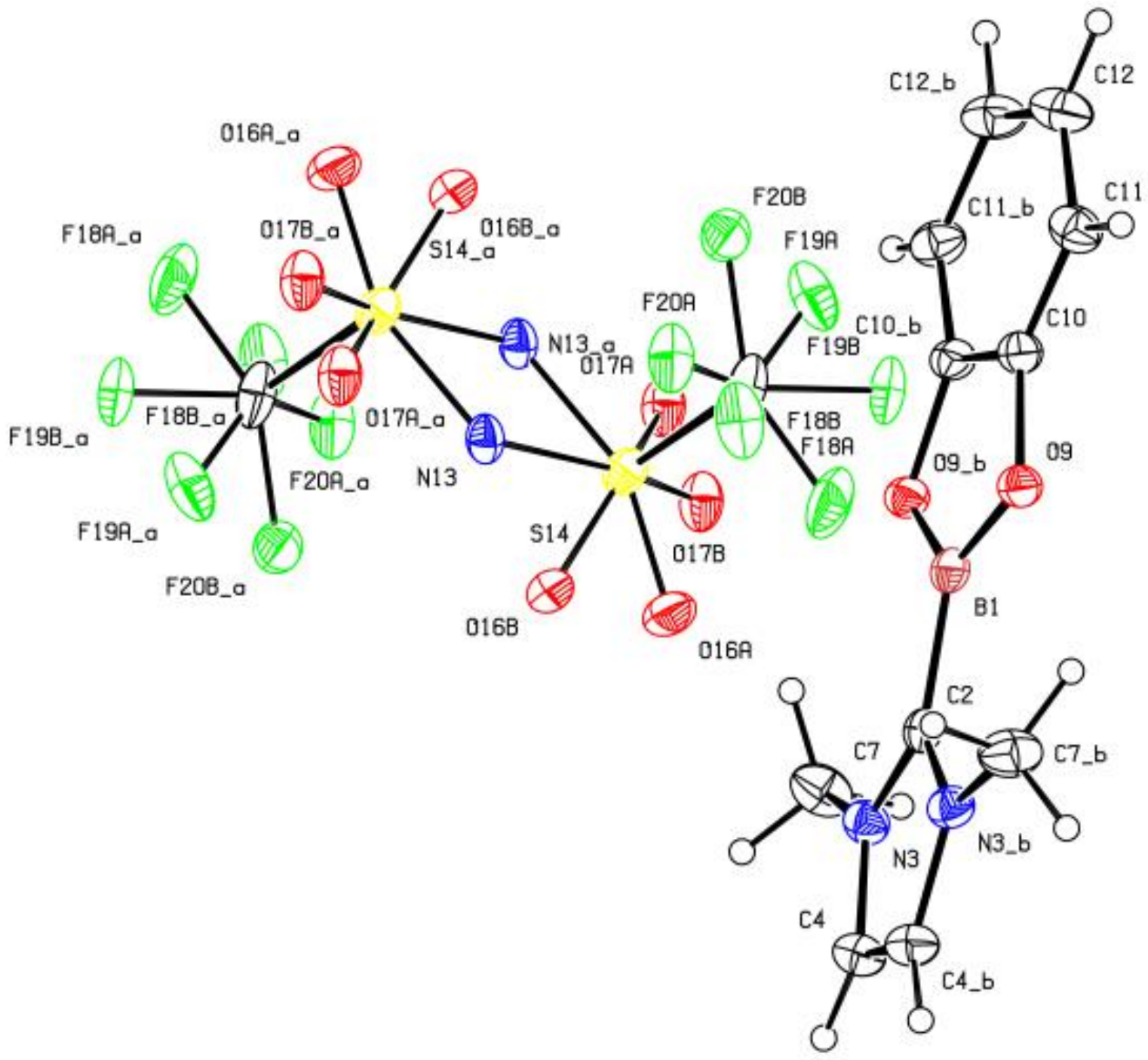

Figure S15: Molecular structure view of ***3b*** including the anion.

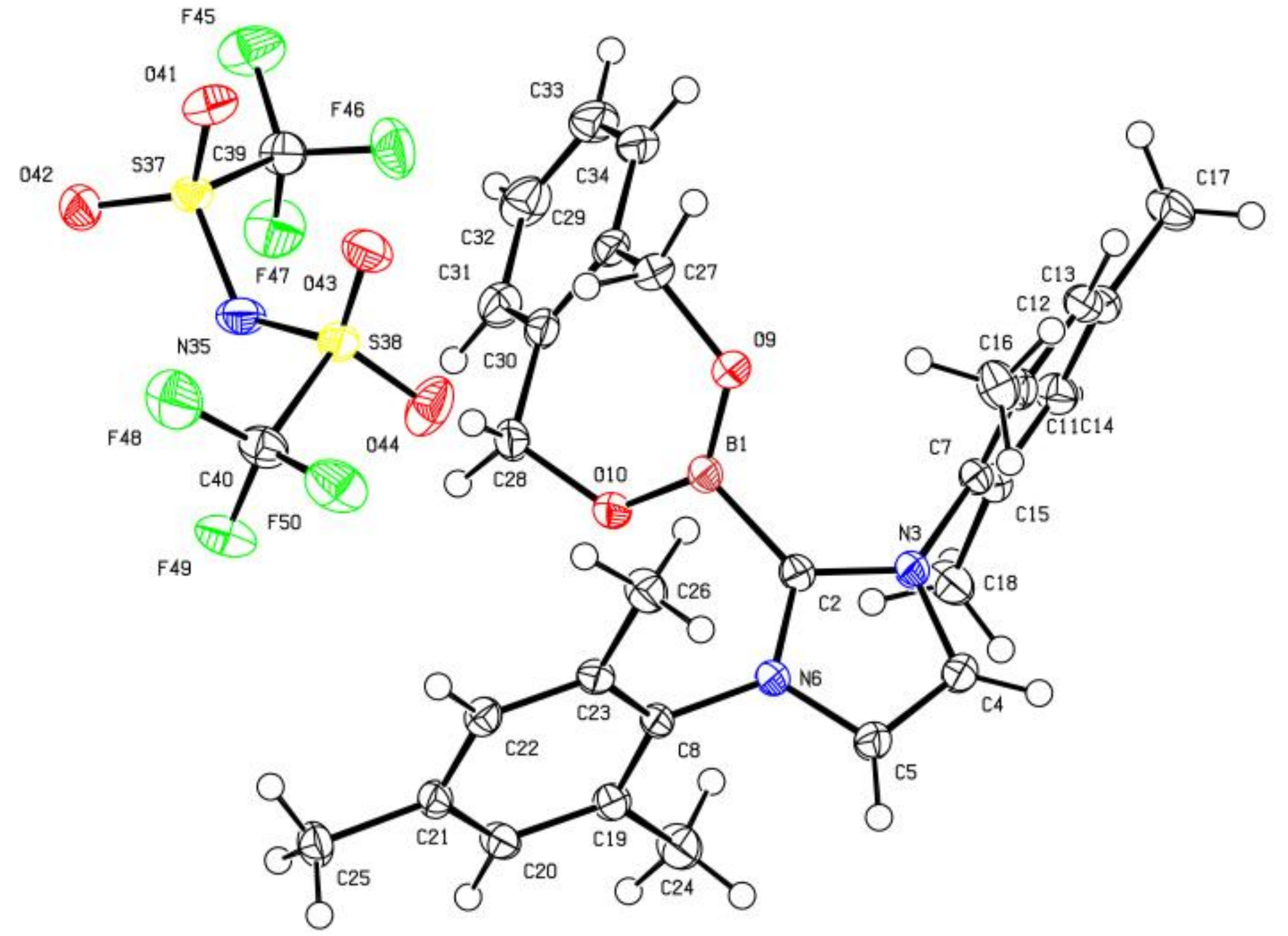

Figure S16: Molecular structure view of ***1f*** including the anion.

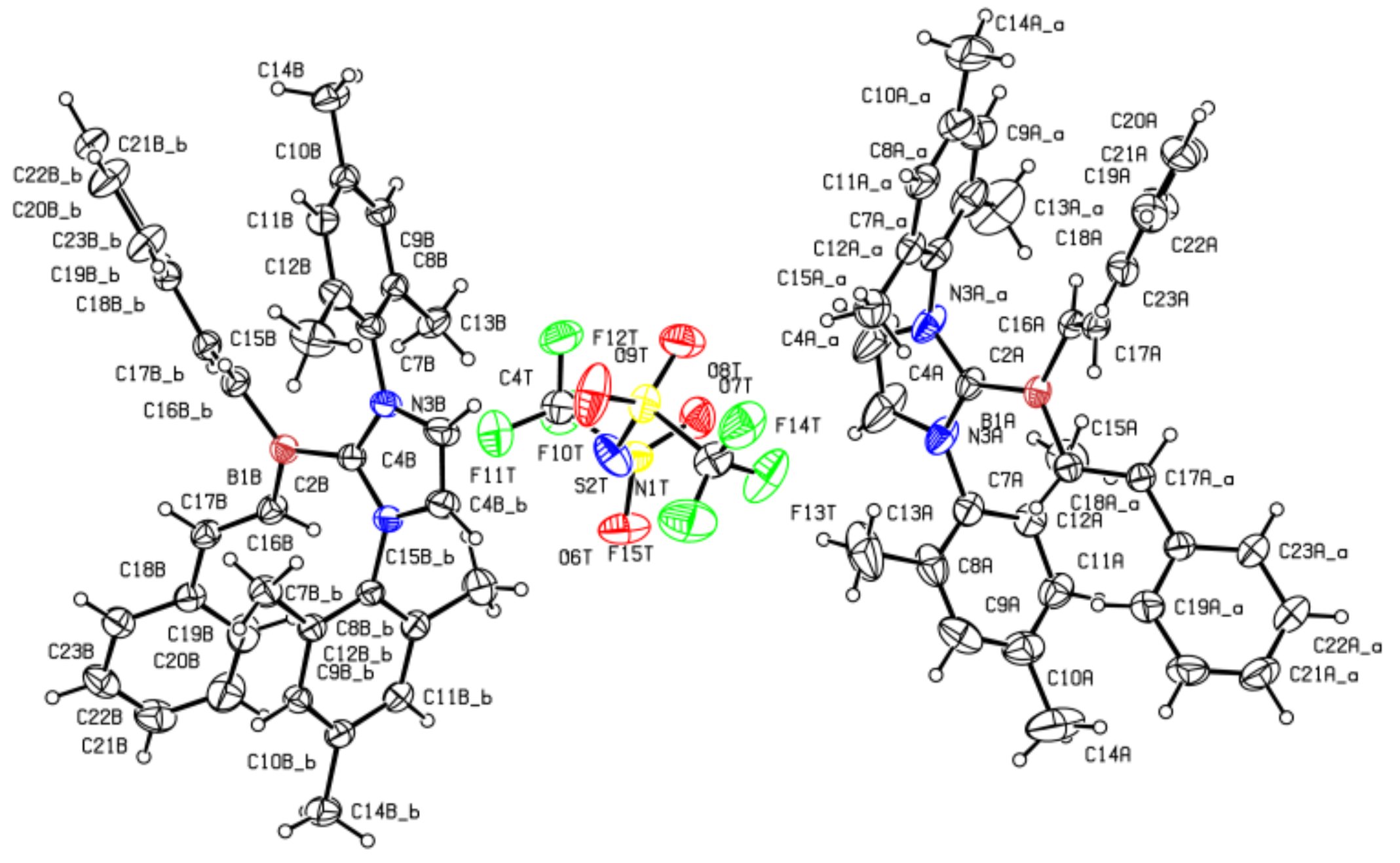

Figure S17: Molecular structure view of ***1d*** including the anion.

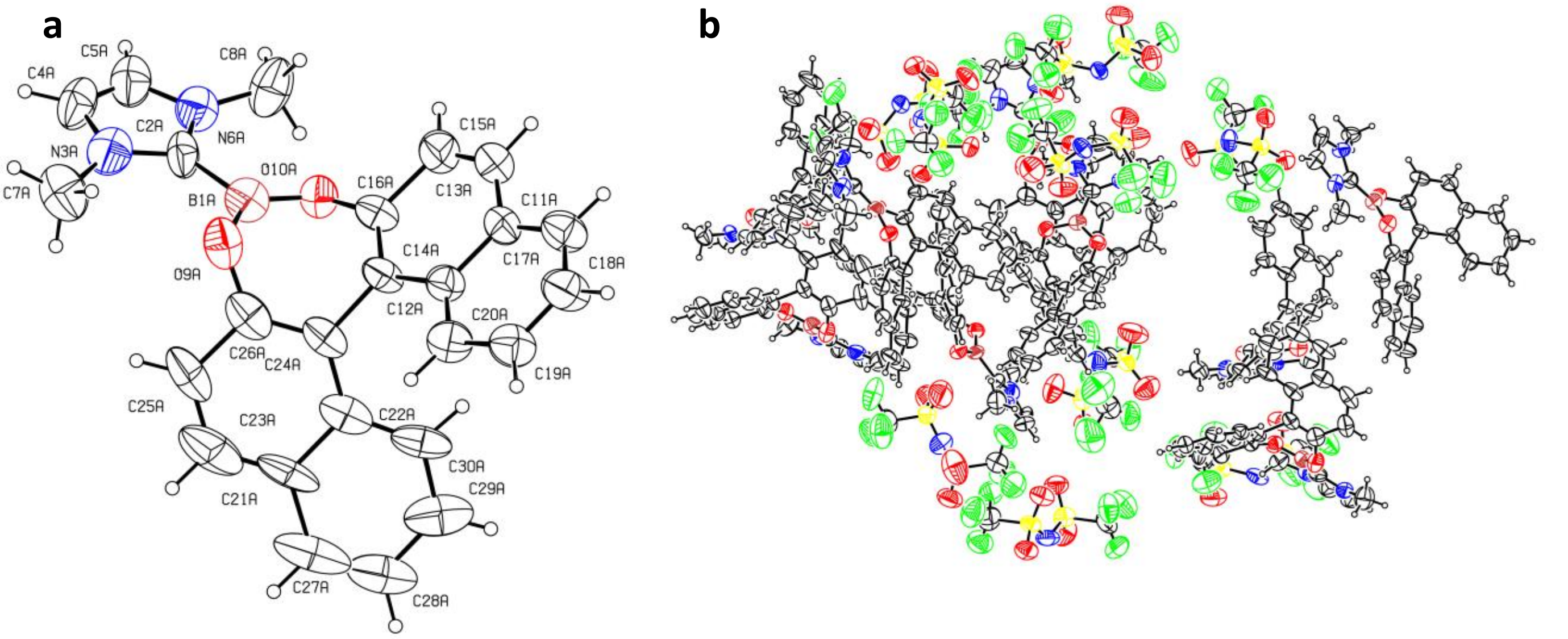

Figure S18: **a**) Molecular structure view of compound ***3e***. Thermal ellipsoids are shown at the 30% probability level. Anion and all remaining independent molecules (eight out of nine) are omitted for clarity. **b**) Full molecular structure view of compound ***3e*** including all the independent molecules and anions.

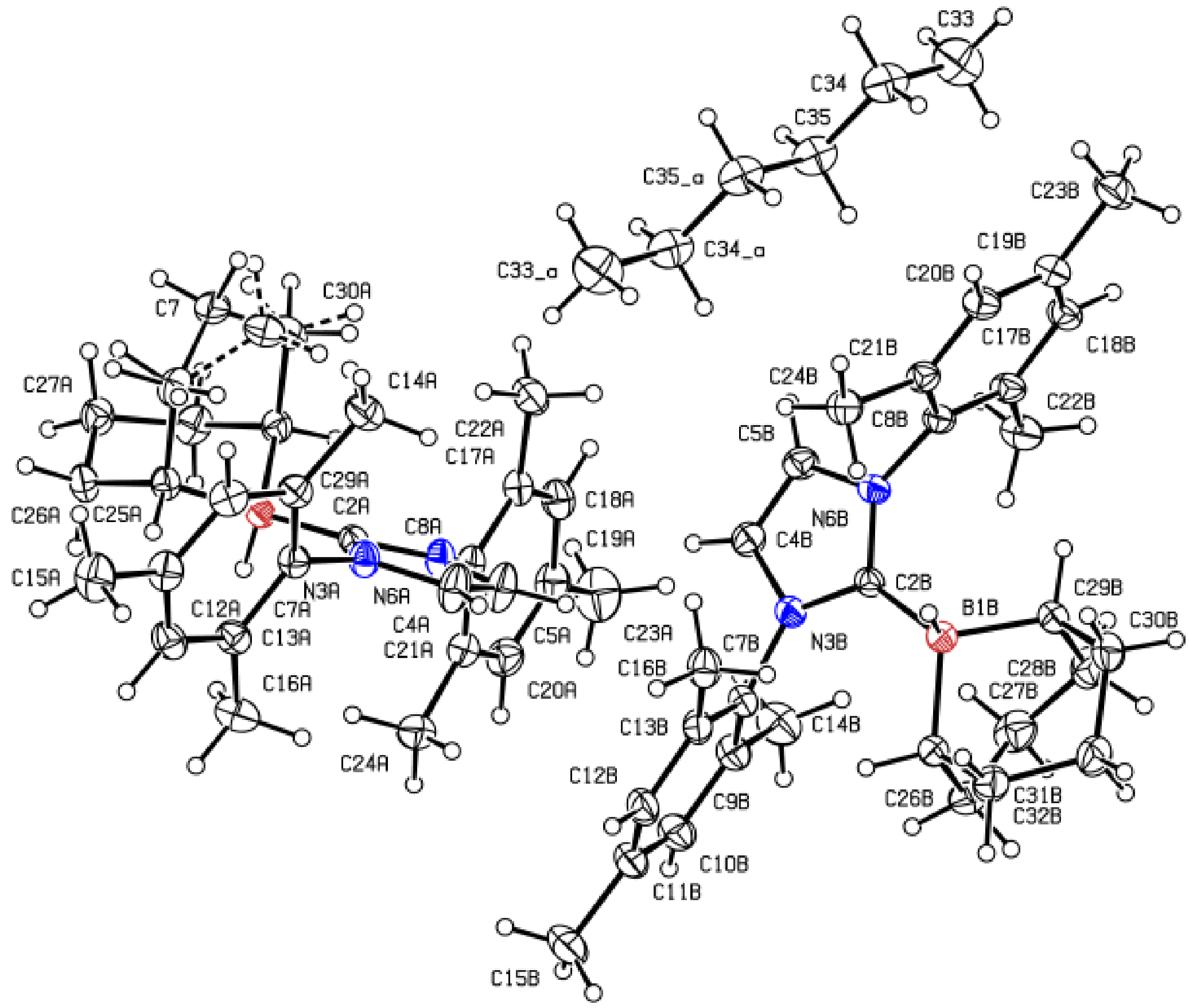

Figure S19: Full molecular structure view of ***1h*H**.

COMPUTATIONAL DETAILS

Fluoride ion affinity (FIA - **Table S3**) and hydride ion affinity (HIA - **Table S4**) were calculated at the final PW6B95(D3)/def2-QZVPP level of theory using geometries optimized at PBE0(D3)/def2-TZVP level and thermal corrections at the same level. In order to account for solvent effects, the PCM model was applied. In all cases the TMS-anchor reaction system was used (Me_3Si^+/Me_3SiF and Me_3Si^+/Me_3SiH for FIA and HIA respectively). The FIA value of 952.5 kJ·mol^{-1} and HIA value of 924 kJ·mol^{-1} reported for Me_3Si^+ at the CCSD(T)/CBS level were used to calculate the gas phase affinities.[6,7] The $FIA_{solv.}$ and $HIA_{solv.}$ were obtained by using the PCM-calculated solvation energies at the final level of theory, according to the mentioned Greb *et al.* procedure.[6] In our case, the F^- solvation energy was calculated to be -325.6 kJ·mol^{-1}, and for H^- equal to -321.1 kJ·mol^{-1} at the PW6B95(D3)/def2-QZVPP level of theory. It has to be highlighted, that due to the problematic nature of F^- and H^- computational solvation, the resulting $FIA_{solv.}$ and $HIA_{solv.}$ values are not absolute and intended just for comparison within the discussed group of compounds.

Table S3: Source data for FIA and $FIA_{solv.}$ calculations (kJ·mol^{-1} at 298.15 K).

Me_3SiF	-1338229.5	kJ
Me_3Si+	-1074747.7	kJ
TMS anchor	952.5	kJ
D*G*(solv.) fluoride	-325.561922	kJ

Comp. (cation, F_add.)	Thermal corr. [kJ*mol^{-1}]	Electr. Energy [Hartree]	Electr. Energy [kJ*mol^{-1}]	Electr. Energy + Therm. Correct. [kJ*mol^{-1}]	D*H*	FIA	PCM kJ	Diff_solv	FIA solv.
1a	1177.93	-951.71997	-2498740.4	-2497562.5			-139.8		
1a_F	1186.59	-1051.98731	-2761992.3	-2760805.7	238.7	**713.8**	-39.3	426.1	**287.8**
1b	1384.22	-1332.79295	-3499247.4	-3497863.2			-133.1		
1b_F	1387.38	-1433.04240	-3762452.3	-3761064.9	280.1	**672.4**	-36.8	421.9	**250.5**
1c	1994.64	-1421.91282	-3733231.6	-3731236.9			-125.2		
1c_F	1999.59	-1522.13942	-3996376.5	-3994376.9	341.9	**610.6**	-32.1	418.7	**191.9**
1d	1827.07	-1569.78130	-4121460.2	-4119633.1			-126.7		
1d_F	1831.87	-1670.01502	-4384623.8	-4382791.9	323.1	**629.4**	-36.5	415.8	**213.6**
1e	1870.48	-1872.08575	-4915160.4	-4913289.9			-128.9		
1e_F	1876.69	-1972.33004	-5178351.8	-5176475.1	296.8	**655.7**	-42.7	411.7	**244.0**
1f	1544.35	-1411.55836	-3706045.9	-3704501.6			-128.3		
1f_F	1548.60	-1511.78596	-3969193.5	-3967644.9	338.6	**613.9**	-34.0	419.8	**194.1**
1g	1609.67	-1564.26249	-4106970.6	-4105360.9			-129.4		
1g_F	1615.40	-1664.50525	-4370157.9	-4368542.5	300.3	**652.2**	-41.7	413.2	**239.0**
1h	1697.09	-1264.39789	-3319676.2	-3317979.1			-128.1		
1h_F	1704.42	-1364.63679	-3582853.4	-3581148.9	312.0	**640.5**	-29.3	424.3	**216.2**
2a	1239.56	-952.91258	-2501871.6	-2500632.1			-136.9		
2a_F	1249.25	-1053.19041	-2765151.0	-2763901.8	212.2	**740.3**	-40.8	421.6	**318.7**
2b	1447.46	-1333.99079	-3502392.3	-3500944.8			-131.5		
2b_F	1452.82	-1434.24257	-3765603.3	-3764150.5	276.2	**676.3**	-41.3	415.8	**260.5**
3a	424.90	-331.23726	-869663.3	-869238.4			-178.1		
3a_F	433.25	-431.51940	-1132954.0	-1132520.8	199.5	**753.0**	-35.8	467.8	**285.2**
3b	631.05	-712.31399	-1870180.1	-1869549.0			-160.2		
3b_F	636.80	-812.56753	-2133395.7	-2132758.9	272.0	**680.5**	-40.9	444.8	**235.7**
3c	1240.48	-801.42412	-2104138.7	-2102898.2			-146.4		
3c_F	1245.87	-901.66522	-2367321.7	-2366075.8	304.3	**648.2**	-33.2	438.7	**209.5**
3d	1072.94	-949.29511	-2492374.0	-2491301.0			-145.3		
3d_F	1078.11	-1049.53511	-2755554.0	-2754475.9	307.0	**645.5**	-41.1	429.7	**215.8**
3e	1116.66	-1251.59997	-3286075.3	-3284958.6			-153.8		
3e_F	1122.98	-1351.86179	-3549312.6	-3548189.6	250.8	**701.7**	-43.4	436.0	**265.7**
3g	856.13	-943.77703	-2477886.2	-2477030.1			-155.8		
3g_F	862.04	-1044.03000	-2741100.4	-2740238.3	273.7	**678.8**	-45.2	436.2	**242.7**
4a	488.99	-2211.87253	-5807270.5	-5806781.5			-17.1		
4a_F	495.12	-2312.03542	-6070248.1	-6069753.0	510.4	**442.1**	-131.6	211.1	**231.0**

Table S4: Source data for HIA and $HIA_{solv.}$ calculations ($kJ\cdot mol^{-1}$ at 298.15 K).

Me_3SiH	-1077046.0	kJ
Me_3Si+	-1074747.7	kJ
TMS anchor	924.0	kJ
DG(solv.) hydride	-321.110456	kJ

Comp. (cation, H_add.)	Thermal corr. [kJ*mol-1]	Electr. Energy [Hartree]	Electr. Energy [kJ*mol-1]	Electr. Energy + Therm. Correct. [kJ*mol-1]	DH	HIA	PCM kJ	Diff_solv	HIA solv.
1a	1177.93	-951.71997	-2498740.4	-2497562.5			-139.8		
1a_H	1198.81	-952.53754	-2500887.0	-2499688.1	172.6	**751.4**	-34.7	426.3	**325.1**
1b	1384.22	-1332.79295	-3499247.4	-3497863.2			-133.1		
1b_H	1404.50	-1333.56996	-3501287.4	-3499882.9	278.5	**645.5**	-35.7	418.5	**227.0**
1c	1994.64	-1421.91282	-3733231.6	-3731236.9			-125.2		
1c_H	2012.91	-1422.68229	-3735251.8	-3733238.9	296.3	**627.7**	-26.8	419.5	**208.2**
1d	1827.07	-1569.78130	-4121460.2	-4119633.1			-126.7		
1d_H	1845.58	-1570.55802	-4123499.5	-4121653.9	277.5	**646.5**	-31.9	415.9	**230.6**
1e	1870.48	-1872.08575	-4915160.4	-4913289.9			-128.9		
1e_H	1890.86	-1872.85856	-4917189.4	-4915298.6	289.6	**634.4**	-42.8	407.1	**227.2**
1f	1544.35	-1411.55836	-3706045.9	-3704501.6			-128.3		
1f_H	1562.26	-1412.31337	-3708028.2	-3706465.9	333.9	**590.1**	-29.6	419.7	**170.4**
1g	1609.67	-1564.26249	-4106970.6	-4105360.9			-129.4		
1g_H	1629.90	-1565.03465	-4108997.9	-4107368.0	291.2	**632.8**	-37.8	412.8	**220.0**
1h	1697.09	-1264.39789	-3319676.2	-3317979.1			-128.1		
1h_H	1717.44	-1265.18042	-3321730.7	-3320013.3	264.1	**659.9**	-24.1	425.1	**234.8**
2a	1239.56	-952.91258	-2501871.6	-2500632.1			-136.9		
2a_H	1261.59	-953.74034	-2504044.9	-2502783.3	147.0	**777.0**	-36.1	421.9	**355.0**
2b	1447.46	-1333.99079	-3502392.3	-3500944.8			-131.5		
2b_H	1467.10	-1334.77032	-3504439.0	-3502971.9	271.3	**652.7**	-37.1	415.6	**237.1**
3a	424.90	-331.23726	-869663.3	-869238.4			-178.1		
3a_H	445.14	-332.06870	-871846.2	-871401.1	135.6	**788.4**	-31.9	467.3	**321.1**
3b	631.05	-712.31399	-1870180.1	-1869549.0			-160.2		
3b_H	650.29	-713.09262	-1872224.4	-1871574.1	273.2	**650.8**	-39.7	441.6	**209.2**
3c	1240.48	-801.42412	-2104138.7	-2102898.2			-146.4		
3c_H	1259.79	-802.20918	-2106199.9	-2104940.1	256.4	**667.6**	-26.2	441.3	**226.3**
3d	1072.94	-949.29511	-2492374.0	-2491301.0			-145.3		
3d_H	1092.21	-950.08034	-2494435.6	-2493343.4	255.9	**668.1**	-34.2	432.2	**235.9**
3e	1116.66	-1251.59997	-3286075.3	-3284958.6			-153.8		
3e_H	1137.31	-1252.38919	-3288147.3	-3287010.0	246.8	**677.2**	-40.1	434.7	**242.4**
3g	856.13	-943.77703	-2477886.2	-2477030.1			-155.8		
3g_H	875.89	-944.55802	-2479936.7	-2479060.8	267.6	**656.4**	-41.6	435.3	**221.1**
4a	488.99	-2211.87253	-5807270.5	-5806781.5			-17.1		
4a_H	510.94	-2212.58582	-5809143.2	-5808632.3	447.5	**476.5**	-125.6	212.7	**263.8**

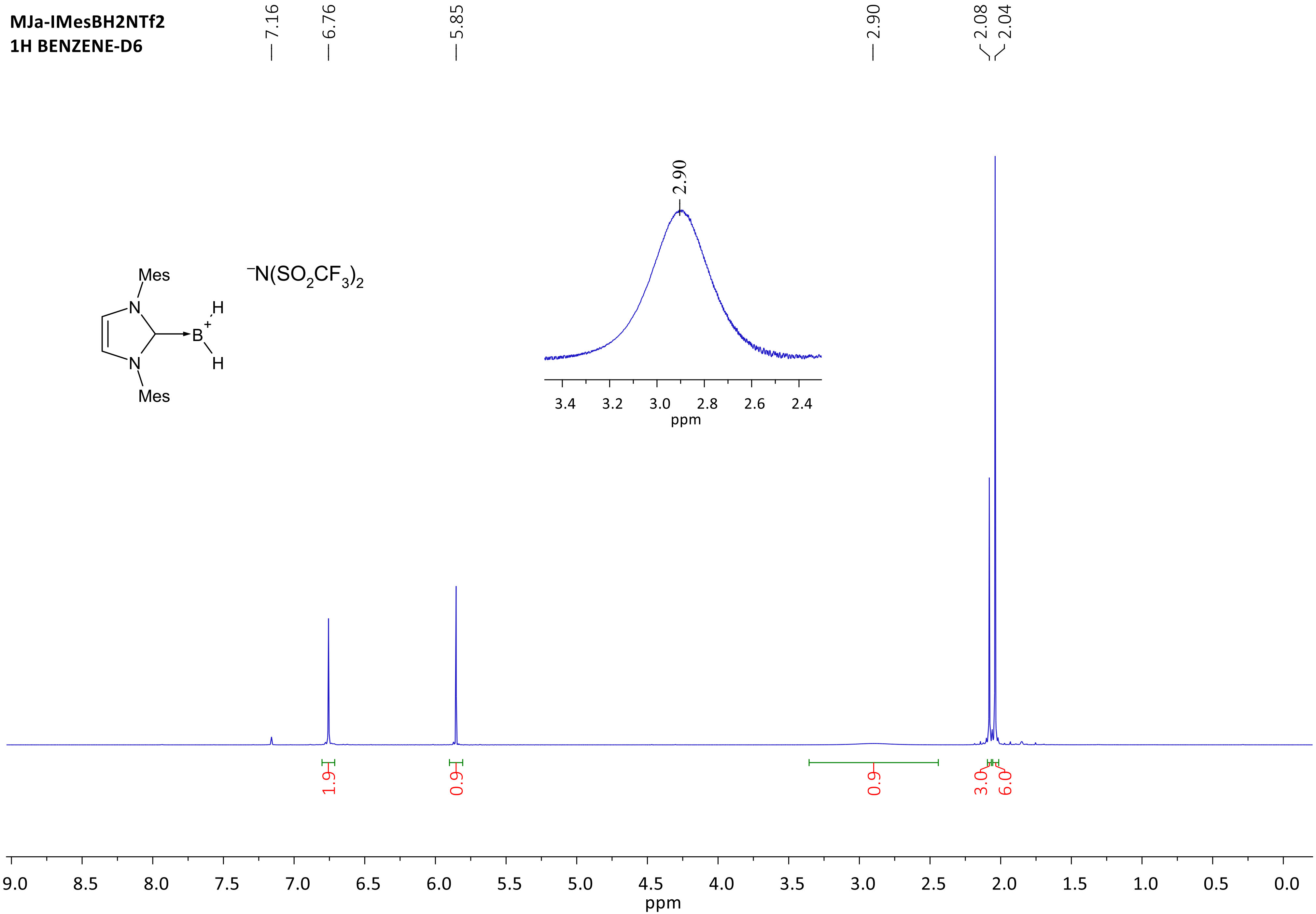

^{1}H NMR for compound ***1a***.

MJa-IMesBH2NTf2
19F BENZENE-D6

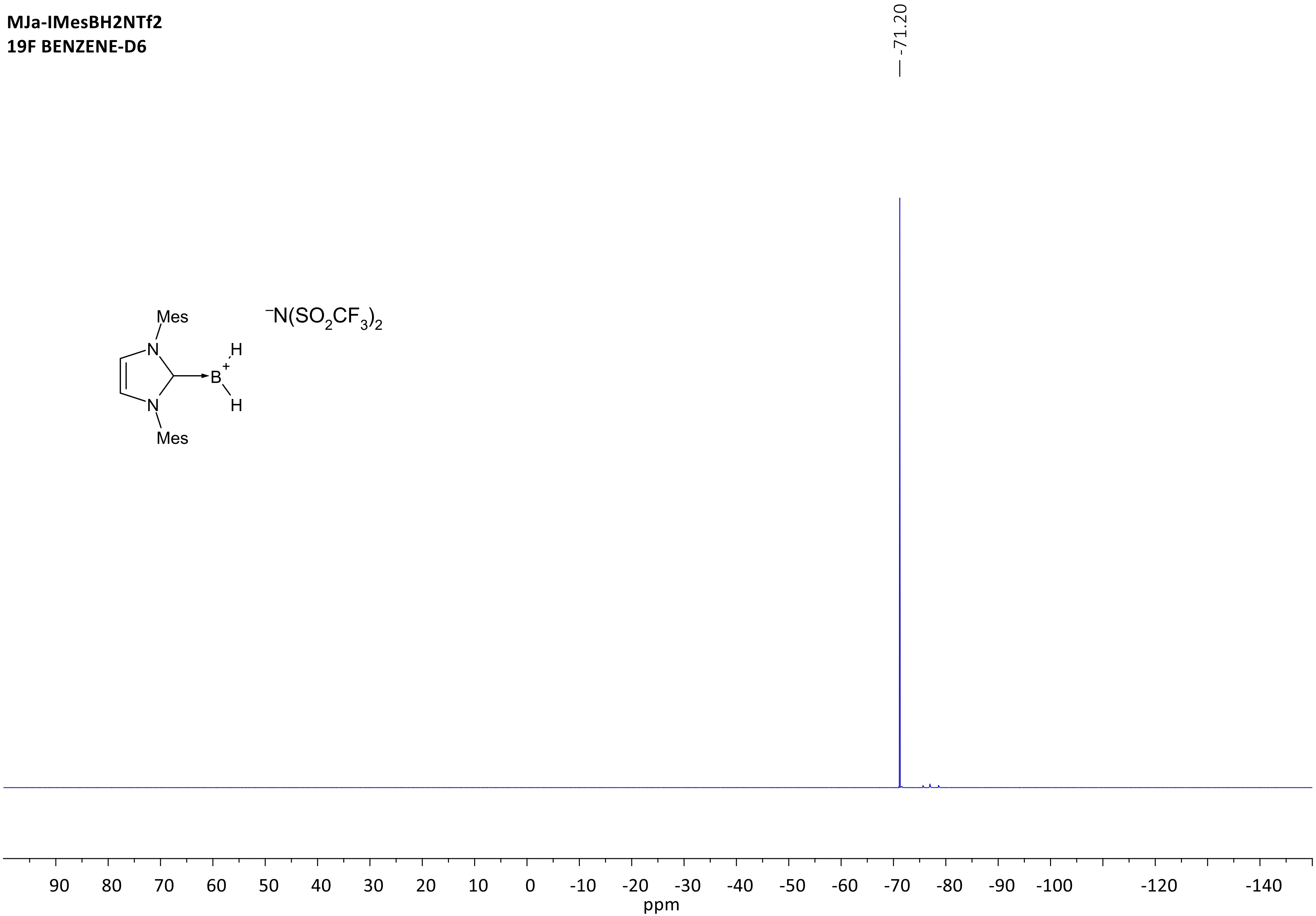

^{19}F NMR for compound ***1a***.

MJa-IMesBH2NTf2
11B BENZENE-D6

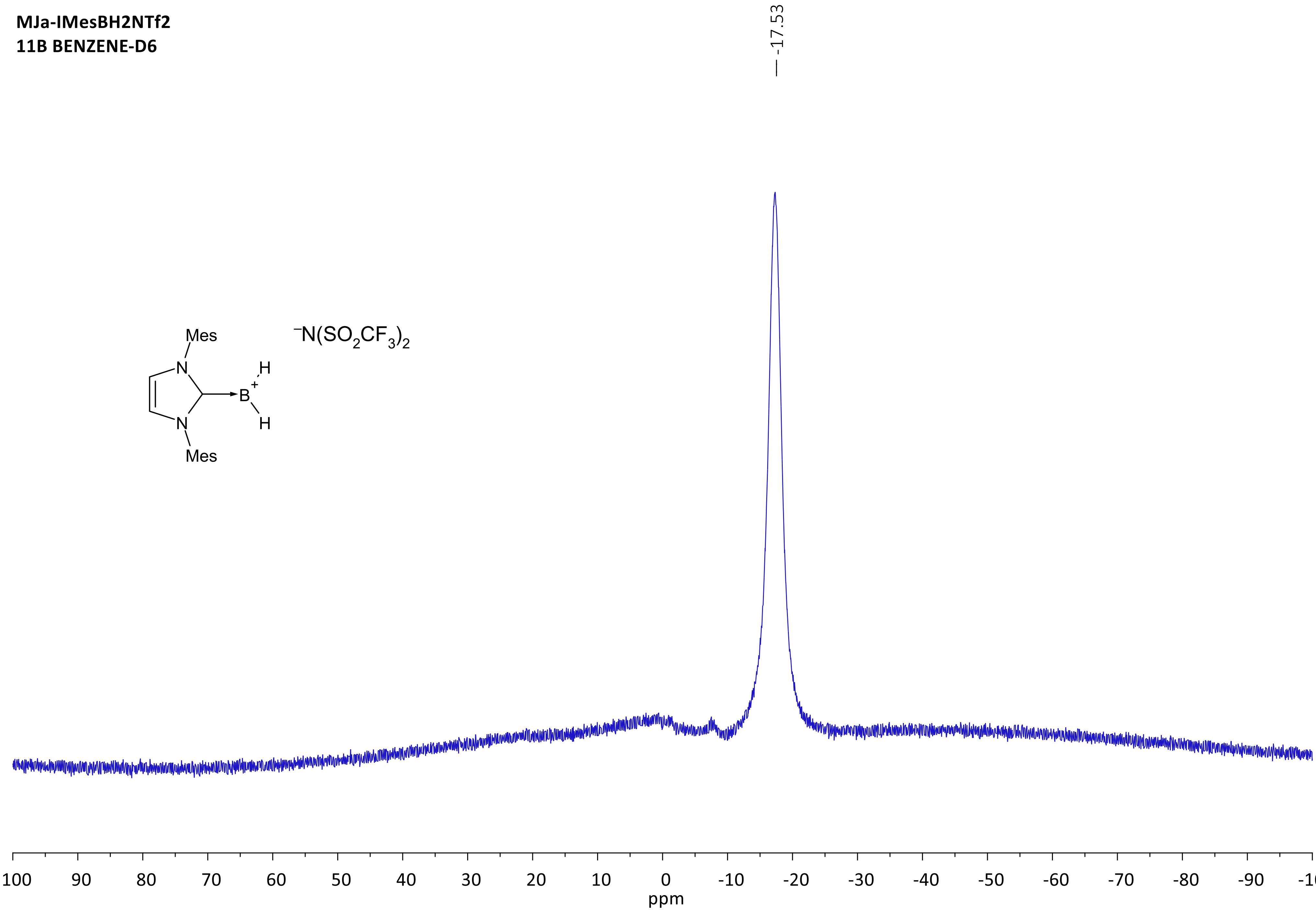

^{11}B NMR for compound ***1a***.

MJa-IMesBH2NTf2
11B BENZENE-D6

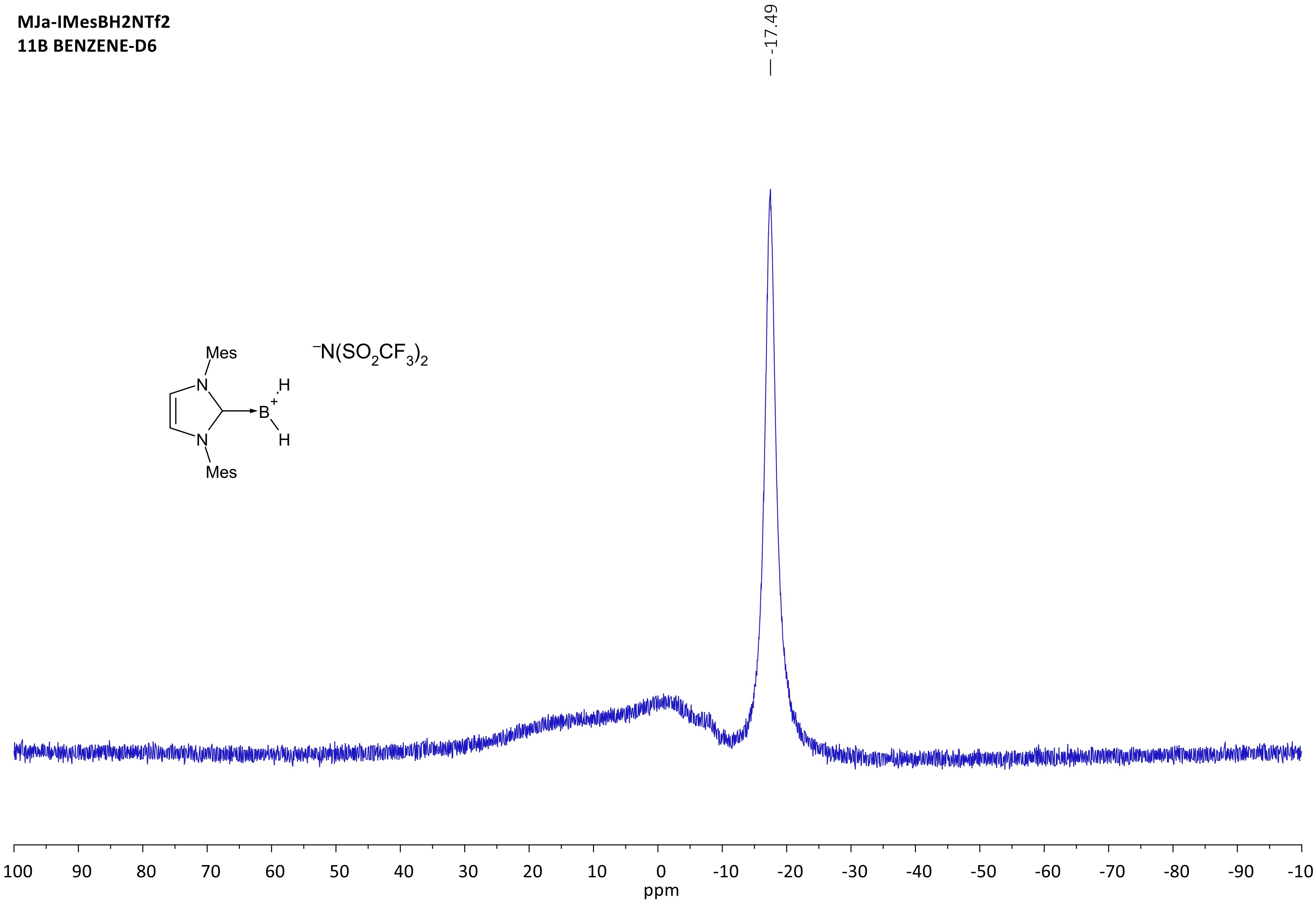

$^{11}B\{^1H\}$ NMR for compound ***1a***.

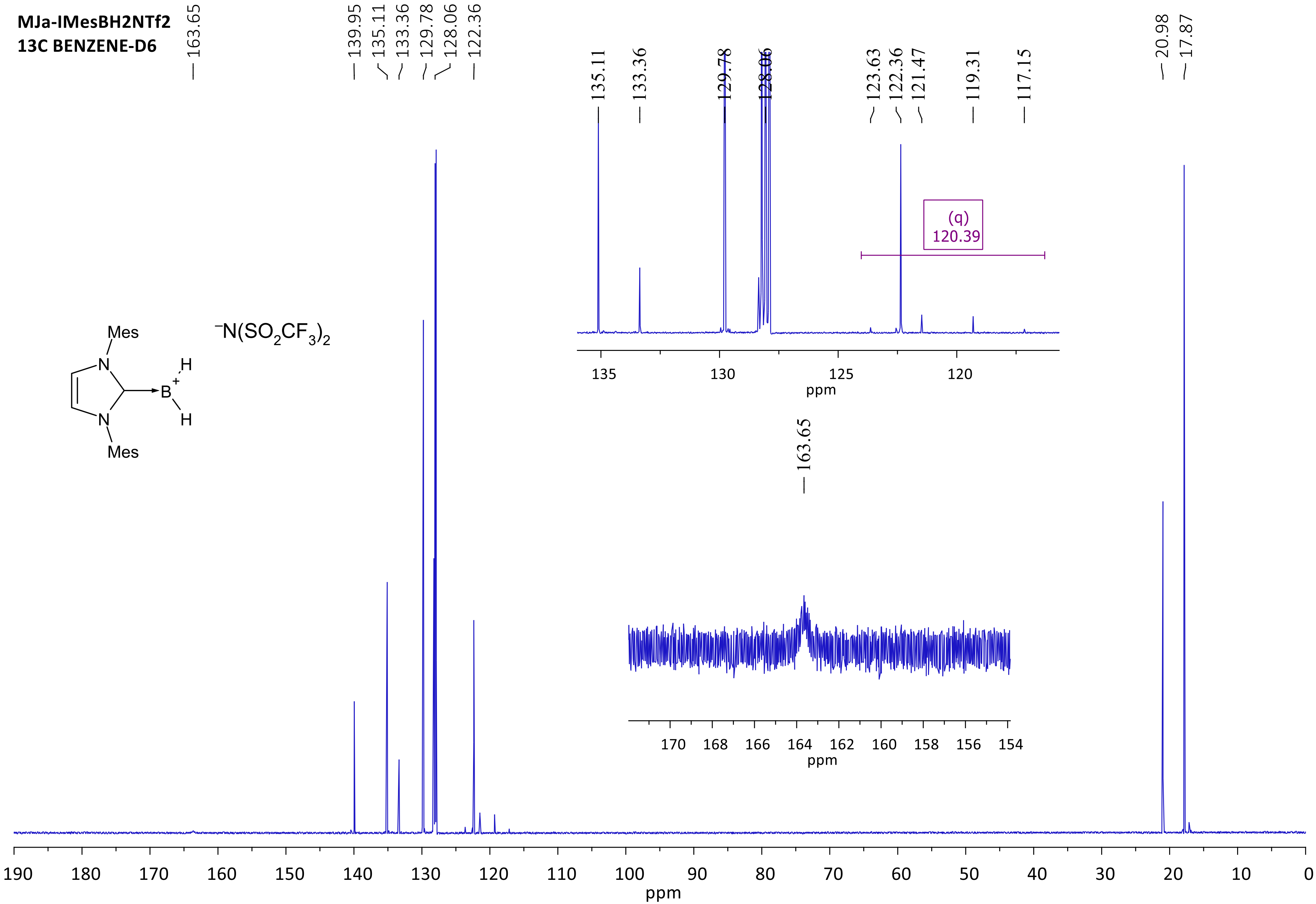

$^{13}C\{^{1}H\}$ NMR for compound ***1a***.

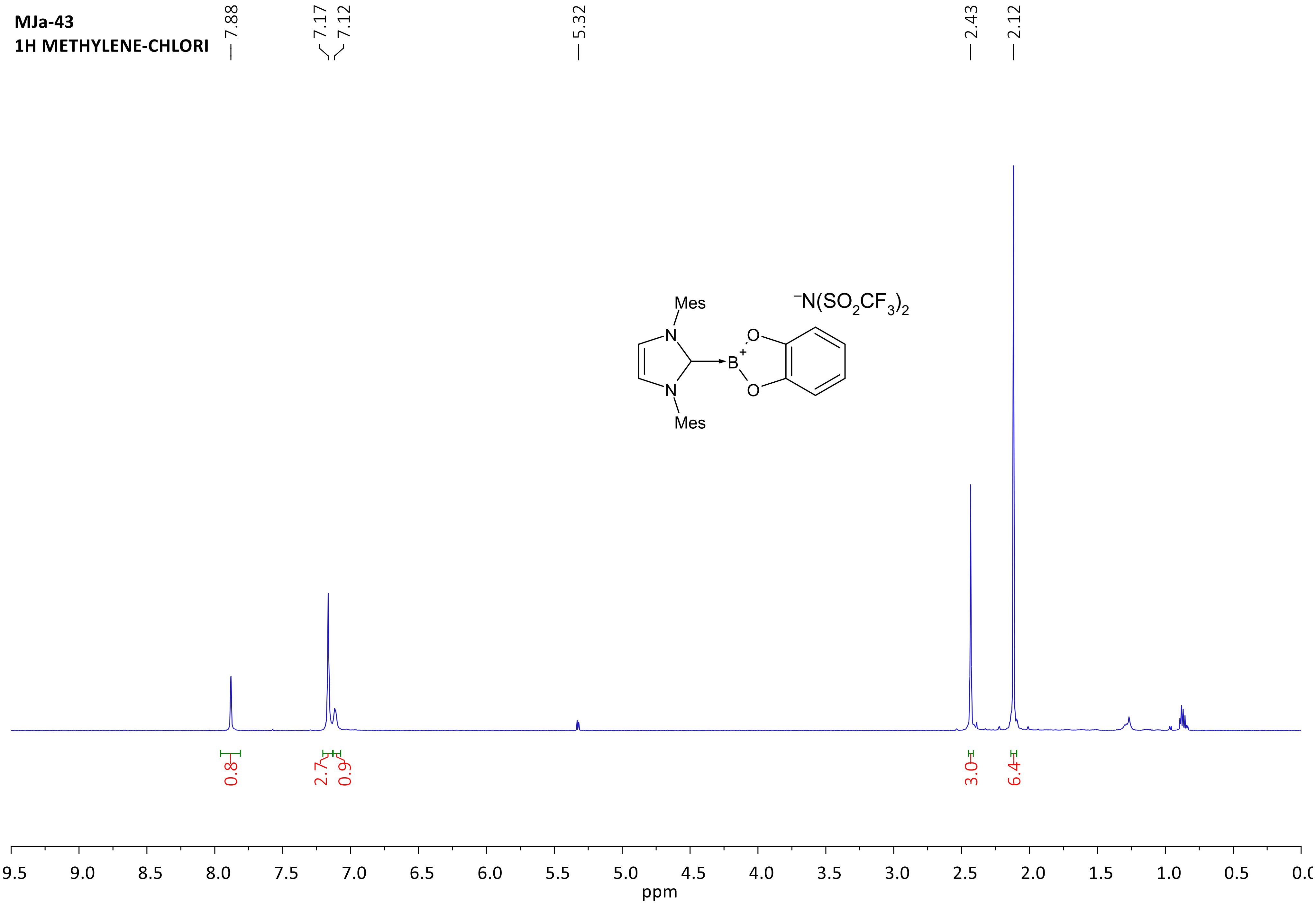

^{1}H NMR for compound ***1b***.

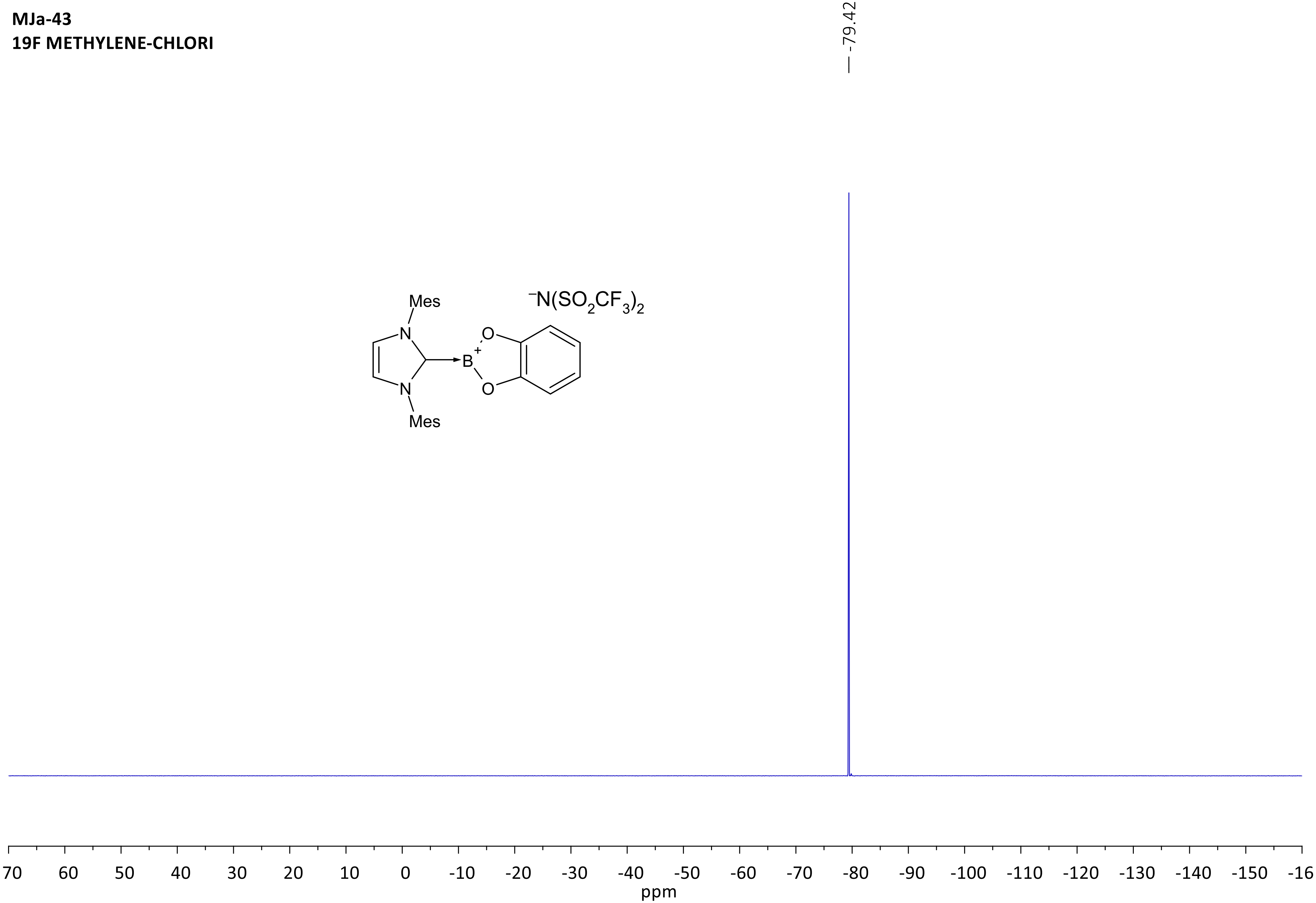

^{19}F NMR for compound ***1b***.

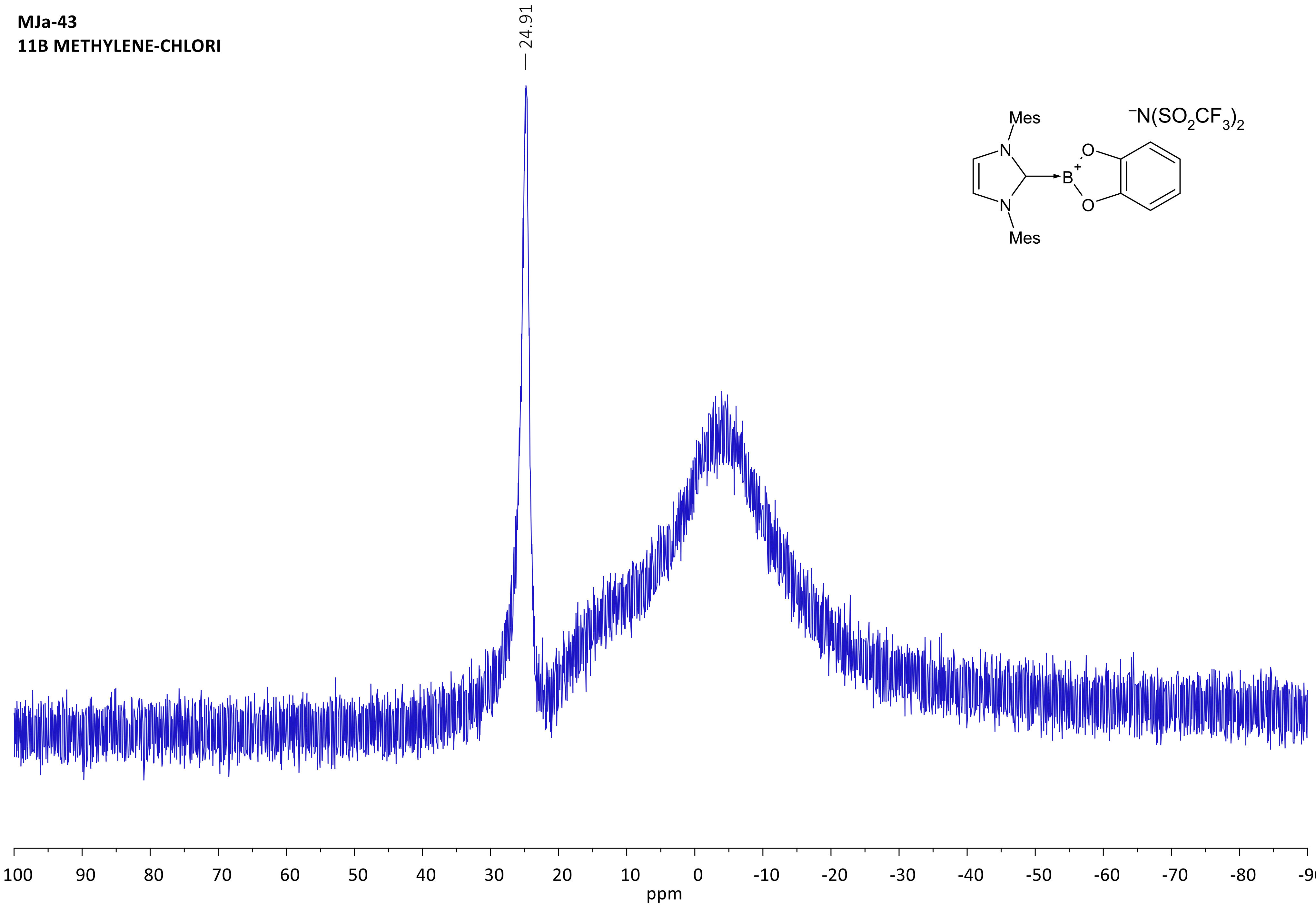

$^{11}B\{^1H\}$ NMR for compound ***1b***.

MJa-43
13C METHYLENE-CHLORI

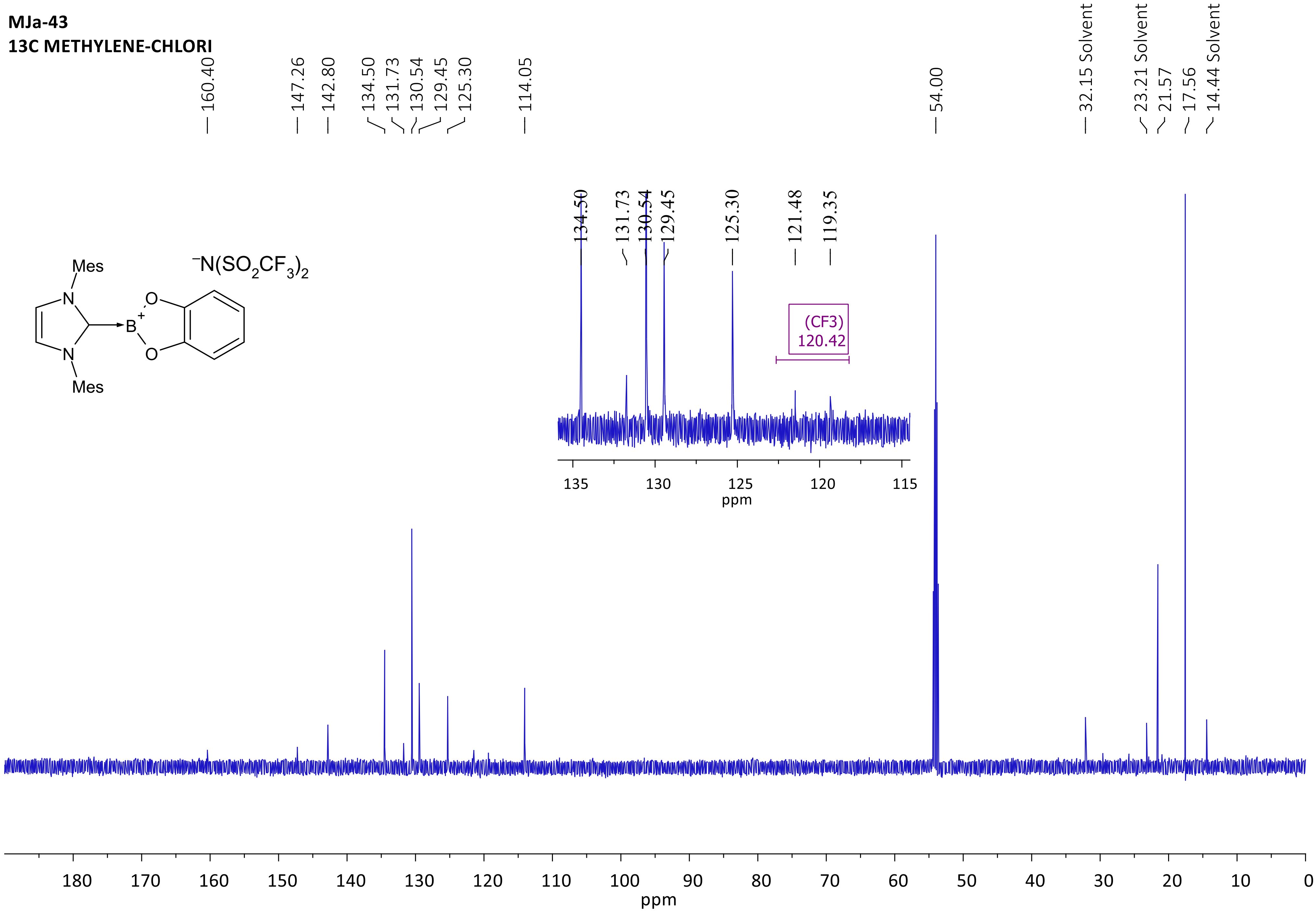

$^{13}C\{^1H\}$ NMR for compound ***1b***.

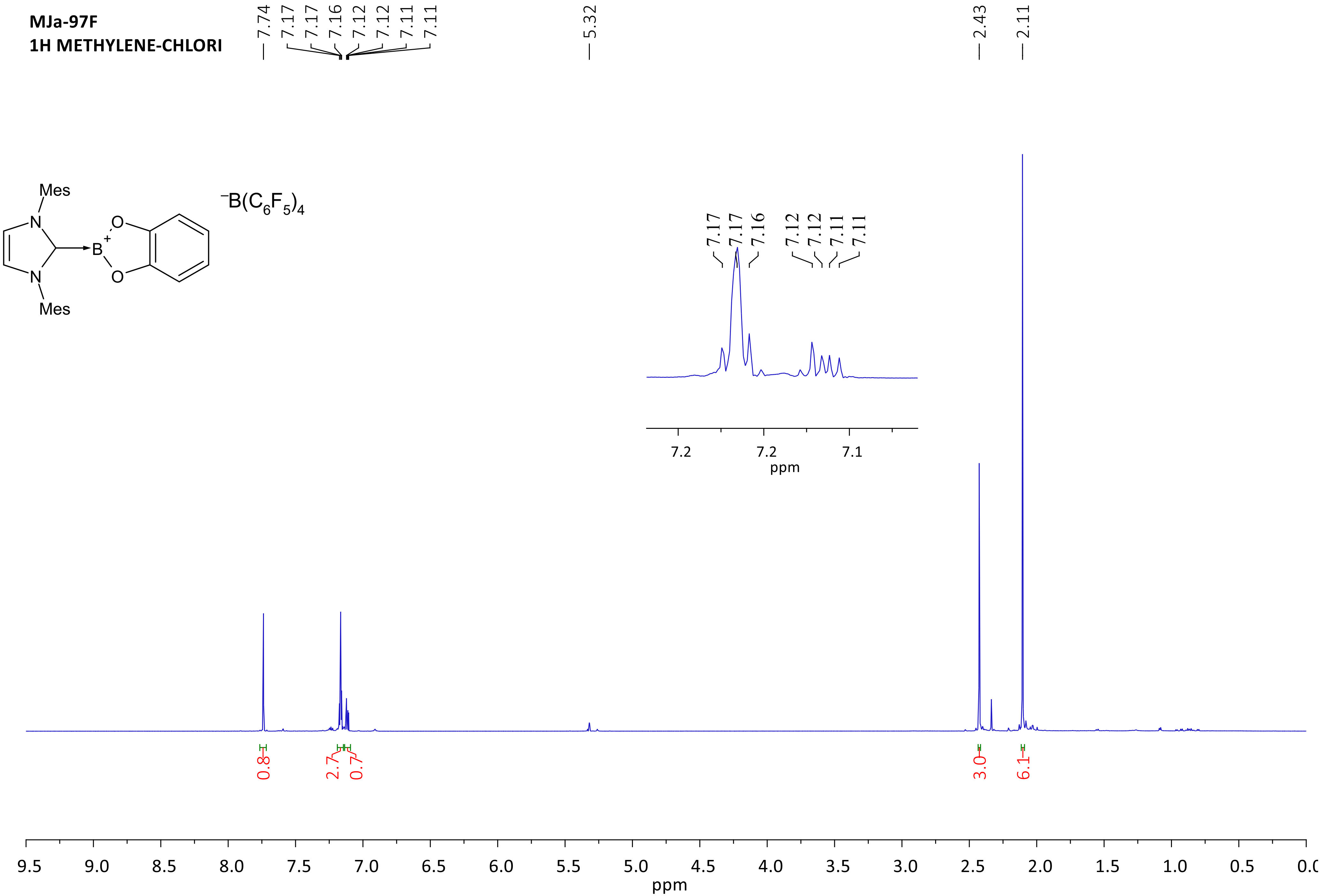

^{1}H NMR for compound ***1b'***.

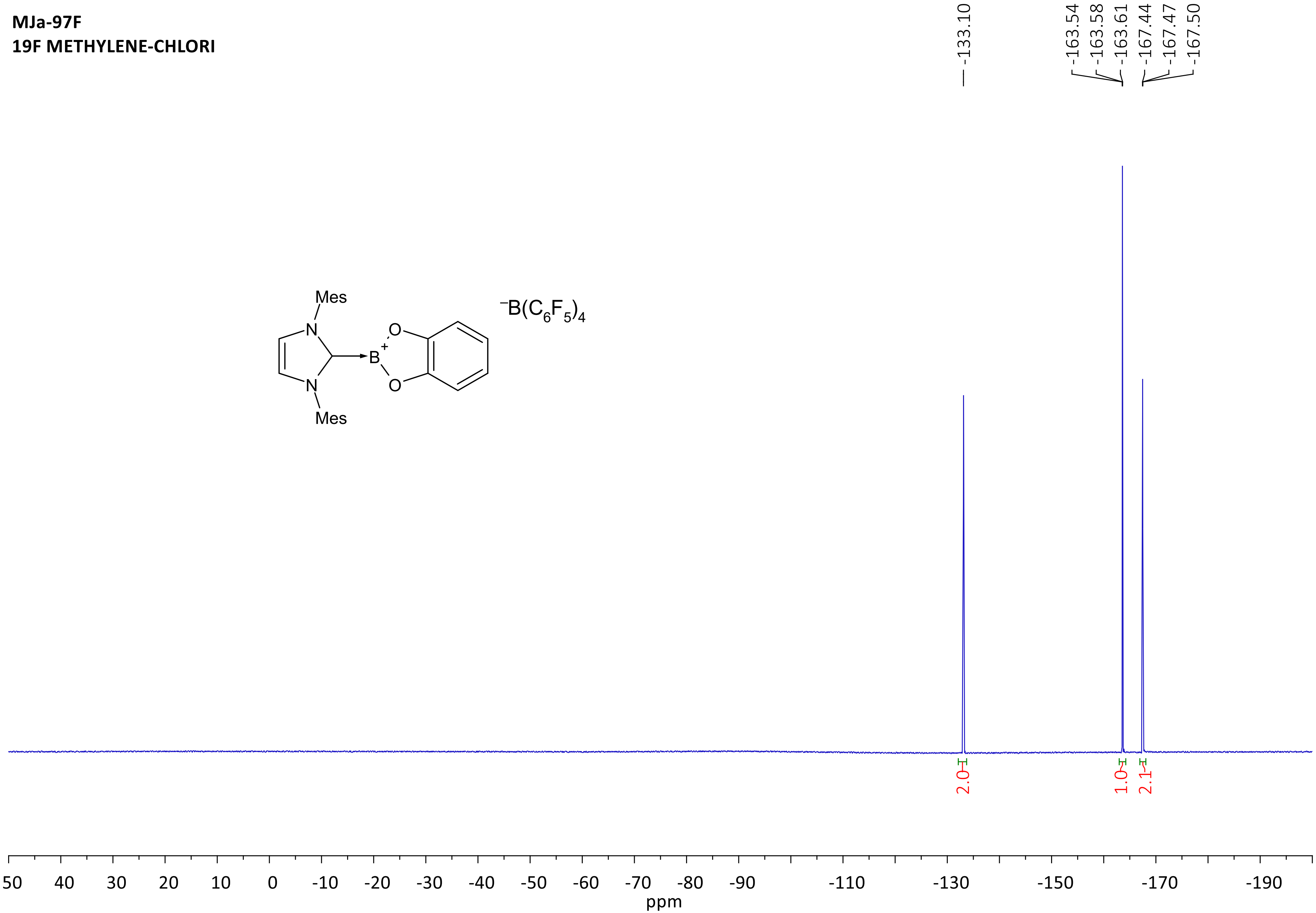

^{19}F NMR for compound ***1b’***.

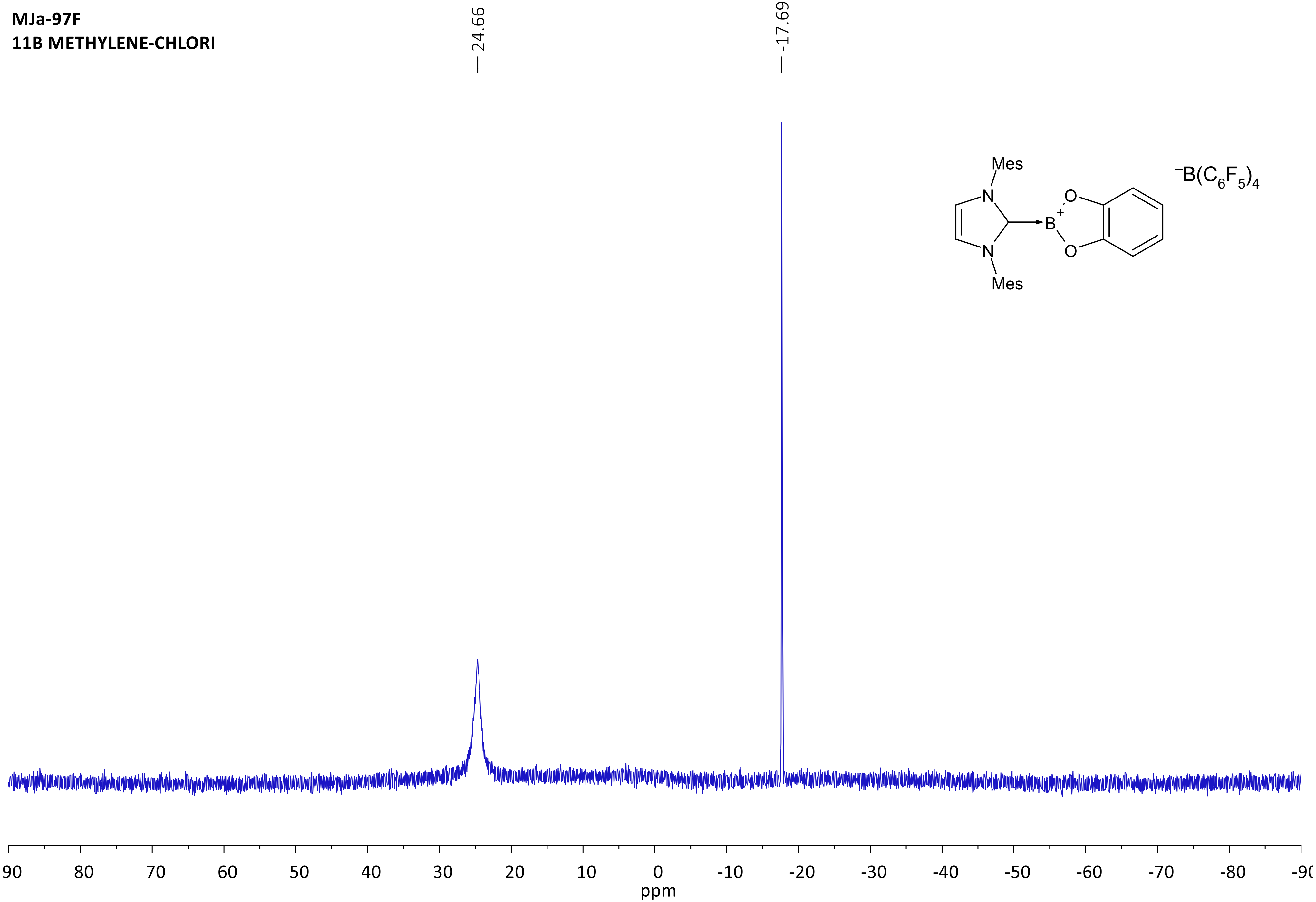

$^{11}B\{^1H\}$ NMR for compound ***1b’***.

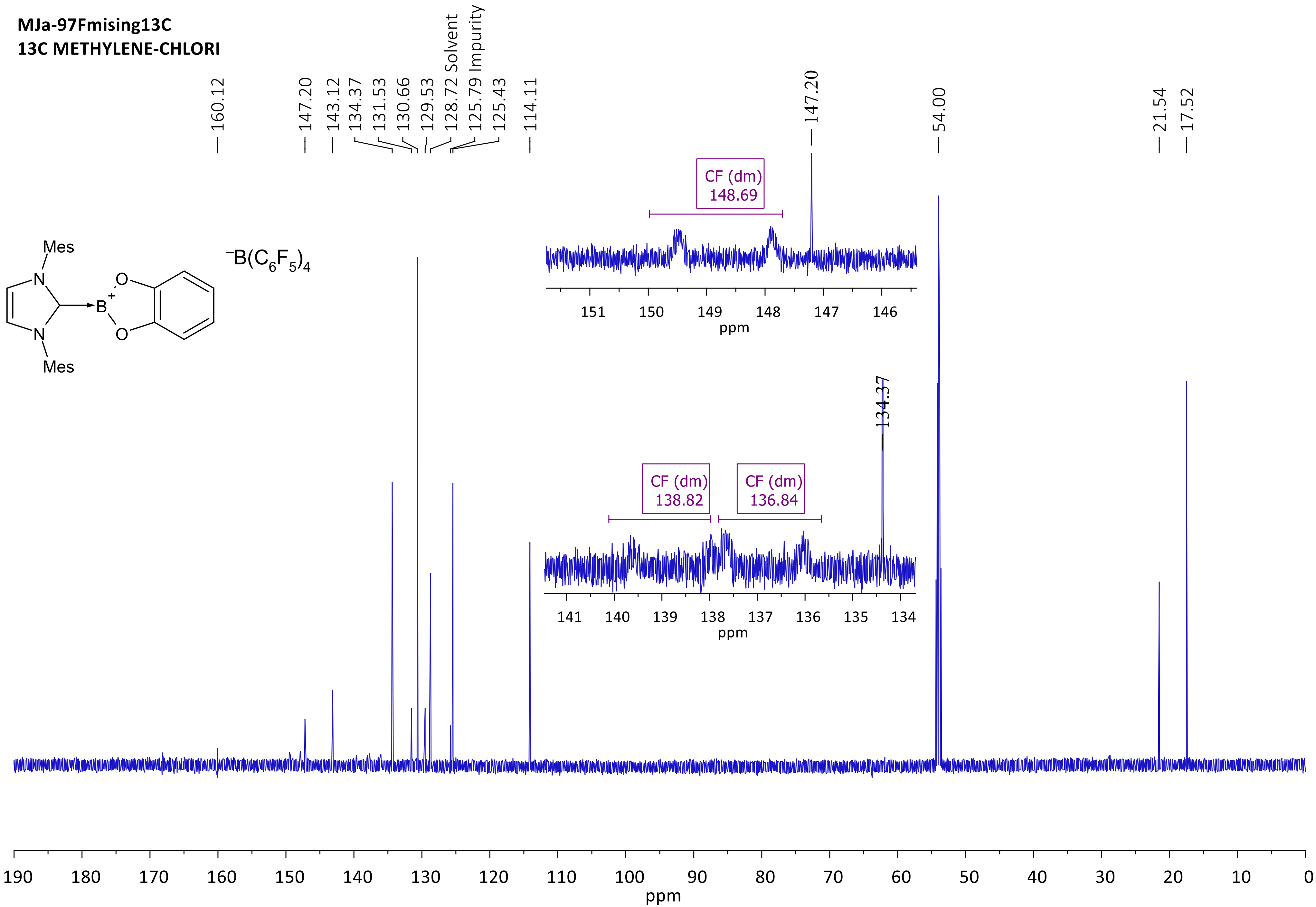

$^{13}C\{^1H\}$ NMR for compound ***1b'***.

MJa-260
1H METHYLENE-CHLORI

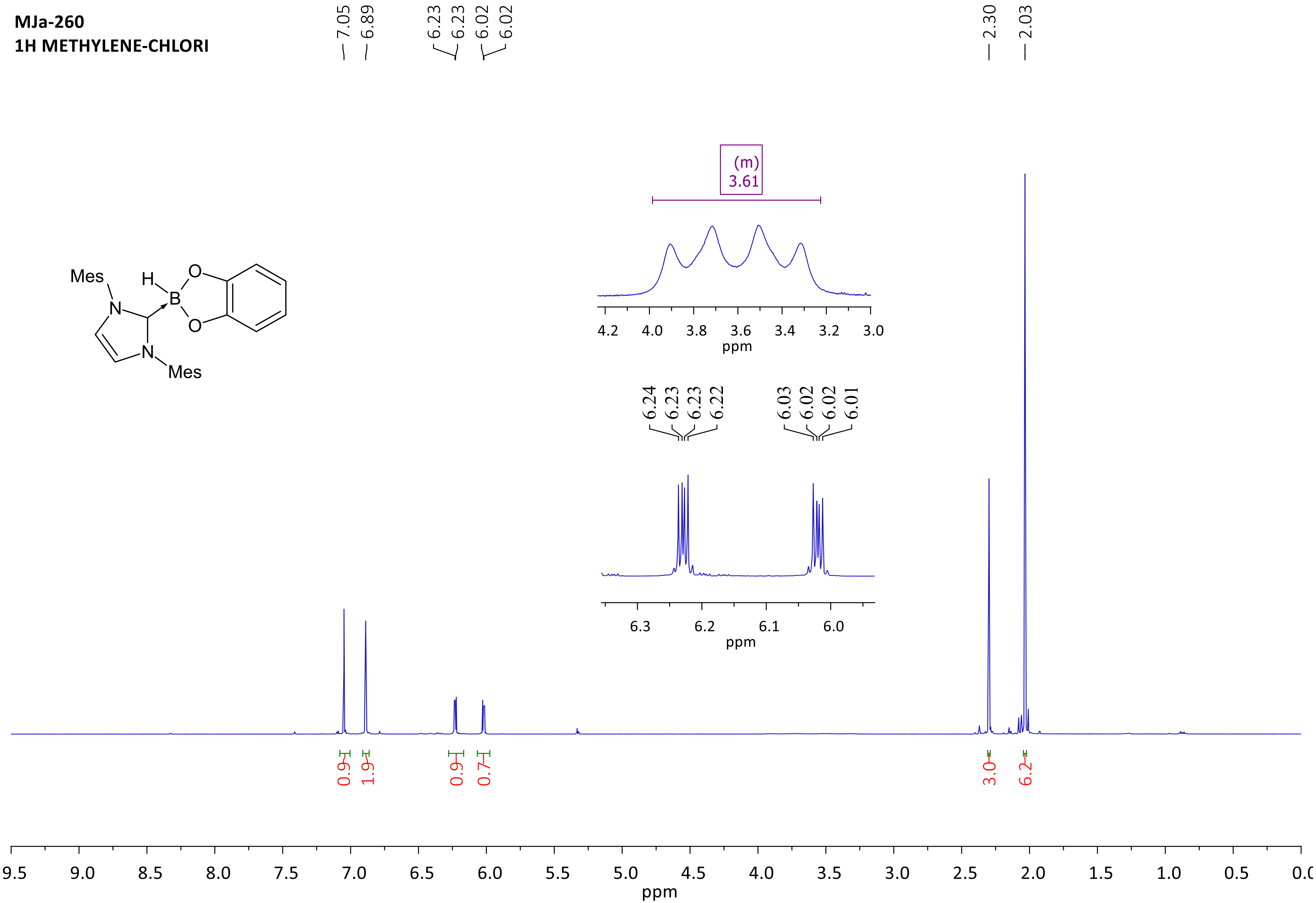

^{1}H NMR for compound ***1b*H**.

MJa-260
11B METHYLENE-CHLORI

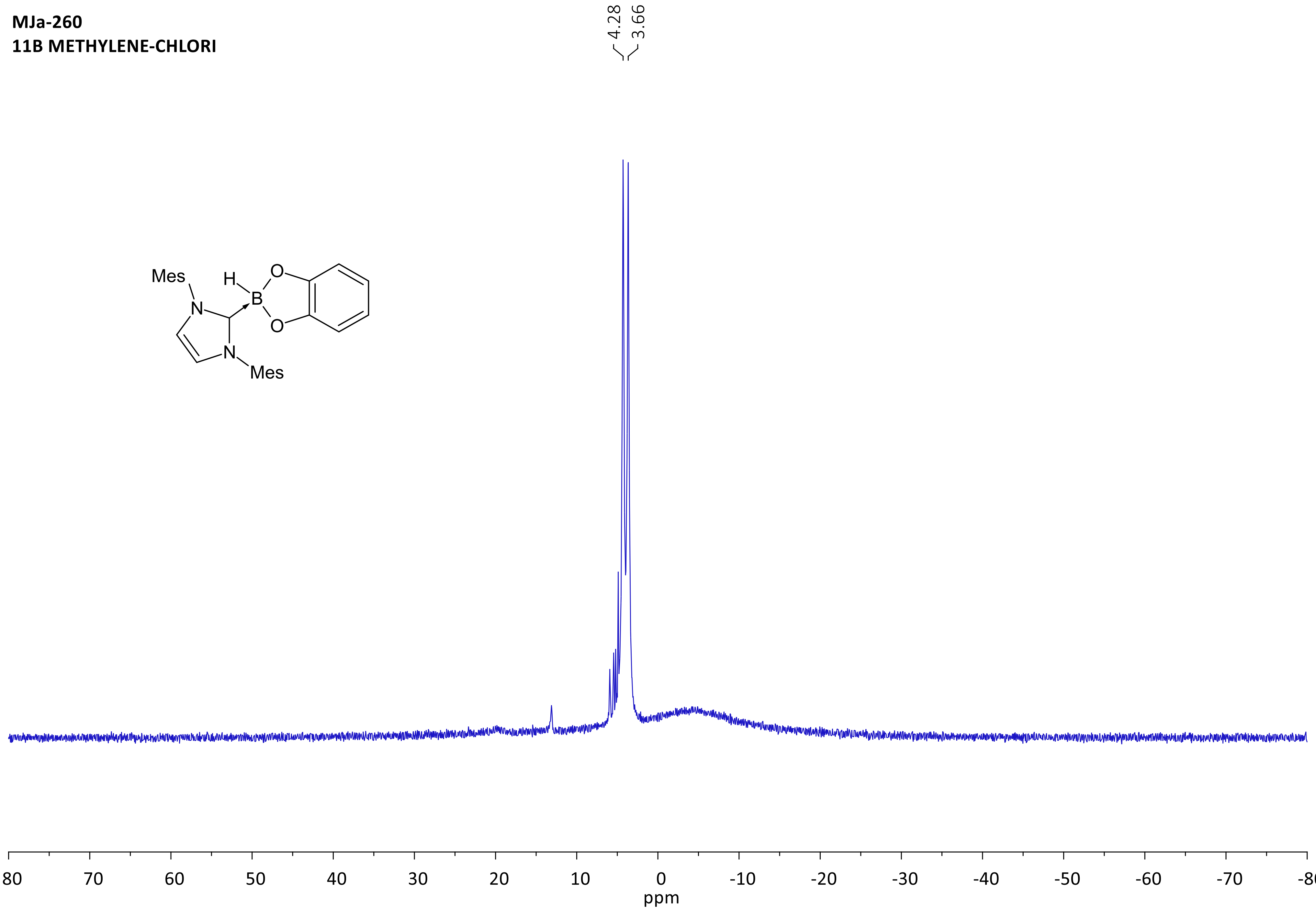

^{11}B NMR for compound ***1b*H**.

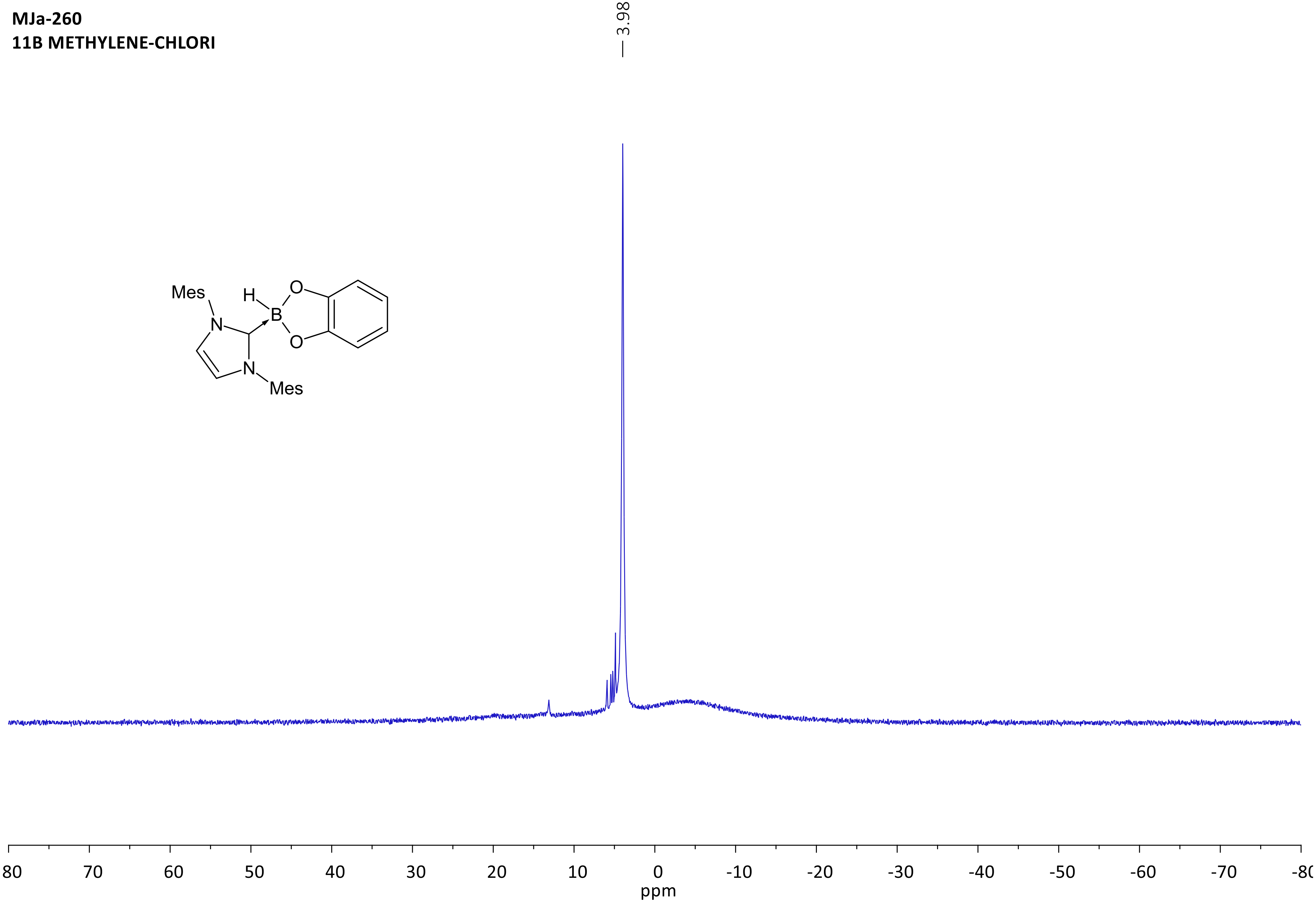

$^{11}B\{^{1}H\}$ NMR for compound ***1b*H**.

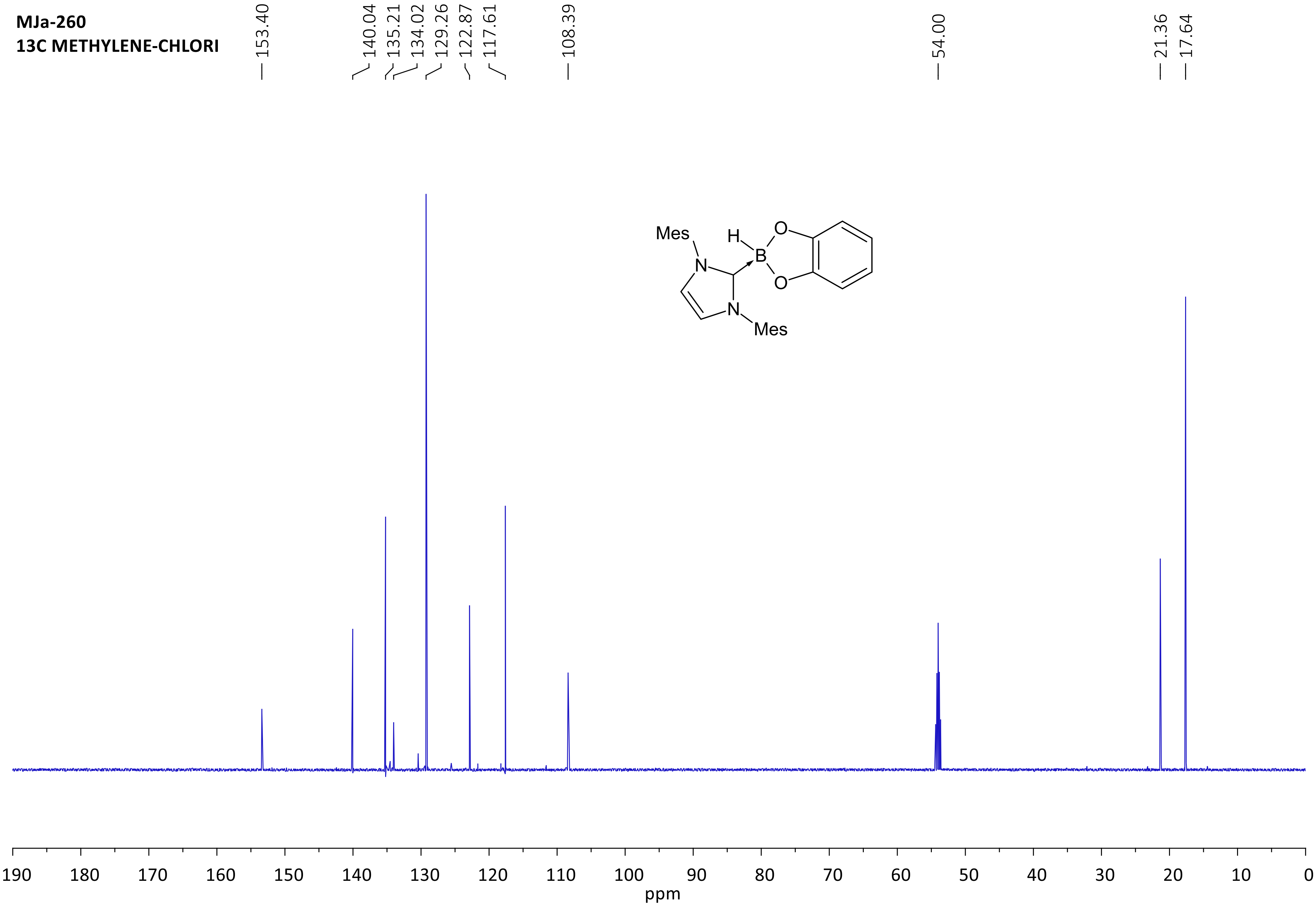

$^{13}C\{^{1}H\}$ NMR for compound ***1b*H**.

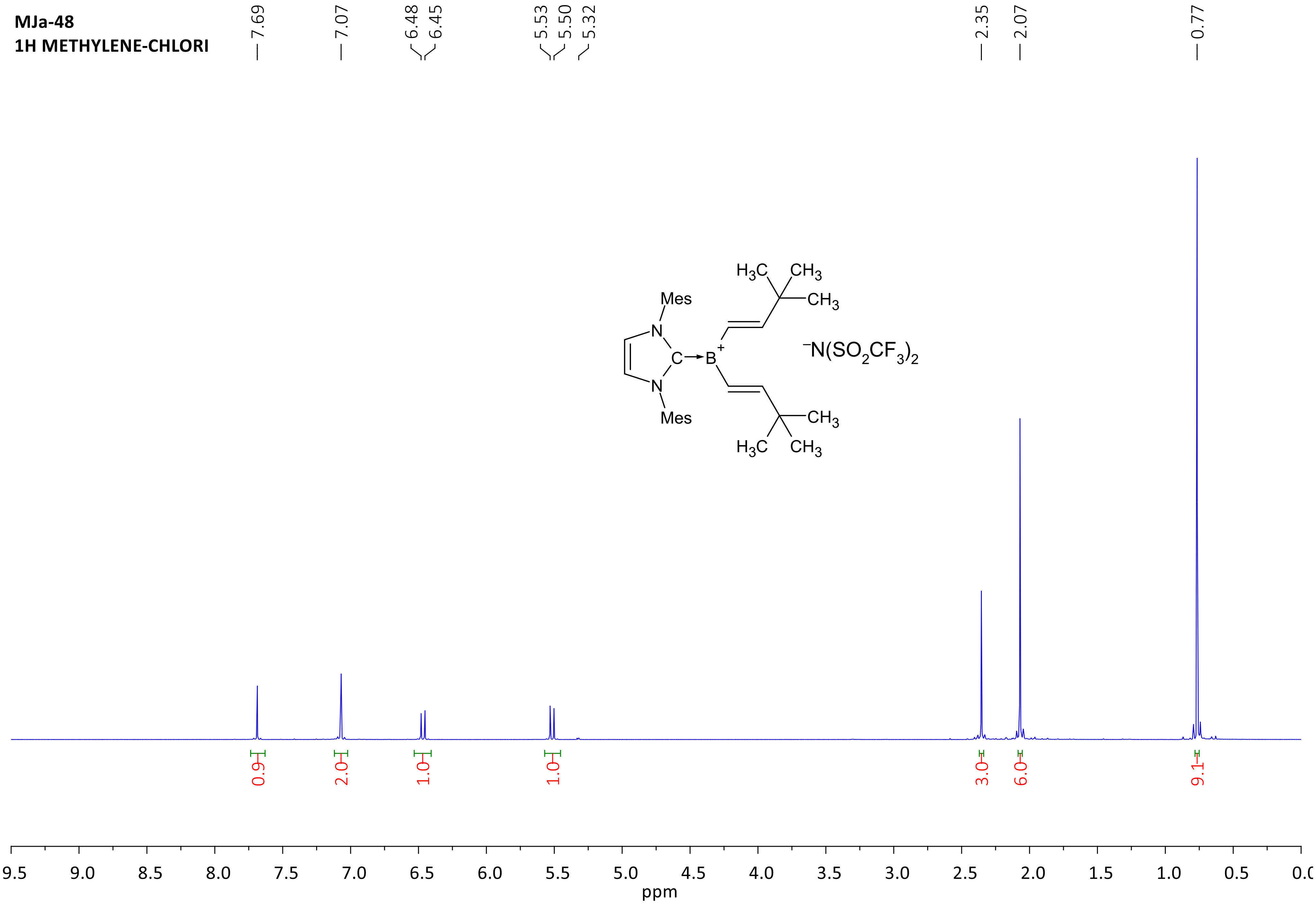

^{1}H NMR for compound ***1c***.

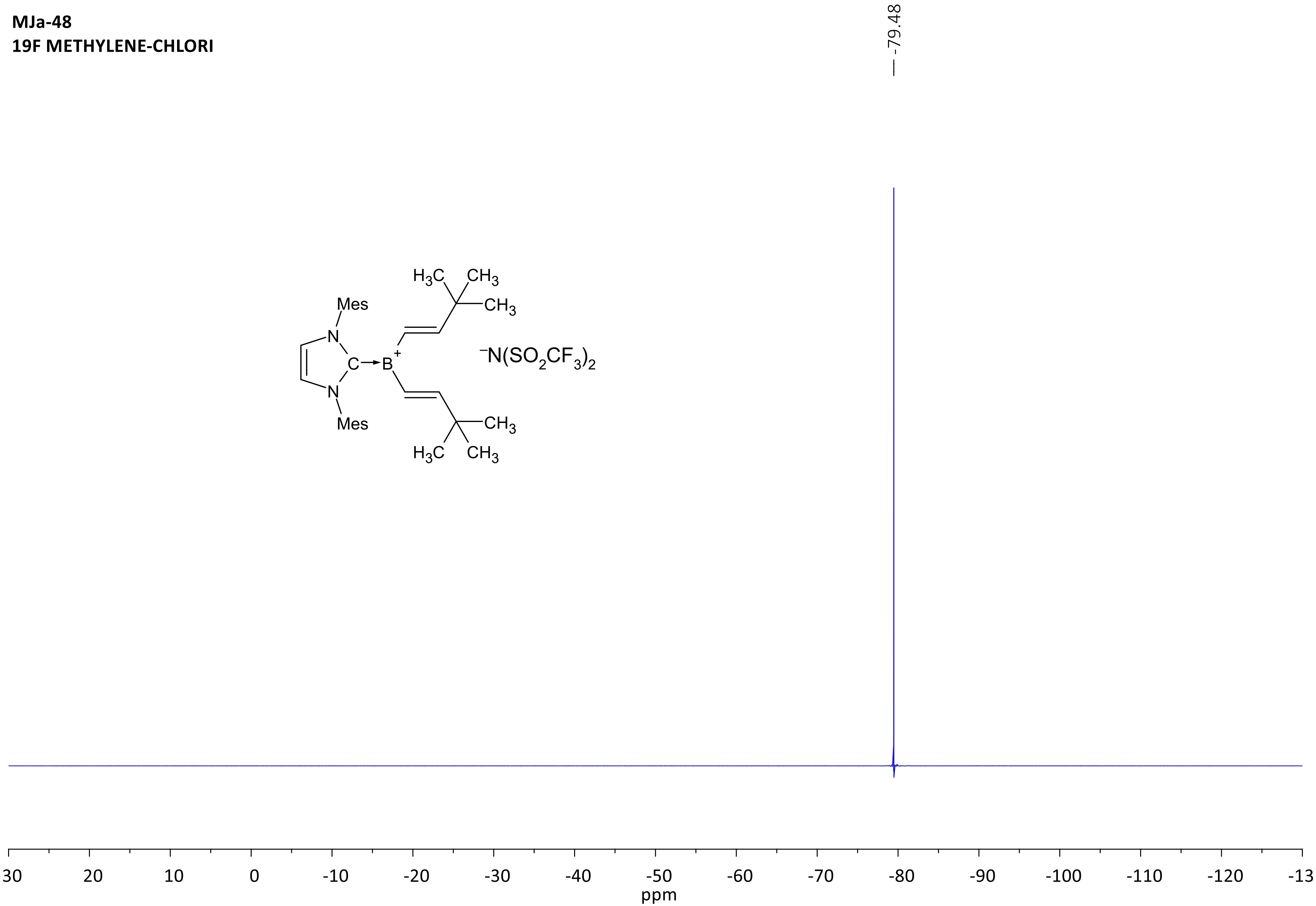

^{19}F NMR for compound ***1c***.

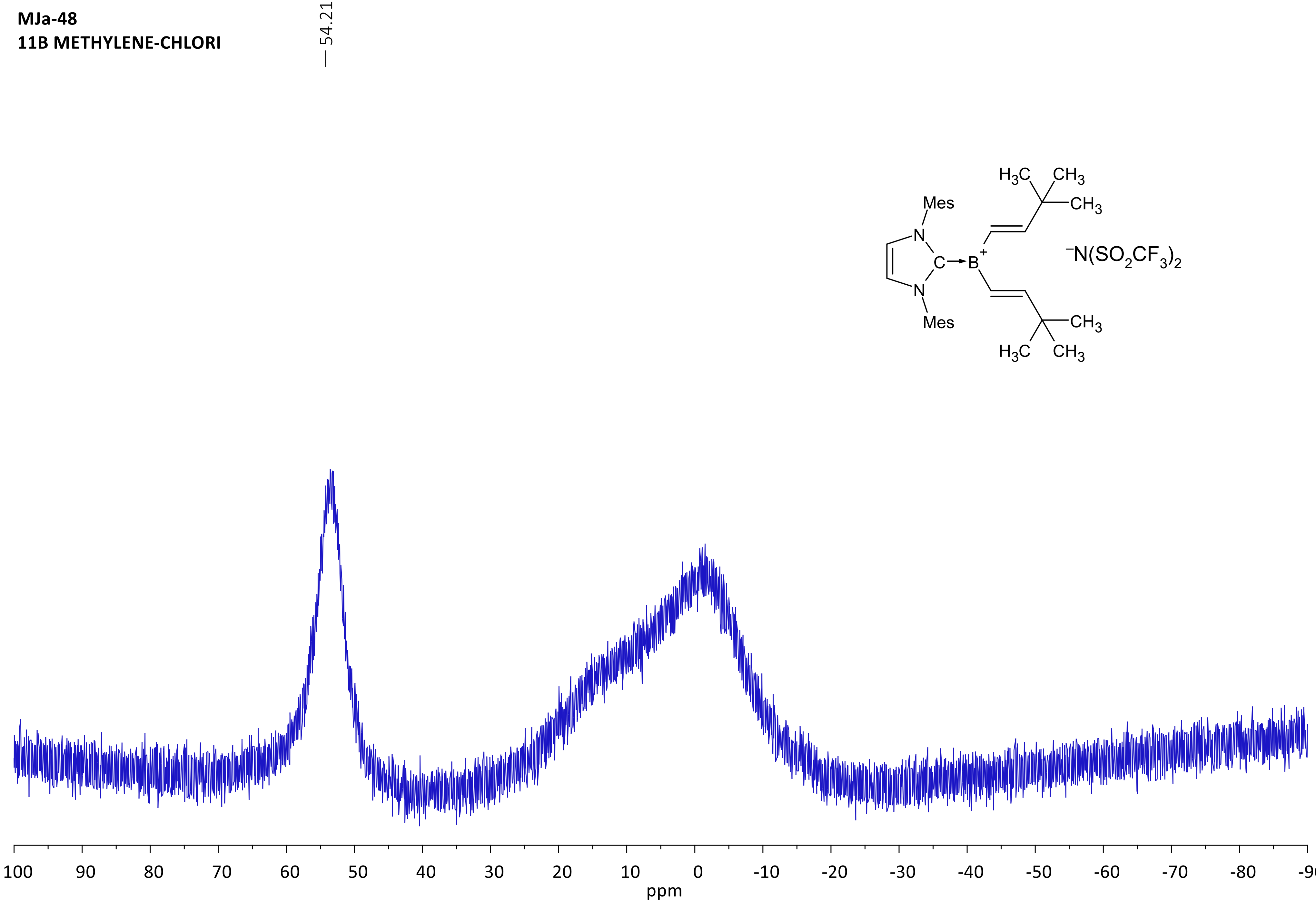

$^{11}B\{^1H\}$ NMR for compound ***1c***.

MJa-48
13C

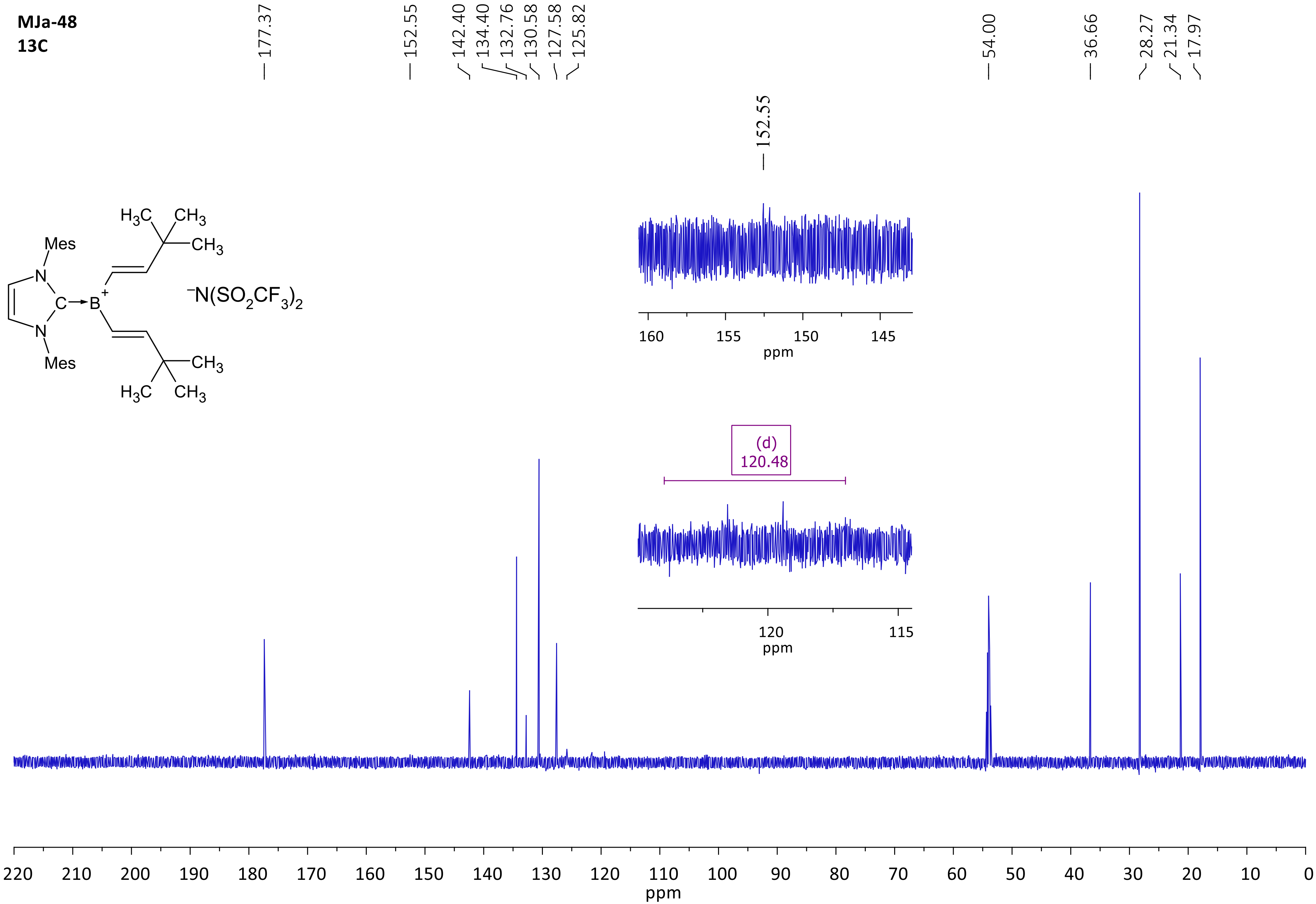

$^{13}C\{^1H\}$ NMR for compound ***1c***.

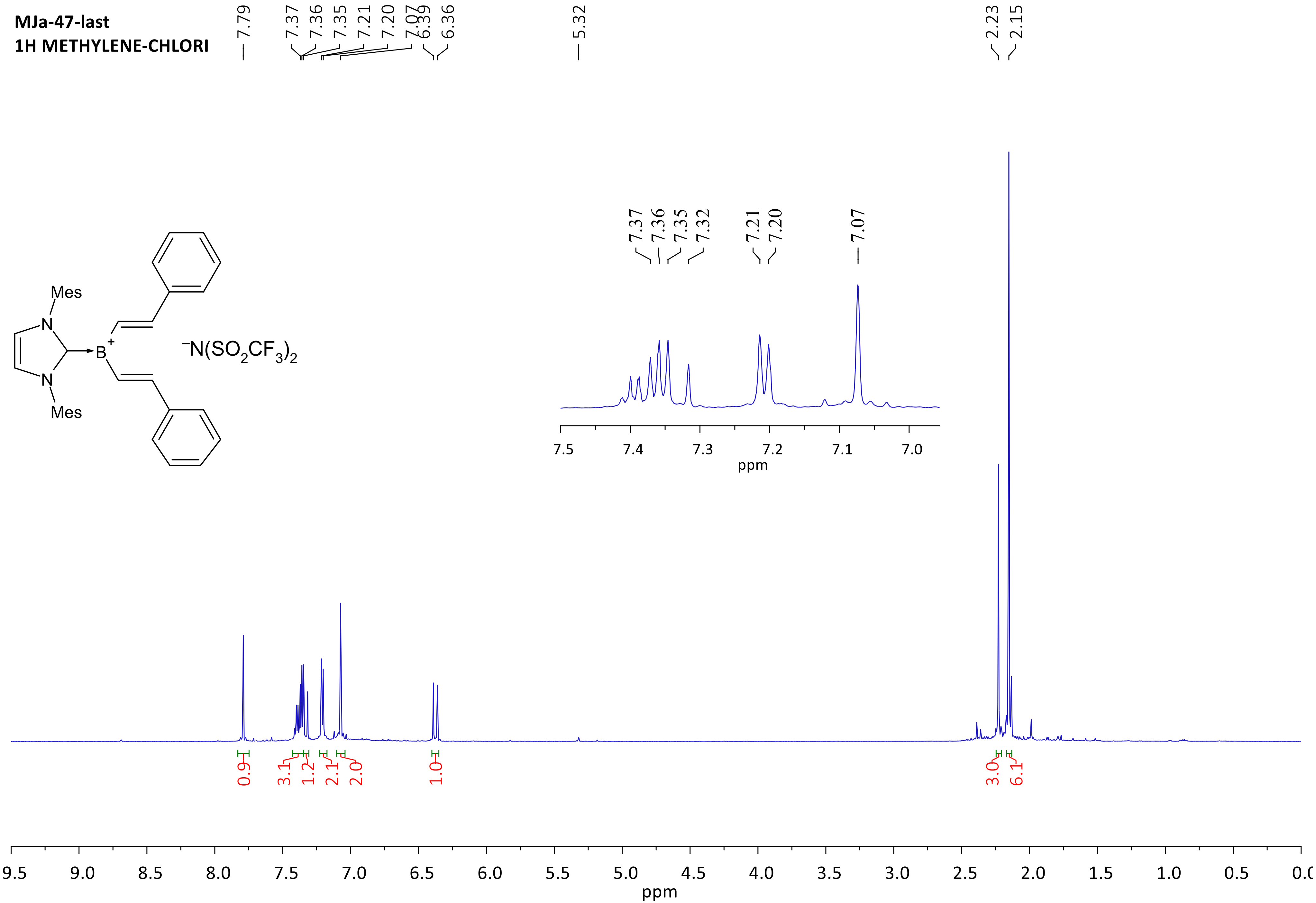

^{1}H NMR for compound ***1d***.

MJa-47-powder
19F METHYLENE-CHLORI

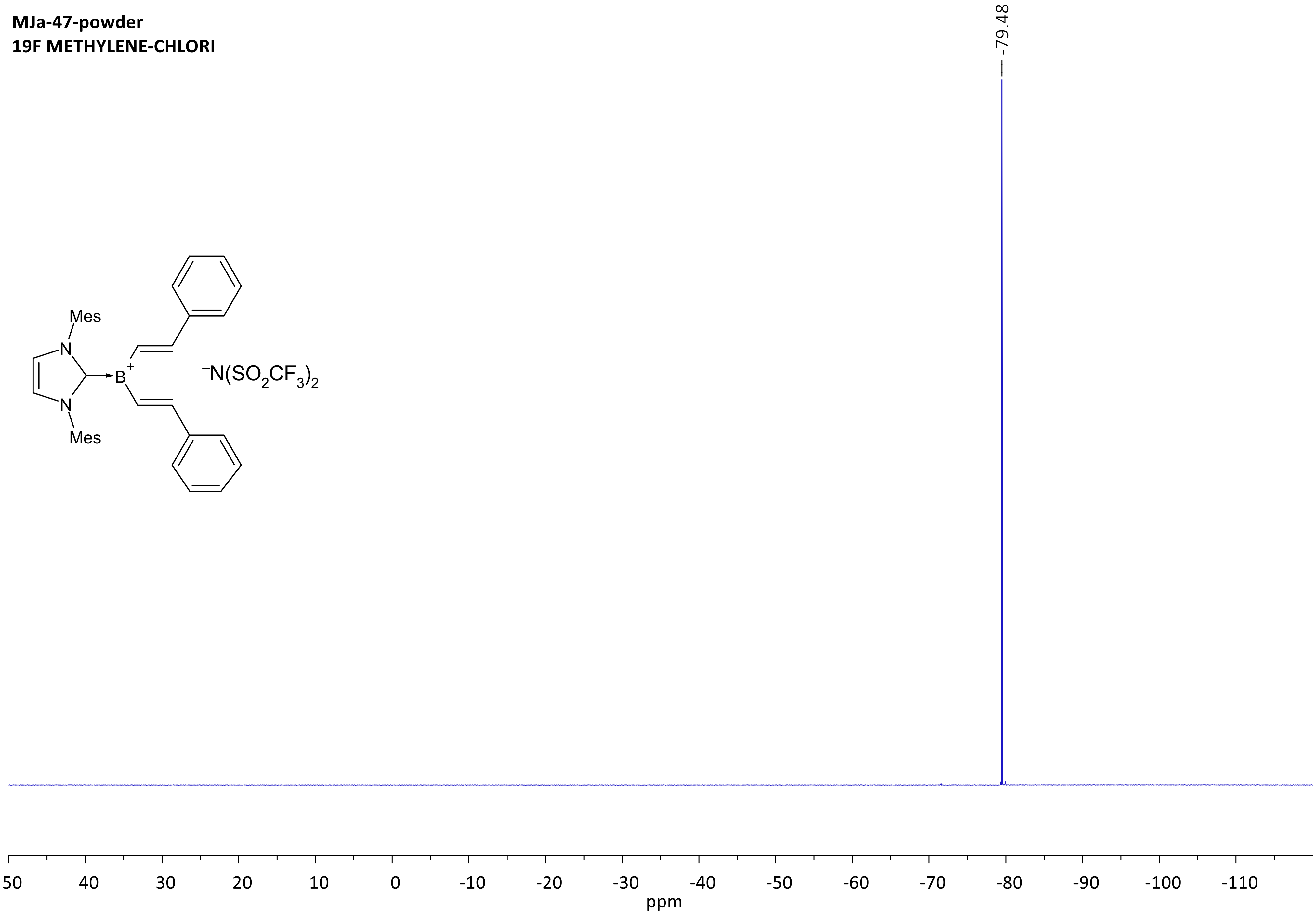

^{19}F NMR for compound ***1d***.

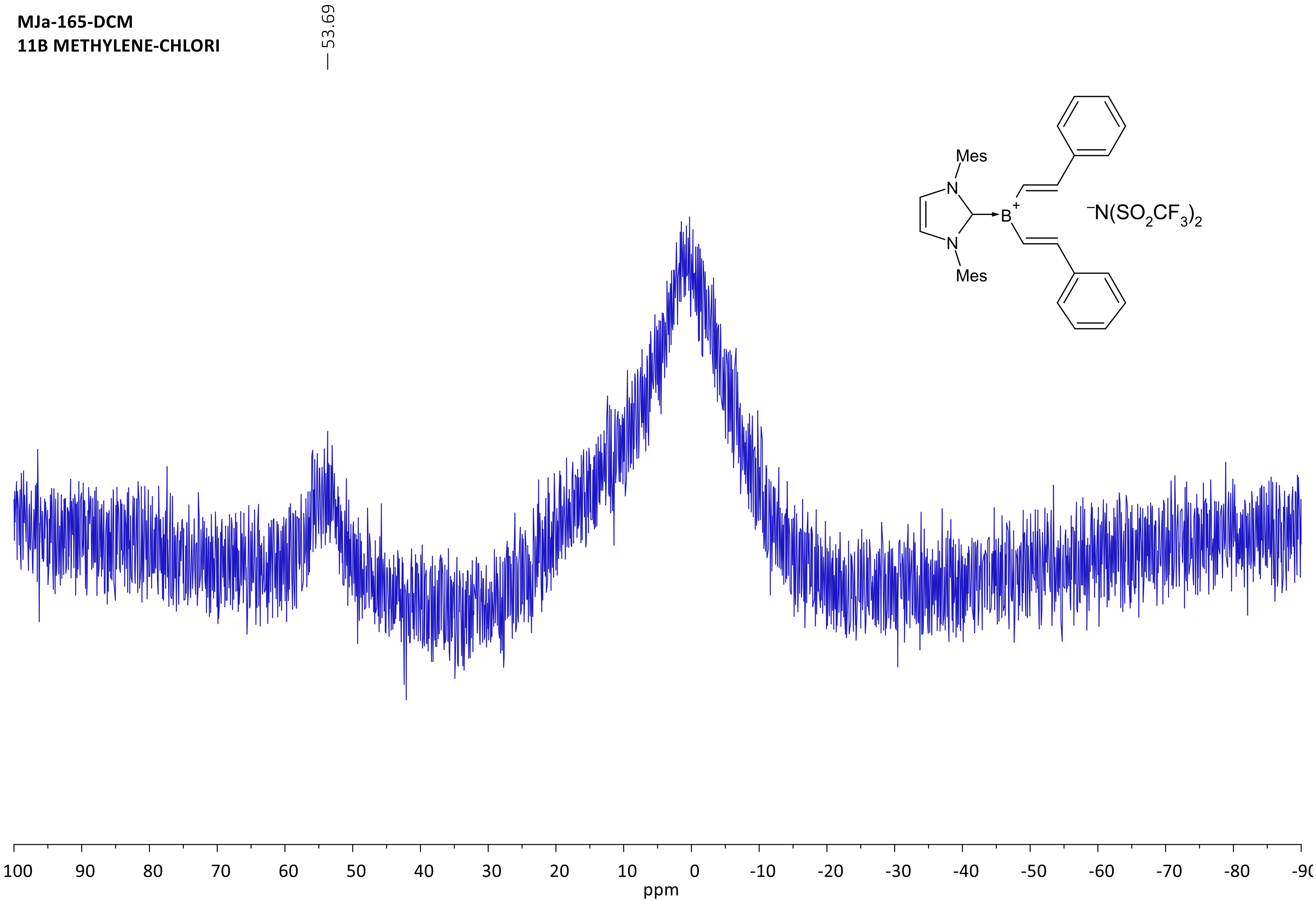

$^{11}B\{^{1}H\}$ NMR for compound ***1d***.

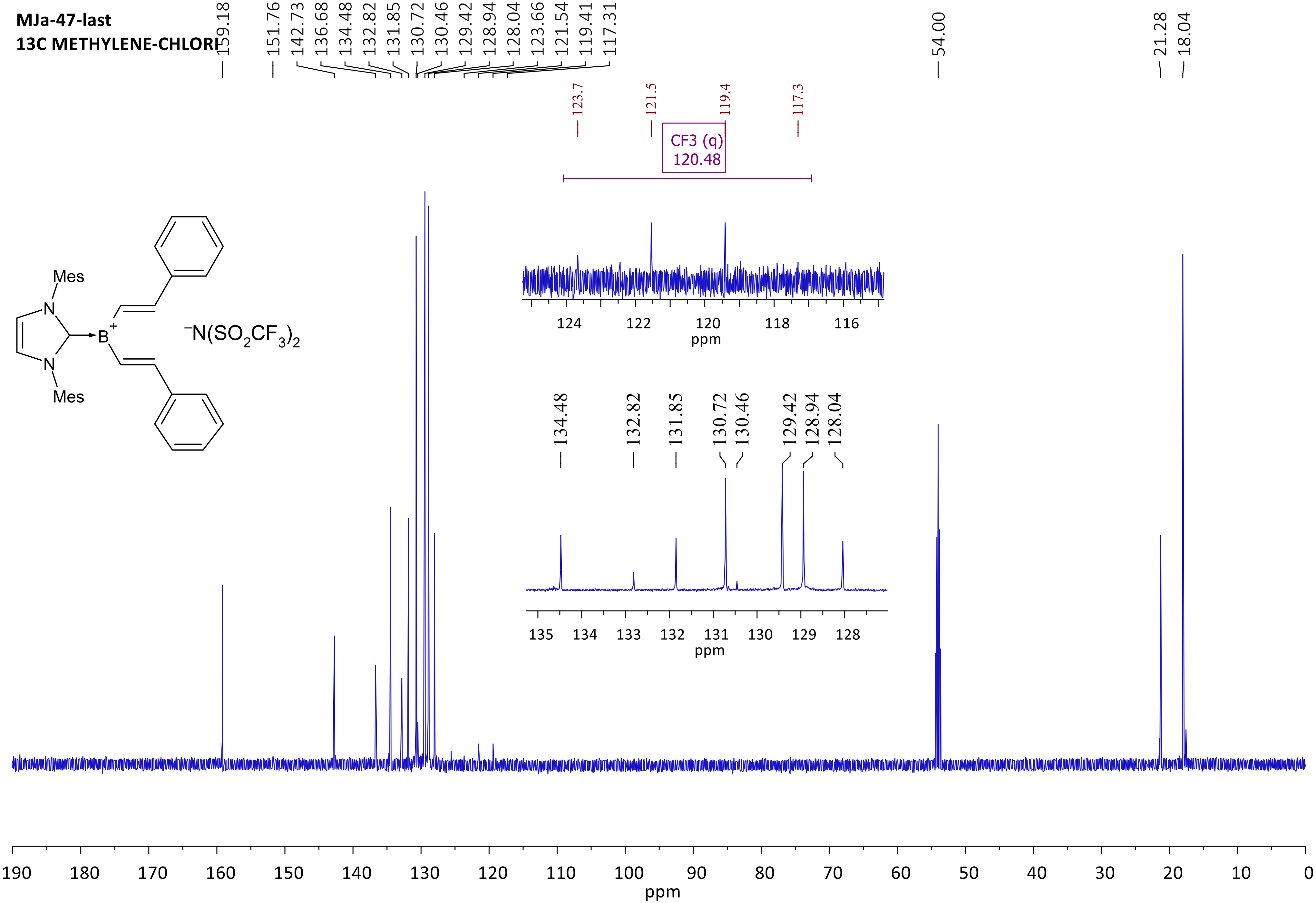

$^{13}C\{^1H\}$ NMR for compound ***1d***.

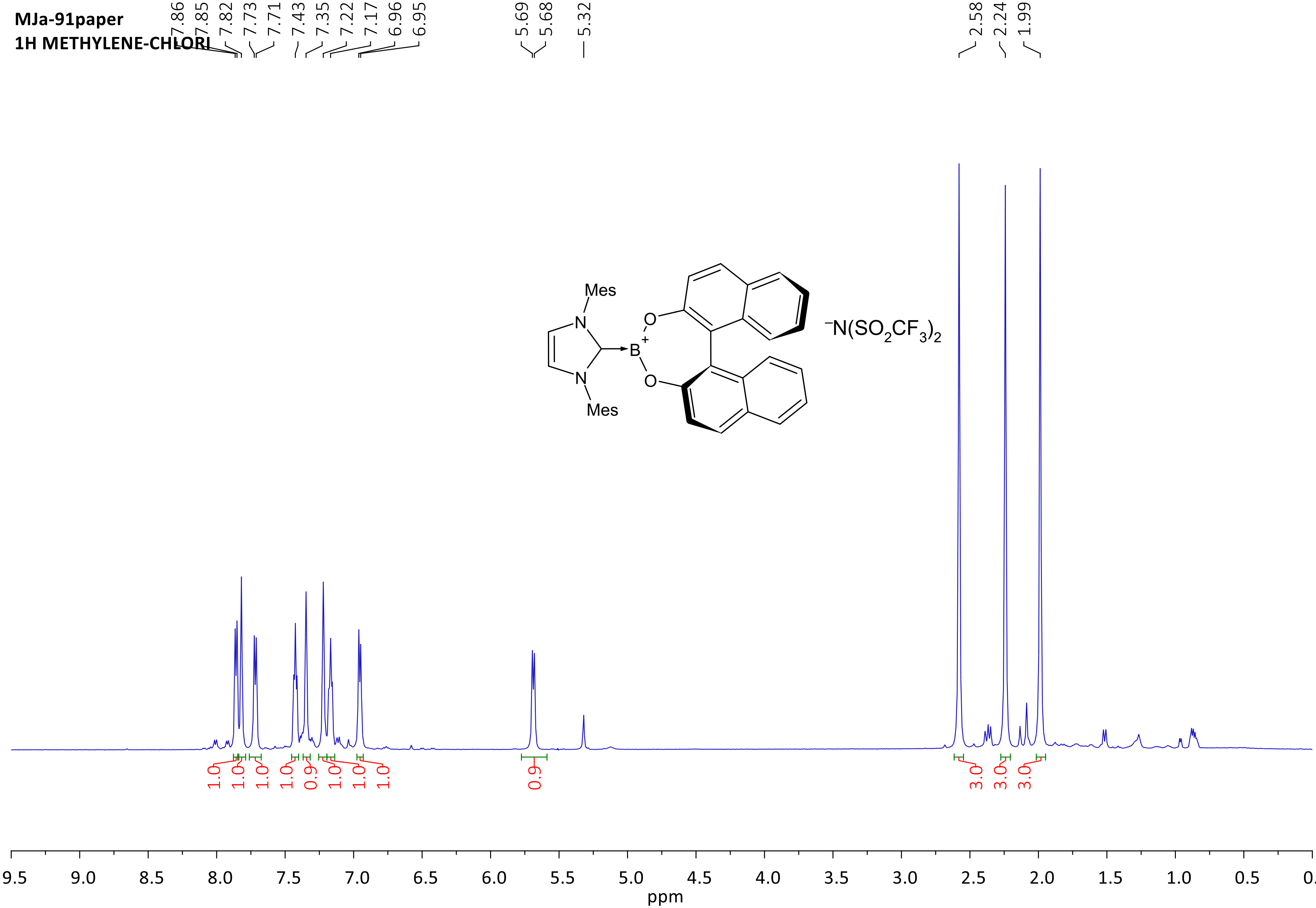

^{1}H NMR for compound ***1e***.

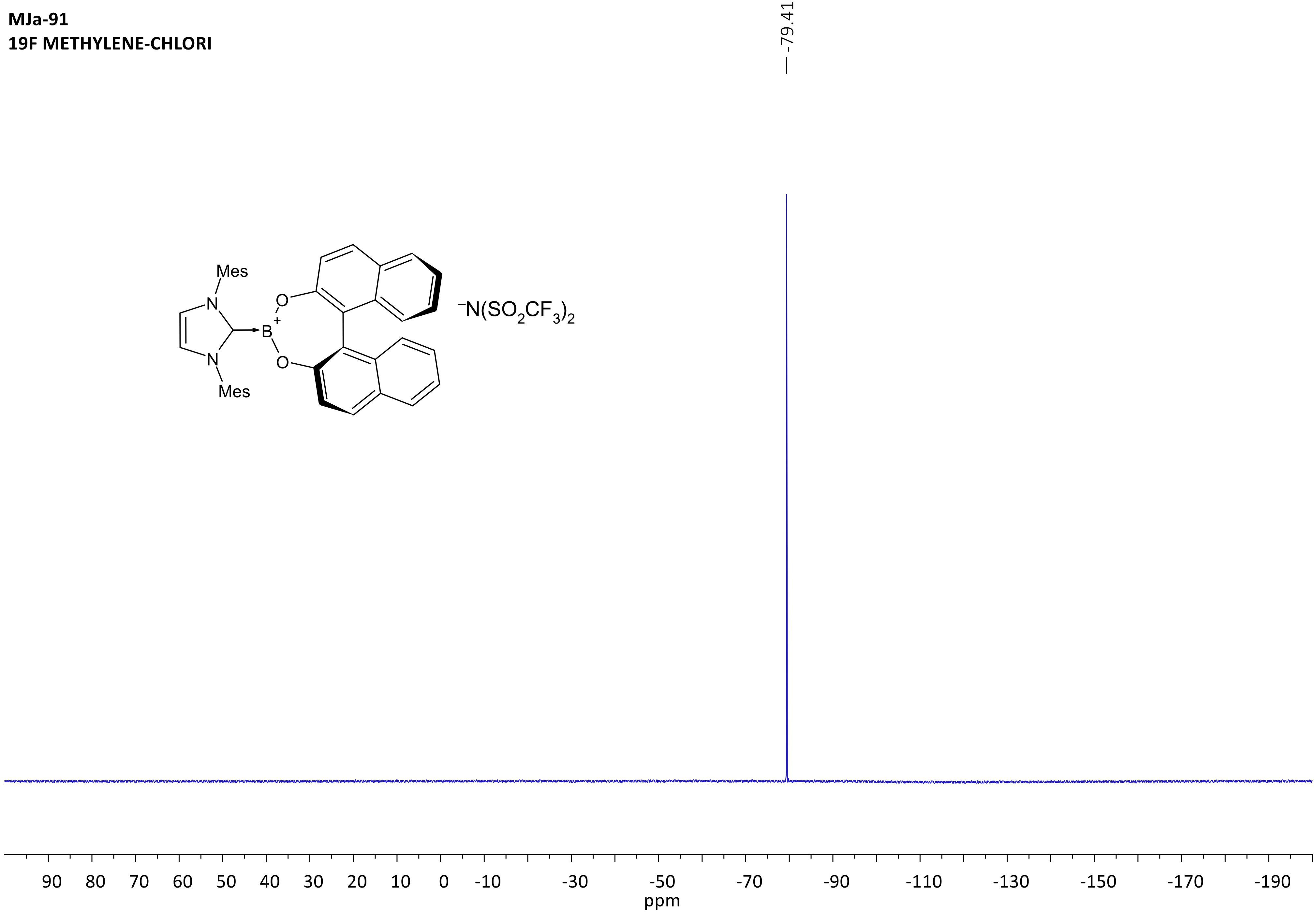

^{19}F NMR for compound ***1e***.

MJa-91paper
11B METHYLENE-CHLORI

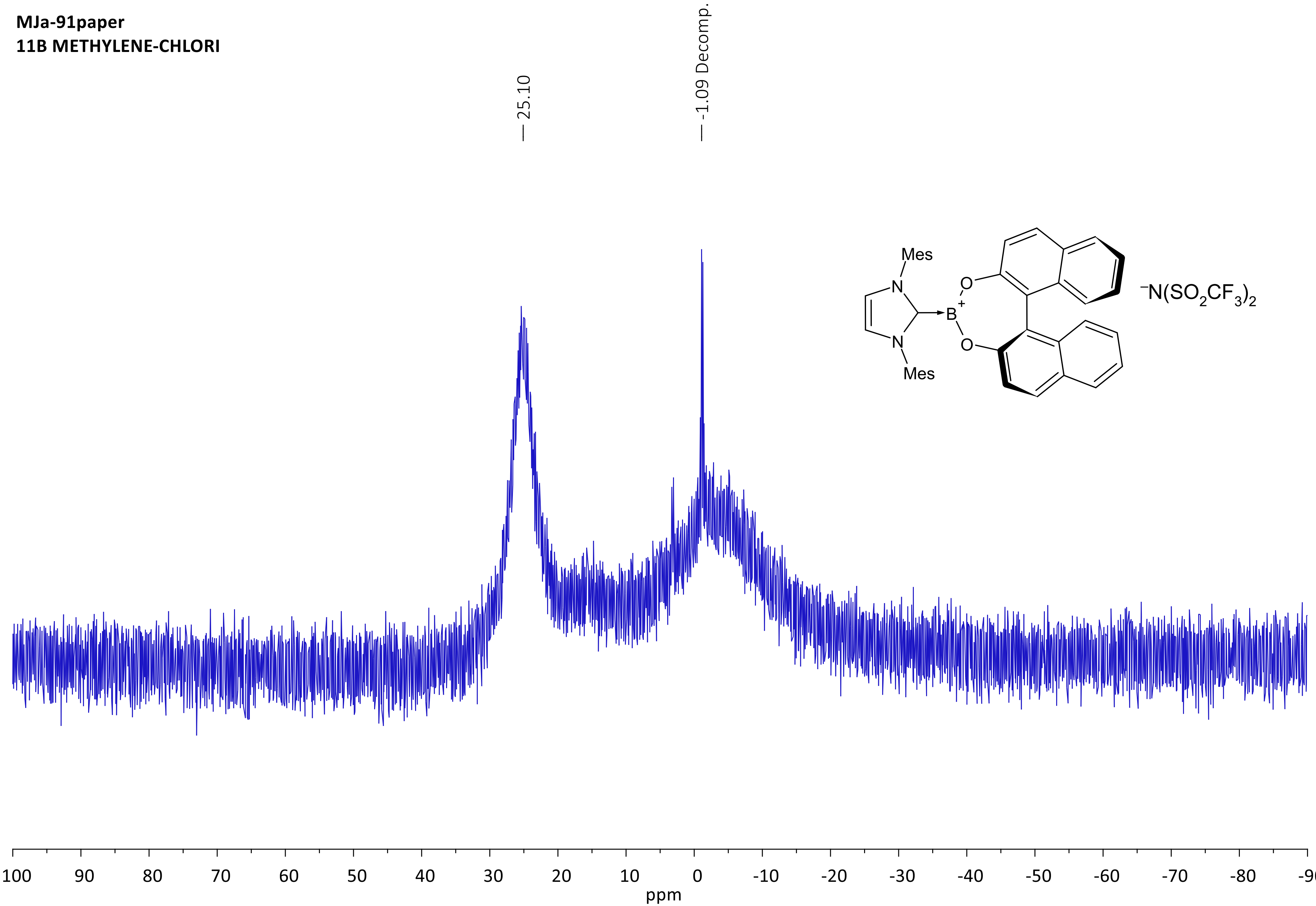

$^{11}B\{^{1}H\}$ NMR for compound ***1e***.

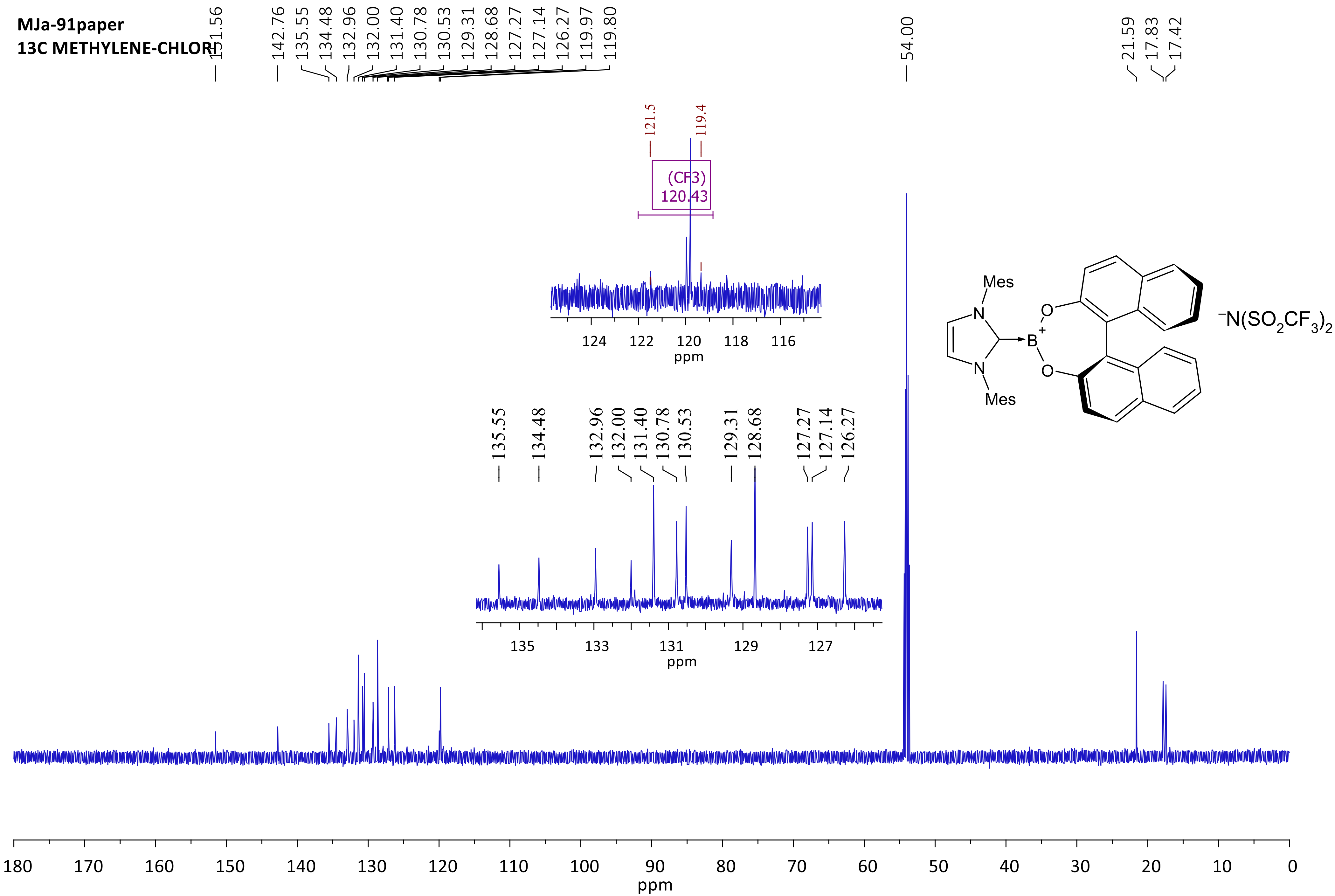

$^{13}C\{^1H\}$ NMR for compound ***1e***.

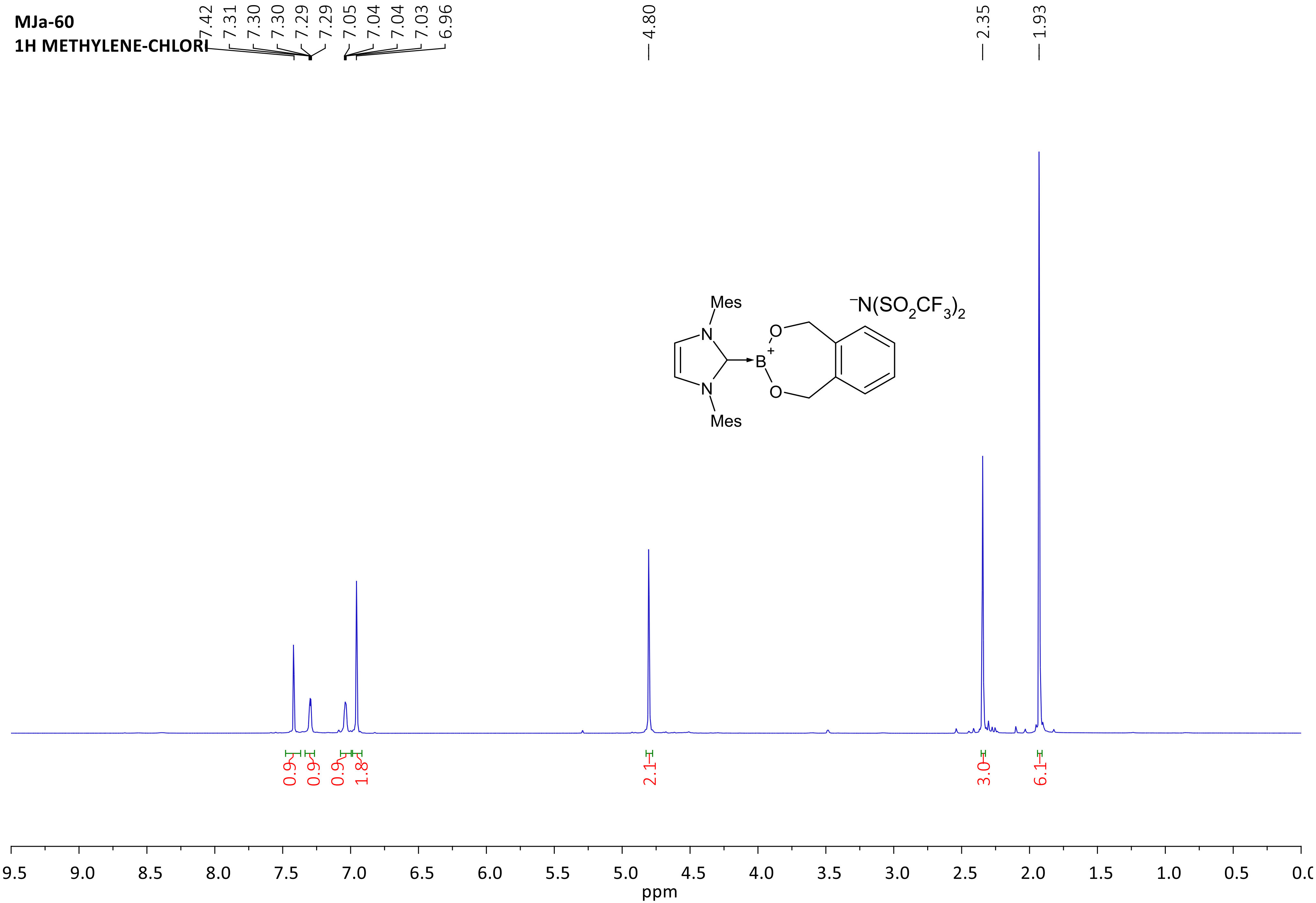

^{1}H NMR for compound ***1f***.

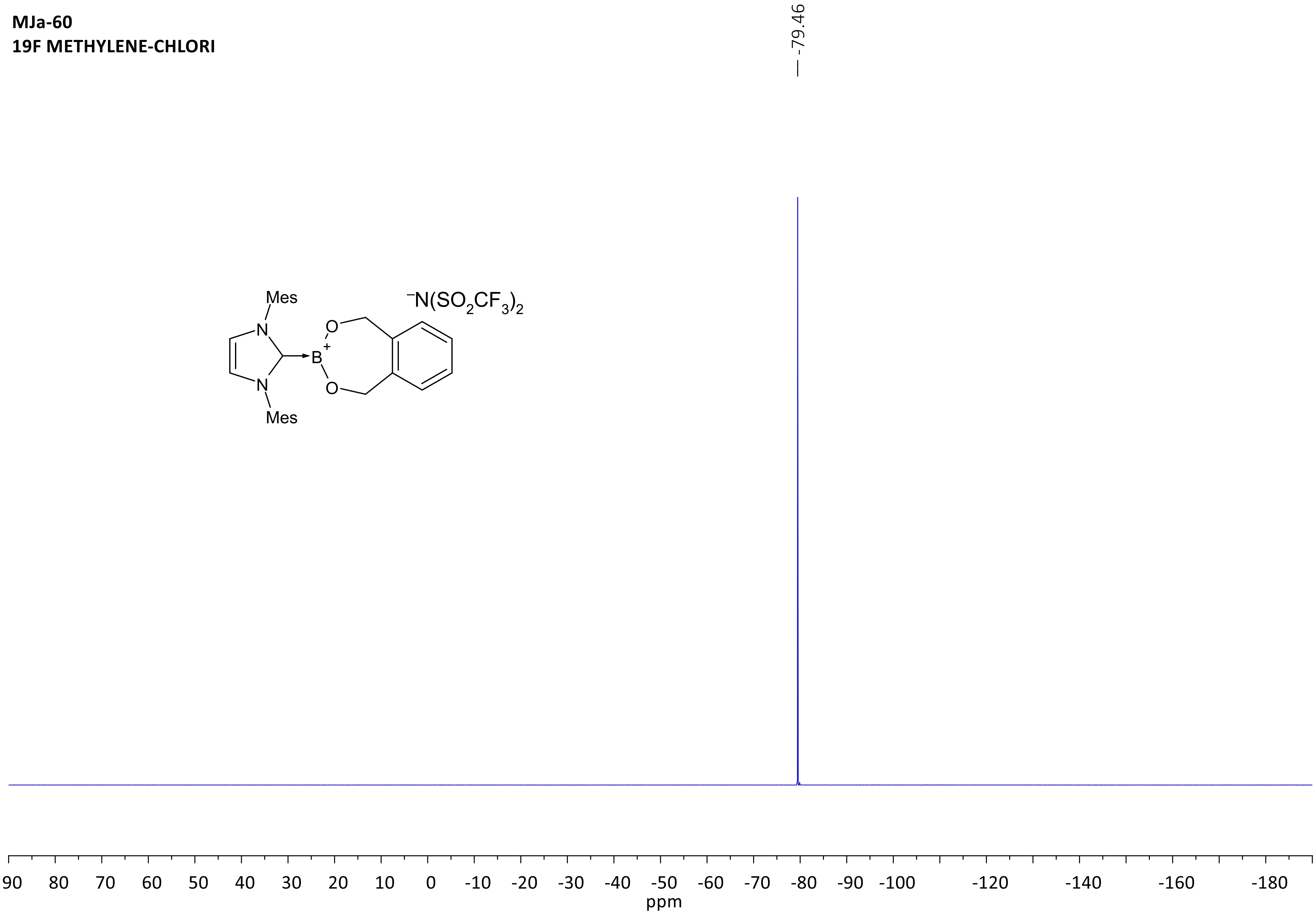

^{19}F NMR for compound ***1f***.

MJa-60
11B METHYLENE-CHLORI

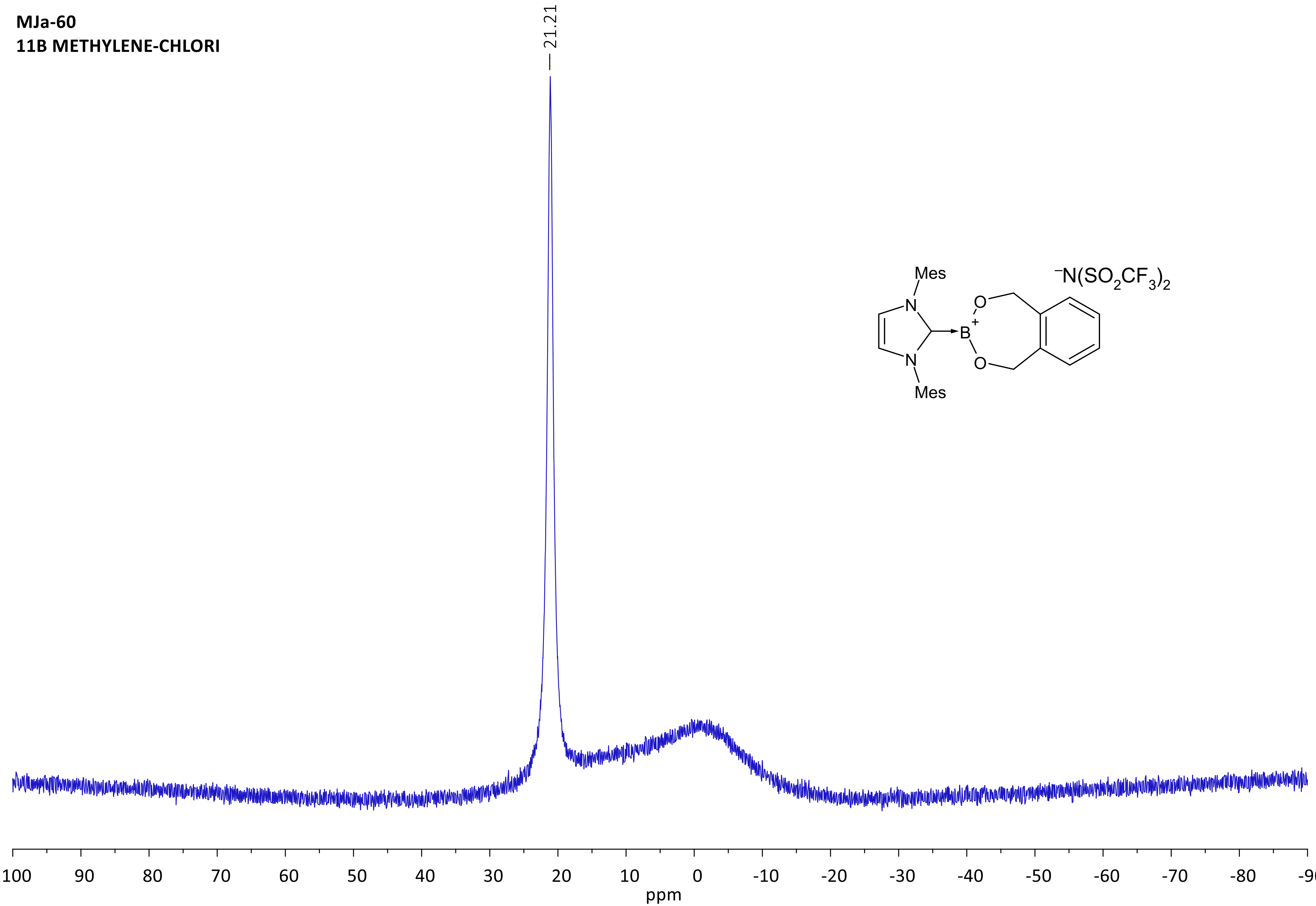

$^{11}B\{^1H\}$ NMR for compound ***1f***.

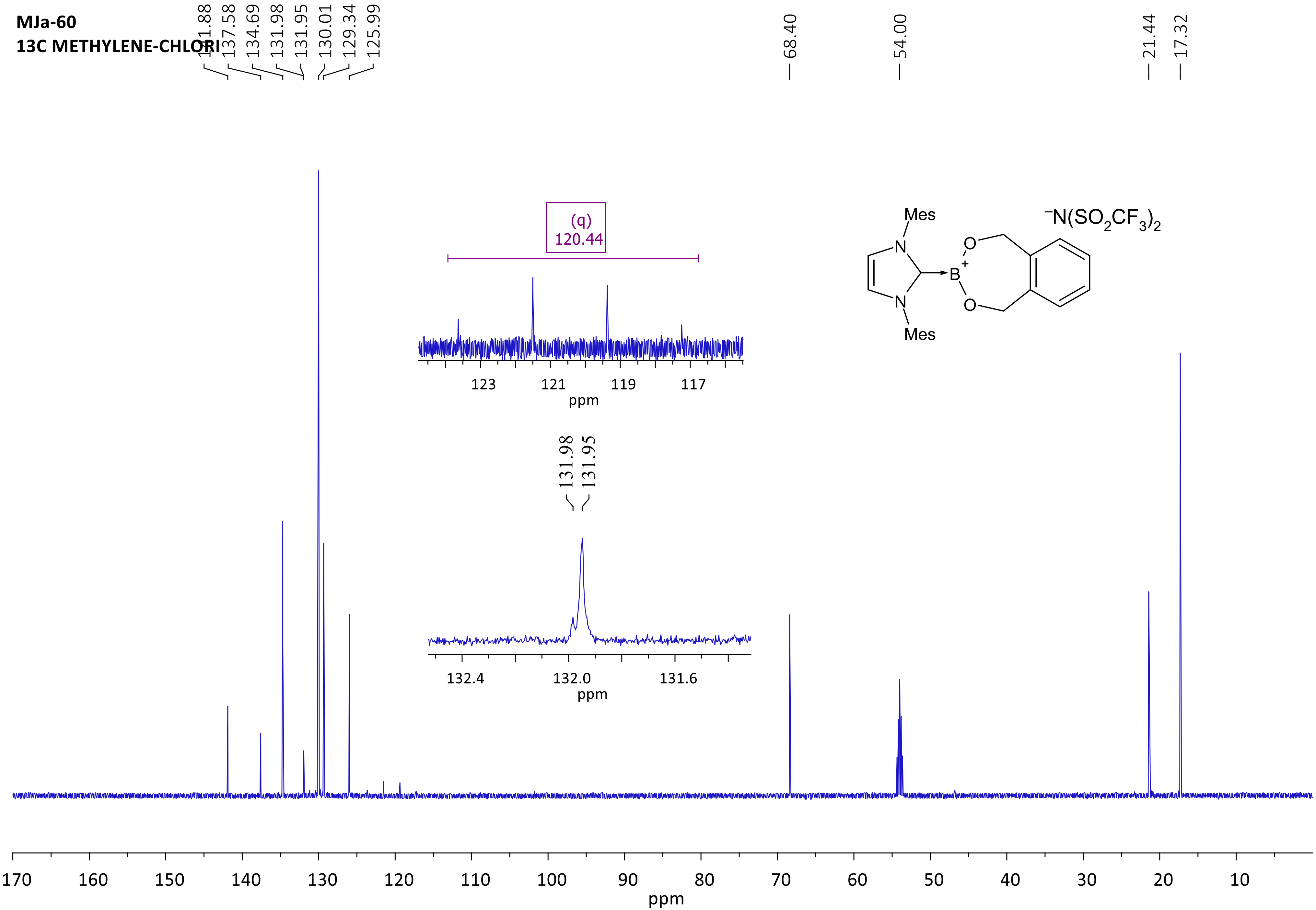

$^{13}C\{^{1}H\}$ NMR for compound ***1f***.

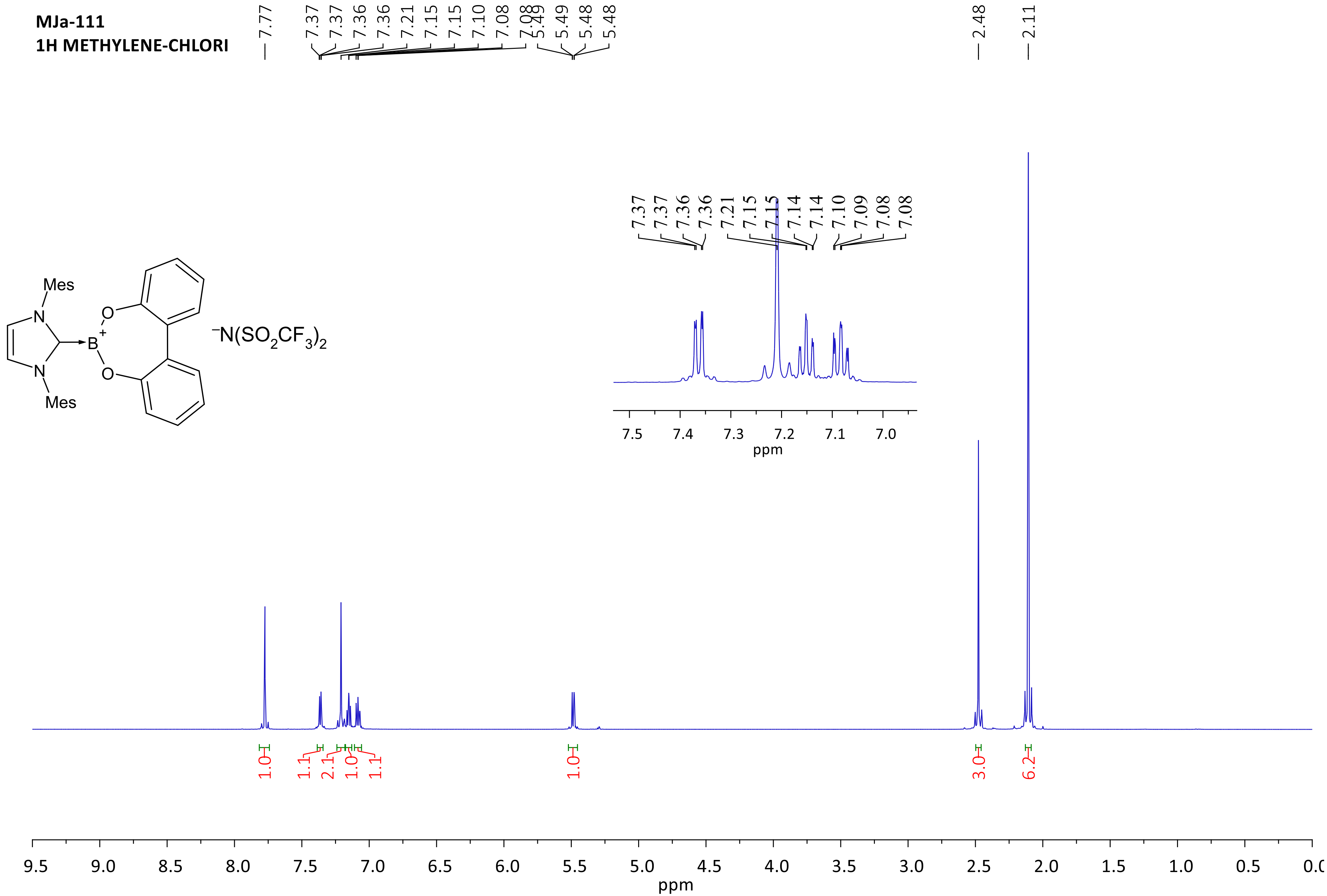

^{1}H NMR for compound ***1g***.

MJa-111
19F METHYLENE-CHLORI

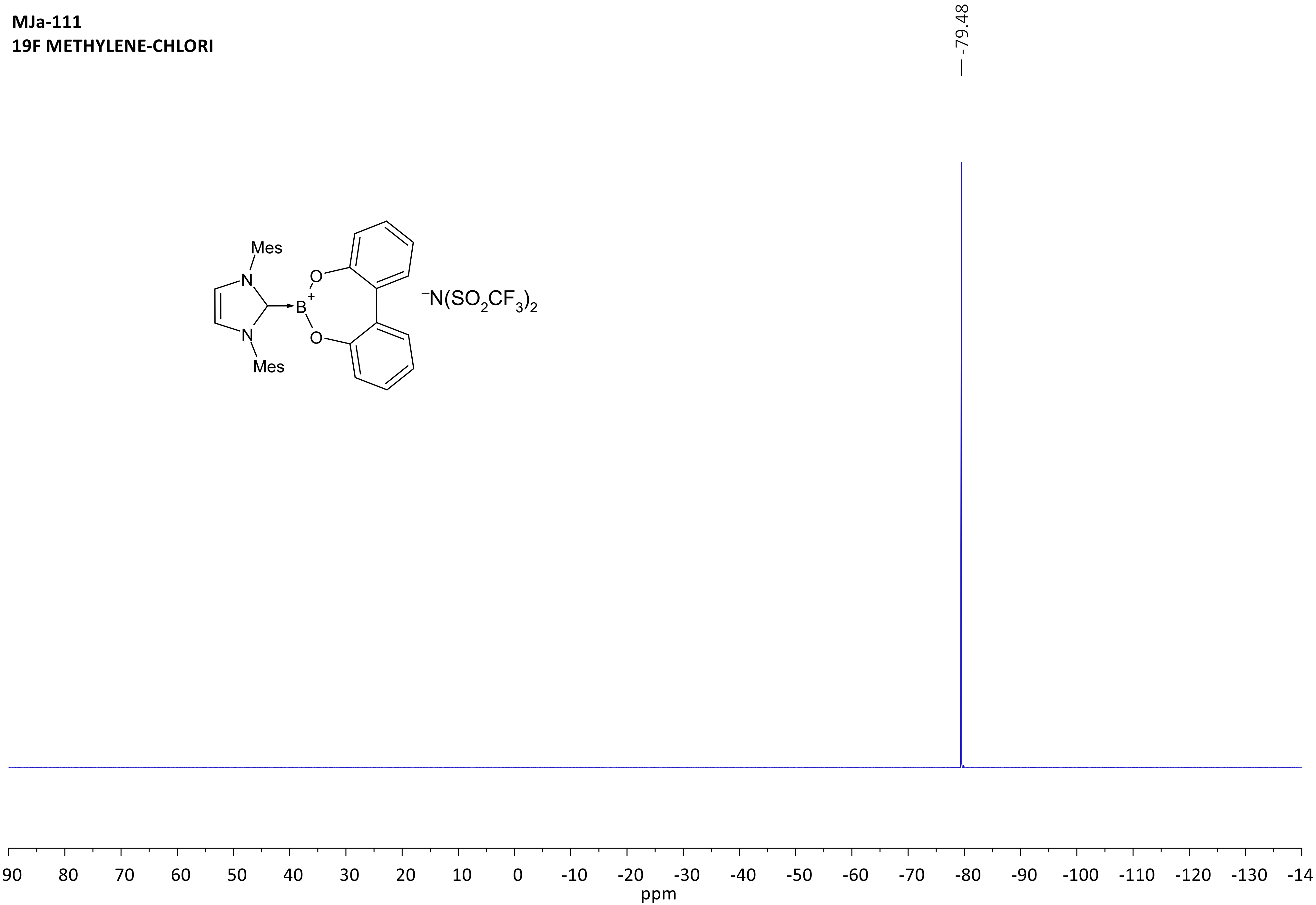

^{19}F NMR for compound ***1g***.

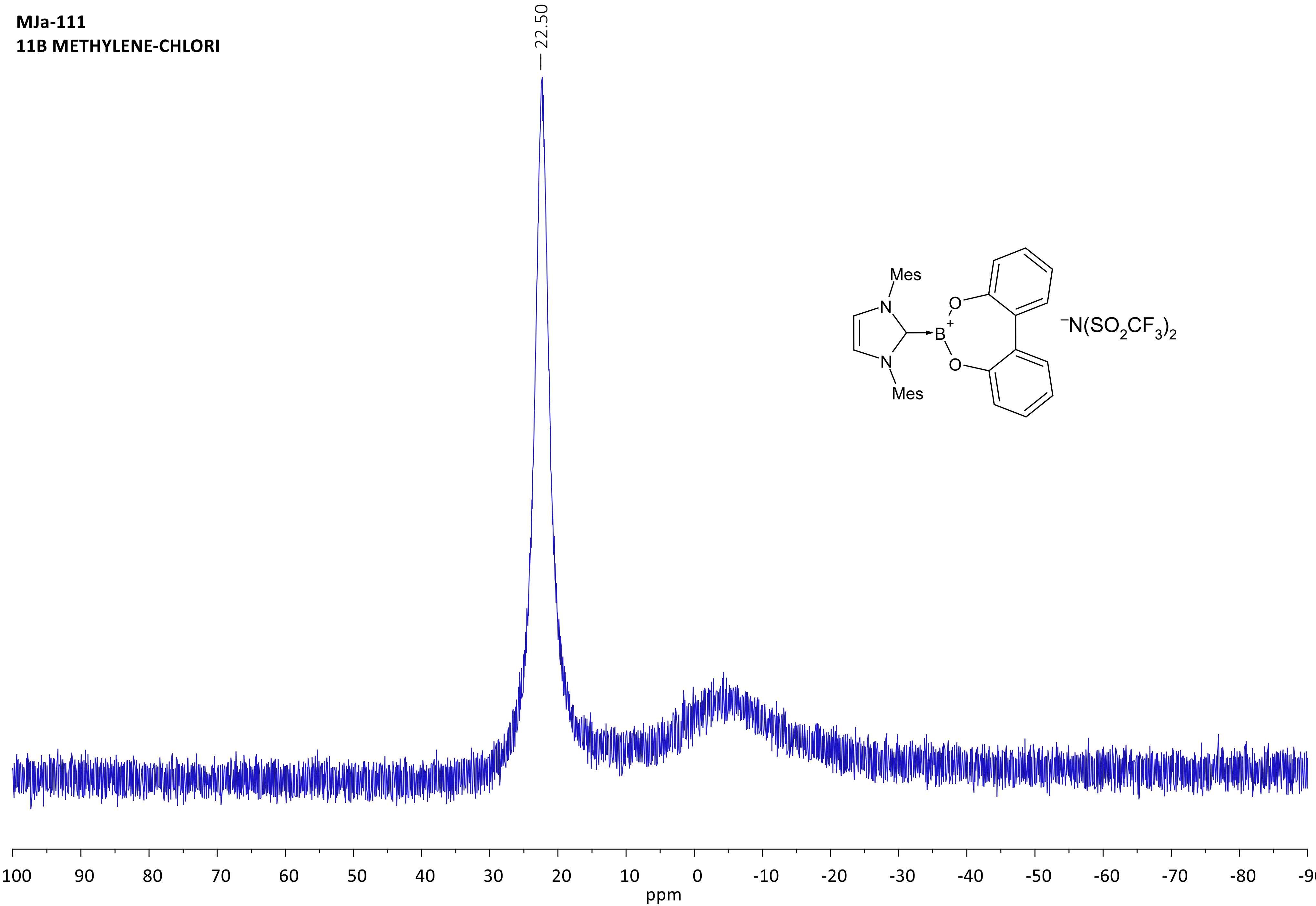

$^{11}B\{^{1}H\}$ NMR for compound ***1g***.

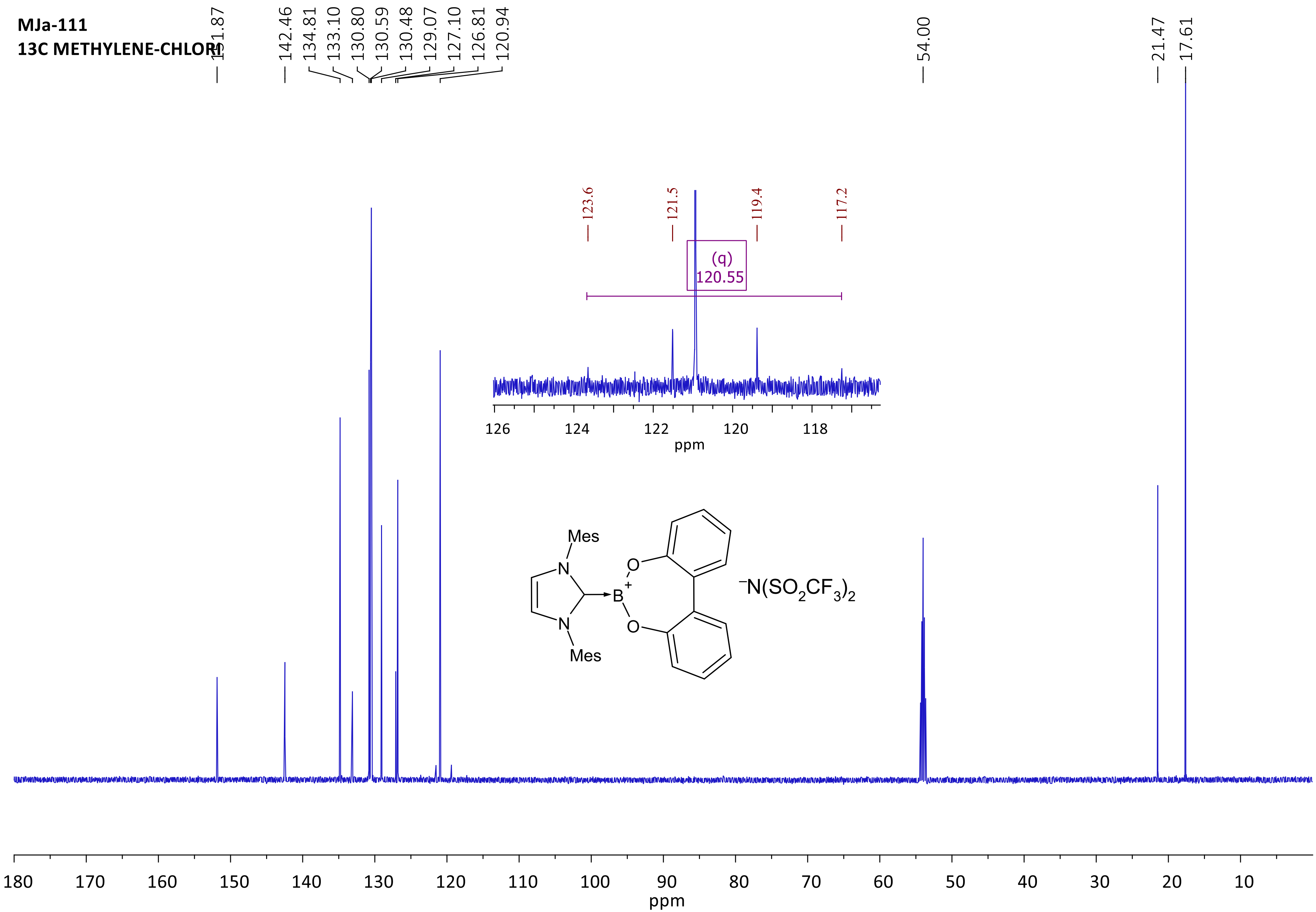

$^{13}C\{^1H\}$ NMR for compound ***1g***.

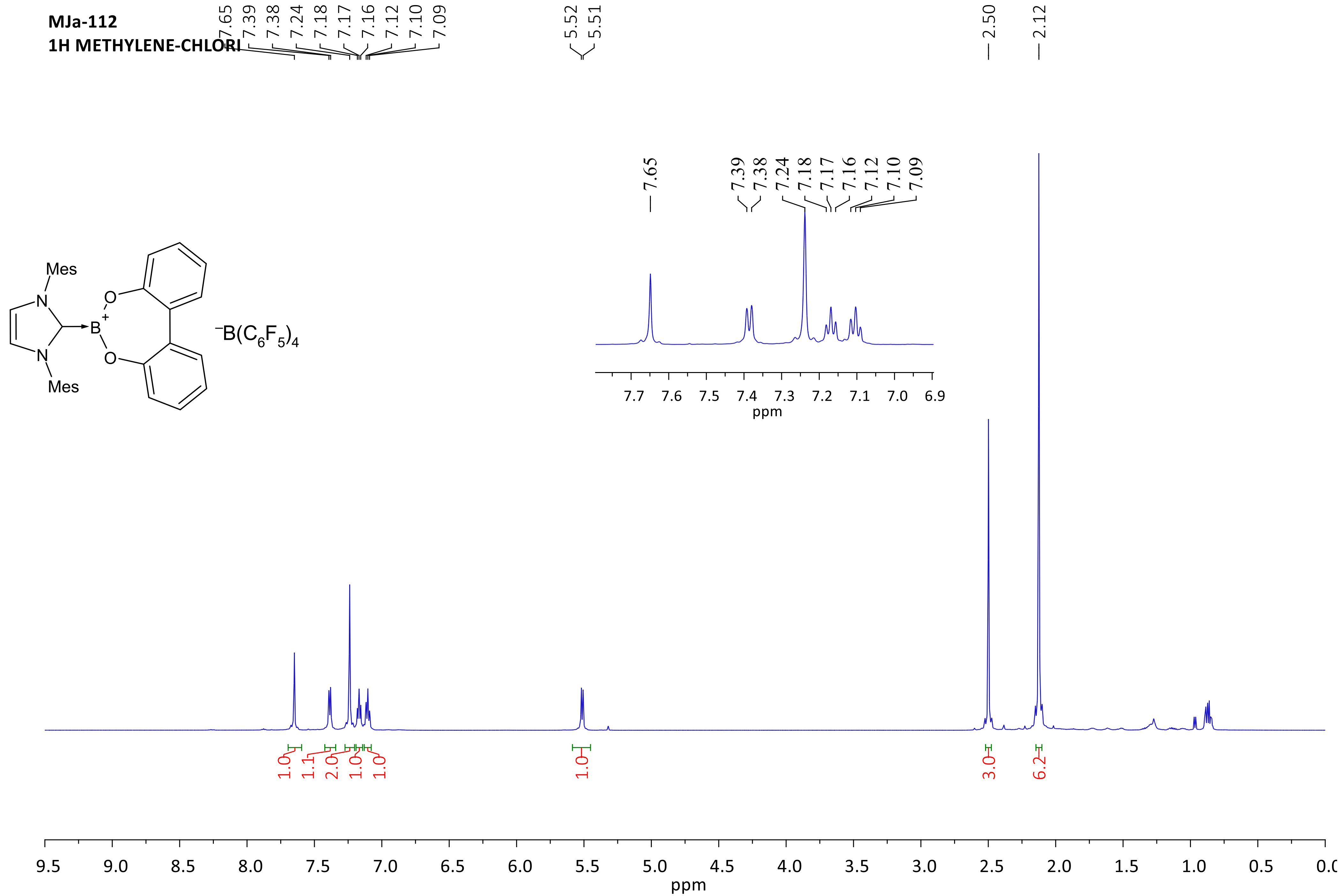

^{1}H NMR for compound ***1g'***.

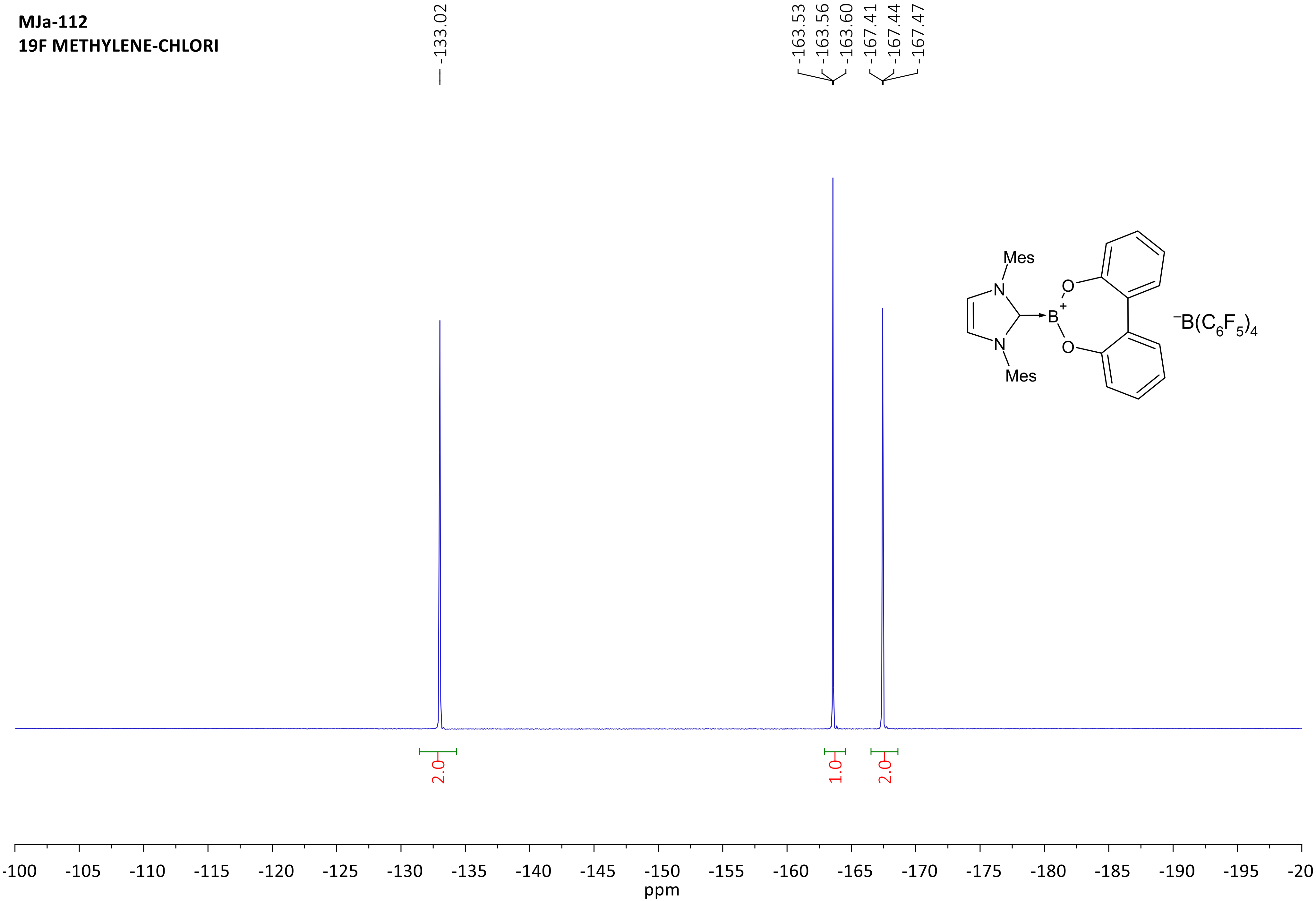

^{19}F NMR for compound ***1g'***.

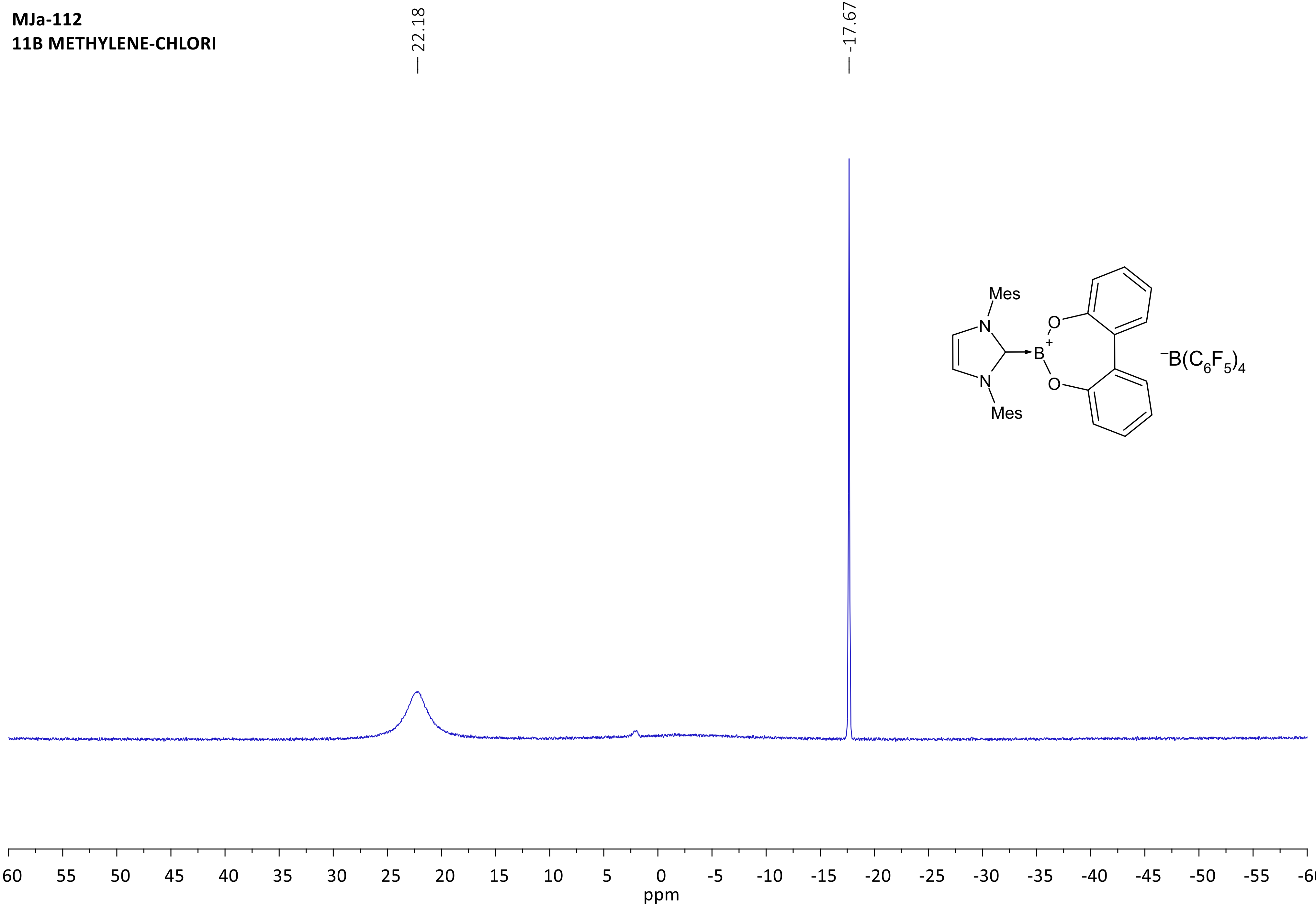

$^{11}B\{^{1}H\}$ NMR for compound ***1g'***.

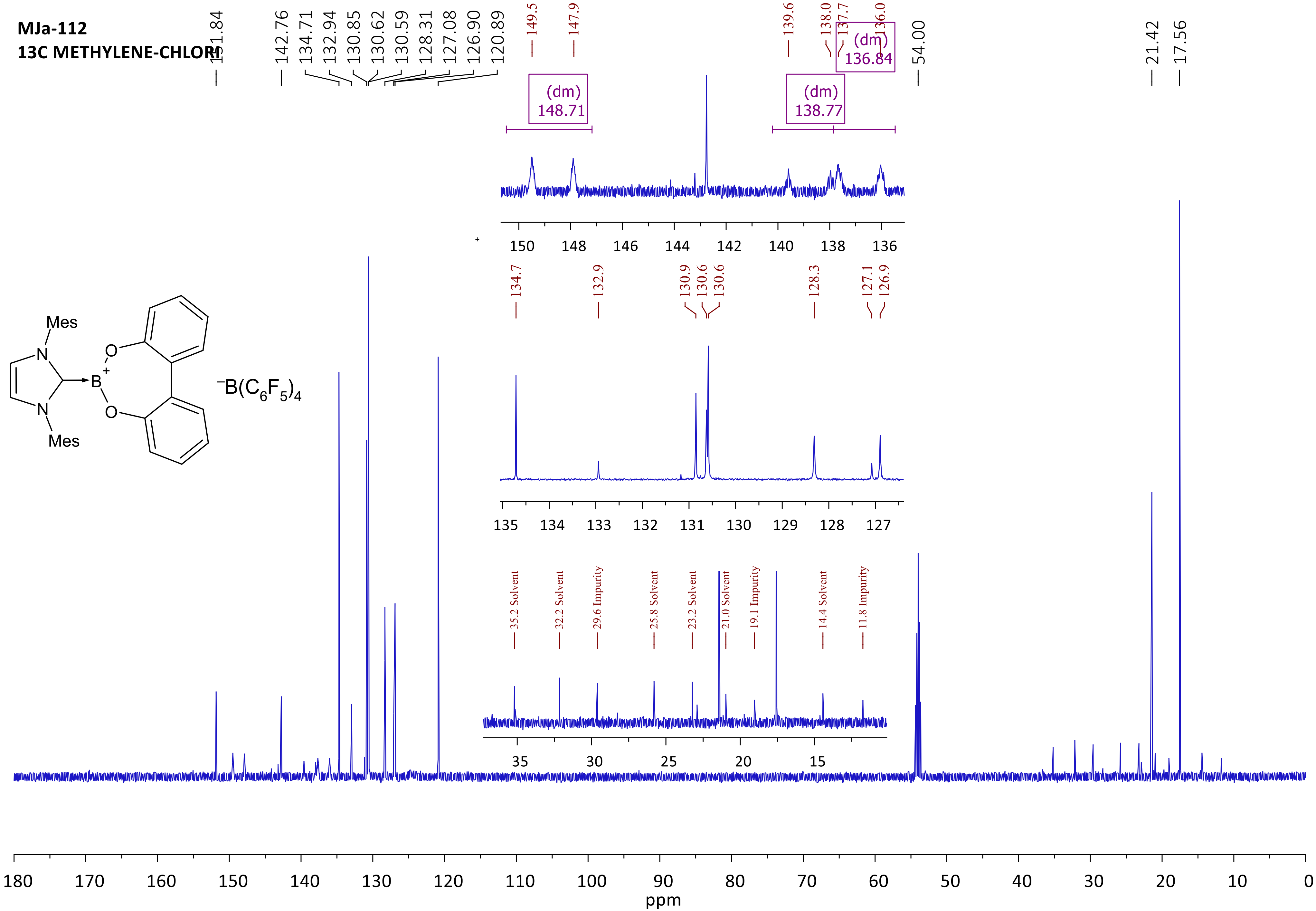

$^{13}C\{^{1}H\}$ NMR for compound ***1g'***.

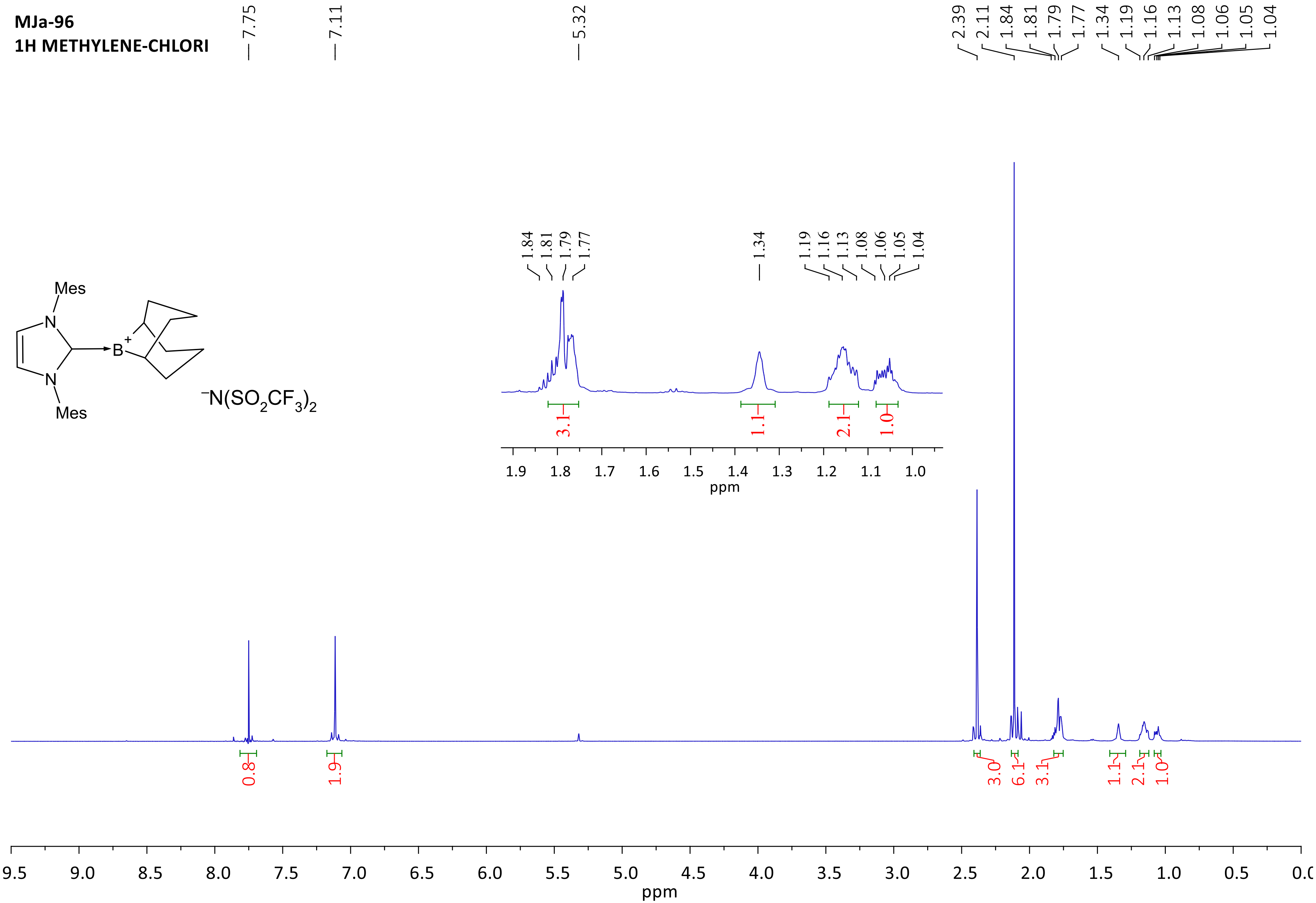

^{1}H NMR for compound ***1h***.

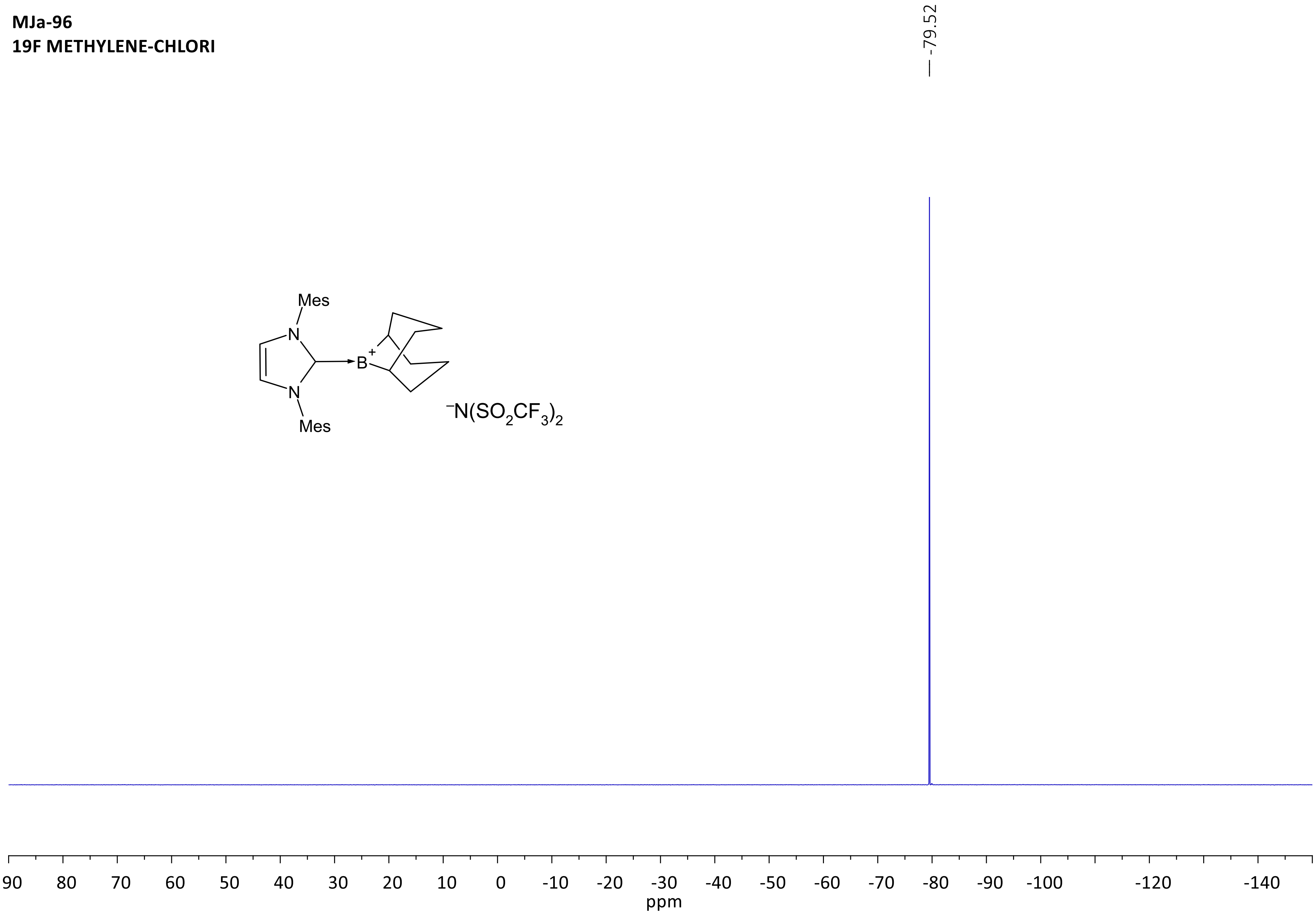

^{19}F NMR for compound ***1h***.

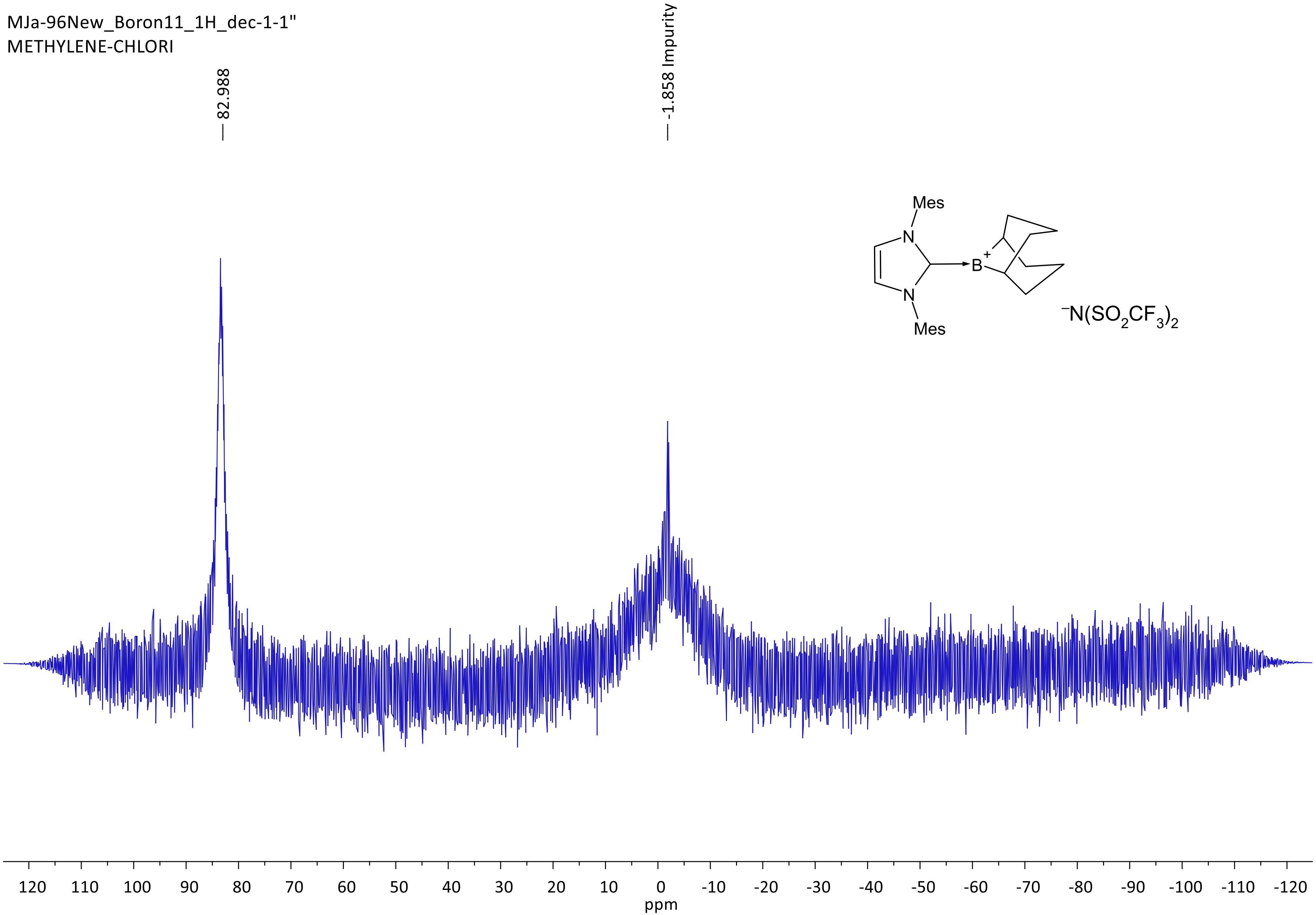

$^{11}B\{^{1}H\}$ NMR for compound ***1h***.

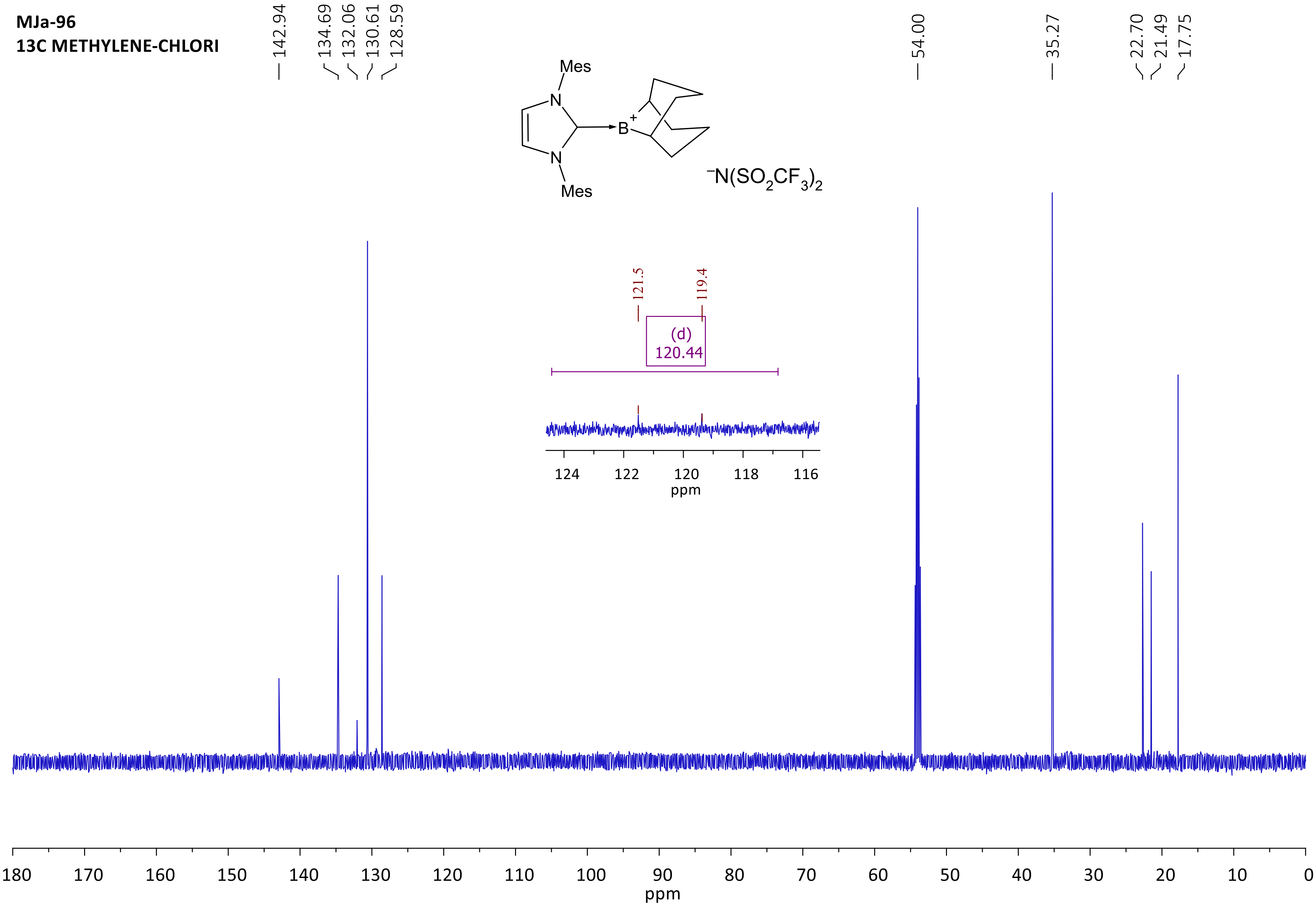

$^{13}C\{^{1}H\}$ NMR for compound ***1h***.

MJa-220-S1
1H METHYLENE-CHLORI

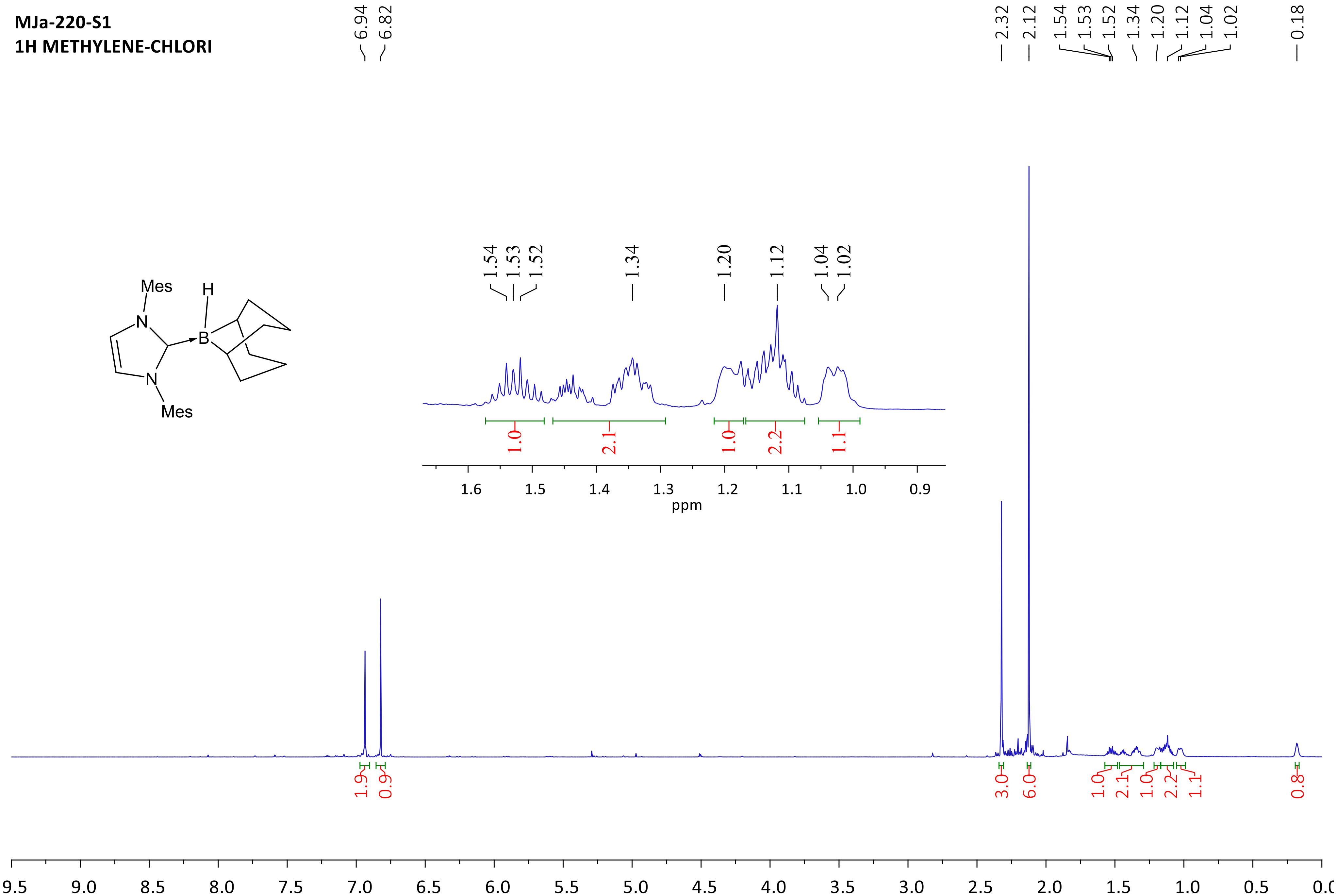

^{1}H NMR for compound ***1h*H**.

MJa-220-S1
11B METHYLENE-CHLORI

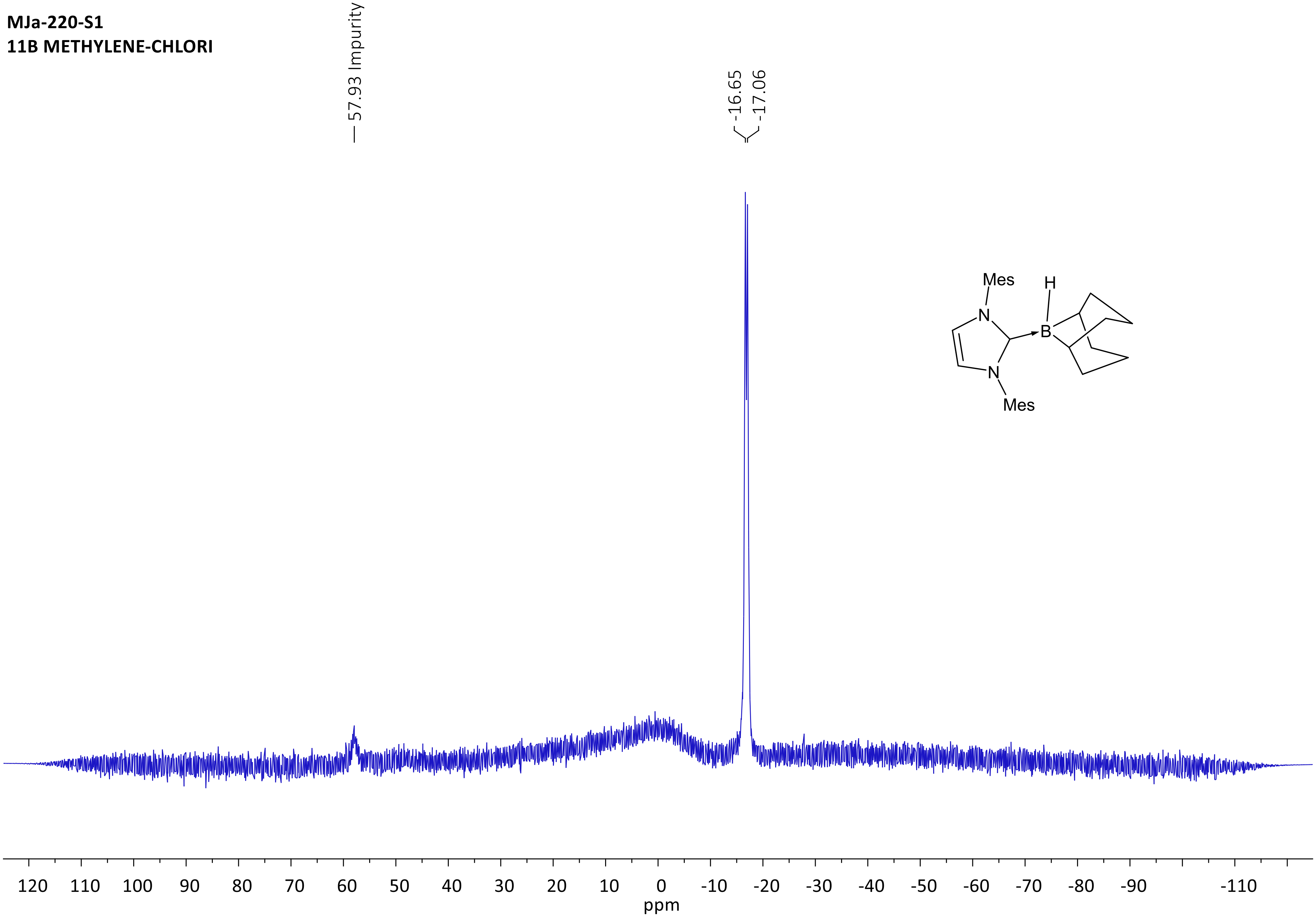

^{11}B NMR for compound ***1h***.

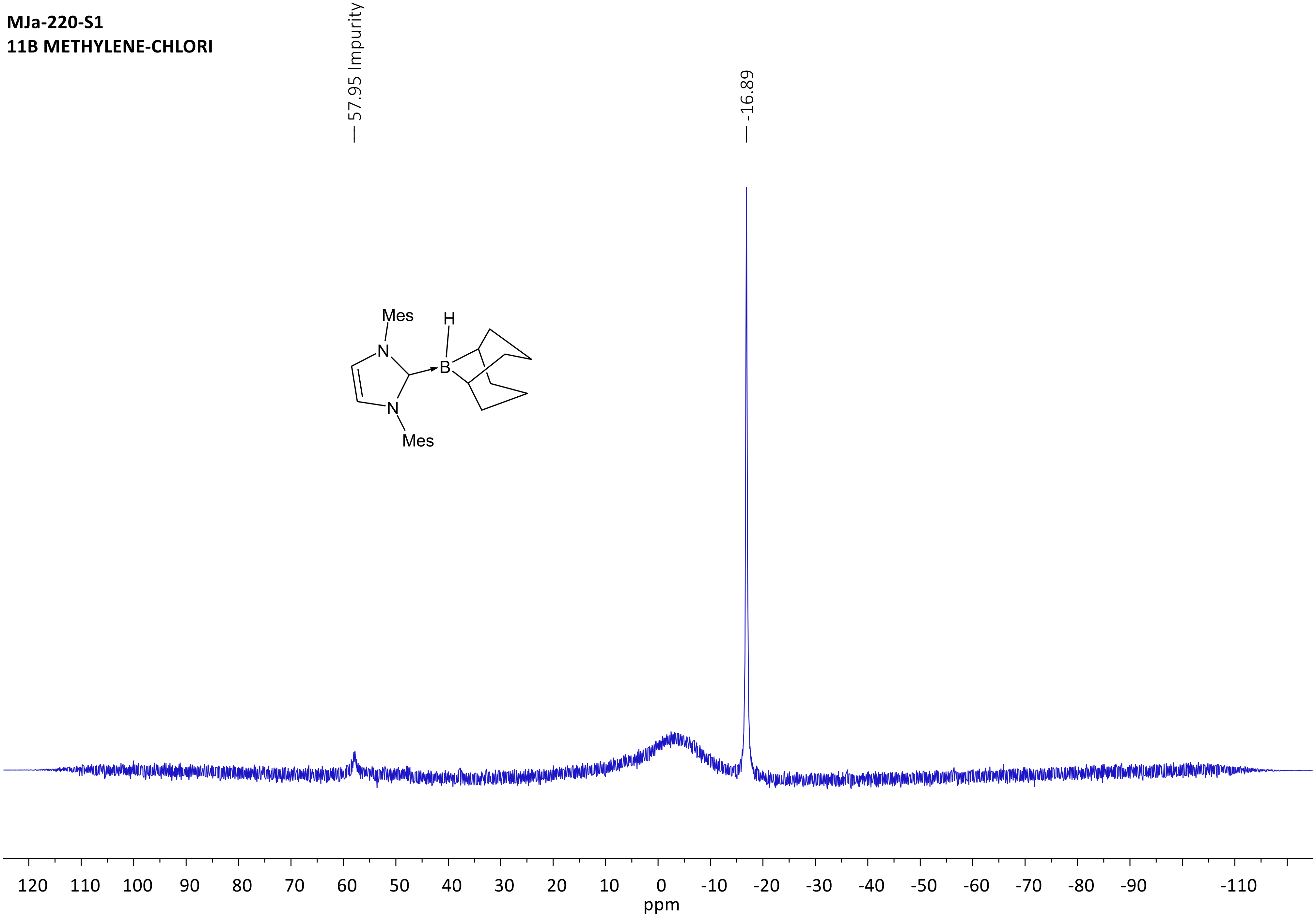

$^{11}B\{^{1}H\}$ NMR for compound ***1h***.

MJa-220-13C
13C METHYLENE-CHLORI

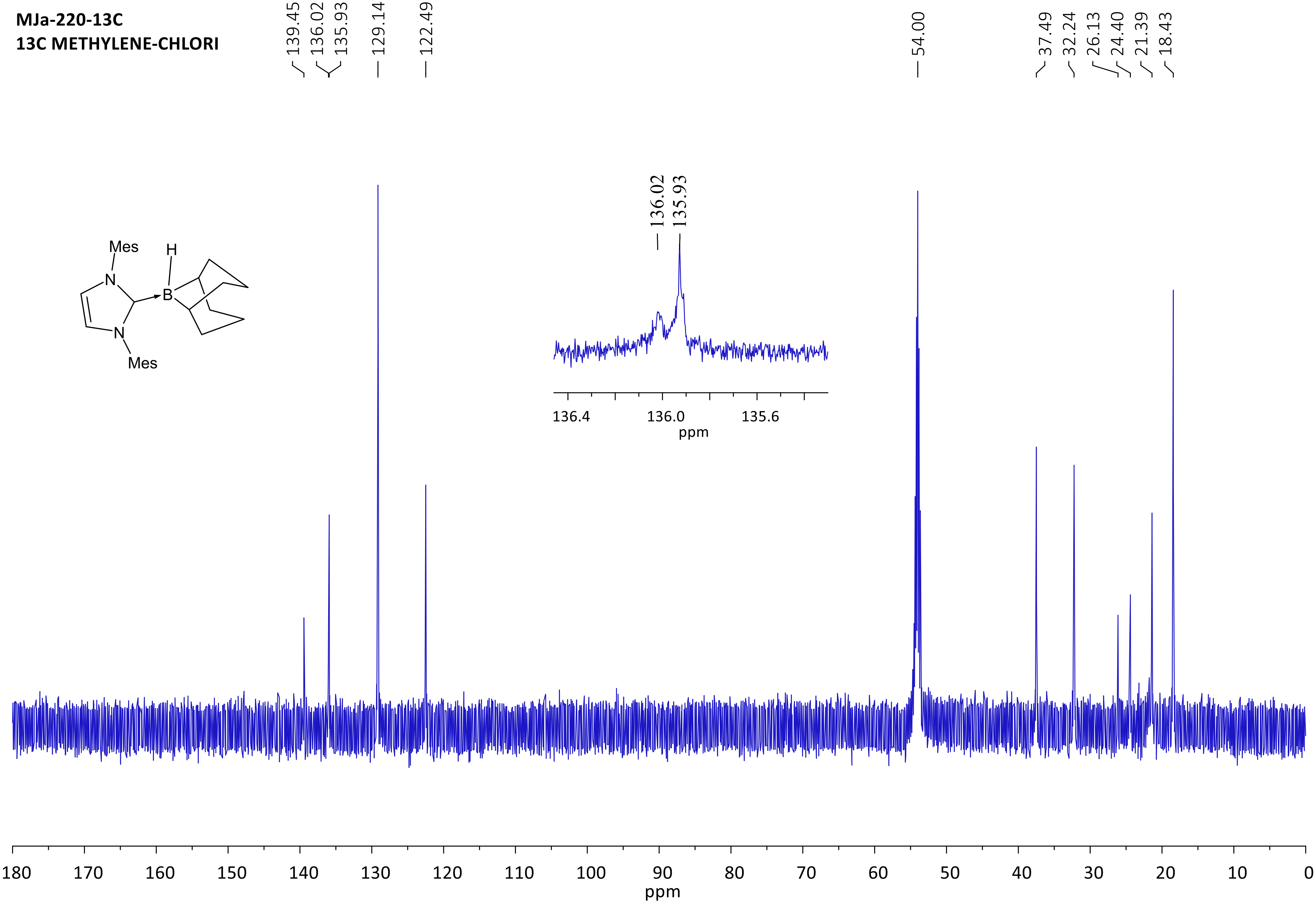

$^{13}C\{^{1}H\}$ NMR for compound ***1h***.

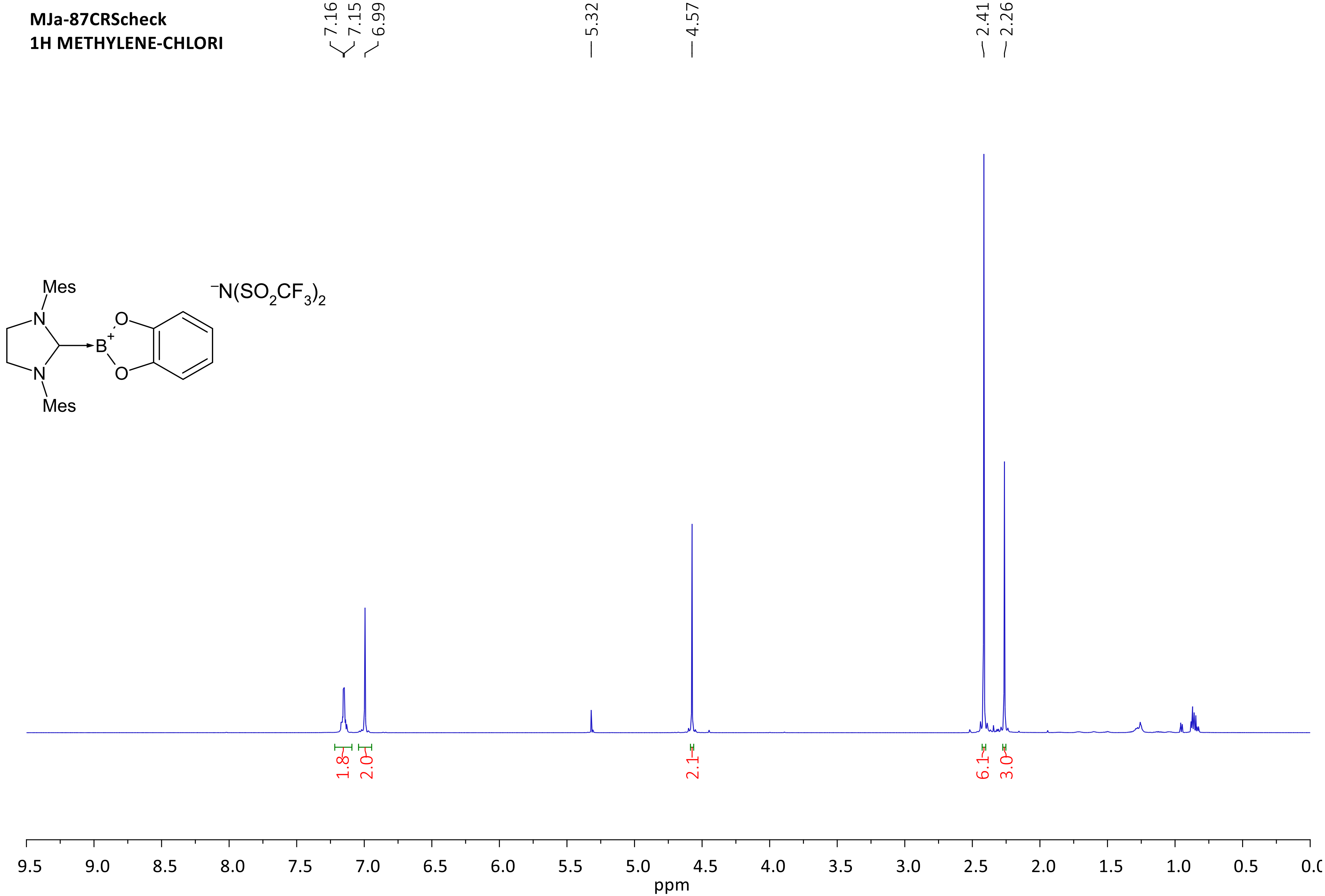

^{1}H NMR for compound ***2b***.

MJa-87CRScheck
19F METHYLENE-CHLORI

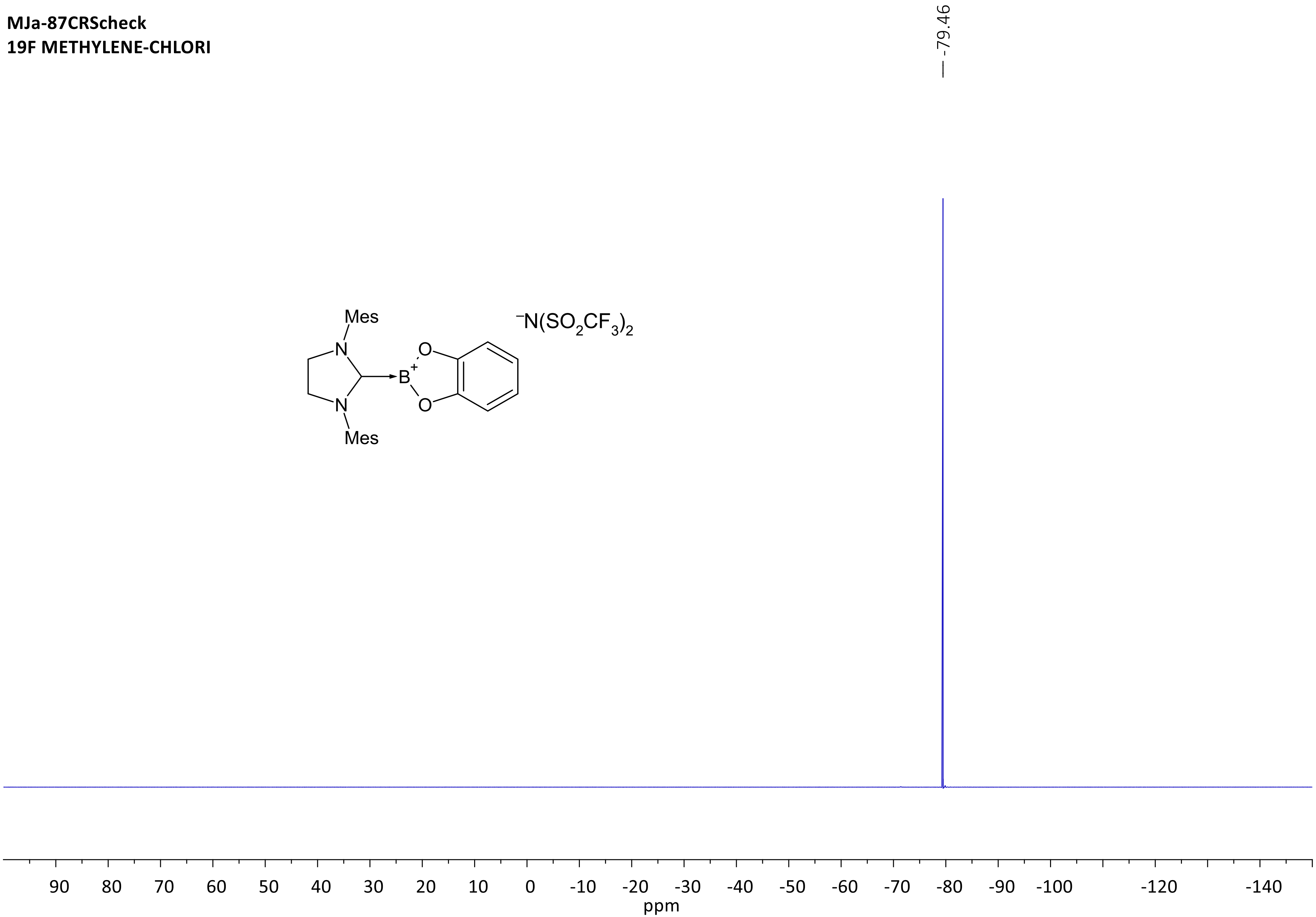

^{19}F NMR for compound ***2b***.

MJa-87CRScheck
11B METHYLENE-CHLORI

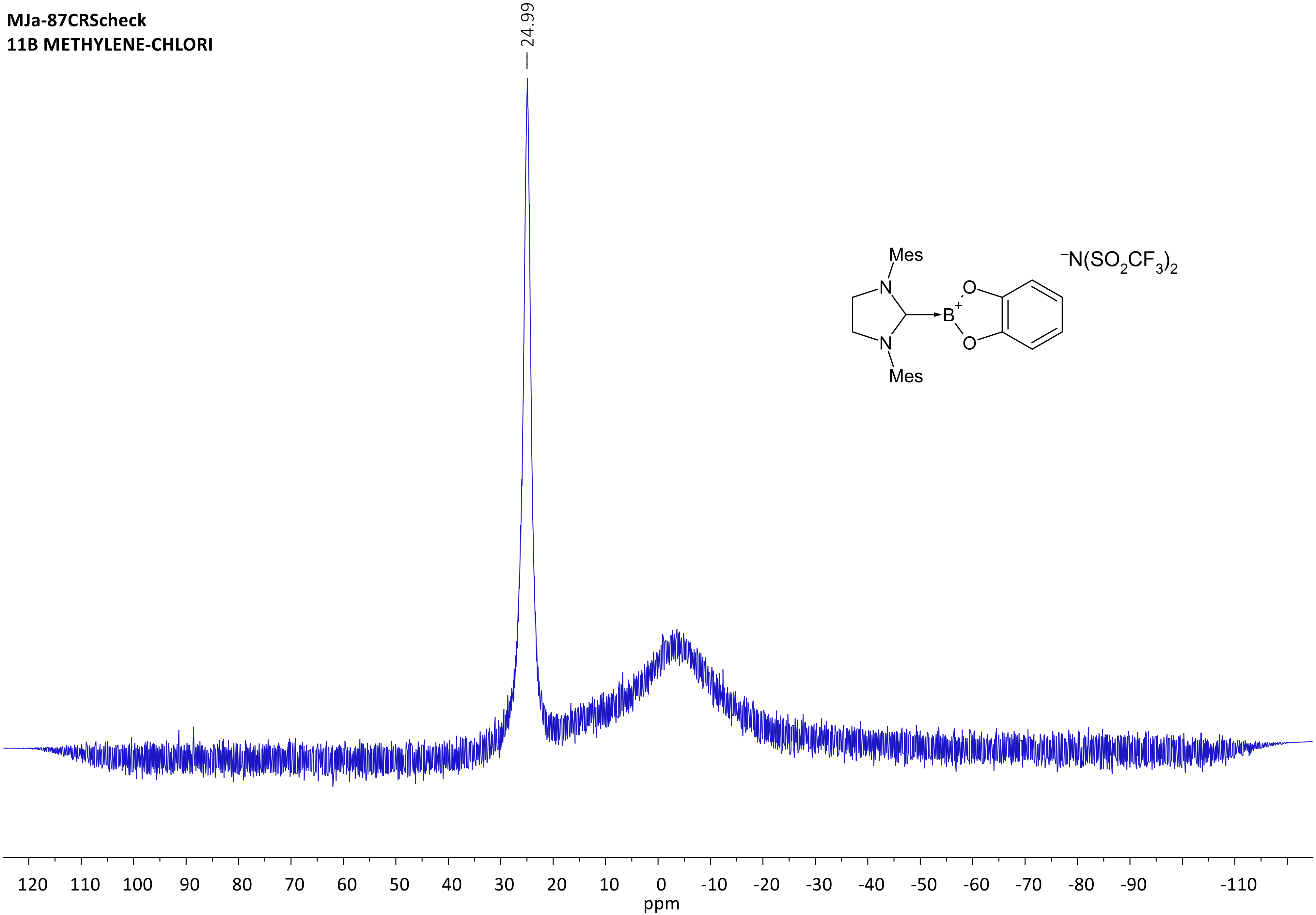

$^{11}B\{^{1}H\}$ NMR for compound ***2b***.

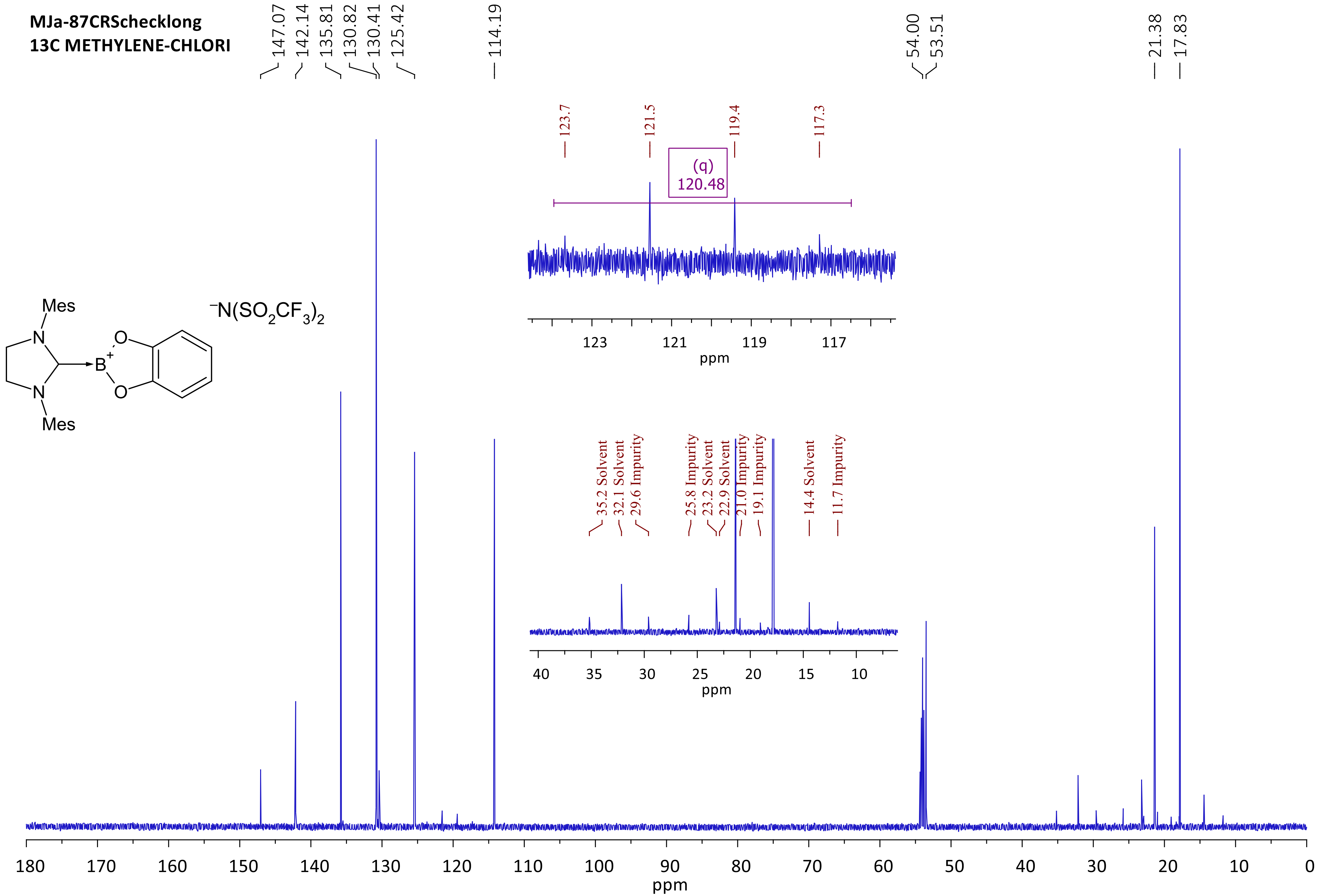

$^{13}C\{^{1}H\}$ NMR for compound ***2b***.

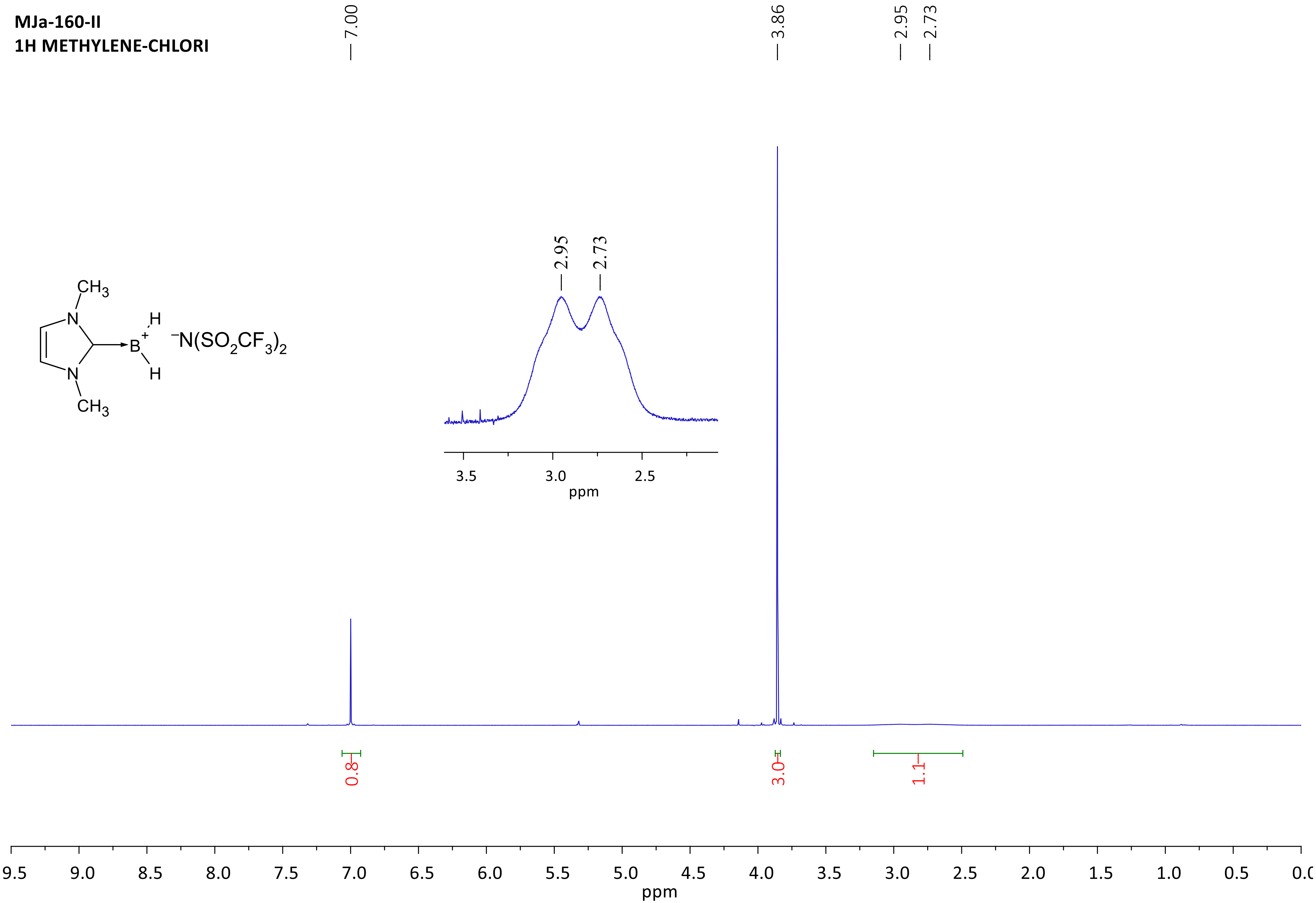

^{1}H NMR for compound ***3a***.

MJa-160-II
19F METHYLENE-CHLORI

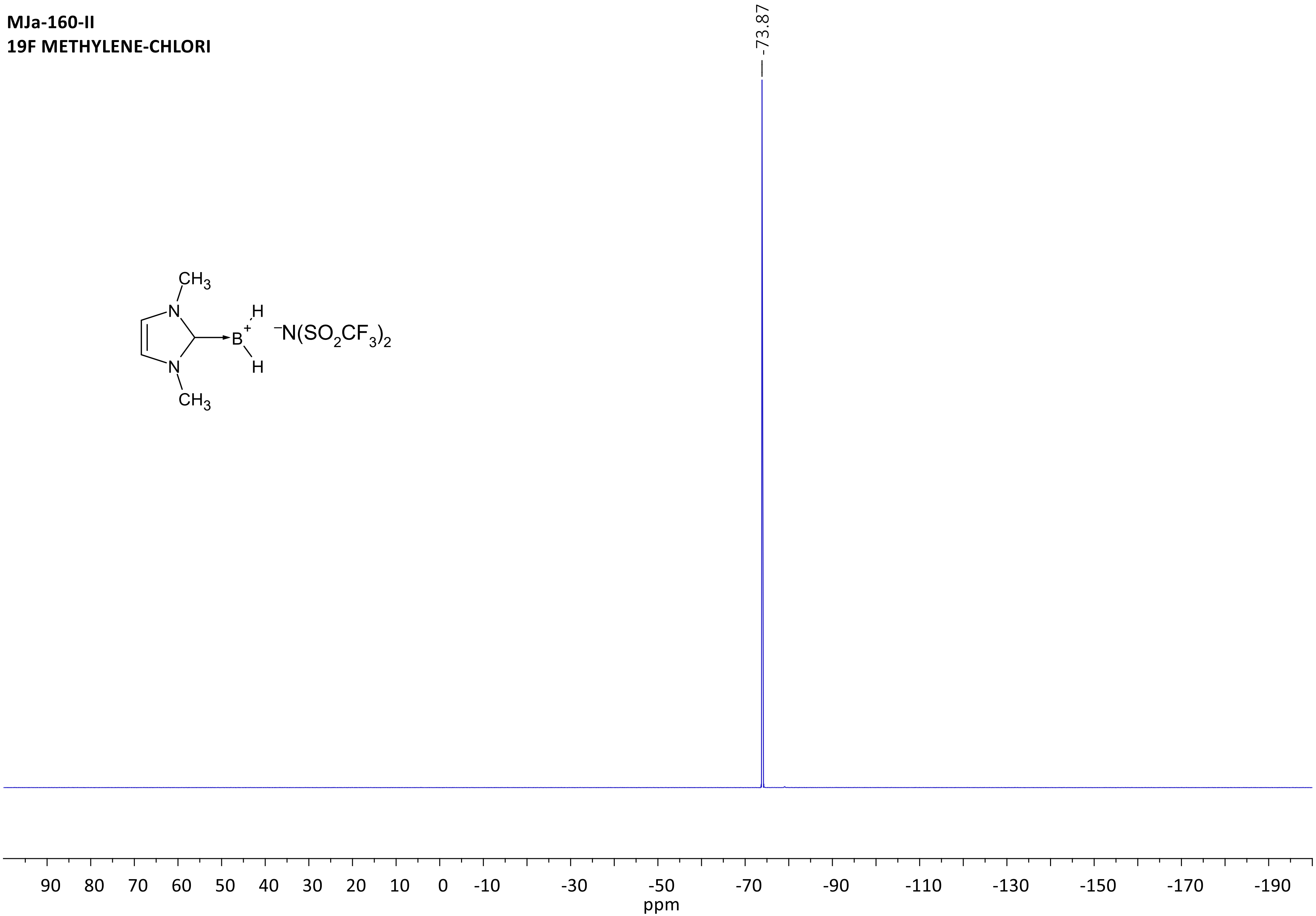

^{19}F NMR for compound ***3a***.

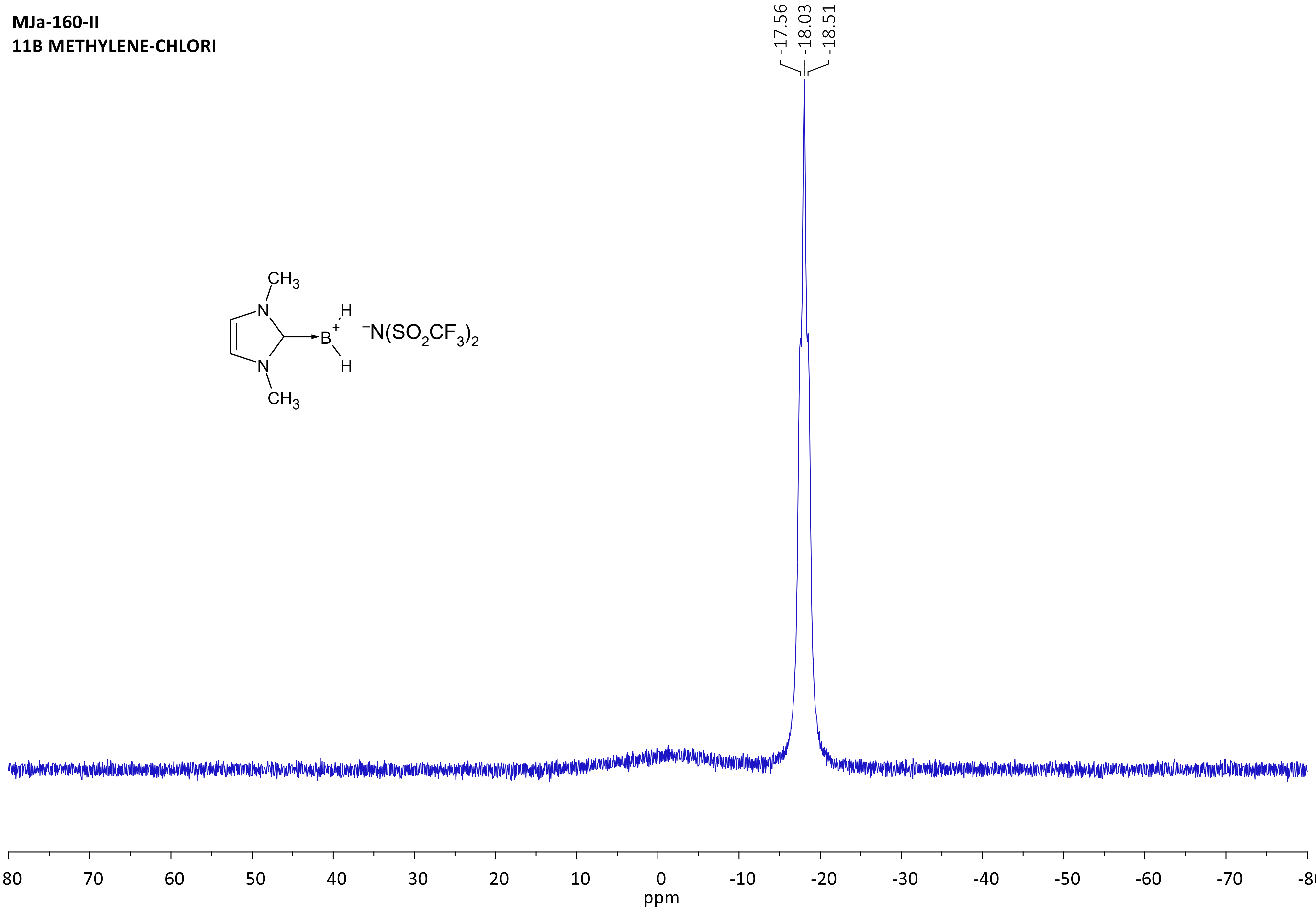

^{11}B NMR for compound ***3a***.

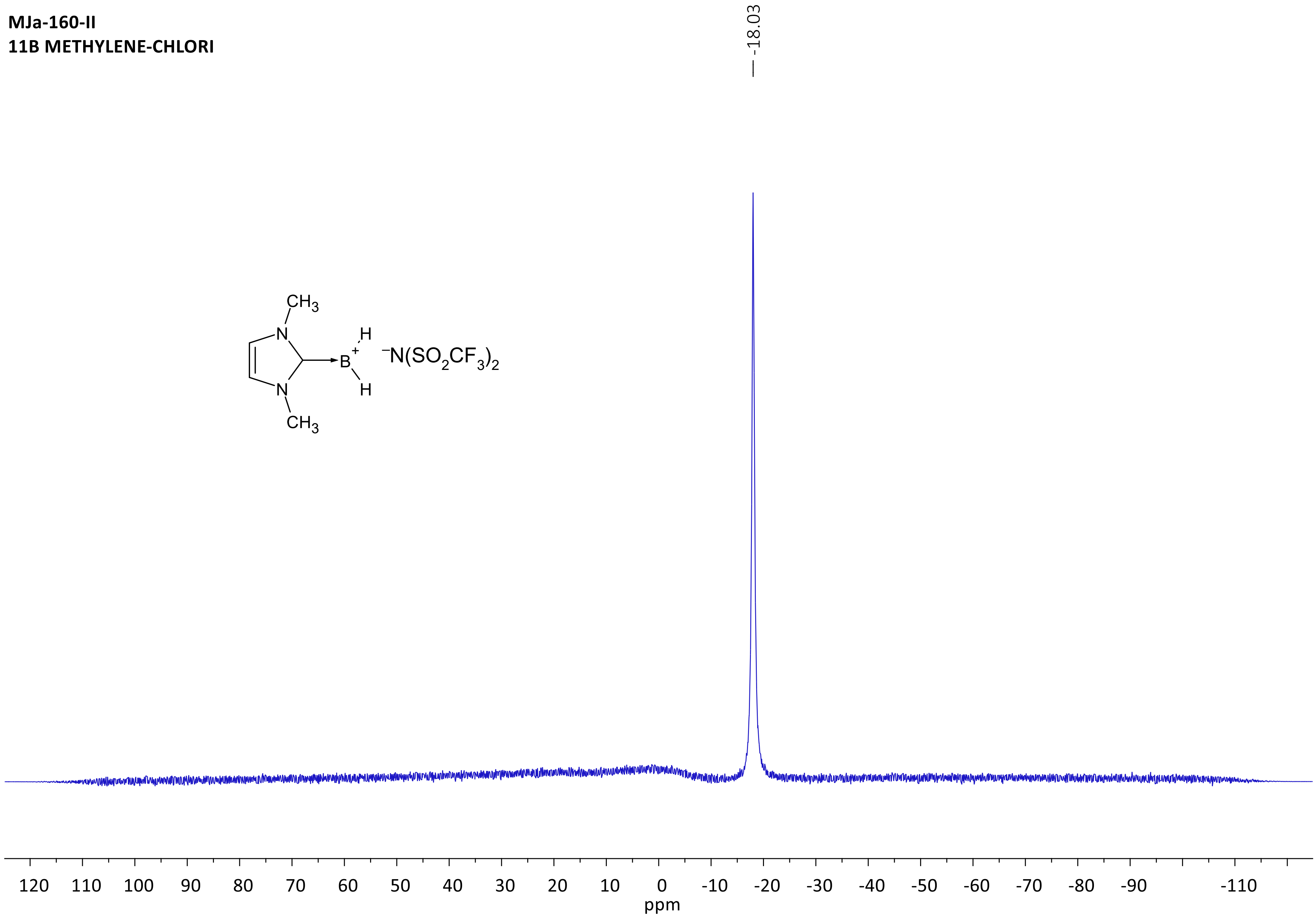

$^{11}B\{^{1}H\}$ NMR for compound ***3a***.

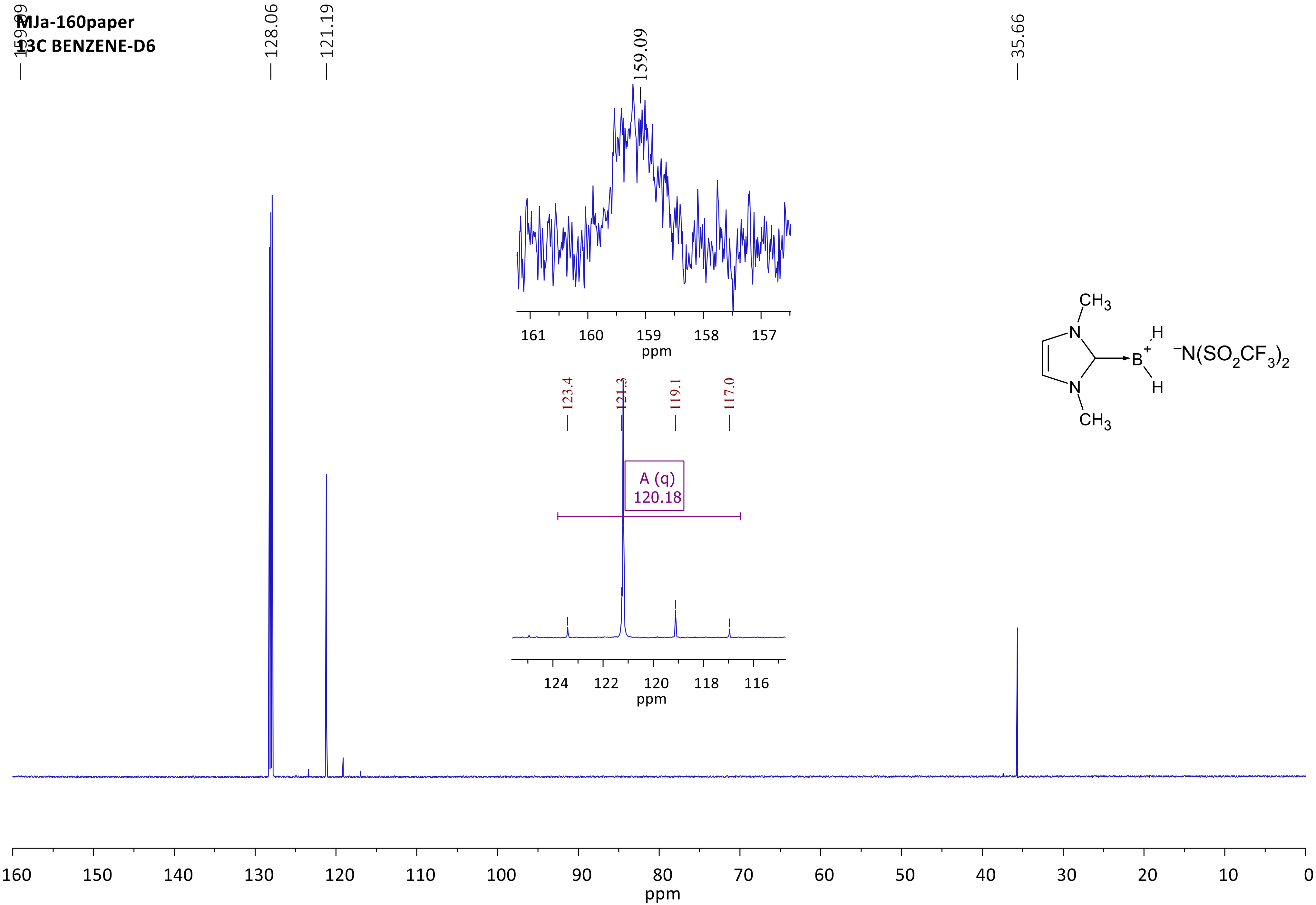

$^{13}C\{^1H\}$ NMR for compound ***3a***.

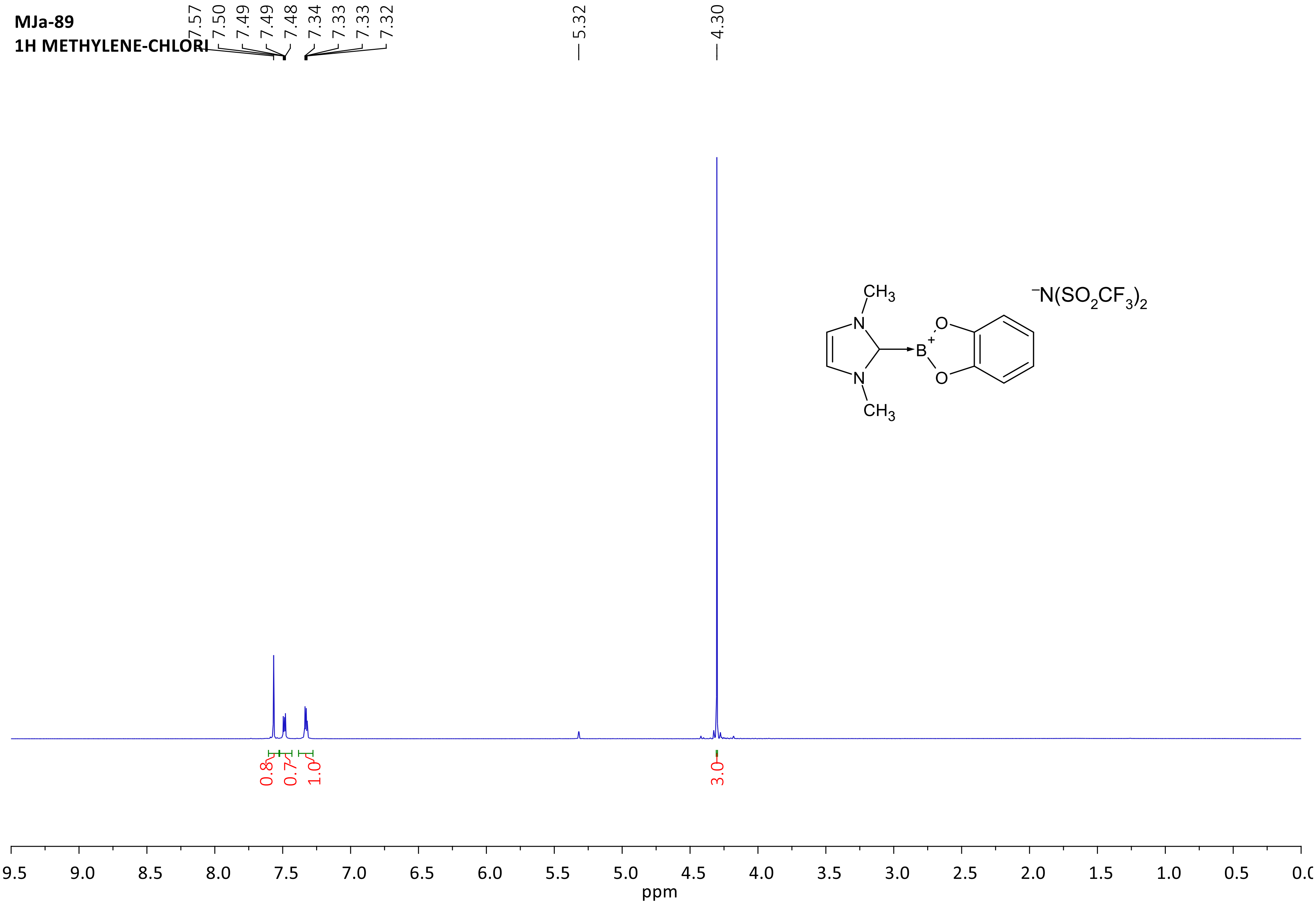

^{1}H NMR for compound ***3b***.

MJa-89
19F METHYLENE-CHLORI

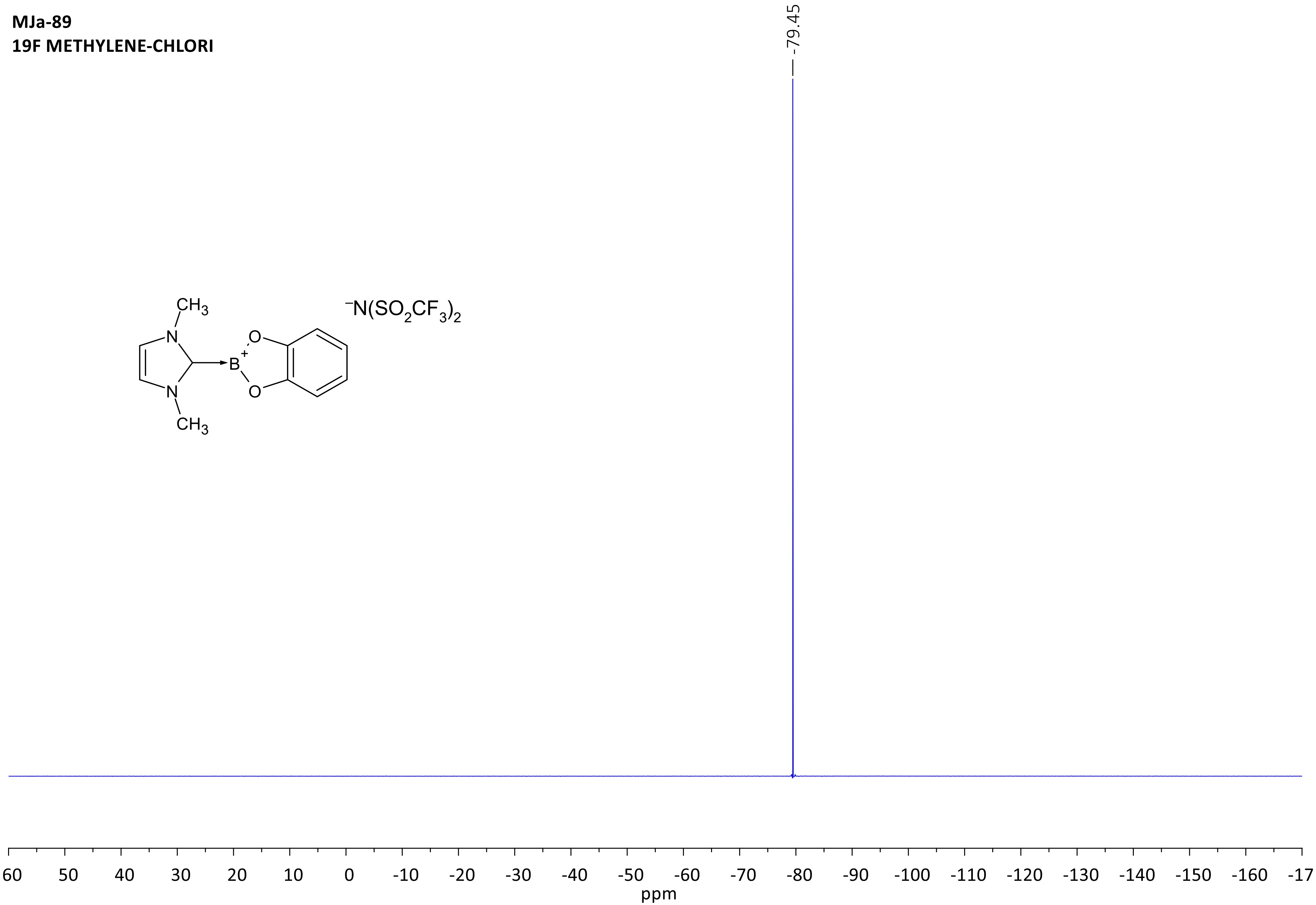

^{19}F NMR for compound ***3b***.

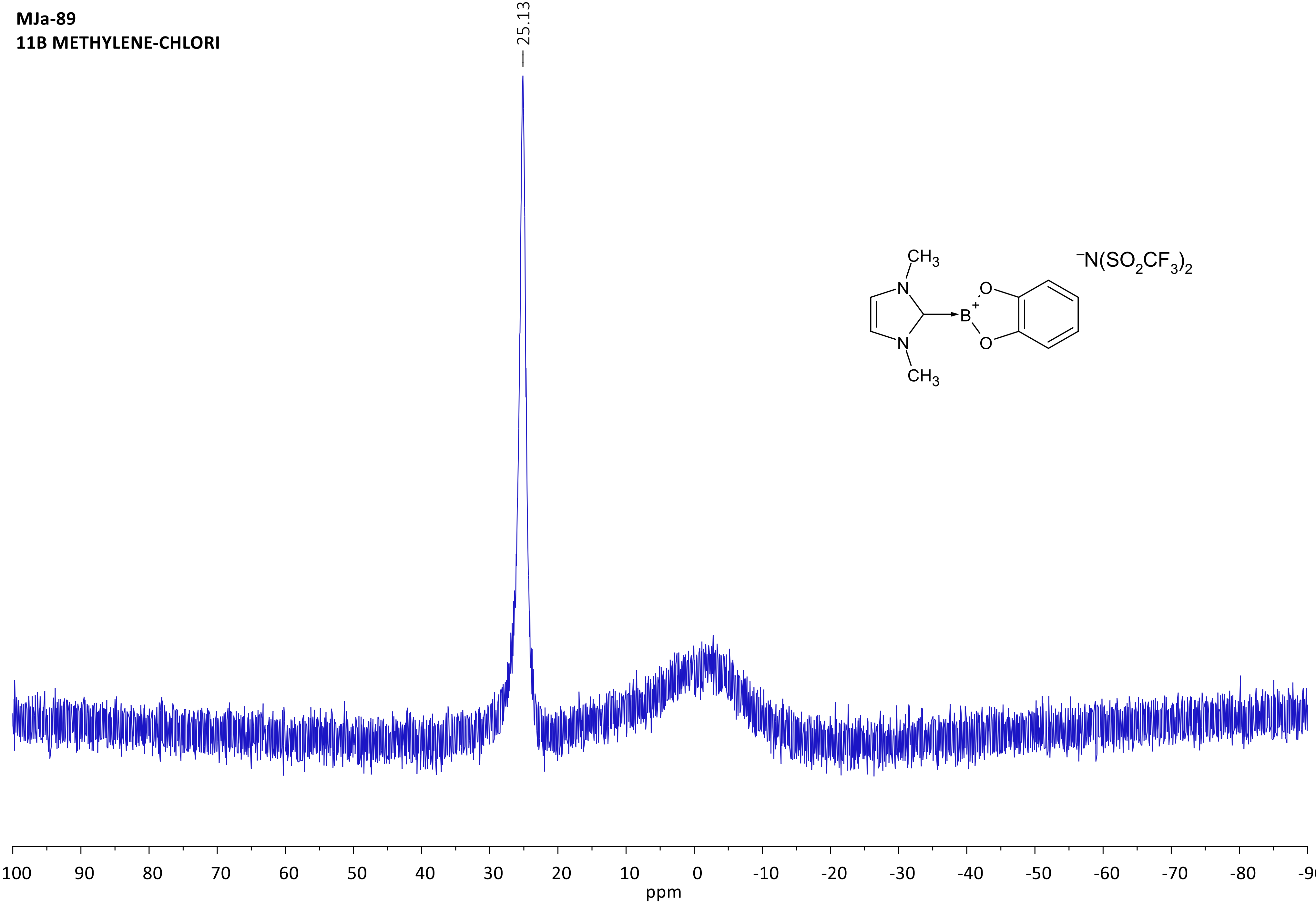

$^{11}B\{^1H\}$ NMR for compound ***3b***.

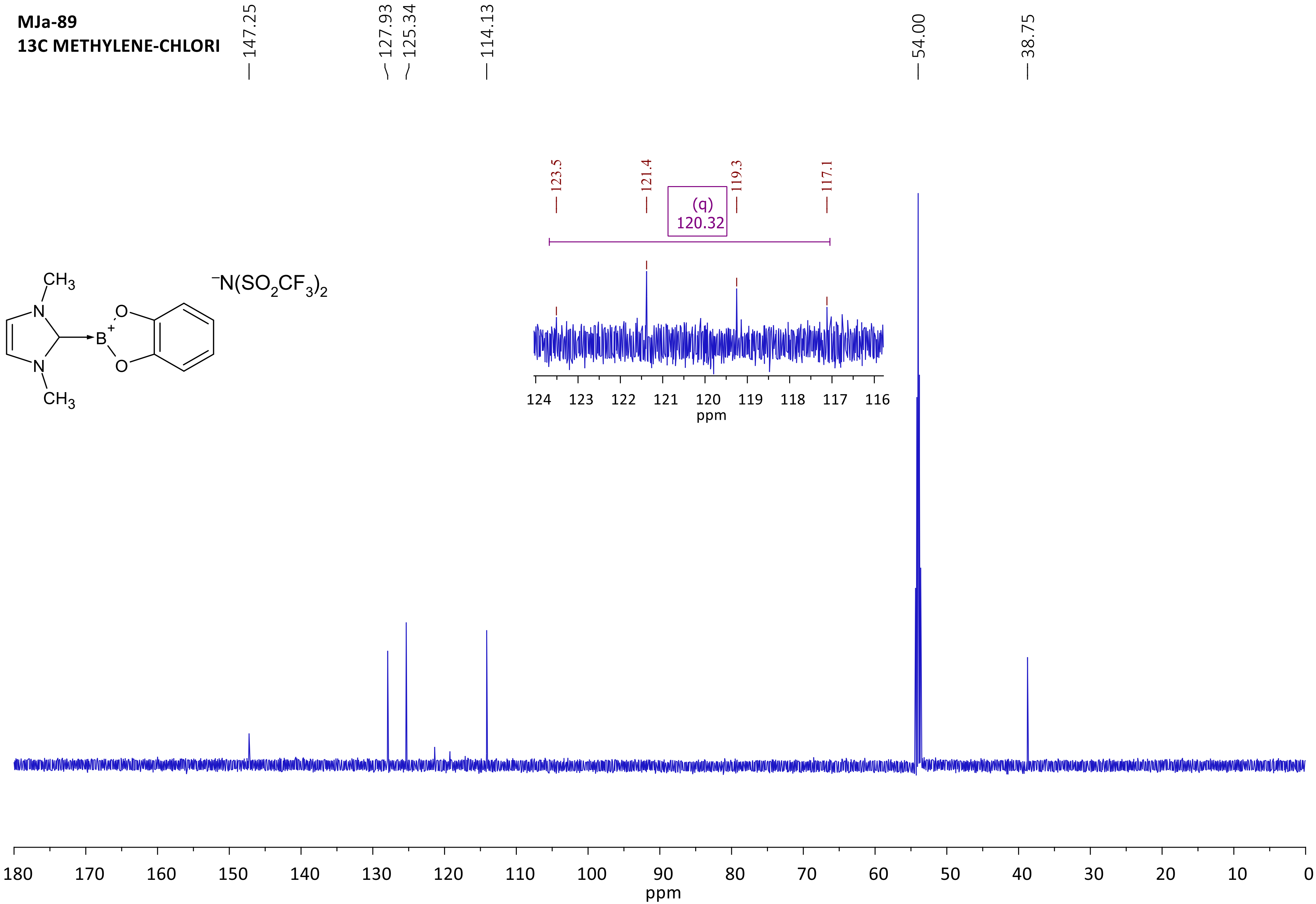

$^{13}C\{^1H\}$ NMR for compound ***3b***.

MJa-158-II-CDCl3
1H CHLOROFORM-D

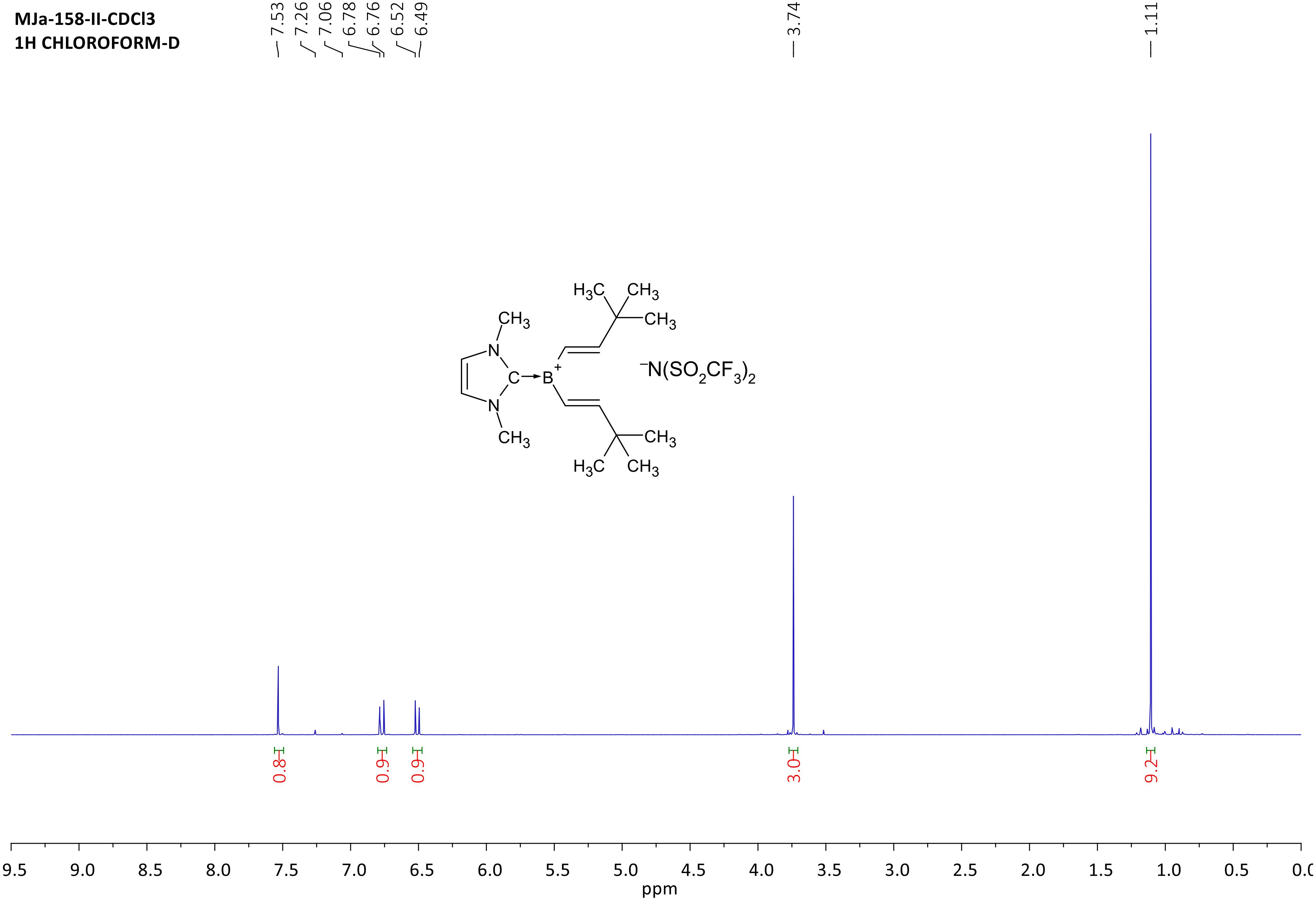

^{1}H NMR for compound ***3c***.

MJa-158-I-CDCl3
19F CHLOROFORM-D

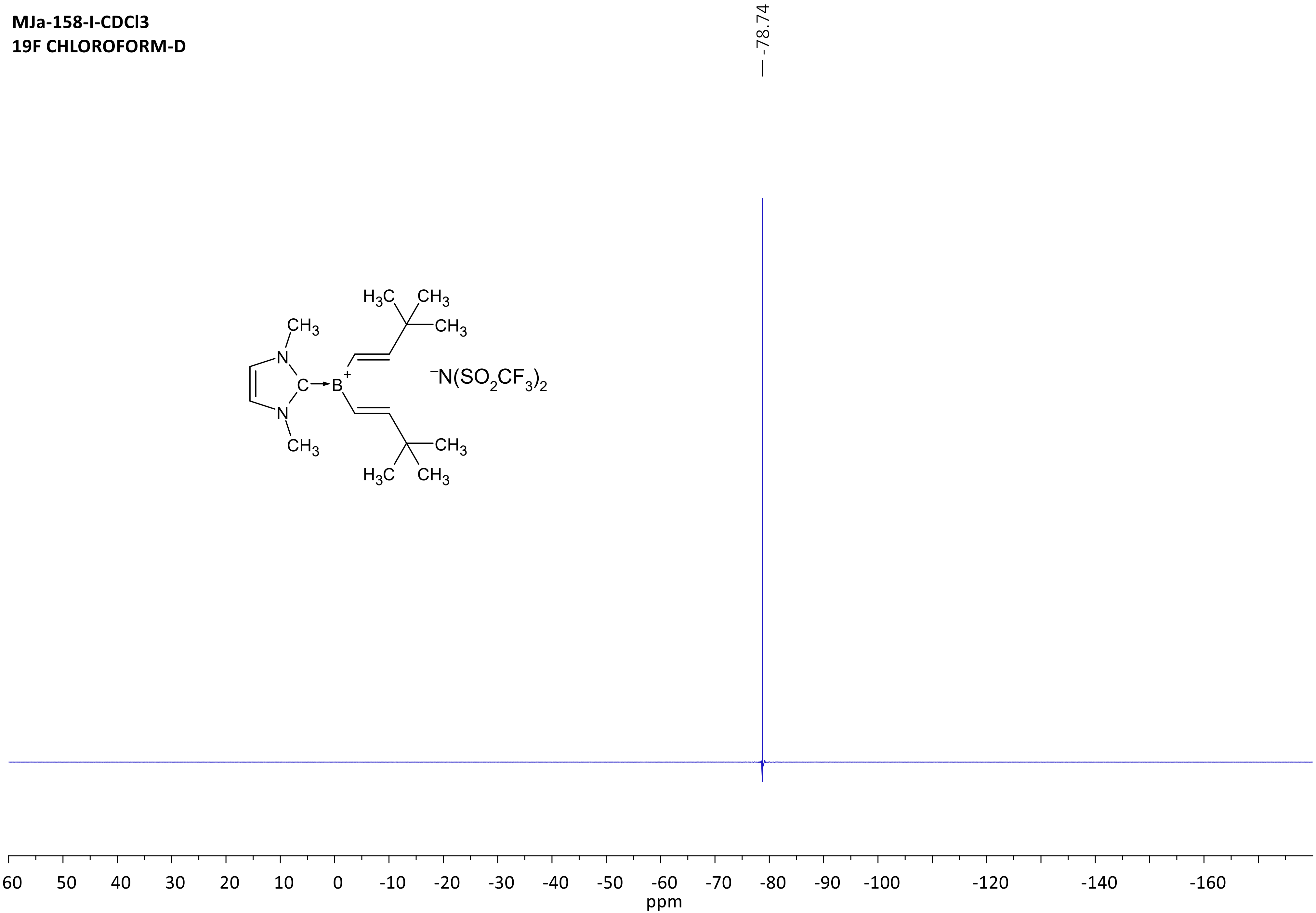

^{19}F NMR for compound ***3c***.

MJa-52
11B METHYLENE-CHLORI

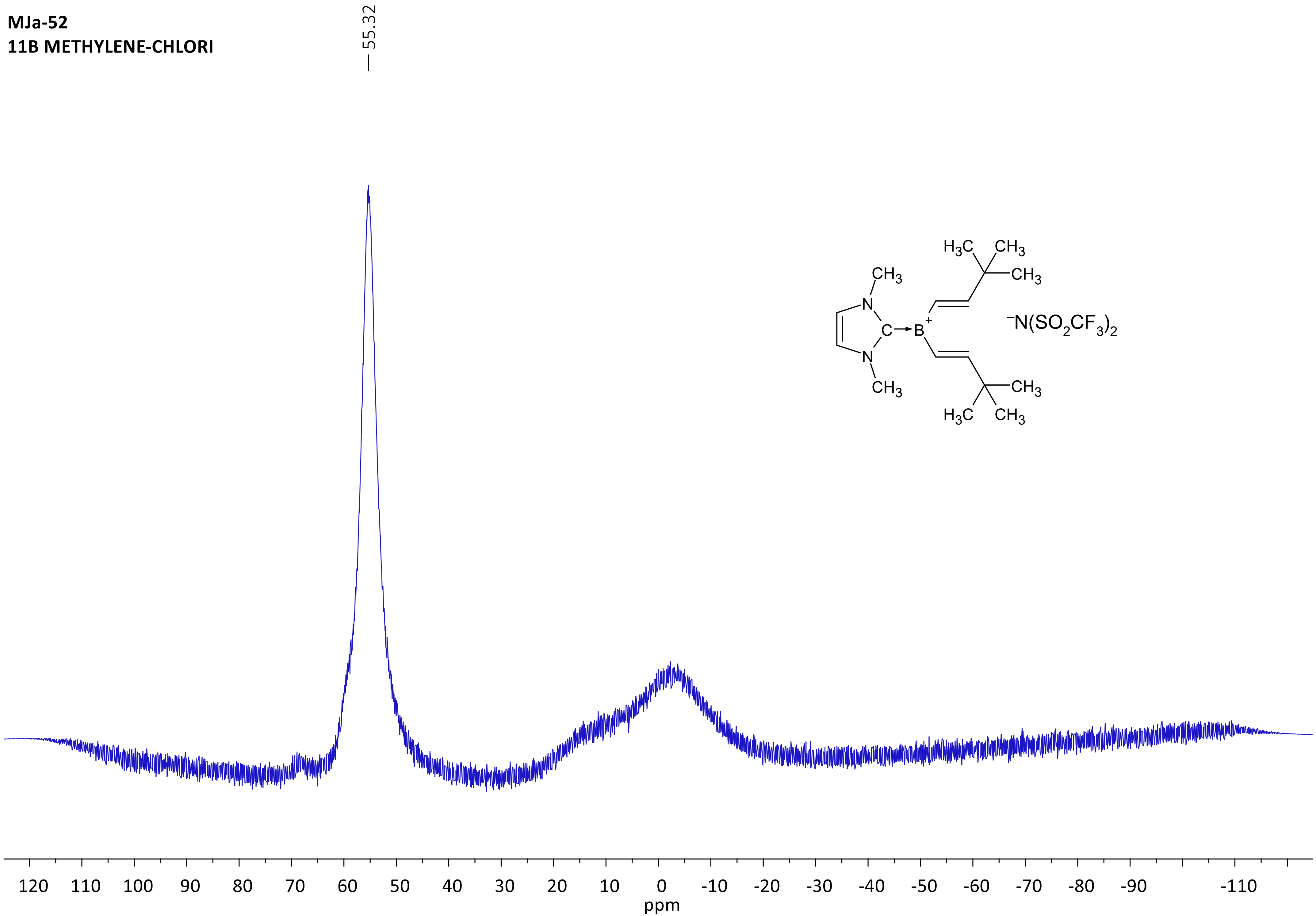

$^{11}B\{^{1}H\}$ NMR for compound ***3c***.

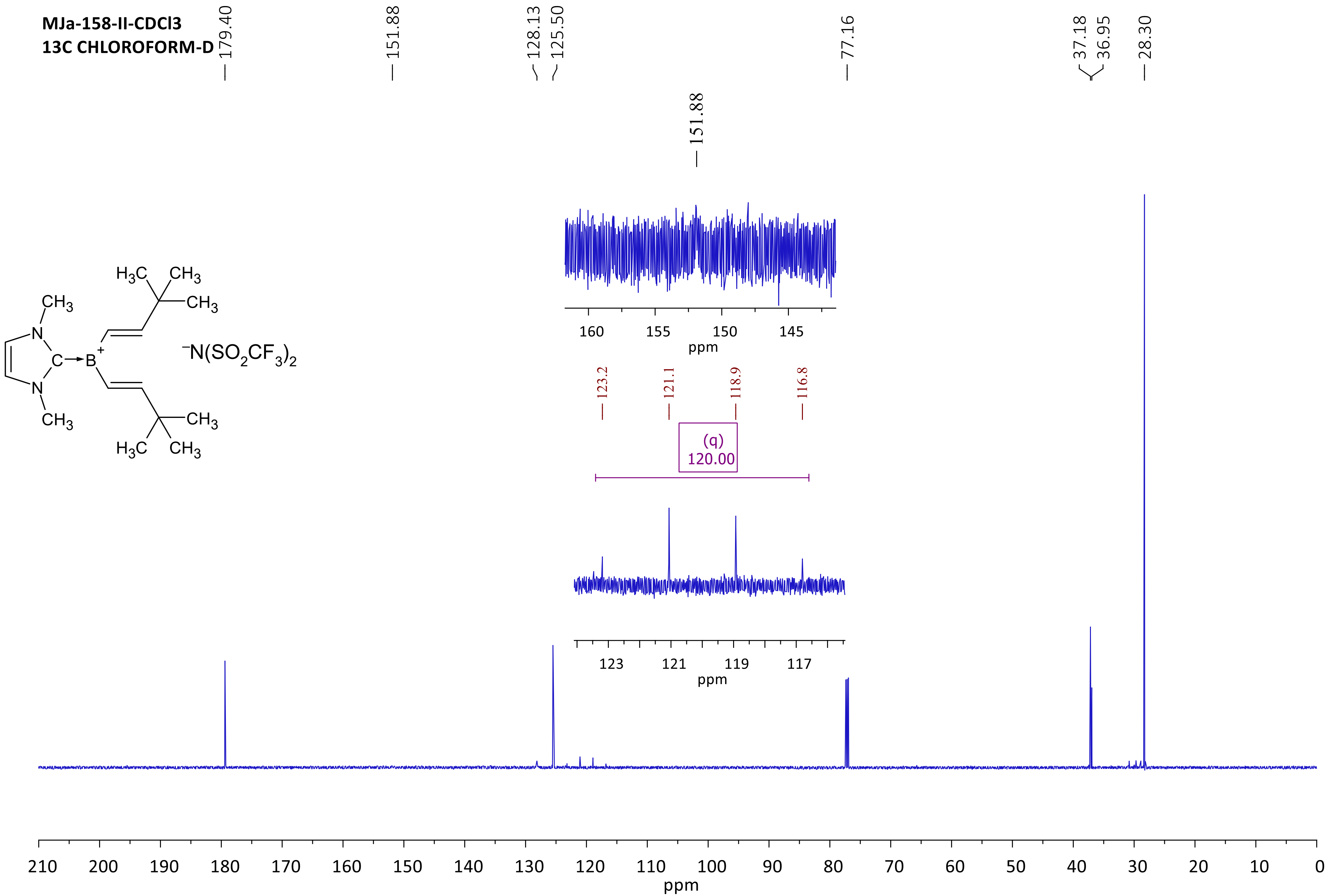

$^{13}C\{^1H\}$ NMR for compound ***3c***.

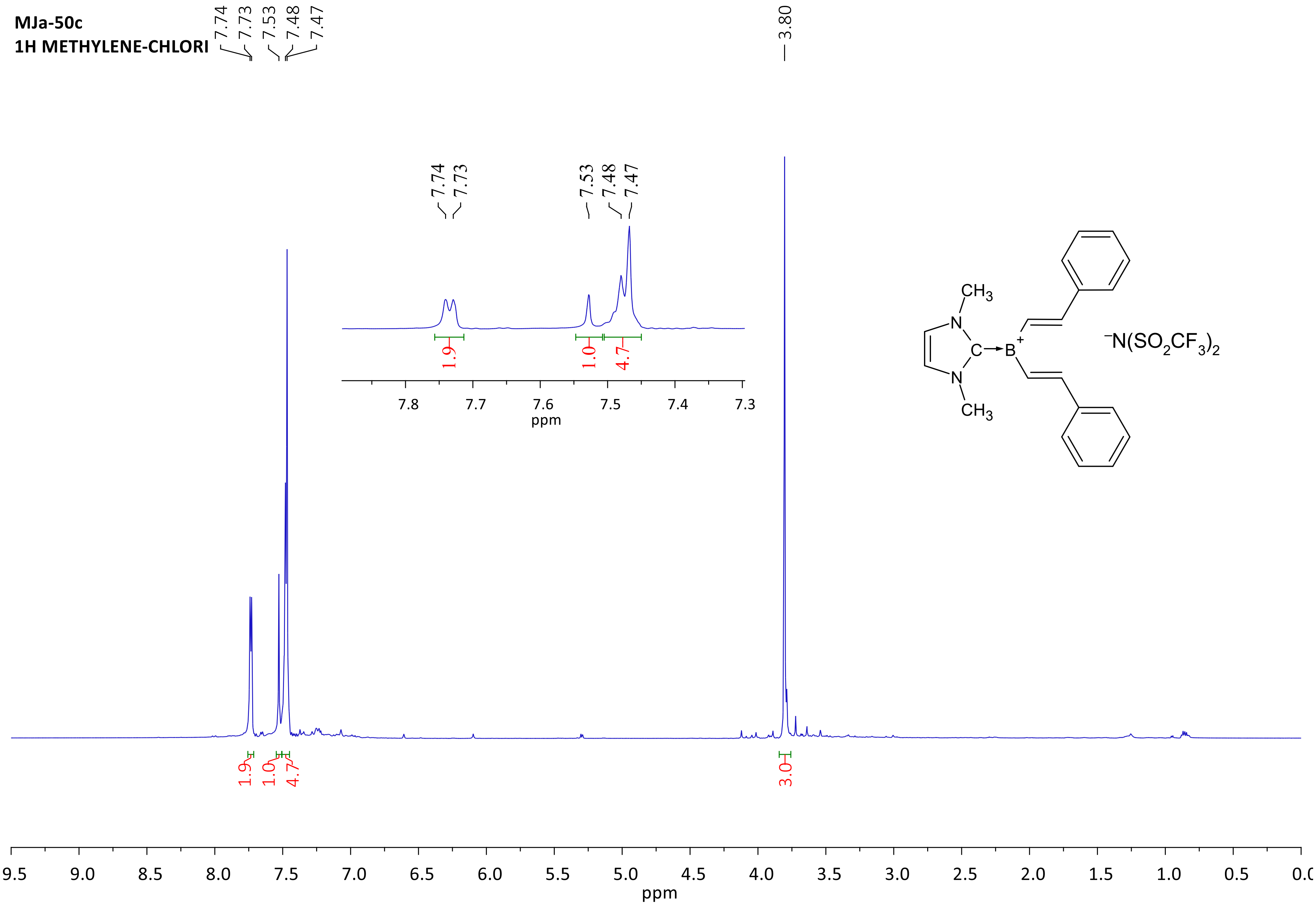

^1H NMR for compound ***3d***.

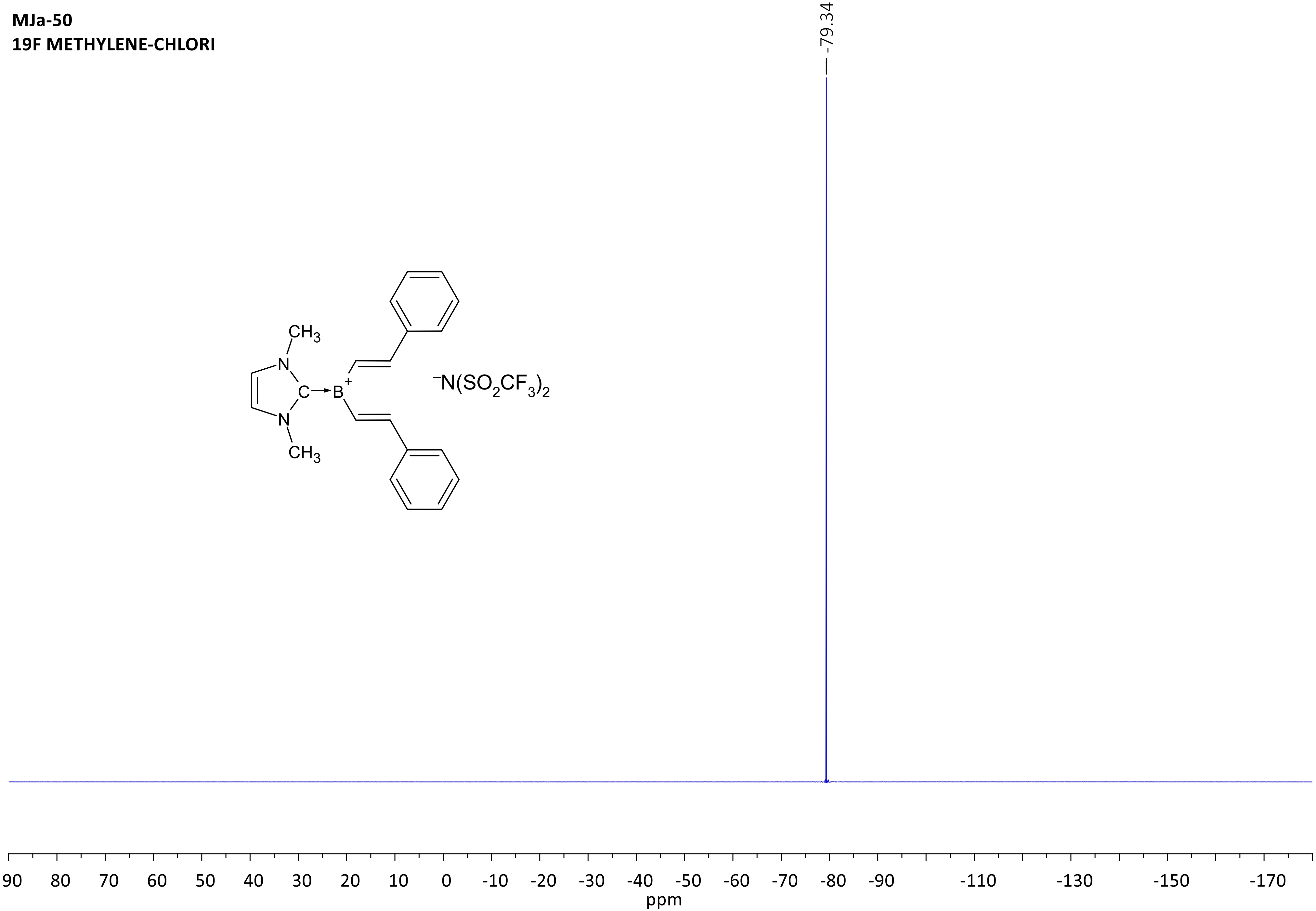

^{19}F NMR for compound ***3d***.

MJa-50
11B METHYLENE-CHLORI

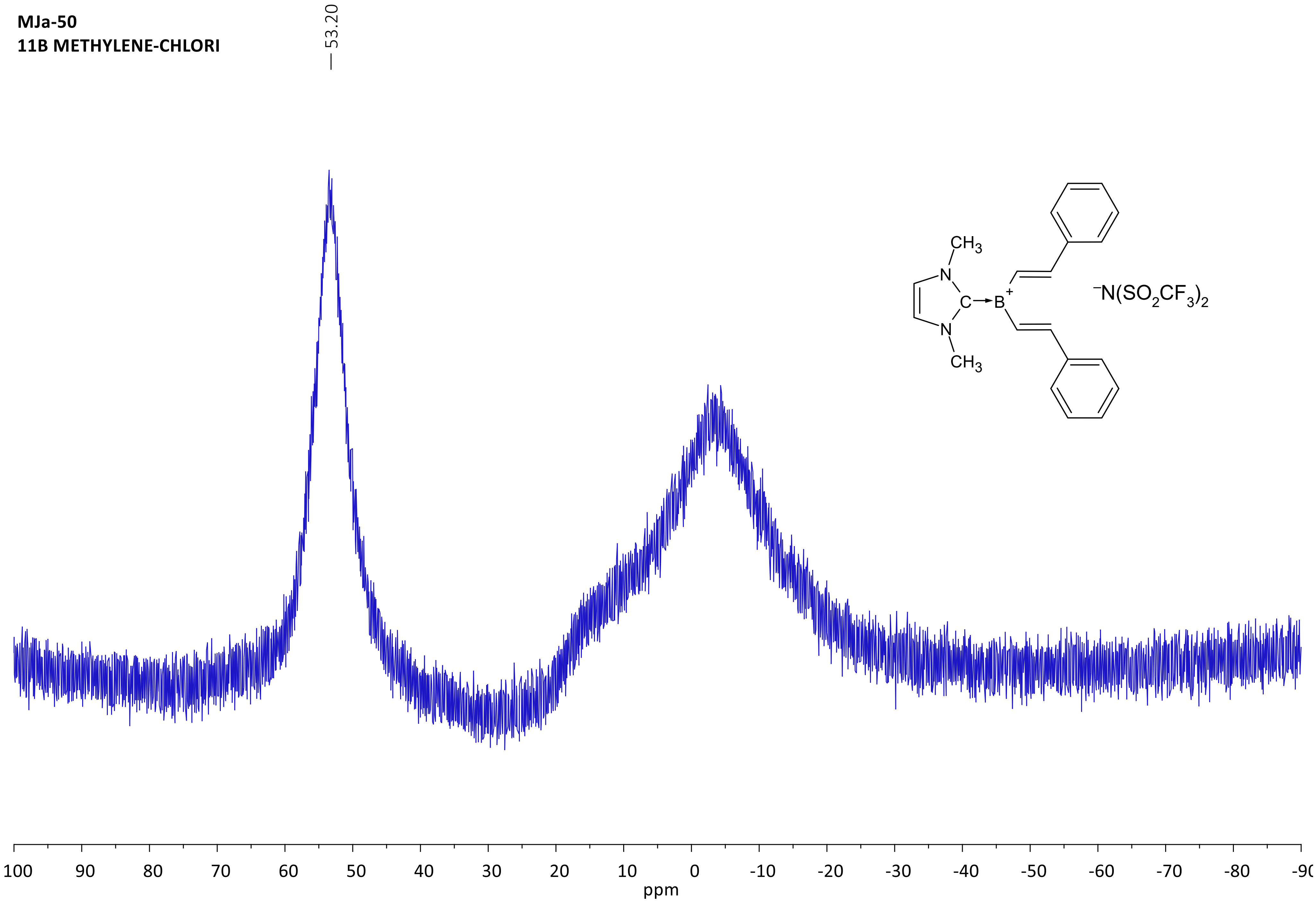

$^{11}B\{^{1}H\}$ NMR for compound ***3d***.

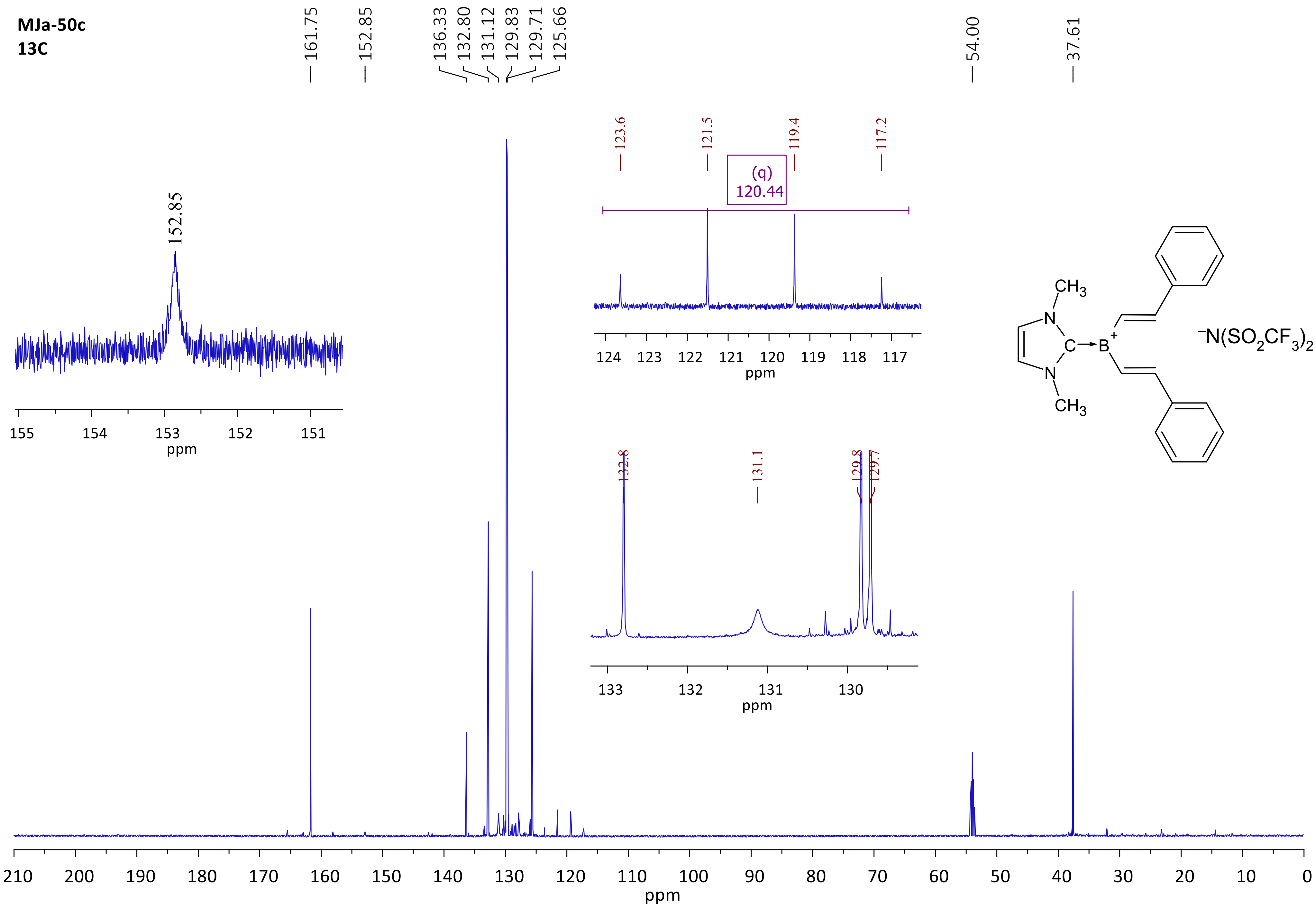

$^{13}C\{^1H\}$ NMR for compound ***3d***.

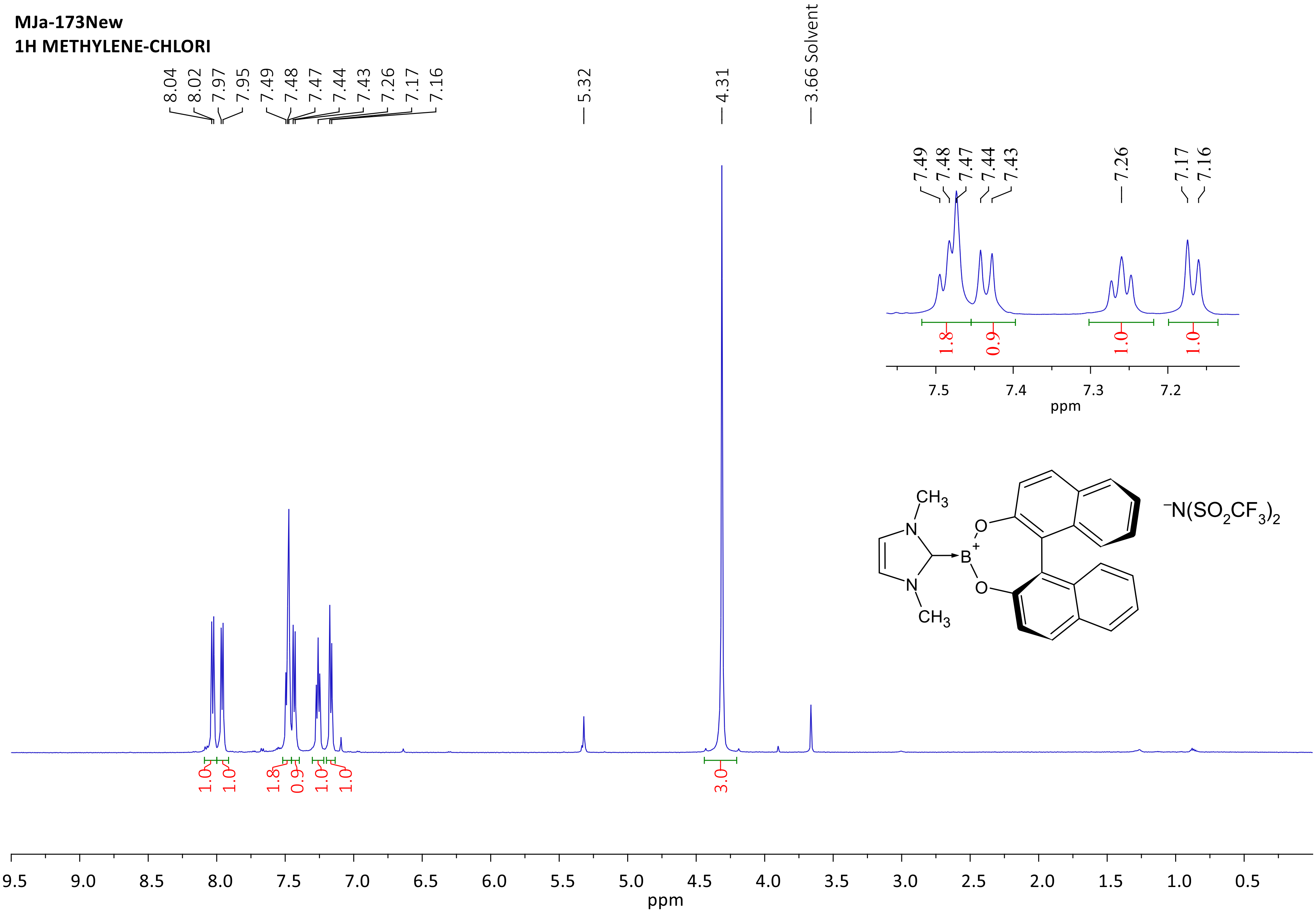

^{1}H NMR for compound ***3e***.

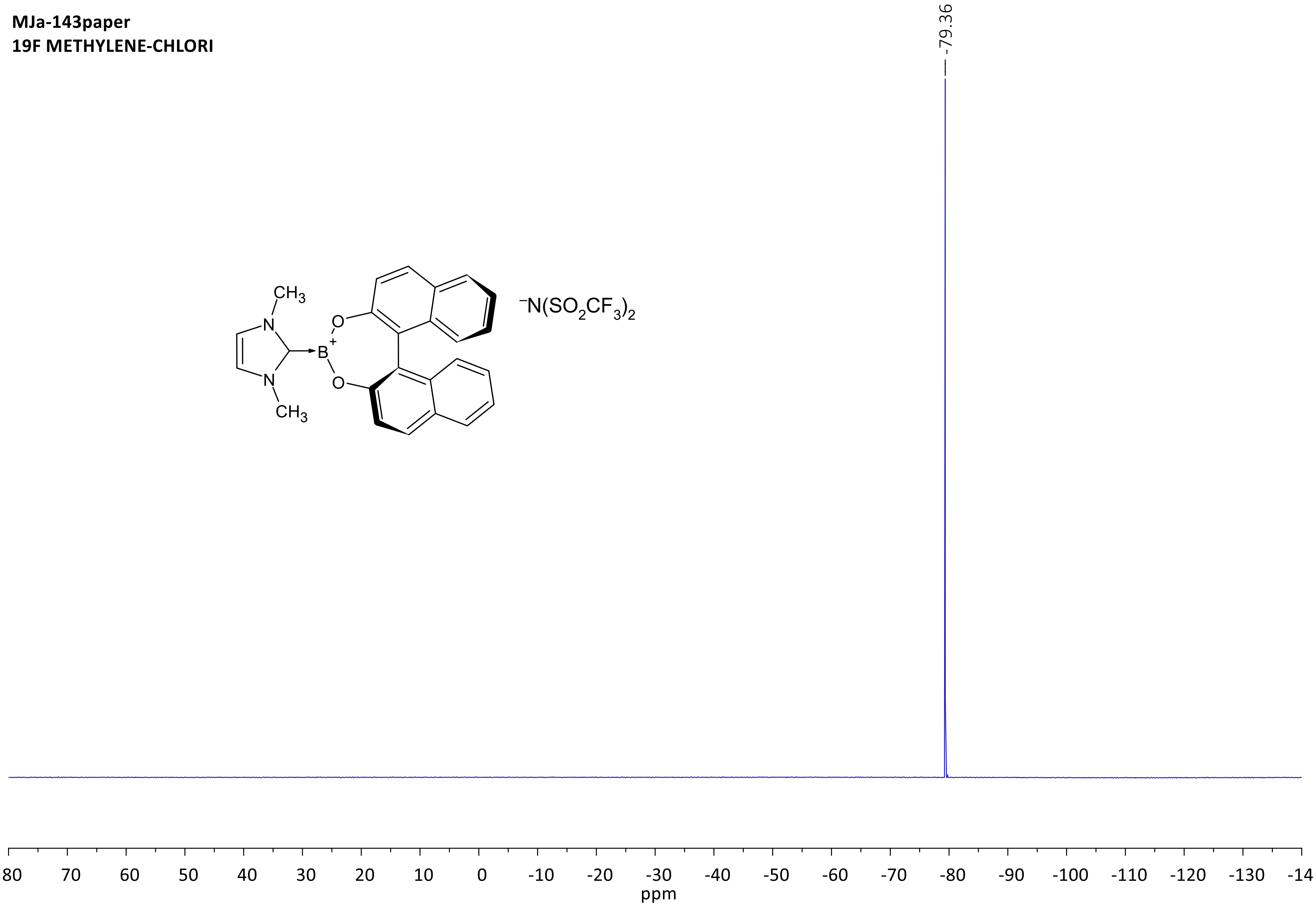

^{19}F NMR for compound ***3e***.

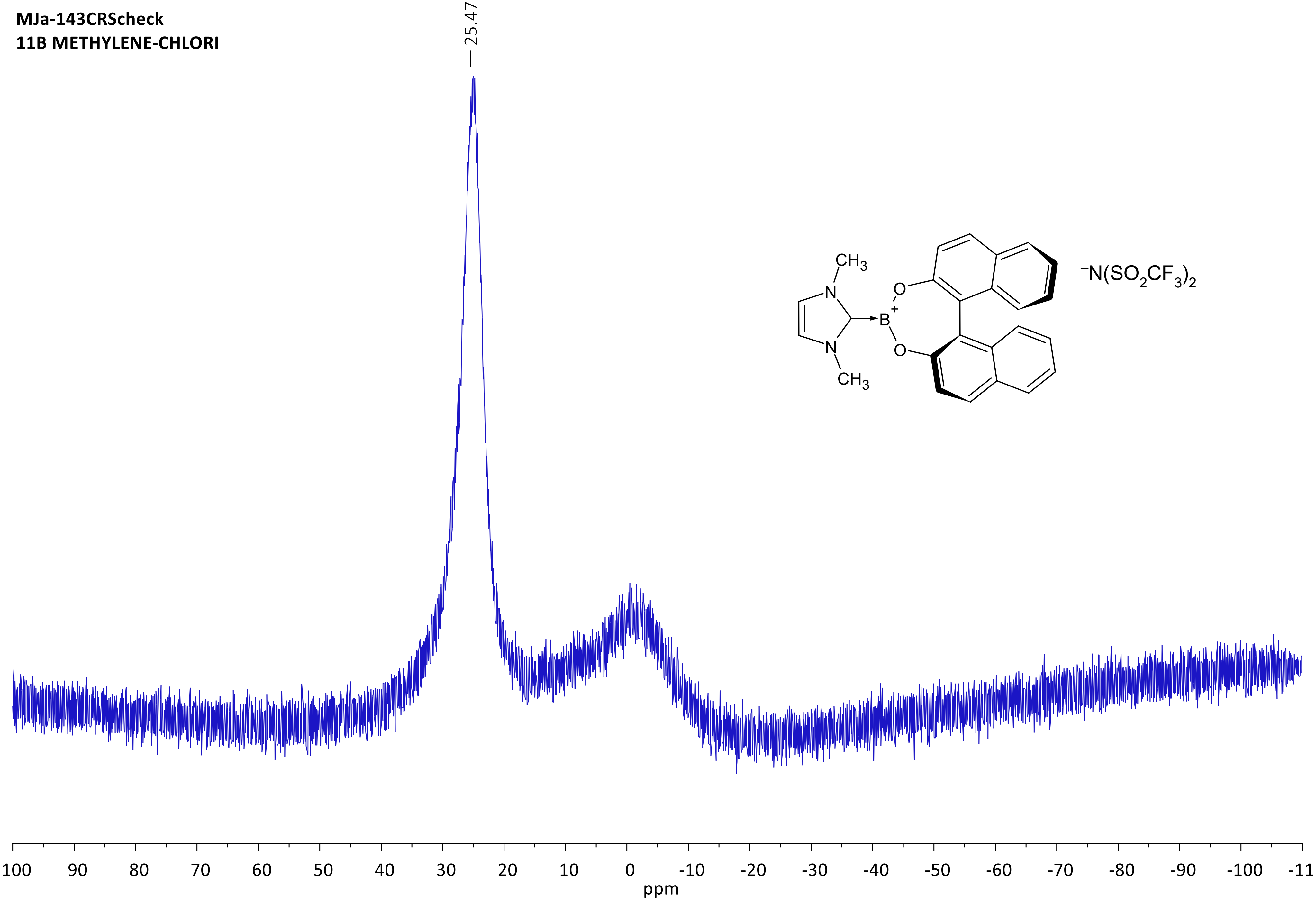

$^{11}B\{^{1}H\}$ NMR for compound ***3e***.

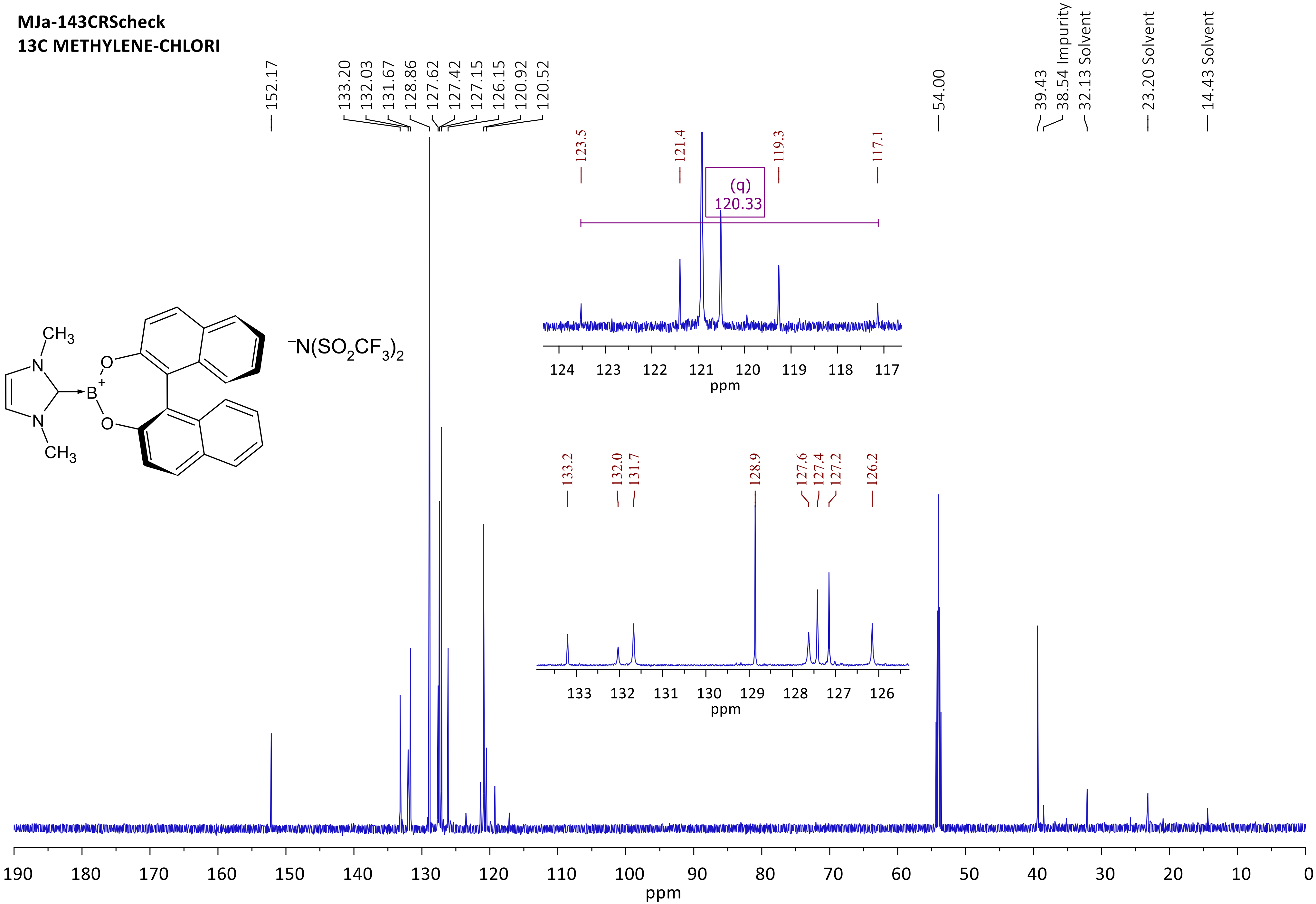

$^{13}C\{^{1}H\}$ NMR for compound ***3e***.

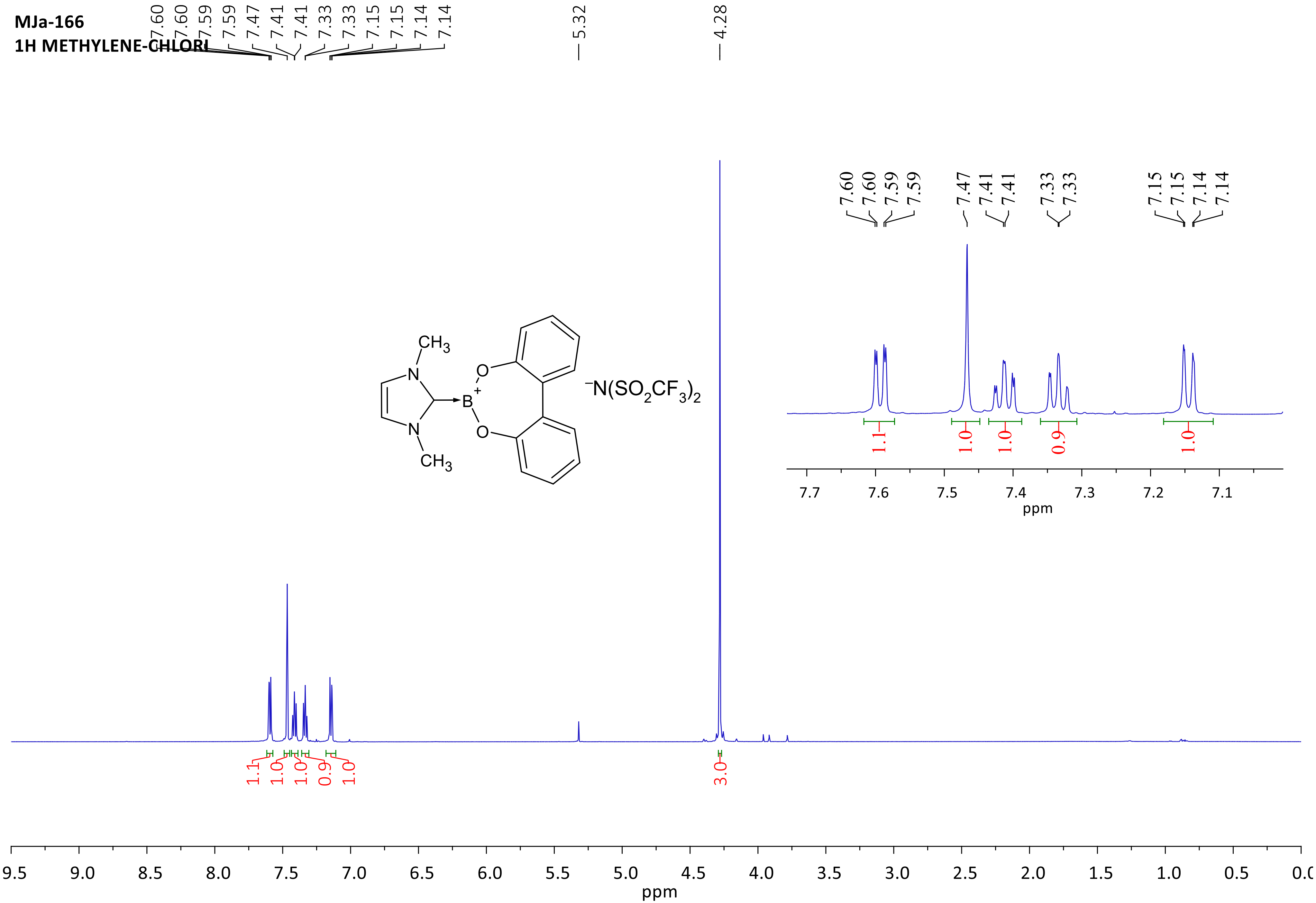

^{1}H NMR for compound ***3g***.

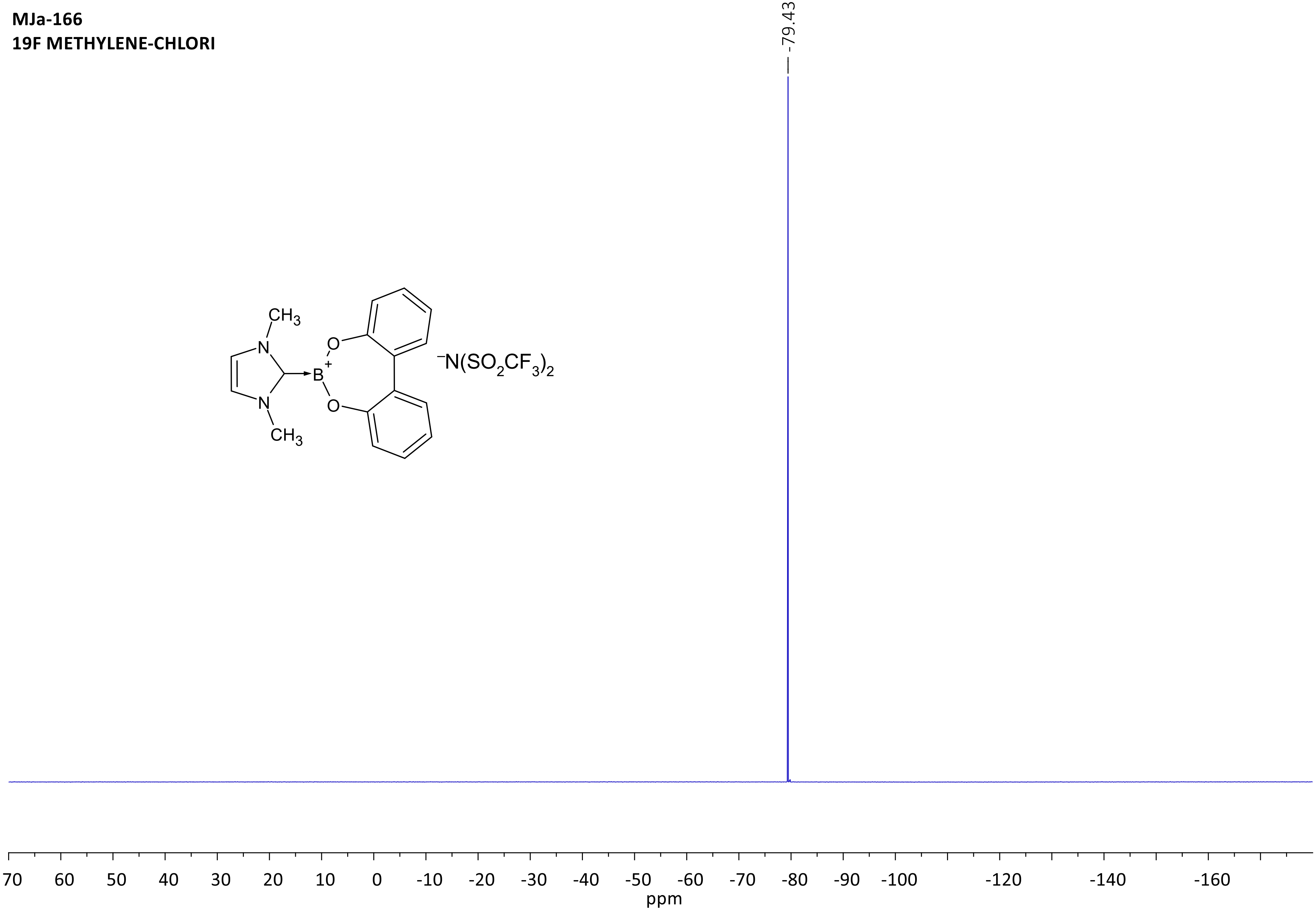

^{19}F NMR for compound ***3g***.

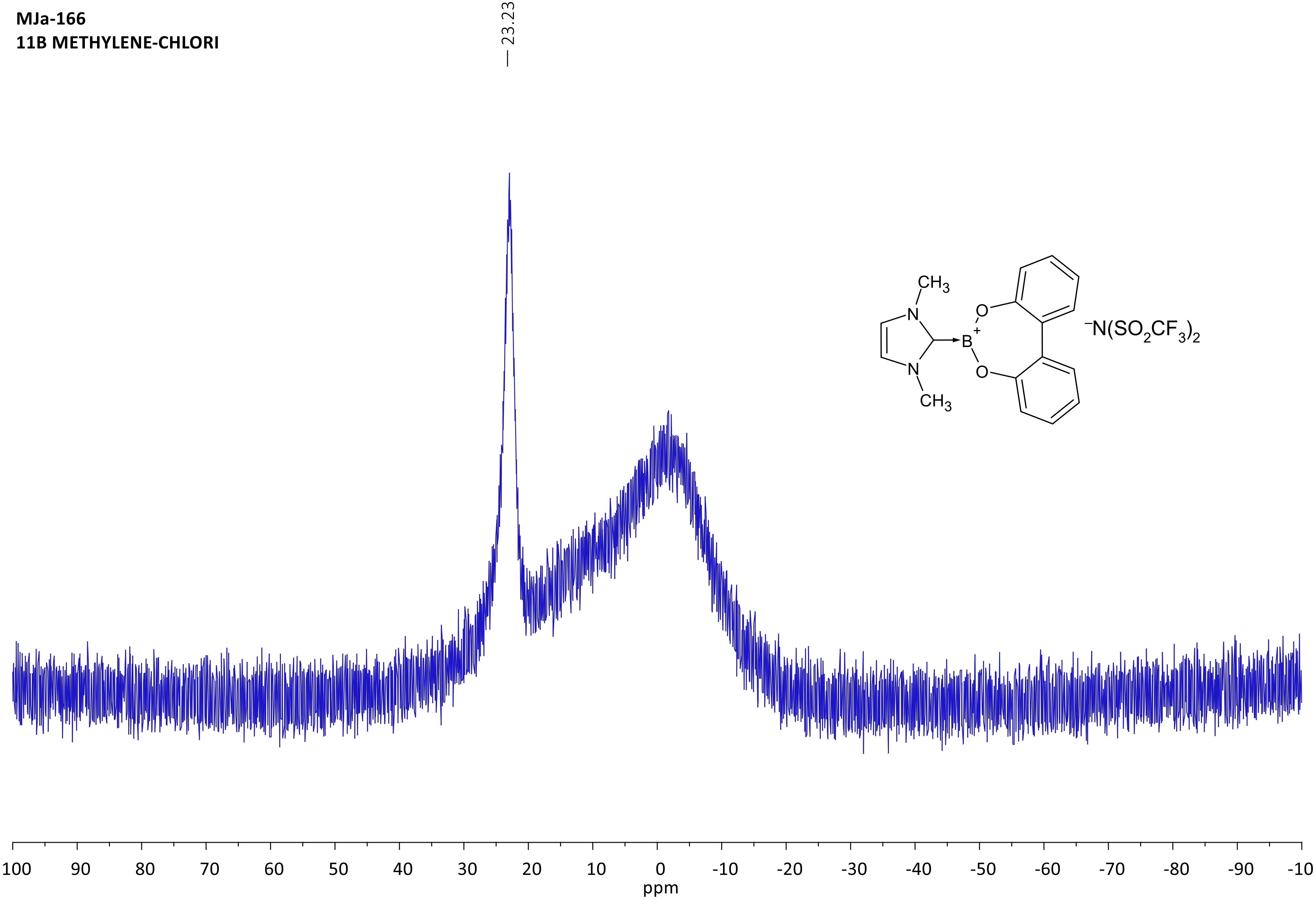

$^{11}B\{^1H\}$ NMR for compound ***3g***.

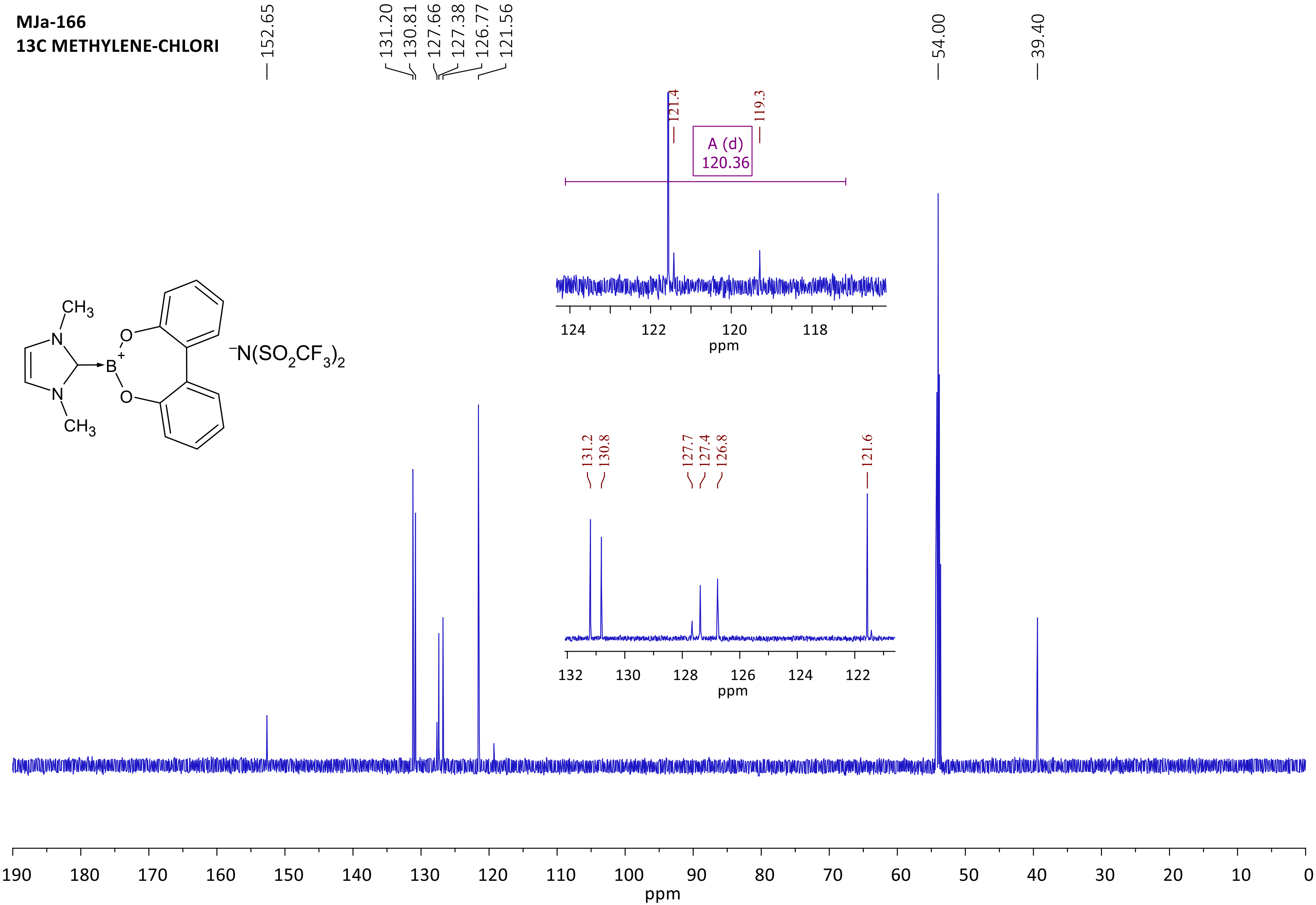

$^{13}C\{^1H\}$ NMR for compound ***3g***.

References

(1) Medici, F.; Gontard, G.; Derat, E.; Lemière, G.; Fensterbank, L. Synthesis of Stable Pentacoordinate Silicon(IV)-NHC Adducts: An Entry to Anionic N-Heterocyclic Carbene Ligands. *Organometallics* **2018**, *37* (4), 517–520. https://doi.org/10.1021/acs.organomet.7b00838.

(2) Lindsay, D. M.; McArthur, D. The Synthesis of Chiral N-Heterocyclic Carbene-Borane and -Diorganoborane Complexes and Their Use in the Asymmetric Reduction of Ketones. *Chem. Commun.* **2010**, *46* (14), 2474–2476. https://doi.org/10.1039/c001466d.

(3) Ono, S.; Watanabe, T.; Nakamura, Y.; Sato, H.; Hashimoto, T.; Yamaguchi, Y. Synthesis of N-Heterocyclic Carbene Boranes via Silver N-Heterocyclic Carbene Complexes. *Polyhedron* **2017**, *137*, 296–305. https://doi.org/10.1016/j.poly.2017.08.045.

(4) Tambutet, G.; Guindon, Y. Diastereoselective Radical Hydrogen Transfer Reactions Using N-Heterocyclic Carbene Boranes. *J. Org. Chem.* **2016**, *81* (22), 11427–11431. https://doi.org/10.1021/acs.joc.6b02066.

(5) Yang, Y.; Han, L.; Taponard, A.; Khrouz, L.; Bucher, C.; Monnereau, C.; Médebielle, M.; Tlili, A. A Shelf-Stable Reagent for Photocatalytic Radical Pentafluorosulfanylation of Styrene Derivatives. *Angew. Chemie - Int. Ed.* **2025**, *64* (31), e202505146. https://doi.org/10.1002/anie.202505146.

(6) Erdmann, P.; Leitner, J.; Schwarz, J.; Greb, L. An Extensive Set of Accurate Fluoride Ion Affinities for P-Block Element Lewis Acids and Basic Design Principles for Strong Fluoride Ion Acceptors. *ChemPhysChem* **2020**, *21* (10), 987–994. https://doi.org/10.1002/cphc.202000244.

(7) Erdmann, P.; Greb, L. Multidimensional Lewis Acidity: A Consistent Data Set of Chloride, Hydride, Methide, Water and Ammonia Affinities for 183 p-Block Element Lewis Acids. *ChemPhysChem* **2021**, *22* (10), 935–943. https://doi.org/10.1002/cphc.202100150.

(8) Li, T.; Zhang, H.; Chan, A. S. C.; Meng, S. S. Manganese-Catalyzed Dehydrogenative Coupling of Silanes and Hydroxyl Compound Controlled by Phosphine Oxide. *Cell Reports Phys. Sci.* **2023**, *4* (3), 101313. https://doi.org/10.1016/j.xcrp.2023.101313.

(9) Mayer, U.; Gutmann, V.; Gerger, W. The Acceptor Number - A Quantitative Empirical Parameter for the Electrophilic Properties of Solvents. *Monatshefte für Chemie* **1975**, *106* (6), 1235–1257. https://doi.org/10.1007/BF00913599.

(10) Gutmann, V. Empirical Parameters for Donor and Acceptor Properties of Solvents. *Electrochim. Acta* **1976**, *21* (9), 661–670. https://doi.org/10.1016/0013-4686(76)85034-7.

(11) Adamczyk-Woźniak, A.; Jakubczyk, M.; Jankowski, P.; Sporzyński, A.; Urbański, P. M. Influence of the Diol Structure on the Lewis Acidity of Phenylboronates. *J. Phys. Org. Chem.* **2013**, *26* (5), 415–419. https://doi.org/10.1002/poc.3102.

(12) Adamczyk-Woźniak, A.; Jakubczyk, M.; Sporzyński, A.; Zukowska, G. Quantitative Determination of the Lewis Acidity of Phenylboronic Catechol Esters - Promising Anion Receptors for Polymer Electrolytes. *Inorg. Chem. Commun.* **2011**, *14* (11), 1753–1755. https://doi.org/10.1016/j.inoche.2011.08.002.

(13) Chen, J.; Lalancette, R. A.; Jäkle, F. Synthesis and Lewis Acid Properties of a Ferrocene-Based Planar-Chiral Borenium Cation. *Chem. Commun.* **2013**, *49* (43), 4893–4895. https://doi.org/10.1039/c3cc41556b.
